# Enantioselective Cobalt(III)-Catalyzed [4 + 1] Annulation of Benzamides: Cyclopropenes as One-Carbon Synthons

**DOI:** 10.1021/jacs.4c16953

**Published:** 2025-04-28

**Authors:** Lenin Kumar Verdhi, Matthew D. Wodrich, Nicolai Cramer

**Affiliations:** † Laboratory of Asymmetric Catalysis and Synthesis, Institute of Chemical Sciences and Engineering, Ecole Polytechnique Fédérale de Lausanne (EPFL), 1015 Lausanne, Switzerland; ‡ Laboratory for Computational Molecular Design, Institute of Chemical Sciences and Engineering, Ecole Polytechnique Fédérale de Lausanne (EPFL), 1015 Lausanne, Switzerland

## Abstract

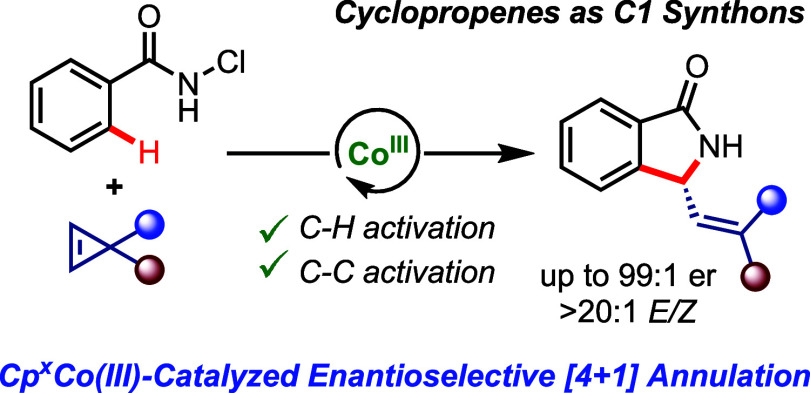

A chiral cyclopentadienyl cobalt(III)-catalyzed enantioselective [4 + 1] annulation of *N*-chlorobenzamides with cyclopropenes is reported. The cobalt catalyst engages in the C–H activation as well as promotes the C–C bond cleavage of the cyclopropene, rendering it as a one-carbon unit for the annulation. The reaction efficiently constructs biologically relevant chiral isoindolinones with selectivities of up to 99:1 er and >20:1 *E*/*Z* ratios. The cobalt(III) catalyst displays a unique orthogonal reactivity profile delivering [4 + 1] annulation products, whereas its rhodium(III) homologue engages in the more classical [4 + 2] annulation pattern. Computational studies reveal the origin of these reactivity divergences.

## Introduction

**Scheme 1:**
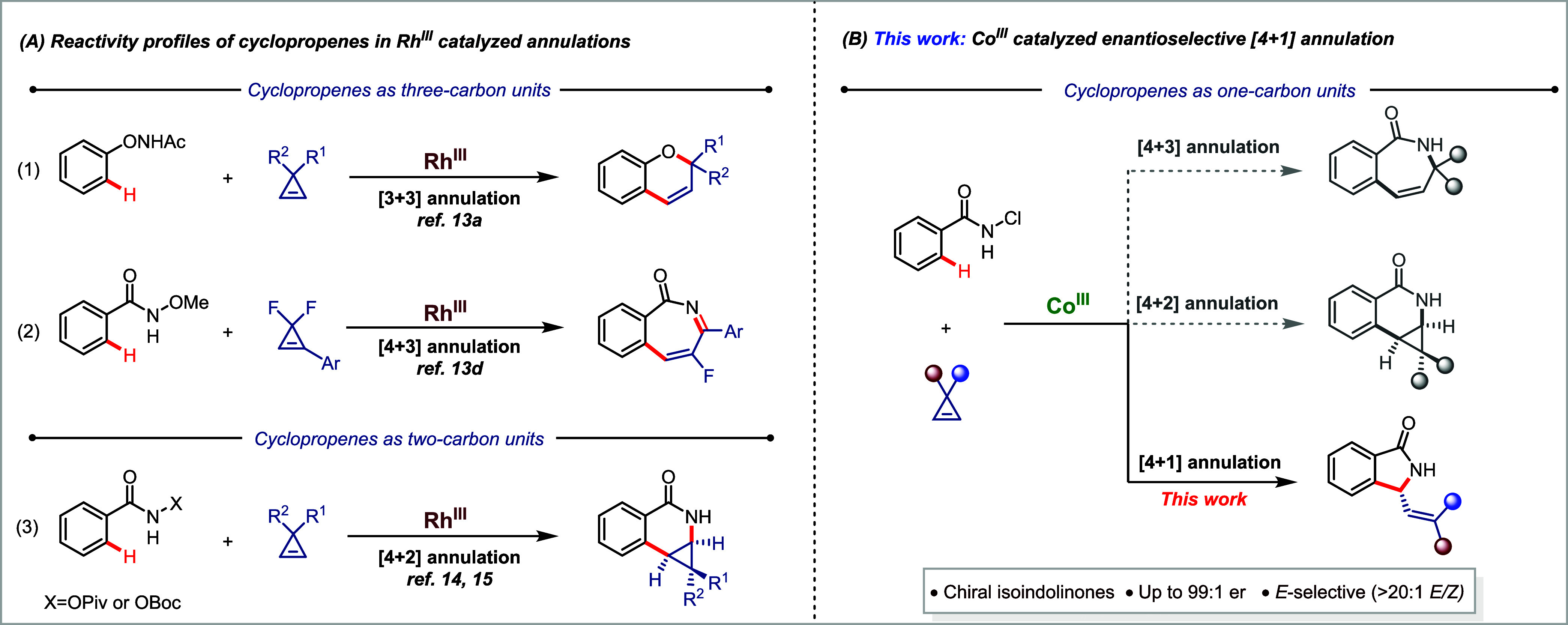
Cyclopropene Reaction Manifold in Transition-Metal-Catalyzed Annulations

Transition-metal-catalyzed asymmetric C–H bond functionalization constitutes a powerful approach for accessing chiral molecules.^1^ While the majority of significant advances in this area have been accomplished with precious 4d and 5d-metal catalysts,^2^ the exploration of inexpensive and earth-abundant 3d-metal catalysts has attracted great attention in recent years.^3^ High-valent cobalt catalysts have emerged as sustainable alternatives to complement the reactivity and selectivity of Rh^III^- and Ir^III^-based catalysts.^4−8^ In this context, Co^III^ complexes^4−6^ bearing chiral cyclopentadienyl (Cp^x^) ligands^9^ have displayed high selectivity levels in enantioselective C–H functionalization. However, the exploitation of Cp^x^Co^III^ complexes for orthogonal reaction profiles compared to that of their Rh and Ir group 9 homologues remains largely underexplored. Cyclopropenes are strained unsaturated cycles and are valuable synthetic building blocks with diverse reactivity profiles.^10^ Besides their reaction profile as simple classical alkenes, cyclopropenes can engage in ring-opening processes in the presence of transition metals, resulting in the formation of the corresponding vinyl metal carbenes.^11^ This unique divergent reactivity enables cyclopropenes to serve as one-, two-, and three-carbon synthons for diverse cycloaddition/annulation reactions.^12^ Despite possessing versatile reactivity, cyclopropenes have been rarely employed as coupling partners in C–H functionalizations, with most of reported examples involving rhodium catalysts.^13−15^ The reaction mode of the cyclopropene largely depends both on its electronic properties and the substrate’s directing group. Wang used cyclopropenes as three-carbon units in achiral Rh^III^-catalyzed transannulation of *N*-phenoxyacetamides (Scheme 1A).^13a^ Yi and Zhou applied *gem*-difluorocyclopropenes as β-monofluorinated three sp^2^-carbon units for the [4 + 3] annulations under Rh catalysis.^13d^ Rovis reported the use of cyclopropenes as coupling partners for Rh^III^-catalyzed diastereoselective [4 + 2] annulations with benzamides, where cyclopropenes display typical olefin reactivity (eq 3, Scheme 1A).^14a^ Waldmann developed an enantioselective version of this transformation using chiral JasCp^x^Rh^III^ catalysts.^15^ However, to our knowledge, cyclopropenes have so far not been used for annulation reactions under Co^III^ catalysis, especially in an enantioselective manner.^16^ Attracted by the broad reactivity profile of cyclopropenes, and in continuation of our pursuit in chiral Cp^x^Co^III^ catalysis for asymmetric C–H functionalization,^4,5^ we aimed to explore the use of cyclopropenes as coupling partners for the annulation of benzamides. Given the unknown reactivity profile of cyclopropenes and *N*-chlorobenzamides under Cp^x^Co^III^ catalysis, two possible reaction pathways could be envisaged. (i) Cobalt catalysts could exhibit similar reactivity to their rhodium homologue, resulting in [4 + 2] annulation products.^14,15^ (ii) Alternatively, the cobalt catalysts could facilitate ring opening of the cyclopropene,^17^ resulting in behavior as either a one- or a three-carbon unit and eventually leading to the corresponding [4 + 1] or [4 + 3] annulation products.

Herein, we disclose an efficient 3d-metal Cp^x^Co^III^-catalyzed enantioselective [4 + 1] annulation of *N*-chlorobenzamides with cyclopropenes (Scheme 1B). This redox-neutral transformation enables the rapid construction of biologically relevant chiral isoindolinones^18,19^ with excellent enantioselectivities. To date, the asymmetric synthesis of chiral isoindolinones via C–H functionalization has been limited to Rh(III) catalysts, most frequently in conjunction with diazo, alkyne, and alkene precursors.^20^ In contrast to previous reports of the behavior of cyclopropenes under rhodium(III) catalysis,^14,15^ the present work demonstrates that cobalt(III) catalysis selectively triggers the ring opening of cyclopropenes, rendering them as one-carbon units for an enantioselective annulation process. Additionally, computational studies are used to explore the divergent behaviors of Co(III) and Rh(III) catalysts by analyzing the underlying mechanism of the cyclopropene ring opening.

## Results and Discussion

### Reaction Optimization

We started our feasibility investigation of the [4 + 1] annulation using *N*-chlorobenzamide **1a** and 3,3-disubstituted cyclopropene **2a** as model substrates (Table 1). Preliminary reaction scouting using achiral Cp*Co(CO)I_2_ catalyst, silver triflate as halide scavenger, and sodium acetate as a CMD-promoting base resulted in the formation [4 + 1] annulated isoindolinone **3aa** in 36% yield as the exclusive reaction product (entry 1). The transformation was performed in a TFE/DCE at 30 °C (for optimization details, see the Supporting Information (SI)). Of note, neither [4 + 2] nor [4 + 3] annulation products were observed during this reaction. The use of chiral catalyst **Co1** bearing a disubstituted binaphthyl-derived Cp^x^-ligand was not competent for the transformation and no product **3aa** was formed (entry 2). In contrast, catalyst **Co2** having a trisubstituted Cp^x^-ligand (R = *i*Pr) provided [4 + 1] annulation product **3aa** in 43% yield and 81.5:18.5 er (entry 3). Notably, the (*E*)-olefin geometry was exclusively observed. Increasing the size of Cp^x^ substituent R from *i*Pr to *t*Bu (**Co3**) drastically improved the selectivity of **3aa** to 96:4 er (entry 4). Similarly, catalyst **Co4** also delivered isoindolinone **3aa** in 95:5 er, albeit in a moderate yield (entry 5). Attempts to improve the yield of **3aa** with catalyst **Co3** by employing alternative halide scavengers such as AgPF_6_, AgSbF_6_, and AgOBz were not successful (entries 6–8). Using an additive combination of silver benzoate and sodium carbonate improved the yield of **3aa** to 55% while maintaining an er of 94:6 (entry 9). Increasing the amount of cyclopropene boosted the yield of **3aa** to 65% (entry 10). Under identical conditions, **Co4** furnished isoindolinone **3aa** in 71% yield with 95:5 er (entry 11). Excluding silver salt from the reaction mixture and using an additive combination of benzoic acid resulted in a slightly improved yield of 74% with an excellent enantioselectivity of 97:3 er (entry 12). Reducing the cobalt catalyst loading to 5 mol % did not affect the enantioselectivity, but did cause a slight reduction in the reaction yield (entry 13). Single-crystal X-ray crystallographic analysis of isoindolinone **3aa** allowed the determination of the absolute configuration to be (*R*) and the double-bond geometry to be (*E*).^21^

**Table 1:**
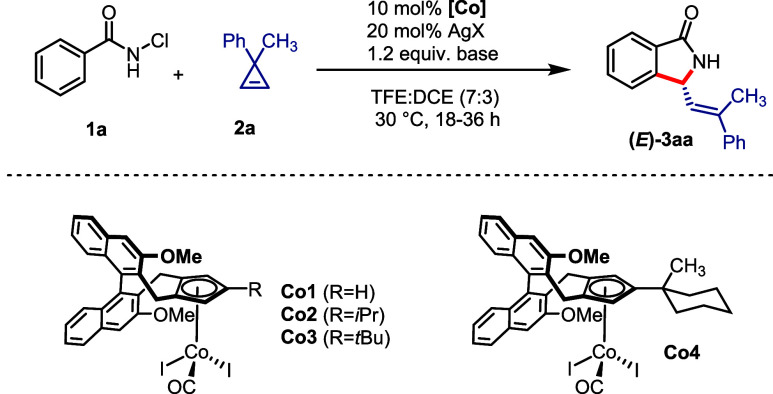
Optimization of the [4 + 1] Annulation^a^

entry	**[Co]**	AgX	base	(*E*)-**3aa** (%)	er
1	Cp*Co	AgOTf	NaOAc	36	na
2	**Co1**	AgOTf	NaOAc	0	na
3	**Co2**	AgOTf	NaOAc	43	81.5:18.5
4	**Co3**	AgOTf	NaOAc	34	96:4
5	**Co4**	AgOTf	NaOAc	23	95:5
6	**Co3**	AgPF_6_	NaOAc	33	95:5
7	**Co3**	AgSbF_6_	NaOAc	28	95:5
8	**Co3**	AgOBz	NaOAc	33	94:6
9^b^	**Co3**	AgOBz	Na_2_CO_3_	55	94:6
10^c^	**Co3**	AgOBz	Na_2_CO_3_	65	96:4
11^c^	**Co4**	AgOBz	Na_2_CO_3_	71	95:5
12^d^	**Co4**		BzOH	74	97:3
			Na_2_CO_3_		
13^e^	**Co4**		BzOH	61	97:3
			Na_2_CO_3_		

a Conditions: 50 μmol **1a**, 100 μmol **2a**, 5.0 μmol **Co**, 10.0 μmol AgX, 1.2 equiv NaOAc, 0.10 M of TFE/DCE (7:3), at 30 °C for 18–36 h. Yields were determined by ^1^H NMR using 1,3,5-trimethoxybenzene as an internal standard.

b 0.5 equiv Na_2_CO_3_.

c 200 μmol **2a**, 5.0 μmol **Co**, 10 μmol AgOBz, 0.5 equiv Na_2_CO_3_, 0.10 M TFE:DCE (1:1),23 °C for 24 h.

d 200 μmol **2a**, 5.0 μmol **Co**, 10 μmol benzoic acid, 0.6 equiv Na_2_CO_3_, 0.10 M TFE:DCE (1:1), 36 h.

e 2.5 μmol **Co**, 5.0 μmol benzoic acid, 0.6 equiv Na_2_CO_3_, 0.10 M TFE:DCE (1:1), 36 h.

### Substrate Scope

**Scheme 2:**
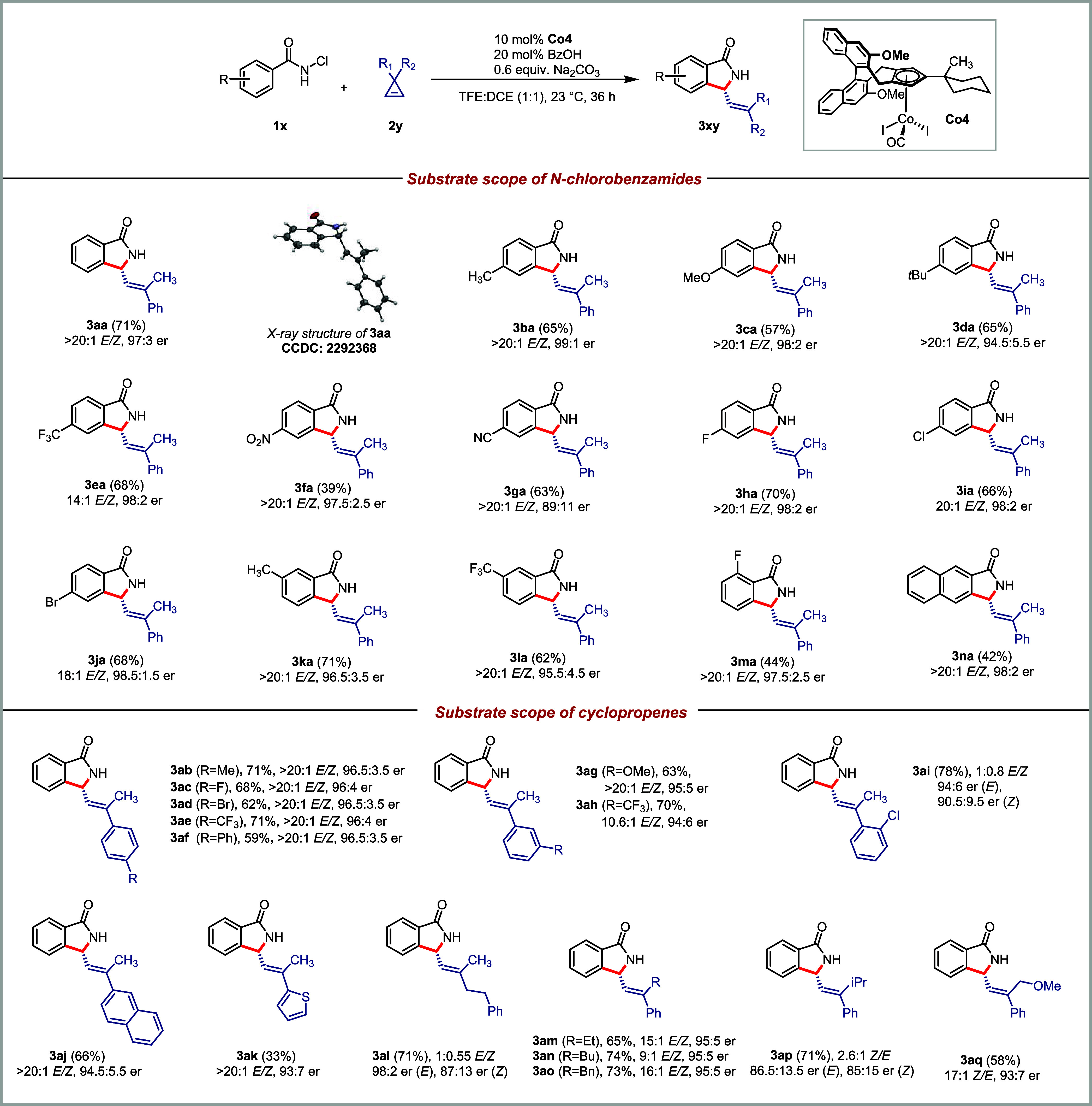
Scope for the Cp^x^Co^III^-Catalyzed Enantioselective [4 + 1] Annulation of Benzamides with Cyclopropenes Conditions: 0.1 mmol **1x**, 0.4 mmol **2y**, 10 mol % **Co4**, 20 mol % BzOH, 60 mol % Na_2_CO_3_, 0.10 M TFE:DCE (1:1), 23 °C, 36 h. Isolated yields. Enantiomeric ratios were determined by chiral HPLC.

Under optimized reaction conditions, the generality of the reaction was investigated (Scheme 2). We first explored the electronic effects of different substituents on *N*-chlorobenzamides. Benzamides bearing electron-donating groups at the *para*-position (**1b**–**1d**) underwent reaction with cyclopropene **2a** to produce the corresponding isoindolinones **3ba-3da** in good yields and excellent enantiomeric ratios. The electron-withdrawing group *p-*CF_3_-substituted benzamide **3e** reacted with **2a**, yielding the product **3ea** with an excellent enantioselectivity of 98:2 er. Benzamide with a strong electron-withdrawing nitro group **1f** yielded the product **3fa** in 97.5:2.5 er, albeit in reduced yield. The coordinating nature of the cyano group (**1g**) led to lower enantioselectivity for the product **3ga**. Additionally, benzamides having *para*-halogen substitution (**1h**–**1j**) were tolerated under the reaction conditions, delivering the corresponding products with high enantioselectivity. Benzamides with *meta*-substituents (**1k**–**1l**) were compatible and afforded the desired annulation compounds exclusively as a single regioisomer. *Ortho*-fluoro-substituted benzamide **1m** reacted smoothly with cyclopropene to yield product **3ma** in an excellent enantioselectivity of 97.5:2.5 er. *N*-Chloronaphthamide **1n** was successfully coupled with cyclopropene, yielding the product in excellent enantioselectivity of 98:2 er.

**Scheme 3:**
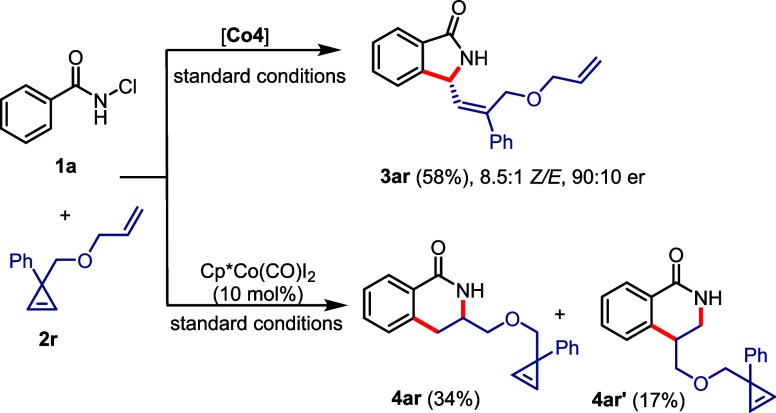
Cp^x^ vs Cp* Ligand Effect on the Reactivity and Selectivity of the Annulation Process

Next, we turned our focus to evaluating the scope of cyclopropenes. A diverse array of cyclopropenes were compatible with the reaction conditions and engaged in the [4 + 1] annulation. Regardless of the electronic nature of substituents at the *para*-position of the aromatic ring, cyclopropenes (**2b**–**2f**) reacted with *N*-chlorobenzamide **1a** to produce the corresponding isoindolinones with good yields, high enantioselectivities, and excellent *E*/*Z* ratios. Cyclopropenes bearing an electron-donating or -withdrawing group at the *meta*-position of the aryl group (**2g**–**2h**) robustly engaged in the transformation. Cyclopropene **2i** having an *ortho*-Cl phenyl substitution maintained good reactivity and enantioselectivity despite a loss of *E*/*Z* selectivity. Naphthyl-substituted cyclopropene **2j** yielded isoindolinone **3aj** in 66% yield with 94.5:5.5 er. Cyclopropene **2k** bearing a 2-thienyl group reacted to produce **3ak** with a slightly reduced enantioselectivity of 93:7 er. Cyclopropene **2l** with ethylbenzene substitution instead of aromatic ring provided product **3al** in good yield albeit with a weak *E*/*Z* selectivity. The enantiomeric ratio of *E*-**3al** was 98:2, whereas *Z*-**3al** was formed with 87:13 er. Changing the methyl group of cyclopropenes to ethyl, butyl, and benzyl (**2m**–**2o**) were competent under reaction conditions and delivered the desired products in good yields and enantioselectivities. Cyclopropene with a sterically demanding isopropyl group **2p** underwent the reaction and provided isoindolinone **3ap** with slightly reduced enantio- and *E*/*Z* selectivity. Cyclopropene bearing a methoxy group at a potentially coordinating distance (**2q**) engages in the transformation, delivering the product **3aq** with enantioselectivity of 93:7 er. We observed a very strong influence of the cyclopentadienyl ligand on the reactivity and selectivity of the annulation process. For instance, when cyclopropene **2r** bearing an additional terminal olefin moiety was subjected to the reaction conditions, [4 + 1] annulation product **3ar** was exclusively obtained with the chiral **Co4** catalyst (Scheme 3). With this Cp^x^ ligand, the coordination and migratory insertion of cyclopropene is faster than that of the terminal olefin. In stark contrast, the achiral Cp*Co(CO)I_2_ complex left the cyclopropene unit completely untouched. It reacted instead selectively with the terminal olefin moiety, leading to a 2:1 mixture of regioisomeric dihydroisoquinolones **4ar** and **4ar′** in the [4 + 2] annulation mode.

**Scheme 4:**
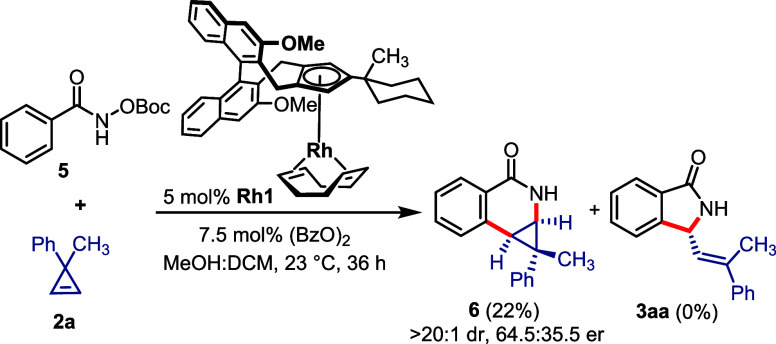
Cp^x^Rh^III^-Catalyzed Enantioselective [4 + 2] Annulation of Benzamide with Cyclopropene

To illustrate the striking reactivity difference between cobalt and rhodium catalysts, we performed the annulation of hydroxamate **5** and cyclopropene **2a** with 5 mol % **Rh1** equipped with same trisubstituted chiral Cp^x^ ligand (Scheme 4). Following Waldmann’s report,^15^ exposing *N*-OBoc-benzamide **5** and cyclopropene **2a** to catalyst **Rh1** yielded only [4 + 2] annulation compound **6**. No ring opening of cyclopropene and no subsequent [4 + 1] annulation product was observed under this rhodium catalysis. The reaction outcome underscores the unique reactivity profile of cobalt in the ring opening of cyclopropenes, ultimately resulting in the [4 + 1] annulation product. Under the same reaction conditions, *N*-chlorobenzamides did not provide either [4 + 1] or [4 + 2] annulation products.

**Scheme 5:**
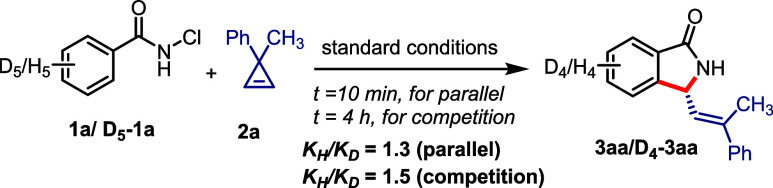
Kinetic Isotope Effect

To obtain insights into the mechanism and critical catalytic steps of the [4 + 1] annulation reaction, kinetic isotope effect (KIE) experiments were conducted (Scheme 5). Both parallel and competitive studies on the **Co4**-catalyzed annulation of **1a** and deuterated **1a** with cyclopropene **2a** showed lower KIE values (*K*_H_/*K*_D_ = 1.3–1.5) (see SI). These results suggest that C–H activation of *N*-chlorobenzamide **1a** may not be involved in the turnover-limiting step of the annulation process.

## Computational Studies

**Figure 1:**
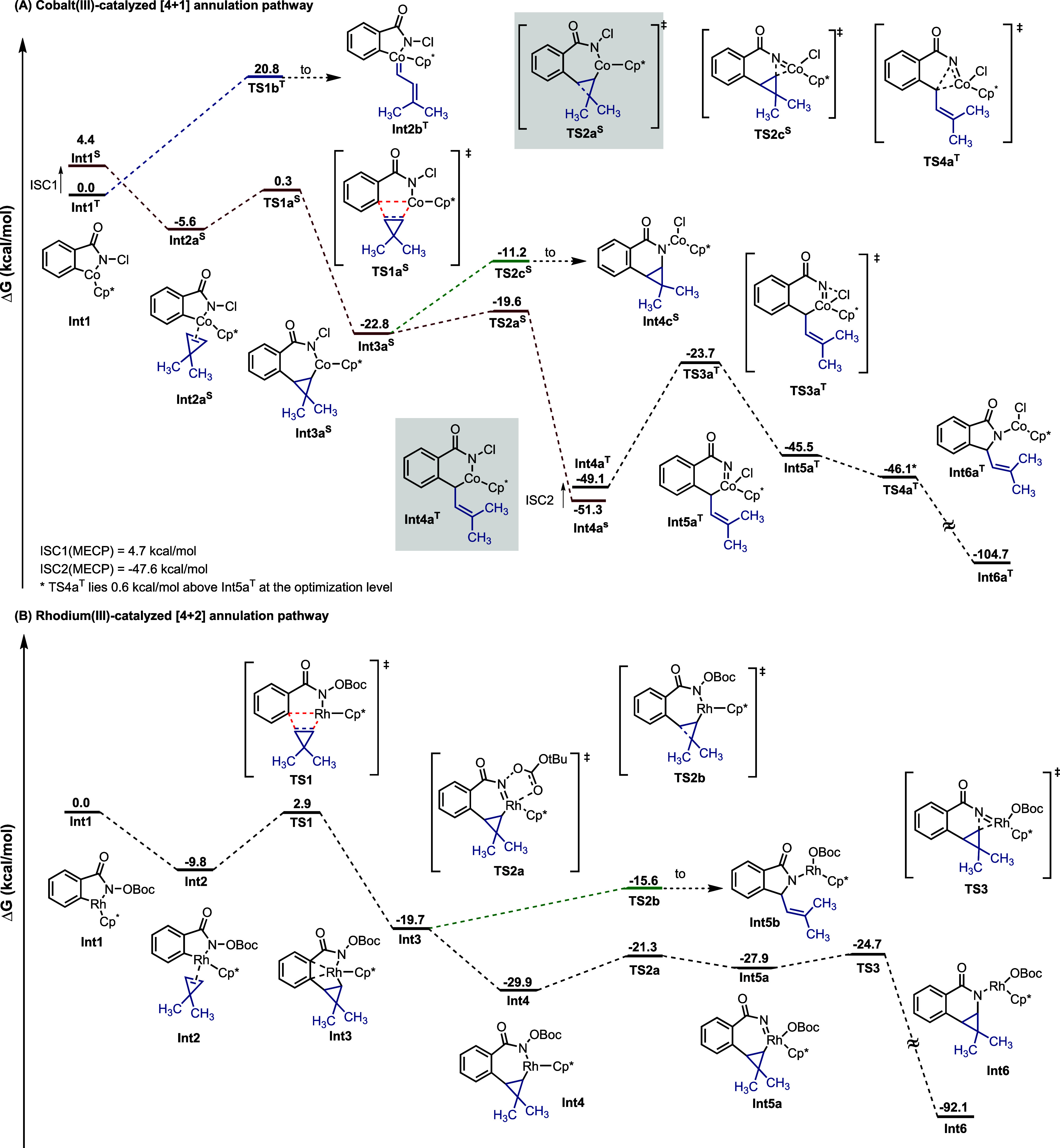
Potential energy surfaces for C–H activation/cyclopropene insertion processes by Co and Rh catalysts computed at the B3PW91-D3(BJ)/def2-TZVP//B3PW91-D3(BJ)/def2-SVP theoretical level in an implicit TFE solvent.

To gain mechanistic insights into the differences of the reaction profiles of Co vs Rh catalysis,^22^ we turned to DFT computations at the B3PW91-D3(BJ)/def2-TZVP//B3PW91-D3(BJ)/def2-SVP level in implicit 2,2,2-trifluorethanol solvent using the SMD model using Gaussian 16 (see below for full computational details) to explore possible reaction pathways. Specifically, we were interested in unraveling the origin of the observed [4 + 1] vs [4 + 2] selectivity difference seen in the employed cobalt and rhodium catalysts and how this relates to cyclopropene ring opening. To focus specifically on these reactivity differences, for cobalt catalysis, we employed *N*-chlorobenzamide **1a** and dimethyl cyclopropene as model substrates along with achiral Cp*Co^III^ as the active catalyst and computed potential mechanistic pathways on both the singlet and triplet potential energy surfaces. Note that the use of this achiral Cp* ligand eliminates the need for large numbers of computations associated with fully mapping the conformational space of bulky chiral ligands,^23^ while still allowing the source of the observed reactivity to be probed. As in previous computational studies,^24^ aza-cobaltacycle intermediate **Int1**^**T**^, which is obtained by sequential N–H deprotonation/C–H activation of **1a** via the CMD process, was chosen as the starting point and assigned a reference ΔG value of 0.0 kcal/mol (Figure 1A).

Here, only the lowest-energy pathways are presented, which involve transitions between the singlet and triplet potential surfaces; the full profiles on both the singlet and triplet potential energy surfaces can be found in the SI (Figure S1). From **Int1**, two possible reaction pathways were envisioned. The first involves migratory insertion of cyclopropene into the Co–C bond to produce seven-membered cobaltacycle intermediate **Int3a**^**S**^ via **Int2a**^**S**^ and **TS1a**^**S**^, while the second involves π-activation of the cyclopropene double bond by electrophilic Co(III) to generate the cobalt carbenoid species **Int2b**^**T**^ via **TS1b**^**T**^. Our computations revealed that the lowest-energy pathway leading to formation of the cobalt carbenoid species **Int2b**^**T**^ [Δ*G*^‡^(**TS1b**^**T**^) = 20.8 kcal/mol] lies 20.5 kcal/mol higher than the migratory insertion pathway leading to a seven-membered cobaltacycle **Int3a**^**S**^ [Δ*G*^‡^(**TS1a**^**S**^) = 0.3 kcal/mol]. As such, we were able to rule out the reaction mechanism involving Co-carbene and focused on the migratory insertion route via **Int3a**^**S**^. From **Int3a**^**S**^, two alternative pathways leading to either [4 + 2] or [4 + 1] annulation products exist. Here, we found the route proceeding by reductive elimination (**TS2c**^**S**^, Δ*G*^‡^ = −11.2 kcal/mol) that ultimately leads to the [4 + 2] product to be less energetically favorable than cyclopropane ring opening (**TS2a**^**S**^, Δ*G*^‡^ = −19.6 kcal/mol) leading to **Int4a**^**S**^. Given that ring opening to **Int4a**^**S**^ is both exothermic and possesses a significant kinetic preference over reductive elimination, formation of the [4 + 1] annulation product should be favored. To reach the final product, **Int4a**^**S**^ would undergo an ISC process to its triplet state **Int4a**^**T**^. As the N–Cl bond is an internal oxidant, the oxidation of Co(III) center in **Int4a**^**T**^ via **TS3a**^**T**^ would lead to the formation of a relatively unstable Co(V)-nitrenoid intermediate **Int5a**^**T**^ (for other examples of Co(V) species in the literature, see ref (25)), which readily undergoes nitrene insertion and protonation of the Co–N bond to give the [4 + 1] annulation product. Examining a similar pathway for Rh catalysis reveals key differences from Co catalysis (Figure 1B). Starting from five-membered rhodacycle **Int1**, the coordination of cyclopropene (**Int2**) and ensuing migratory insertion into the Rh–C via **TS1** leads to the seven-membered rhodacycle **Int4**. At this stage, the energetics of the two possible reaction pathways leading to the [4 + 2] and [4 + 1] annulation products could be established. Here, the ring opening of cyclopropane to generate five-membered intermediate **Int5b** via **TS2b** (Δ*G*^‡^ = −15.6 kcal/mol) was found unfavorable relative to the formation of Rh-nitrenoid intermediate **Int5a** via **TS2a** (Δ*G*^‡^ = −21.3 kcal/mol), leading to the [4 + 2] annulation product. Notably, the carbonyl group of the Boc-moiety coordinates with the Rh-metal in **TS2a**, which facilitates the formation of Rh-nitrenoid intermediate **Int5a** relative to the cyclopropane ring-opening **Int5b** that leads to the [4 + 1] annulation product. The different natures of the employed internal oxidants of the substrates (OBoc for Rh and Cl for Co) as well as the specific manner they possibly can coordinate may contribute to the divergent reactivity observed in rhodium’s [4 + 2] annulation compared to cobalt’s [4 + 1] annulation. Moreover, analysis of partial charges (see SI Table S13 for details) indicates a more pronounced difference between the positive charge on the metal center and the charge on the internal oxidant group in the rhodium system (**Int4**) compared to the cobalt system (**Int3a**^**S**^), which may allow easy cleavage of the N–O bond to form the nitrenoid intermediate **Int5a**. From **Int5a**, the [4 + 2] annulation product would be obtained by nitrene insertion followed by Rh–N bond protonation.

### Mechanistic Proposal

**Scheme 6:**
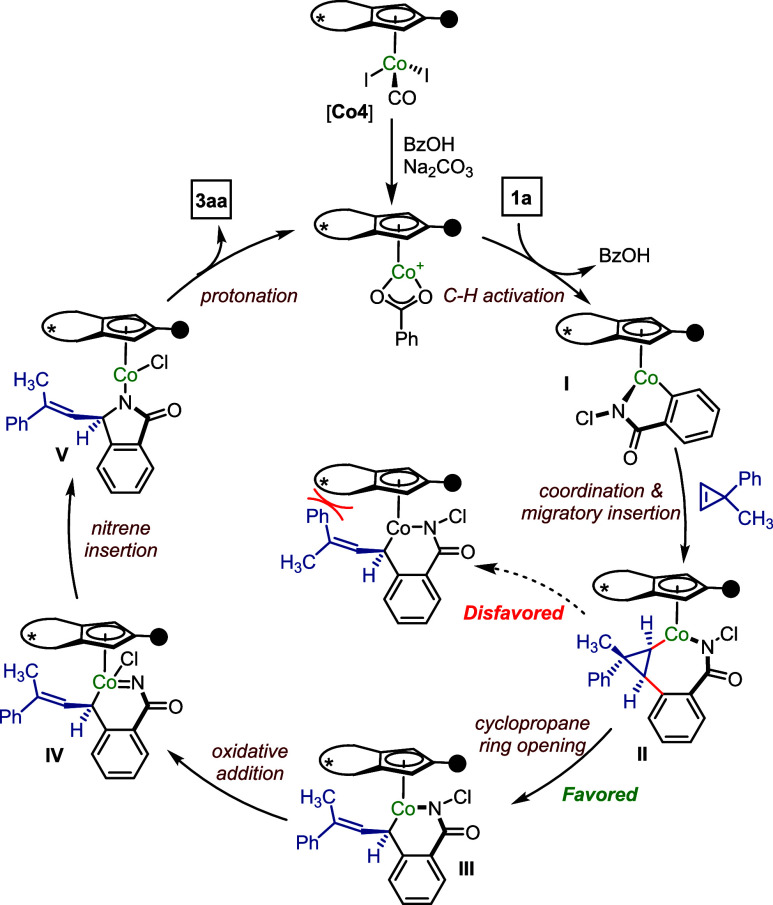
Proposed Mechanism

Based on computational mechanistic studies, we propose the following catalytic cycle (Scheme 6). The catalytic cycle begins with the initial activation of cobalt catalyst **Co4**, followed by the C–H activation of *N*-chlorobenzamide **1a**, which affords cobaltacycle **I**. The facial selective coordination of cyclopropene and subsequent migratory insertion generates bicyclic metallacycle **II**. The ensuing cobalt-induced ring opening of cyclopropane leads to the thermodynamically favorable (*E*)-olefin geometry of intermediate **III**. In contrast, the pathway leading to (*Z*)-olefin geometry is less favorable due to possible steric hindrance between the phenyl group of cyclopropene and the binaphthyl backbone of the chiral **Co4** catalyst. The aza-cobaltacycle intermediate **III** undergoes oxidative addition to generate Co-nitrene species **IV**. Finally, nitrene insertion and Co–N protonation results in the formation of chiral isoindolinone **3aa**.

## Conclusions

In summary, we have successfully demonstrated a Cp^x^Co^III^-catalyzed enantioselective [4 + 1] annulation approach for the coupling of *N*-chlorobenzamides with cyclopropenes while also showcasing the use of cyclopropenes as one-carbon synthons for an asymmetric annulation process. The cobalt catalyst displays a unique ability to engage in the C–H activation step as well as to promote C–C bond activation for the cyclopropene ring opening. The distinct transformation enables the rapid construction of biologically relevant chiral isoindolinones with excellent enantioselectivities of up to 99:1 er. The method showcases a unique and orthogonal reaction profile of the Cp^x^Co^III^ catalyst compared to its rhodium homologues and aids in fostering an understanding of the intriguing reactivity differences of the catalytically prolific group 9 metals.

### Computational Details

The geometries of all species were optimized in the gas phase at the B3PW91^26^-D3(BJ)^27^/def2-SVP^28^ level as implemented in Gaussian16.^29^ Relevant species were characterized as either minima (zero imaginary frequencies) or transition states (one imaginary frequency) on the potential energy surface through examination of vibrational frequencies. Refined energy estimates were obtained by computing single point energies on the optimized B3PW91-D3(BJ)/def2-SVP geometries at the B3PW91-D3(BJ)/def2-TZVP^28^ level that included solvation corrections (in 2,2,2-trifluoroethanol) using the SMD solvation model.^30^ Free energy corrections were determined using the quasi rigid-rotor harmonic oscillator model^31^ and corrected from translational entropy in solution^32^ following the approach proposed by Martin, Hay, and Pratt^33^ (13.24 mol/L in 2,2,2-trifluoroethanol) as implemented in the Goodvibes package.^34^ Reported free energies found in this article include electronic energies at the B3PW91-D3(BJ)/def2-TZVP//B3PW91-D3(BJ)/def2-SVP level along with free energy corrections at the B3PW91-D3(BJ)/def2-SVP level. The accuracy of the employed def2-TZVP basis set for single/triplet splitting was ensured by comparisons with other basis sets (see the SI). B3PW91-D3(BJ)/def2-TZVP level computations to obtain the oxidation state (SI Table S6) of the transition metal employed the localized orbital bonding analysis (LOBA) method proposed by Head-Gordon^35^ as implemented in the multiwfn package.^36^ Minimum energy crossing points were determined using easyMECP^37^ based on the original method of Harvey.^38^

## Supporting Information Available

Synthetic procedures and characterization data for all new compounds (PDF)
Optimized geometries (ZIP)

## References

[ref1] aColobert F.; Wencel-Delord J. C–H Activation for Asymmetric Synthesis; Wiley-VCH, 2019.

[ref2] aLiu C.-X.; Yin S.-Y.; Zhao F.; Yang H.; Feng Z.; Gu Q.; You S.-L. Rhodium Catalyzed Asymmetric C–H Functionalization Reactions. Chem. Rev. 2023, 123, 10079–10134. 10.1021/acs.chemrev.3c00149. 37527349

[ref3] aWoźniak Ł.; Cramer N. Enantioselective C–H Bond Functionalizations by 3d Transition-Metal Catalysts. Trends Chem. 2019, 1, 471–484. 10.1016/j.trechm.2019.03.013.

[ref4] Ozols K.; Jang Y.-S.; Cramer N. Chiral Cyclopentadienyl Cobalt(III) Complexes Enable Highly Enantioselective 3d-Metal-Catalyzed C-H Functionalizations. J. Am. Chem. Soc. 2019, 141, 5675–5680. 10.1021/jacs.9b02569. 30901216

[ref5] aOzols K.; Onodera S.; Woźniak Ł.; Cramer N. Cobalt(III)-Catalyzed Enantioselective Intermolecular Carboamination by C–H Functionalization. Angew. Chem., Int. Ed. 2021, 60, 655–659. 10.1002/anie.202011140. 32986927

[ref6] aZheng Y.; Zhang W. Y.; Gu Q.; Zheng C.; You S.-L. Cobalt(III)-Catalyzed Asymmetric Ring-Opening of 7-Oxabenzo-norbornadienes via Indole C-H Functionalization. Nat. Commun. 2023, 14, 1094 10.1038/s41467-023-36723-6. 36841798 PMC9968317

[ref7] aPesciaioli F.; Dhawa U.; Oliveira J. C. A.; Yin R.; John M.; Ackermann L. Enantioselective Cobalt(III)-Catalyzed C–H Activation Enabled by Chiral Carboxylic Acid Cooperation. Angew. Chem., Int. Ed. 2018, 57, 15425–15429. 10.1002/anie.201808595. 30289577

[ref8] avon Münchow T.; Dana S.; Xu Y.; Yuan B.; Ackermann L. Enantioselective Electrochemical Cobalt-Catalyzed Aryl C–H Activation Reactions. Science 2023, 379, 1036–1042. 10.1126/science.adg2866. 36893225

[ref9] aMas-Roselló J.; Herraiz A. G.; Audic B.; Laverny A.; Cramer N. Chiral Cyclopentadienyl Ligands: Design, Syntheses, and Applications in Asymmetric Catalysis. Angew. Chem., Int. Ed. 2021, 60, 13198–13224. 10.1002/anie.202008166. 32672405

[ref10] aLi P.; Zhang X.; Shi M. Recent Developments in Cyclopropene Chemistry. Chem. Commun. 2020, 56, 5457–5471. 10.1039/D0CC01612H. 32406444

[ref11] aVicente R. C–C Bond Cleavages of Cyclopropenes: Operating for Selective Ring-Opening Reactions. Chem. Rev. 2021, 121, 162–226. 10.1021/acs.chemrev.0c00151. 32639746

[ref12] aHuang J. Q.; Yu M.; Yong X.; Ho C. Y. NHC-Ni(II)-Catalyzed Cyclopropene-Isocyanide [5 + 1] Benzannulation. Nat. Commun. 2022, 13, 4145 10.1038/s41467-022-31896-y. 35842422 PMC9288548

[ref13] aZhang H.; Wang K.; Wang B.; Yi H.; Hu F.; Li C.; Zhang Y.; Wang J. Rhodium(III)-Catalyzed Transannulation of Cyclopropenes with *N*-Phenoxyacetamides through C–H Activation. Angew. Chem., Int. Ed. 2014, 53, 13234–13238. 10.1002/anie.201408555. 25267691

[ref14] aSemakul N.; Jackson K. E.; Paton R. S.; Rovis T. Heptamethyl indenyl (Ind*) Enables Diastereoselective Benzamidation of Cyclopropenes via Rh(III)-Catalyzed C–H Activation. Chem. Sci. 2017, 8, 1015–1020. 10.1039/C6SC02587K. 28451239 PMC5354047

[ref15] Shaaban S.; Li H.; Merten C.; Antonchick A. P.; Waldmann H. Rhodium (III)-Catalyzed Enantioselective Benzamidation of Cyclopropenes. Synthesis 2021, 53, 2192–2200. 10.1055/s-0040-1706026.

[ref16] Achar T. K.; Al-Thabaiti S. A.; Mokhtar M.; Maiti D. Enantioselective Annulation Reactions through C(sp^2^)–H Activation with Chiral Cp^x^M^III^ Catalysts. Chem. Catal. 2023, 3, 100575 10.1016/j.checat.2023.100575.

[ref17] aKim Y. L.; Park S.-a.; Choi S.-M.; Park J.-U.; Kim J. H. Co^III^-Catalyzed C–H Alkenylation and Allylation with Cyclopropenes via Sequential C–H/C–C Bond Activation. Org. Lett. 2021, 23, 6674–6679. 10.1021/acs.orglett.1c02219. 34474571

[ref18] aBelliotti T. R.; Brink W. A.; Kesten S. R.; Rubin J. R.; Wustrow D. J.; Zoski K. T.; Whetzel S. Z.; Corbin A. E.; Pugsley T. A.; Heffner T. G.; Wise L. D. Isoindolinone Enantiomers Having Affinity for the Dopamine D4 Receptor. Bioorg. Med. Chem. Lett. 1998, 8, 1499–1502. 10.1016/S0960-894X(98)00252-2. 9873377

[ref19] Gao W.; Chen M.-w.; Ding Q.; Peng Y. Catalytic Asymmetric Synthesis of Isoindolinones. Chem. - Asian J. 2019, 14, 1306–1322. 10.1002/asia.201900080. 30859747

[ref20] aYe B.; Cramer N. Asymmetric Synthesis of Isoindolones by Chiral Cyclopentadienyl Rhodium(III)- Catalyzed C-H Functionalizations. Angew. Chem., Int. Ed. 2014, 53, 7896–7899. 10.1002/anie.201404895. 24916401

[ref21] CCDC 2292368 (**3aa**) contains the supplementary crystallographic data for this paper. These data are provided free of charge by the Cambridge Crystallographic Data Centre.

[ref22] Wodrich M. D.; Chang M.; Gallarati S.; Wozniak L.; Cramer N.; Corminboeuf C. Mapping Catalyst–Solvent Interplay in Competing Carboamination/Cyclopropanation Reactions. Chem. - Eur. J. 2022, 28, e202200399 10.1002/chem.202200399. 35522013 PMC9401068

[ref23] aLaplaza R.; Sobez J.-G.; Wodrich M. D.; Reiher M.; Corminboeuf C. The (not so) simple prediction of enantioselectivity – A pipeline for high-fidelity computations. Chem. Sci. 2022, 13, 6858–6864. 10.1039/D2SC01714H. 35774159 PMC9200111

[ref24] Gao H.; Wang W.; Lv X.; Lu G.; Li Y. Mechanism of Co(III)-Catalyzed Annulation of *N*-Chlorobenzamide with Styrene and Origin of Cyclopentadienyl Ligand-Controlled Enantioselectivity. Org. Chem. Front. 2023, 10, 1643–1650. 10.1039/D3QO00038A.

[ref25] aZhang L.; Liu Y.; Deng L. Three-Coordinate Cobalt(IV) and Cobalt(V) Imido Complexes with N-Heterocyclic Carbene Ligation: Synthesis, Structure, and Their Distinct Reactivity in C–H Bond Amination. J. Am. Chem. Soc. 2014, 136, 15525–15528. 10.1021/ja509731z. 25330361

[ref26] aBecke A. D. Density-Functional Thermochemistry. III. The Role of Exact Exchange. J. Phys. Chem. A 1993, 98, 5648–5652. 10.1063/1.464913.

[ref27] aGrimme S.; Antony J.; Ehrlich S.; Krieg H. A Consistent and Accurate ab initio Parameterization of Density Functional Theory Dispersion Correction (DFT-D) for the 94 Elements H-Pu. J. Chem. Phys. 2010, 132, 154104 10.1063/1.3382344. 20423165

[ref28] Weigend F.; Ahlrichs R. Balanced Basis Sets of Split Valence, Triple Zeta Valence and Quadruple Zeta Valence Quality for H to Rn: Design and Assessment of Accuracy. Phys. Chem. Chem. Phys. 2005, 7, 3297–3305. 10.1039/b508541a. 16240044

[ref29] Frisch M. J.; Trucks G. W.; Schlegel H. B.; Scuseria G. E.; Robb M. A.; Cheeseman J. R.; Scalmani G.; Barone V.; Petersson G. A.; Nakatsuji H.; Li X.; Caricato M.; Marenich A. V.; Bloino J.; Janesko B. G.; Gomperts R.; Mennucci B.; Hratchian H. P.; Ortiz J. V.; Izmaylov A. F.; Sonnenberg J. L.; Williams-Young D.; Ding F.; Lipparini F.; Egidi F.; Goings J.; Peng B.; Petrone A.; Henderson T.; Rana-singhe D.; Zakrzewski V. G.; Gao J.; Rega N.; Zheng G.; Liang W.; Hada M.; Ehara M.; Toyota K.; Fukuda R.; Hasegawa J.; Ishida M.; Nakajima T.; Honda Y.; Kitao O.; Nakai H.; Vreven T.; Throssell K.; Montgomery J. A. Jr; Peralta J. E.; Ogliaro F.; Bearpark M. J.; Heyd J. J.; Brothers E. N.; Kudin K. N.; Staroverov V. N.; Keith T. A.; Kobayashi R.; Normand J.; Raghavachari K.; Rendell A. P.; Burant J. C.; Iyengar S. S.; Tomasi J.; Cossi M.; Millam J. M.; Klene M.; Adamo C.; Cammi R.; Ochterski J. W.; Martin R. L.; Morokuma K.; Farkas O.; Foresman J. B.; Fox D. J. Gaussian 16, Revision C.01; Gaussian, Inc.: Wallingford CT, 2016.

[ref30] Marenich A. V.; Cramer C. J.; Truhlar D. G. Universal Solvation Model Based on Solute Electron Density and on a Continuum Model of the Solvent Defined by the Bulk Dielectric Constant and Atomic Surface Tensions. J. Phys. Chem. B 2009, 113, 6378–6396. 10.1021/jp810292n. 19366259

[ref31] Grimme S. Supramolecular Binding Thermodynamics by Dispersion-Corrected Density Functional Theory. Chem. - Eur. J. 2012, 18, 9955–9964. 10.1002/chem.201200497. 22782805

[ref32] Gallarati S.; Dingwall P.; Fuentes J. A.; Bühl M.; Clarke M. L. Understanding Catalyst Structure–Selectivity Relationships in Pd-Catalyzed Enantioselective Methoxycarbonylation of Styrene. Organometallics 2020, 39, 4544–4556. 10.1021/acs.organomet.0c00613.

[ref33] Martin R. L.; Hay P. J.; Pratt L. R. Hydrolysis of Ferric Ion in Water and Conformational Equilibrium. J. Phys. Chem. A 1998, 102, 3565–3573. 10.1021/jp980229p.

[ref34] aLuchini G.; Alegre-Requena J. V.; Guan Y.; Funes-Ardoiz I.; Paton R. S. GoodVibes, v3.0.1, 2019. https://github.com/patonlab/.

[ref35] Thom A. J. W.; Sundstrom E. J.; Head-Gordon M. LOBA: A Localized Orbital Bonding Analysis to Calculate Oxidation States, with Application to a Model Water Oxidation Catalyst. Phys. Chem. Chem. Phys. 2009, 11, 11297–11304. 10.1039/b915364k. 20024398

[ref36] aLu T.; Chen F. Multiwfn: A Multifunctional Wavefunction Analyzer. J. Comput. Chem. 2012, 33, 580–592. 10.1002/jcc.22885. 22162017

[ref37] Rodriguez-Guerra J.; Funes-Ardoiz I.; Maseras F. EasyMECP Zenodo 2020 10.5281/zenodo.4293421.

[ref38] Harvey J. N.; Aschi M.; Schwarz H.; Koch W. The singlet and triplet states of phenyl cation. A hybrid approach for locating minimum energy crossing points between non-interacting potential energy surfaces. Theor. Chem. Acc. 1998, 99, 95–99. 10.1007/s002140050309.

