# Supplementary material for: Enantioselective Cobalt(III)-Catalyzed [4 + 1] Annulation of Benzamides: Cyclopropenes as One-Carbon Synthons
Source: J Am Chem Soc. 2025 Apr 28;147(18):15041–9. doi: 10.1021/jacs.4c16953 (PMC12063185; doi:10.1021/jacs.4c16953)
Supplement: Supplementary file 1 — ja4c16953_si_001.pdf [file ja4c16953_si_001.pdf]

Supporting Information

Enantioselective Cobalt(III)-Catalyzed [4+1]-Annulation of Benzamides: Cyclopropenes as One-Carbon Synthons

Lenin Kumar Verdhi,[1] Matthew D. Wodrich,[2] Nicolai Cramer[1]*

[1]Laboratory of Asymmetric Catalysis and Synthesis, Institute of Chemical Sciences and Engineering, Ecole Polytechnique Fédérale de Lausanne (EPFL), 1015 Lausanne, Switzerland.

[2]Laboratory for Computational Molecular Design, Institute of Chemical Sciences and Engineering, Ecole Polytechnique Fédérale de Lausanne (EPFL), 1015 Lausanne, Switzerland.

*Email: nicolai.cramer@epfl.ch

Table of Contents

General methods S2
Synthesis of cobalt and rhodium complexes S3
Synthesis of *N*-chlorobenzamides S3
Synthesis of cyclopropenes S5
General procedure for reaction optimization S7
General procedure for cobalt catalyzed [4+1] annulation S11
Substrate scope S12
Cp^x vs Cp* ligand effect on annulation process S45
Procedure for Rh-catalyzed [4+2] annulation S47
Kinetic Isotope Effect studies S49
X-ray Structure of (*R*,*E*)-**3aa** S52
Computational details S56
References S60
NMR Spectra S61

General methods:

Unless otherwise indicated, all reactions were carried out under nitrogen atmosphere by using standard Schlenk or glovebox techniques in an oven-dried glassware with magnetic stirring. Reagents were purchased and used as obtained from the suppliers. Solvents were obtained using a solvent purification system with an aluminum oxide column (Innovative Technologies). Dichloromethane (DCM), diethyl ether, 2,2,2-Trifluoroethanol (TFE), and 1,2-dichloroethane (DCE) were degassed via freeze-pump-thaw technique. Sodium carbonate was grinded to fine powder in a glovebox and stored in a dry box. Flash chromatography was performed with Silicycle silica gel 60 (0.040- 0.063 μm grade). Analytical thin layer chromatography was performed with commercial glass plates coated with 0.25 mm silica gel (E. Merck, Kieselgel 60 F254). Compounds were either visualized under UV-light at 254 nm or by dipping the plates in an aqueous potassium permanganate solution followed by heating. Melting points of solids were measured using BÜCHI Melting Point B-450 apparatus.

NMR spectra: Proton nuclear magnetic resonance (^{1}H NMR) data was acquired on a Bruker AVANCE400 (400 MHz), Bruker DRX-400 (400 MHz), Bruker AVANCEIII-400 (400 MHz) or Bruker DRX-600 (600 MHz) spectrometer at 298 K unless otherwise noted. Chemical shifts (δ) for ^{1}H NMR are reported in ppm relative to incompletely deuterated NMR solvent [$CDCl_3$ δ = 7.26 ppm, CD_3OD = δ 3.31 ppm, $(CD_3)_2CO$ = δ 2.05 ppm, $(CD_3)_2SO$ = δ 2.50 ppm]. Splitting patterns are designated as s (singlet), d (doublet), t (triplet), q (quartet), m (multiplet), br (broad singlet). Proton-decoupled ^{13}C nuclear magnetic resonance ($^{13}C\{1H\}$ NMR) data were acquired on a Bruker AVANCE400 (101 MHz), or DRX600 (151 MHz) spectrometer at 298 K unless noted otherwise. Chemical shifts are reported in ppm relative to residual solvent peaks in $CDCl_3$ (77.16 ppm), CD_3OD (49.00 ppm), $(CD_3)_2CO$ (29.84 and 206.26 ppm), $(CD_3)_2SO$ (39.52 ppm). Proton-decoupled ^{19}F nuclear magnetic resonance ($^{19}F\{1H\}$ NMR) data were acquired at 376 MHz on a Bruker AVANCEIII-400 spectrometer at 298 K.

IR Spectra: Infrared (IR) data were recorded on an Alpha-P Bruker FT-IR Spectrometer. Absorptions are given in wavenumbers (cm^{-1}).

Mass spectra: High resolution mass spectra (HRMS) measurements were obtained by an Agilent LC-MS TOF and Xevo G2-S QTOF mass spectrometers. High resolution mass values are given in *m/z*.

HPLC: The enantiomeric ratio of compounds was measured on an Agilent HPLC system using CHIRALPAK columns with hexane and isopropanol (IPA) as solvents.

Optical rotations: Optical rotation of the compounds was measured on a Polartronic M polarimeter using a 10.0 cm cell with a Na 589 nm filter.

X-ray crystallography: X-ray analysis was performed by Dr. R. Scopelliti at the EPF Lausanne.

Synthesis of catalysts and substrates

Chiral cyclopentadienyl cobalt and rhodium catalysts

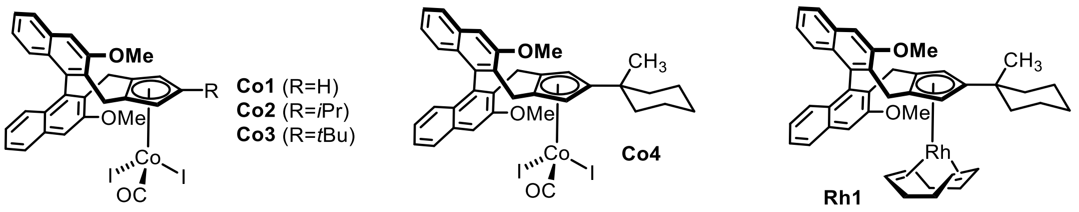

Co1 to **Co4** complexes were synthesized following a reported procedure by Cramer *et al.*[1,2]

Rh1 complex was synthesized following a reported procedure by Cramer *et al.*[3]

N-Chlorobenzamides

General procedure for the synthesis of *N*-chlorobenzamides (GP1)

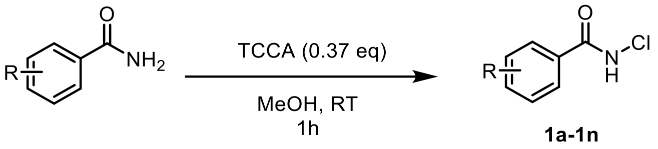

According to previously reported procedure,[4] to a stirred solution of benzamide (10.0 mmol, 1.0 eq.) in methanol (20 mL), trichloroisocyanuric acid (0.37 eq) was added at room temperature. After being stirred at 23 ˚C for 1-2 h, the reaction mixture was filtered and washed with dichloromethane (2x10 mL). The filtrate was concentrated in vacuo. The obtained crude product was subjected to silica gel column chromatography using ethyl acetate/pentane as an eluent to obtain the desired product.

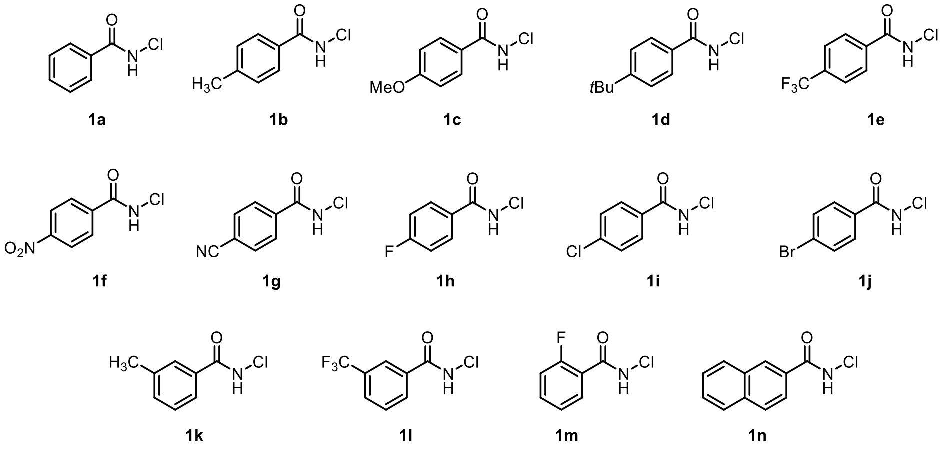

***N*-Chlorobenzamides used in this work**

N-Chlorobenzamides (**1a-1c, 1f-1k, 1m**) are known compounds[4]

4-(tert-butyl)-*N*-chlorobenzamide (1d)

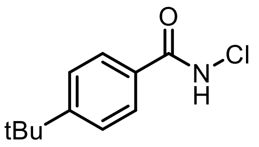

The reaction was carried out on a 5.0 mmol scale.

53% yield (550 mg), White colour solid.

m.p. = 98-100 ̊C. **R$_f$** = 0.42 (pentane/ethylacetate = 4/1). **^{1}H NMR** (400 MHz, Acetone-d$_6$) δ 9.37 (s, 1H), 7.82 (d, *J* = 8.6 Hz, 2H), 7.54 (d, *J* = 8.6 Hz, 2H), 1.33 (s, 9H). **^{13}C NMR** (101 MHz, Acetone-d$_6$) δ 166.7, 156.4, 130.5, 128.9, 126.4, 35.5, 31.3. **HRMS** (APCI/QTOF): *m/z* calcd. for $C_{11}H_{15}ClNO^+$ $[M + H]^+$: 212.0837; found: 212.0835. **IR** (ATR): 3059, 2871, 1644, 1609, 1453, 1299, 1277, 852, 709, 655, 627 cm^{-1}.

***N*-chloro-4-(trifluoromethyl)benzamide (1e)**

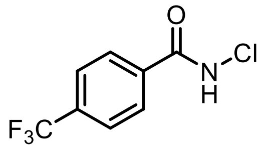

The reaction was carried out on a 5.0 mmol scale.

72% yield (807 mg), White colour solid.

m.p. = 195-198 ̊C. **R$_f$** = 0.30 (pentane/ethylacetate = 4/1).**^{1}H NMR** (400 MHz, Acetone-d$_6$) δ 8.08 (d, *J* = 8.1 Hz, 2H), 7.87 (d, *J* = 8.2 Hz, 2H). **^{13}C NMR** (101 MHz, Acetone-d$_6$) δ 165.7, 137.0, 133.8 (q, J_{F-C} = 32.3 Hz), 129.4, 126.5 ((q, J_{F-C} = 4.0 Hz), 123.3 (q, J_{F-C} = 272.7 Hz) **^{19}F NMR** (376 MHz, Acetone-d$_6$) δ -63.6. **HRMS** (APCI/QTOF): *m/z* calcd. for $C_8H_6ClF_3NO^+$ $[M + H]^+$: 224.0085; found: 224.0086. **IR** (ATR): 3089, 1656, 1461, 1323, 1277, 1161, 1125, 1115, 1065, 858, 768, 666 cm^{-1}.

***N*-chloro-3-(trifluoromethyl)benzamide (1l)**

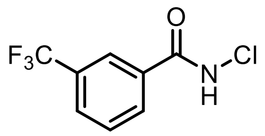

The reaction was carried out on a 7.5 mmol scale.

88% yield (1478 mg), white colour solid.

m.p. = 136-138 ̊C. **R$_f$** = 0.36 (pentane/ethylacetate = 4/1). **^{1}H NMR** (400 MHz, Acetone-d$_6$) δ 8.20 – 8.14 (m, 2H), 7.95 (d, *J* = 7.8 Hz, 1H), 7.78 (t, *J* = 7.7 Hz, 1H). **^{13}C NMR** (101 MHz, Acetone-d$_6$) δ 165.5, 134.3, 132.4, 131.3 (q, J_{F-C} = 31.9 Hz), 130.80, 129.5 (q, J_{F-C} = 3.7 Hz), 125.3 (q, J_{F-C} = 4.0 Hz), 124.9 (q, J_{F-C} = 272.7 Hz). **^{19}F NMR** (376 MHz, Acetone-d6) δ -63.4. **HRMS** (APCI/QTOF): *m/z* calcd. for $C_8H_6ClF_3NO^+$ $[M + H]^+$: 224.0085; found: 224.0088. **IR** (ATR): 3210, 1649, 1469, 1422, 1328, 1263, 1185, 1113, 1073, 691, 677 cm^{-1}.

***N*-chloro-2-naphthamide (1n)**

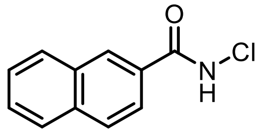

The reaction was carried out on a 5.0 mmol scale.

53% yield (550 mg), white colour solid.

m.p. = 146-149 ℃. **R_f** = 0.31 (pentane/ethylacetate = 4/1). **^{1}H NMR** (400 MHz, Acetone-d_6) δ 8.47 (s, 1H), 8.06 – 7.96 (m, 3H), 7.93 (dd, *J* = 8.6, 1.8 Hz, 1H), 7.68 – 7.58 (m, 2H). **^{13}C NMR** (101 MHz, Acetone-d_6) δ 167.0, 135.8, 133.4, 130.6, 129.8, 129.3, 129.2, 128.9, 128.6, 127.8, 124.9. **HRMS** (APCI/QTOF): *m/z* calcd. for $C_{11}H_9ClNO^+$ [M + H]$^+$: 206.0367; found: 206.0371. **IR** (ATR): 3056, 2852, 1650, 1470, 1445, 1428, 1284, 773, 759, 476 cm^{-1}.

Cyclopropenes

General procedure for the synthesis of cyclopropenes (GP2)

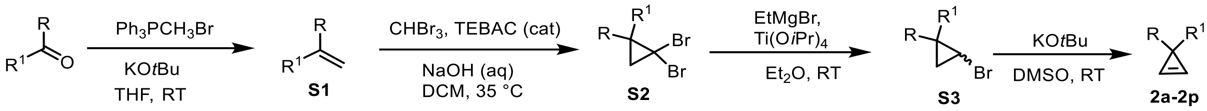

Synthesis of **S1**:

Under nitrogen atmosphere, potassium *tert*-butoxide (12 mmol, 1.2 eq.) was added to a stirred mixture of methyltriphenylphosphonium bromide (12 mmol, 1.2 eq) in anhydrous THF. The resulting yellow colour reaction mixture was allowed to stir at room temperature for 1 h. A solution of the ketone (10 mmol, 1.0 eq.) in anhydrous THF was added dropwise over a period of 10 min. The resulting reaction mixture was stirred at room temperature for 1.5-2 hours. The crude mixture was then passed through a pad of silica and washed with hexane. The filtrate was concentrated, and the obtained crude product was subjected to silica gel flash column chromatography using pentane as an eluent to get the alkene **S1**.

Synthesis of **S2**:

To a solution of alkene **S1** (1.0 eq.), bromoform (1.6 eq.), benzyltriethylammonium chloride (TEBAC) (1 mol%) in dichloromethane was added a dropwise aqueous solution of 50% NaOH. After completion of addition, the reaction mixture was warmed to 35 °C for 48 h. The reaction mixture was cooled to room temperature and diluted with water and DCM. The aqueous phase was extracted with DCM. The combined organic phases were washed with saturated NaCl solution and separated. The organic layer was dried over Na_2SO_4 and the solvent was removed

under reduced pressure. The reaction mixture was purified by flash column chromatography using pentane as an eluent to get **S2**.

<u>Synthesis of **S3**:</u>

Under nitrogen atmosphere, Ti(*i*PrO)$_4$ (10 mol%) was added to a solution of dibromocyclopropane **S2** (1.0 eq.) in anhydrous Et$_2$O. The resulting mixture was cooled in an ice bath and added a 3.0 M solution of EtMgBr in Et$_2$O (1.2 eq.) dropwise. The obtained brown-black colour mixture was stirred at room temperature for 4 h. After which, the reaction mixture was cooled in an ice bath and quenched with 10% H$_2$SO$_4$ solution. After stirring for 10 min, the organic phase was separated, and the aqueous phase was extracted with Et$_2$O. The combined organic phases were washed with saturated NaHCO$_3$ followed by saturated NaCl solution. The organic layer was separated dried over Na$_2$SO$_4$ and filtered. The filtrate was concentrated in vacuo and the crude product was purified by flash column chromatography using pentane as eluent to afford **S3** as mixture of diastereomers.

<u>Synthesis of cyclopropenes **(2)**</u>

Under nitrogen atmosphere, to a solution of monobromocyclopropane **S3** (1.0 eq.) in anhydrous DMSO, KO*t*Bu (1.3 eq.) was added. The resulting dark colour reaction mixture was stirred at room temperature for overnight (12-16 h). After which, water was added to the reaction mixture and extracted Et$_2$O. The combined organic phases were washed with saturated NaCl solution. The organic layer was separated, dried over Na$_2$SO$_4$ and filtered. The solvent was removed under vacuo and the crude product was purified by flash column chromatography using pentane as eluent to afford the desired cyclopropene **2**.

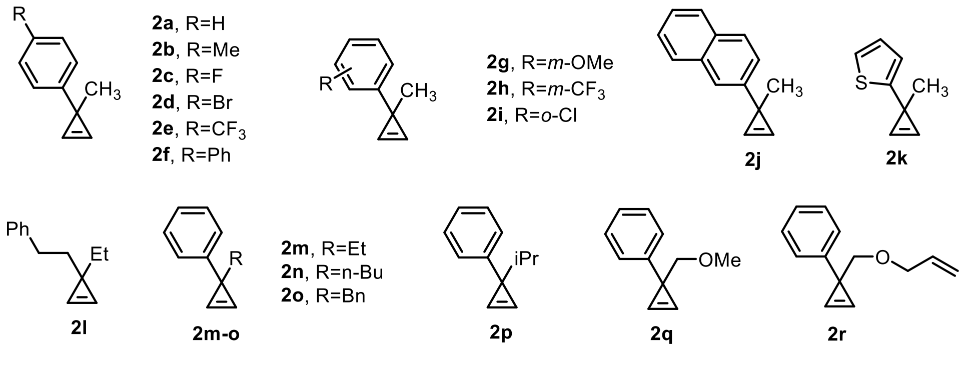

Cyclopropenes used in this work

Cyclopropenes (**2a-2k**, **2m-2n, 2p**)[5-7], **2l**[8] and **2o**[9] were synthesized according to GP2. Cyclopropene **2q** was synthesized according to previously reported procedure.[5]

Synthesis of cyclopropene (2r)

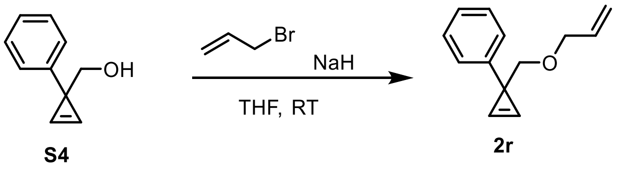

Under nitrogen atmosphere, a solution of the alcohol **S4**[10] (500 mg, 3.42 mmol) in anhydrous THF was cooled to 0 ˚C and added NaH (1.2 eq). The mixture was stirred for 10 minutes at 0 ˚C. Allyl bromide (2 eq) was added dropwise and the resulting mixture stirred at room temperature for overnight. The mixture was quenched with saturated NH_4Cl solution and extracted with Et_2O. The combined organic phases were washed with water and brine solution, then dried over Na_2SO_4 and filtered. The filtrate was concentrated, and the obtained residue was subjected to silica gel flash column chromatography to obtain the product **2r**.

(1-((allyloxy)methyl)cycloprop-2-en-1-yl)benzene (2r)

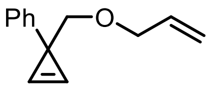

55% yield (350 mg), colourless liquid. **R$_f$** = 0.69 (pentane/ethylacetate = 20/1). **^{1}H NMR** (400 MHz, $CDCl_3$) δ 7.31 – 7.24 (m, 6H), 7.19 – 7.15 (m, 1H), 5.99-5.89 (m, 1H), 5.27 (dq, *J* = 17.2, 1.7 Hz, 1H), 5.18 (dq, *J* = 10.4, 1.5 Hz, 1H), 4.04 (dt, *J* = 5.7, 1.4 Hz, 2H), 3.90 (s, 2H). **^{13}C NMR** (101 MHz, $CDCl_3$) δ 146.7, 135.1, 128.1, 126.5, 125.6, 117.0, 112.8, 77.4, 71.7, 26.8. **HRMS** (ESI/QTOF): *m/z* calcd. for $C_{13}H_{14}NaO^+$ $[M + Na]^+$: 209.0937; found: 209.0932. **IR** (ATR): 3082, 3058, 3023, 2851, 1726, 1645, 1601, 1493, 1092, 992, 925, 699, 631 cm^{-1}.

Enantioselective Co^{III}-catalyzed [4+1] annulation of benzamides with cyclopropenes

General procedure for optimization studies:

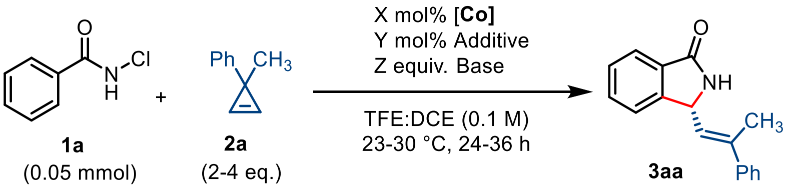

To an oven dried 5 mL microwave tube, *N*-chlorobenzamide (0.05 mmol, 1 eq.), cobalt catalyst (X mol%), silver salt (Y mol%), and base (Z mol%) were added. The reaction vessel was connected to Schlenk line and evacuated with vacuum and then refilled with nitrogen (3 cycles). Under nitrogen atmosphere, degassed trifluoroethanol (0.35 mL) was added and stirred for 5 -

10 min. Cyclopropene (2-4 eq.) was dissolved in degassed dichloroethane (0.15 mL) and added to the above reaction mixture. The resulting reaction mixture was stirred at specified temperature for specified time. The rection mixture was quenched by addition of acetic acid (0.1 mL) and ethylacetate (0.5 mL), stirred for 10 min at 23 °C, and subsequently filtered through a pad of silica gel. The resulting crude reaction mixture was analyzed by quantitative NMR with 1,3,5-trimethoxy benzene as the internal standard. For er determination, the crude mixture was purified by silica gel column chromatography (eluent pentane/ethylacetate 20% → 30%) and analyzed by HPLC using a chiral stationary phase (Chiralpak IB, 4.6 x 250 mm; 15% i-PrOH/hexane, 1.0 mL/min, 254 nm).

Table S1. Screening of Cobalt catalysts

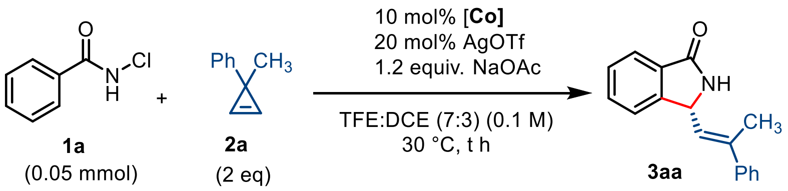

entry	**Co**-catalyst	time	Yield(%)	er
1	Cp*Co(CO)I_2	36	36	---
2	Co1	36	---	---
3	Co2	18	43	81.5:18.5
**4**	**Co3**	**36**	**34**	**96:4**
5	Co4	36	23	95:5

Table S2. Screening of silver salts

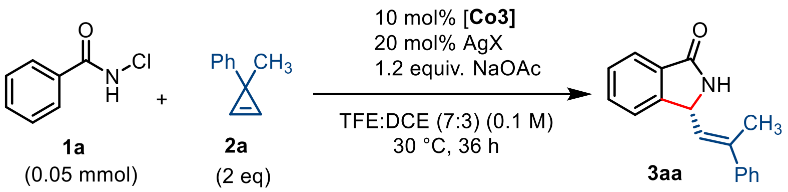

entry	Ag salt	Yield (%)	er
1	AgOTf	34	96:4
2	$AgSbF_6$	28	95:5
3	$AgPF_6$	33	95:5
4	AgOBz	33	94:6

Table S3. Screening of additives and bases

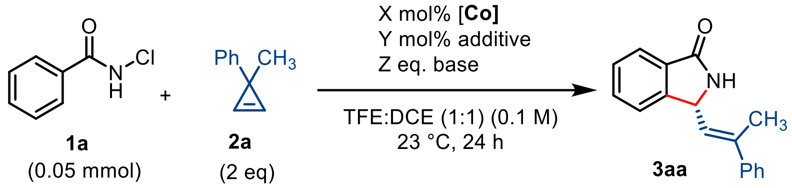

entry	[Co] (X mol%)	Additive (Y mol%)	Base (Z eq)	Yield (%)	er
1	**Co3** (5)	AgOBz (10)	Na_2CO_3 (0.5 eq)	46	95:5
2	**Co4** (5)	AgOBz (10)	Na_2CO_3 (0.5 eq)	52	95:5
3	**Co3** (10)	AgOBz (20)	Na_2CO_3 (0.5 eq)	55	94:6
4[a]	**Co4** (5)	AgOBz (10)	Na_2CO_3 (0.5 eq)	64	96.5:3.5
5[a,b]	**Co4** (5)	Benzoic acid (10)	Na_2CO_3 (0.5 eq)	61	97:3
6[a,b]	**Co4** (5)	*p*-CF_3-benzoic acid (10)	Na_2CO_3 (0.5 eq)	61	96.5:3.5
7[a,b]	**Co4** (5)	*p*-MeO-benzoic acid (10)	Na_2CO_3 (0.5 eq)	48	97:3
8[a,b]	**Co4** (5)	Pentafluorobenzoic acid (10)	Na_2CO_3 (0.5 eq)	31	97:3
9[a,b]	**Co4** (5)	Adamantane carboxylic acid (10)	Na_2CO_3 (0.5 eq)	39	97.5:2.5
10[a,b]	**Co4** (5)	Benzoic acid (10) + proton sponge	Na_2CO_3 (0.5 eq)	54	97:3
11[a,b]	**Co4** (5)	Benzoic acid (10) + 4Å molecular sieves (20 mg)	Na_2CO_3 (0.5 eq)	60	96.5:3.5
12[a]	**Co4** (5)	Benzoic acid (10)	K_2CO_3 (0.5 eq)	25	---
13[a]	**Co4** (5)	Benzoic acid (10)	Cs_2CO_3 (0.5 eq)	20	---
14[a]	**Co4** (5)	Benzoic acid (10)	Ag_2CO_3 (0.5 eq)	---	---
15[a]	**Co4** (10)	AgOBz (10)	Na_2CO_3 (0.5 eq)	71	95:5
16[a]	**Co3** (10)	AgOBz (10)	Na_2CO_3 (0.5 eq)	65	95:5
**17[a,b]**	**Co4** (10)	**Benzoic acid (10)**	**Na_2CO_3 (0.6eq)**	**74**	**97:3**

[a] 4eq of cyclorpropene **2a,** [b]36 hours.

Table S4. Screening of solvents

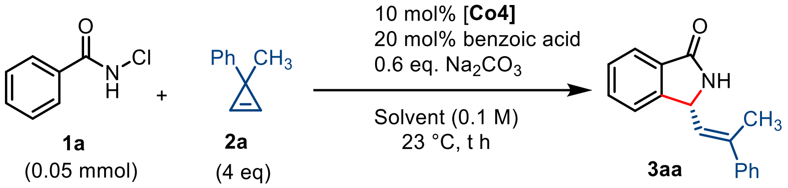

entry	Solvent	time	Yield (%)	er
**1**	**TFE:DCE (1:1)**	**36**	**74**	**97:3**
2	TFE:Tetrachloroethane	36	46	97:3
3	TFE:DCM (1:1)	36	63	95.5:4.5
4	TFE:THF	24	---	---
5	TFE:2-Me-THF	24	---	---
6	TFE:Acetonitrile	24	---	---

Table S5. Effect of concentration

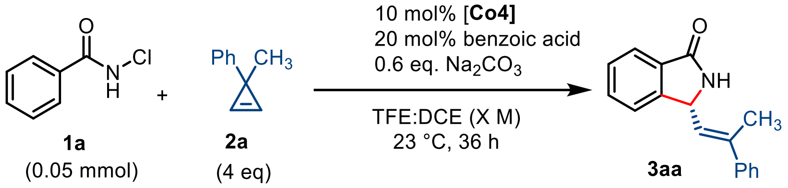

entry	Concentration	time	Yield (%)	er
**1**	**0.1 M**	**36**	**74**	**97:3**
2	0.5	36	44	97:3
3	0.05	36	68	94.5:5.5

Table S6. Screening of Directing Group's

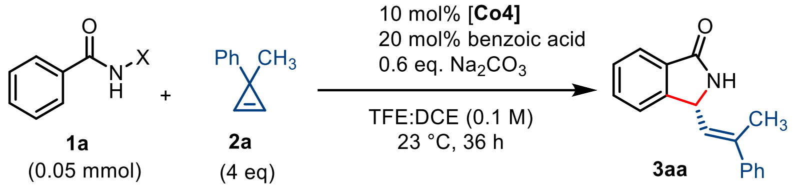

entry	X	Yield (%)	er
**1**	**Cl**	**74**	**97:3**
2	OMe	0	---
3	OPiv	0	---
4	OBoc	10	55:45

General procedure for Enantioselective Co(III)-catalyzed [4+1] annulation of benzamides with cyclopropenes (for substrate scope)

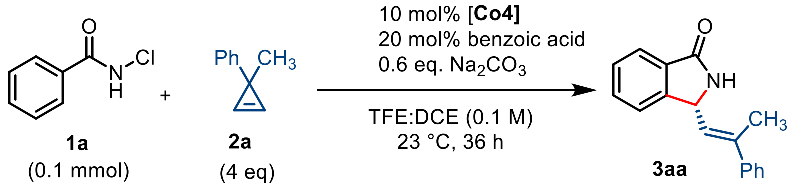

To an oven dried 5 mL microwave tube, *N*-chlorobenzamide (0.1 mmol, 1 eq.), cobalt catalyst (10 mol%), benzoic acid (20 mol%) and Na_2CO_3 (0.6 eq.) were added. The reaction vessel was connected to Schlenk line and evacuated with vacuum and then refilled with nitrogen (3 cycles). Under nitrogen atmosphere, degassed trifluoroethanol (0.5 mL) was added and stirred for 5 - 10 min. Cyclopropene (4 eq.) was dissolved in degassed dichloroethane (0.5 mL) and added to the above reaction mixture. The resulting reaction mixture was stirred at room temperature for 36 hours. The rection mixture was quenched by addition of acetic acid (0.2 mL) and ethylacetate (1.0 mL), stirred for 10 min at 23 °C, and subsequently filtered through a pad of silica gel and washed with ethylacetate. The resulting crude reaction mixture was concentred in vacuo and obtained residue was subjected to flash chromatography on silica gel (eluent: 30-40% ethyl acetate in pentane) to obtain the chiral isoindolinone **3**.

Racemic trace: Racemic samples were prepared by using 5-10 mol% of achiral $Cp^*Co(CO)I_2$ complex as a catalyst.

(*R*,*E*)-3-(2-phenylprop-1-en-1-yl)isoindolin-1-one (3aa)

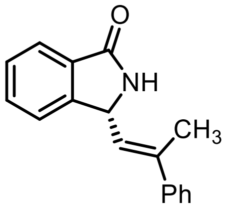

71% yield (17.8 mg, 0.071 mmol, *E/Z* > 20:1), white solid. **m.p.** = 162-165 ℃. **R$_f$** = 0.18 (pentane/ethylacetate = 1/1). **^{1}H NMR** (400 MHz, Methanol-d$_4$): δ 7.79 (d, *J* = 7.6 Hz, 1H), 7.62 (t, *J* = 7.2 Hz, 1H), 7.50 (dd, *J* = 17.3, 7.5 Hz, 2H), 7.44 – 7.37 (m, 2H), 7.30 (t, *J* = 7.4 Hz, 2H), 7.27 – 7.20 (m, 1H), 5.65 (d, *J* = 9.3 Hz, 1H), 5.43 (dq, *J* = 9.3, 1.5 Hz, 1H), 2.38 (d, *J* = 1.3 Hz, 3H). **^{13}C NMR** (101 MHz, Methanol-d$_4$): δ 173.0, 149.1, 143.9, 141.6, 133.5, 132.9, 129.5, 129.4, 128.6, 126.9, 125.4, 124.5, 124.3, 56.9, 16.8. **HRMS** (ESI/QTOF): *m/z* calcd. for $C_{17}H_{16}NO^+$ $[M + H]^+$: 250.1226; found: 250.1228. **IR** (ATR): 3214, 3058, 1689, 1468, 748, 693 cm^{-1}. **[α]$_D$ ^{20}** = +15.3 (c = 0.6, $CHCl_3$). **Chiral HPLC:** Chiralpak IB, 4.6 x 250 mm; 15% i-PrOH/hexane, 1.0 mL/min, 254 nm; t$_R$ (minor) = 7.38 min, t$_R$ (major) = 16.70 min, 97:3 *er*.

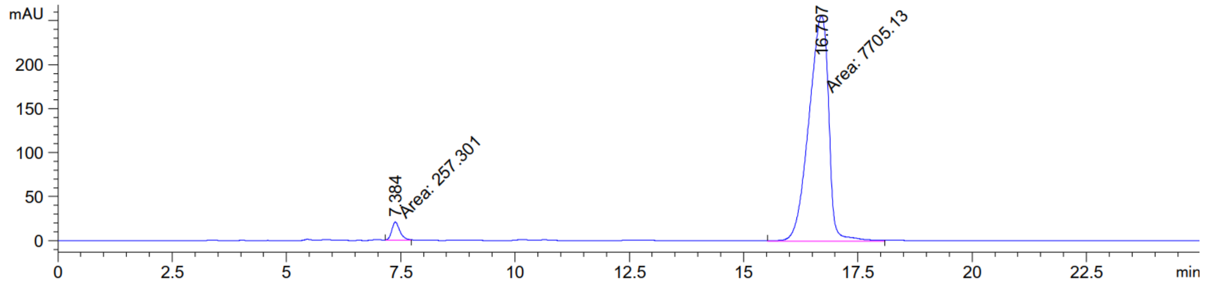

Peak #	RetTime [min]	Type	Width [min]	Area [mAU*s]	Height [mAU]	Area %
1	7.384	MM	0.2079	257.30148	20.62520	3.2314
2	16.707	MM	0.5014	7705.13232	256.13919	96.7686

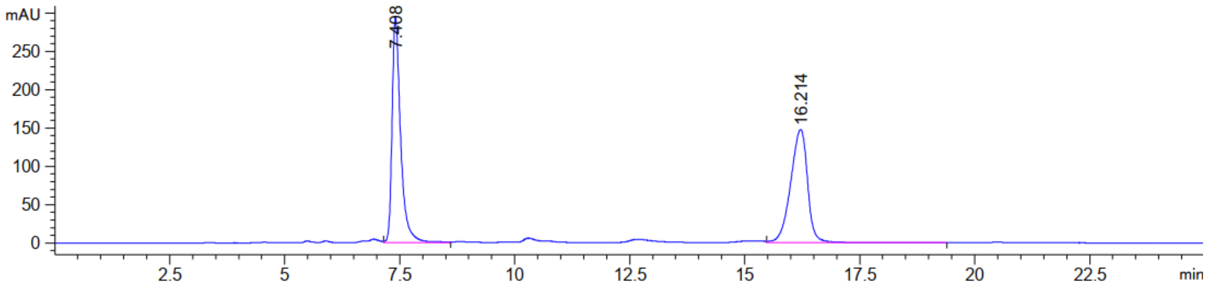

Peak #	RetTime [min]	Type	Width [min]	Area [mAU*s]	Height [mAU]	Area %
1	7.408	VV	0.1925	3798.56299	294.48297	49.9975
2	16.214	VB	0.4003	3798.94897	147.60013	50.0025

(*R*,*E*)-5-methyl-3-(2-phenylprop-1-en-1-yl)isoindolin-1-one (3ba)

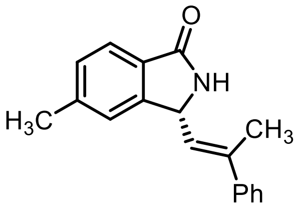

65% yield (17.2 mg, 0.065 mmol, *E/Z* > 20:1), pale yellow colour solid. **m.p.** = 219-221 ˚C. **R_f** = 0.17 (pentane/ethylacetate =1/1). **^{1}H NMR** (400 MHz, Methanol-d_4): δ 7.67 (d, *J* = 7.8 Hz, 1H), 7.44 – 7.39 (m, 2H), 7.36 – 7.21 (m, 5H), 5.60 (d, *J* = 9.3 Hz, 1H), 5.44 (dq, *J* = 9.3, 1.4 Hz, 1H), 2.45 (s, 3H), 2.38 (d, *J* = 1.4 Hz, 3H). **^{13}C NMR** (101 MHz, Methanol-d_4): δ 173.2, 149.6, 144.6, 143.9, 141.5, 130.5, 130.3, 129.4, 128.6, 126.9, 125.6, 124.8, 124.1, 56.8, 21.9, 16.8. **HRMS** (ESI/QTOF): *m/z* calcd. for $C_{18}H_{18}NO^+$ [M + H]$^+$: 264.1383, found 264.1387. **IR** (ATR): 3207, 2923, 2854, 1692, 1619, 1445, 759, 698 cm^{-1}. **[α]$_D$ ^{20}** = +54.3 (c = 1.0, $CHCl_3$). **Chiral HPLC:** Chiralpak IB, 4.6 x 250 mm; 15% i-PrOH/hexane, 1.0 mL/min, 254 nm; t_R (minor) = 7.18 min, t_R (major) = 14.60 min, 99:1 *er*

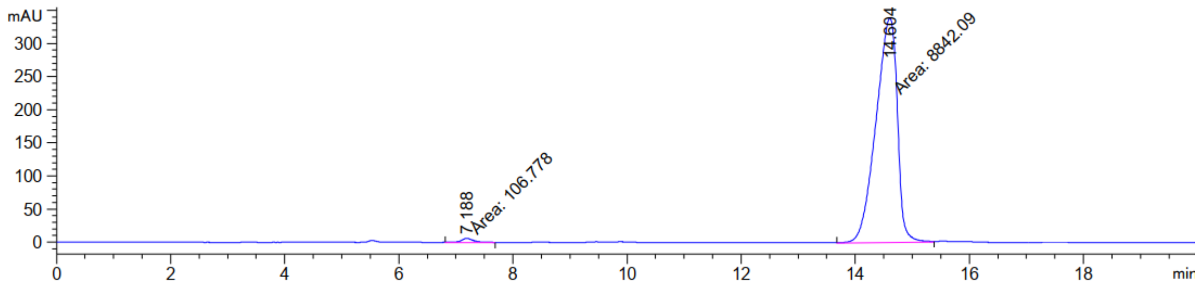

Peak #	RetTime [min]	Type	Width [min]	Area [mAU*s]	Height [mAU]	Area %
1	7.188	MM	0.2721	106.77804	6.54078	1.1932
2	14.604	MM	0.4348	8842.09375	338.96167	98.8068

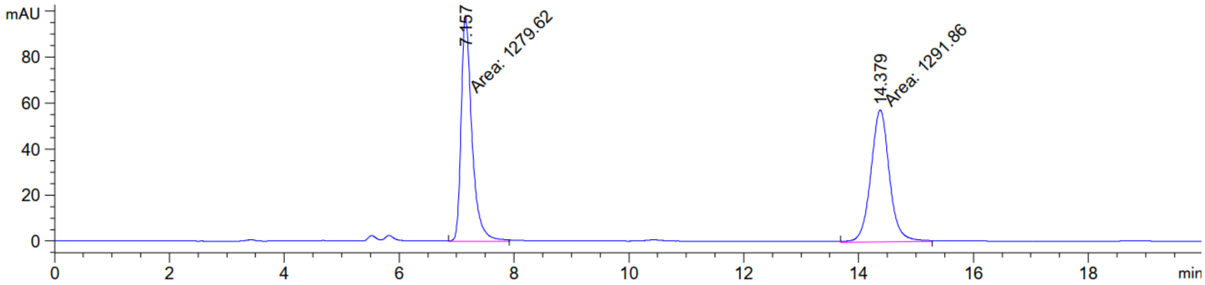

Peak #	RetTime [min]	Type	Width [min]	Area [mAU*s]	Height [mAU]	Area %
1	7.157	MM	0.2173	1279.61658	98.13945	49.7619
2	14.379	MM	0.3753	1291.86365	57.36500	50.2381

(*R*,*E*)-5-methoxy-3-(2-phenylprop-1-en-1-yl)isoindolin-1-one (3ca)

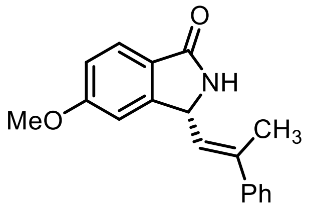

57% yield (15.8 mg, 0.057 mmol, *E/Z* > 20:1), pale brown colour solid. **m.p. =** 174-176 ̊C. **R_f** = 0.37 (pentane/ethylacetate = 1/1). **^{1}H NMR** (400 MHz, $CDCl_3$): δ 7.71 (d, *J* = 8.5 Hz, 1H), 7.37 – 7.30 (m, 2H), 7.29 – 7.18 (m, 3H), 6.93 (dd, *J* = 8.4, 2.2 Hz, 1H), 6.77 (d, *J* = 2.2 Hz, 1H), 6.62 (s, 1H), 5.51 (dd, *J* = 9.3, 1.6 Hz, 1H), 5.44 (d, *J* = 9.3 Hz, 1H), 3.79 (s, 3H), 2.29 (s, 3H). **^{13}C NMR** (101 MHz, $CDCl_3$): δ 170.8, 163.3, 149.6, 142.1, 139.6, 128.4, 127.8, 125.8, 125.2, 124.9, 124.3, 114.9, 107.9, 55.7, 55.1, 16.6. **HRMS** (ESI/QTOF): *m/z* calcd. for $C_{18}H_{18}NO_2^+$ $[M + H]^+$: 280.1332, found 280.1336. **IR** (ATR): 3225, 3060, 2923, 1687, 1610, 1492, 1280, 1250, 1025, 760, 695 cm^{-1}. **$[\alpha]_D^{20}$** = +114.3 (c = 1.0, $CHCl_3$). **Chiral HPLC:** Chiralpak IB, 4.6 x 250 mm; 15% i-PrOH/hexane, 1.0 mL/min, 254 nm; t_R (minor) = 9.62 min, t_R (major) = 19.03 min, 98:2 *er*

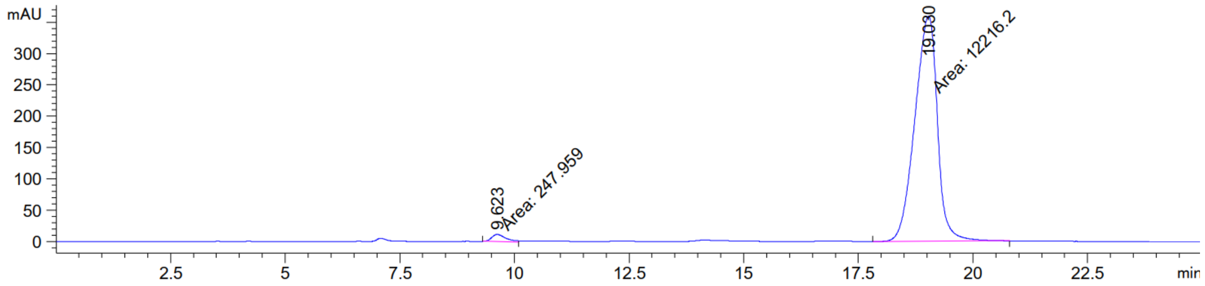

Peak #	RetTime [min]	Type	Width [min]	Area [mAU*s]	Height [mAU]	Area %
1	9.623	MM	0.3589	247.95888	11.51587	1.9894
2	19.030	MM	0.5675	1.22162e4	358.79990	98.0106

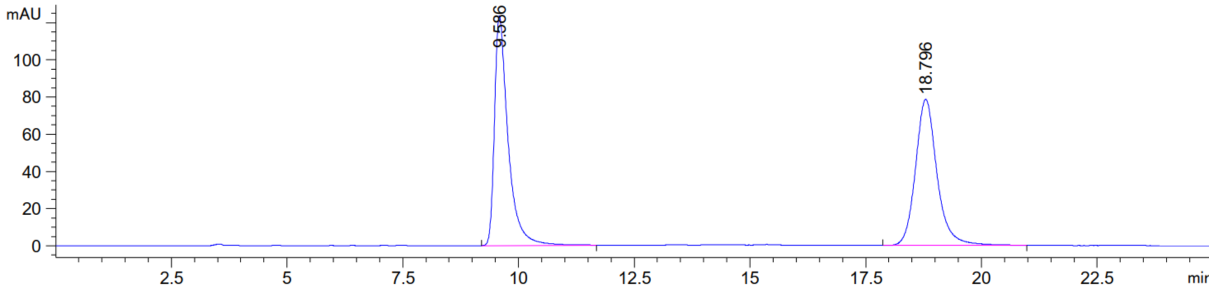

Peak #	RetTime [min]	Type	Width [min]	Area [mAU*s]	Height [mAU]	Area %
1	9.586	BB	0.3002	2521.25024	123.49993	49.7508
2	18.796	BB	0.4919	2546.50879	78.61654	50.2492

(*R*,*E*)-5-(tert-butyl)-3-(2-phenylprop-1-en-1-yl)isoindolin-1-one (3da)

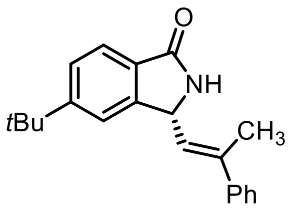

65% yield (19.7 mg, 0.065 mmol, *E/Z* > 20:1), White colour solid. **m.p.** = 248-250 ̊C. **R$_f$** = 0.23 (Pentane/Ethylacetate =1/1). **^{1}H NMR** (400 MHz, $CDCl_3$): δ 7.79 (d, *J* = 8.1 Hz, 1H), 7.53 (dd, *J* = 8.1, 1.3 Hz, 1H), 7.41 (d, *J* = 7.1 Hz, 2H), 7.32 (m, 4H), 6.75 (s, 1H), 5.60 (ABq, *J* = 9.3 Hz, 2H), 2.39 (d, *J* = 1.3 Hz, 3H), 1.35 (s, 9H). **^{13}C NMR** (101 MHz, $CDCl_3$): δ 171.1, 156.3, 147.5, 142.3, 139.7, 129.1, 128.5, 127.8, 126.1, 126.0, 125.1, 123.5, 119.8, 55.5, 35.5, 31.5, 16.8. **HRMS** (ESI/QTOF): *m/z* calcd. for $C_{21}H_{23}NNaO^+$ [M + Na]$^+$: 328.1672, found 328.1674. **IR** (ATR): 3218, 2962, 1694, 1619, 1422, 1362, 756, 697 cm^{-1}. **[α]$_D$ ^{20}** = +81.5 (c = 1.0, $CHCl_3$). **Chiral HPLC:** Chiralpak IB, 4.6 x 250 mm; 15% i-PrOH/hexane, 1.0 mL/min, 254 nm; t$_R$ (minor) = 6.25 min, t$_R$ (major) = 12.30 min, 94.5:5.5 *er*

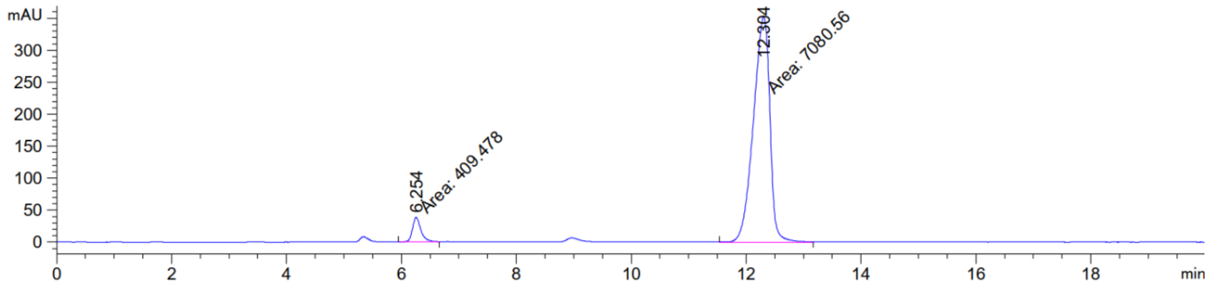

Peak #	RetTime [min]	Type	Width [min]	Area [mAU*s]	Height [mAU]	Area %
1	6.254	MM	0.1760	409.47830	38.77383	5.4670
2	12.304	MM	0.3347	7080.55615	352.58194	94.5330

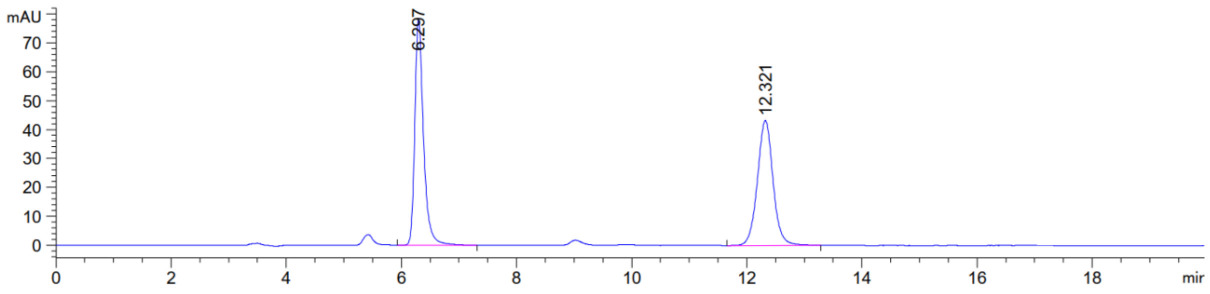

Peak #	RetTime [min]	Type	Width [min]	Area [mAU*s]	Height [mAU]	Area %
1	6.297	BB	0.1583	819.79816	78.17732	50.2117
2	12.321	BB	0.2873	812.88556	43.25325	49.7883

(*R*,*E*)-3-(2-phenylprop-1-en-1-yl)-5-(trifluoromethyl)isoindolin-1-one (3ea)

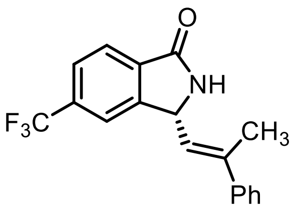

68% yield (21.6 mg, 0.068 mmol, *E/Z* = 14:1), white colour solid. **m.p.** = 188-191 ℃. **R$_f$** = 0.26 (pentane/ethylacetate =1/1). **^{1}H NMR** (400 MHz, $CDCl_3$,): δ 7.90 (d, *J* = 8.0 Hz, 1H), 7.68 (d, *J* = 7.9 Hz, 1H), 7.56 (s, 1H), 7.33 (d, *J* = 7.0 Hz, 2H), 7.23 (dt, *J* = 22.1, 8.8 Hz, 4H), 5.57 (d, *J* = 9.3 Hz, 1H), 5.46 (d, *J* = 9.3 Hz, 1H), 2.33 (s, 3H). **^{13}C NMR** (101 MHz, $CDCl_3$): δ 169.7, 147.6, 141.9, 141.2, 135.1, 134.3 (q, $J_{F\text{-}C}$ = 32.3 Hz), 128.6, 128.2, 126.0, 125.9 (q, $J_{F\text{-}C}$ = 4.0 Hz), 124.6, 123.8 (q, $J_{F\text{-}C}$ = 274 Hz), 123.4, 120.6 (q, $J_{F\text{-}C}$ = 3.7 Hz), 55.6, 16.9. **^{19}F NMR** (376 MHz, $CDCl_3$): δ -62.3. **HRMS** (ESI/QTOF): *m/z* calcd. for $C_{18}H_{15}F_3NO^+$ [M + H]$^+$: 318.1100; found: 318.1100. **IR** (ATR): 3229, 3086, 2927, 1704, 1434, 1327, 1168, 1128, 761, 696 cm^{-1}. **[α]$_D$^{20}** = +13.2 (c = 1.0, $CHCl_3$). **Chiral HPLC:** Chiralpak IB, 4.6 x 250 mm; 15% i-PrOH/hexane, 1.0 mL/min, 254 nm; t$_R$ (minor) = 7.31 min, t$_R$ (major) = 16.55 min, 98:2 *er*

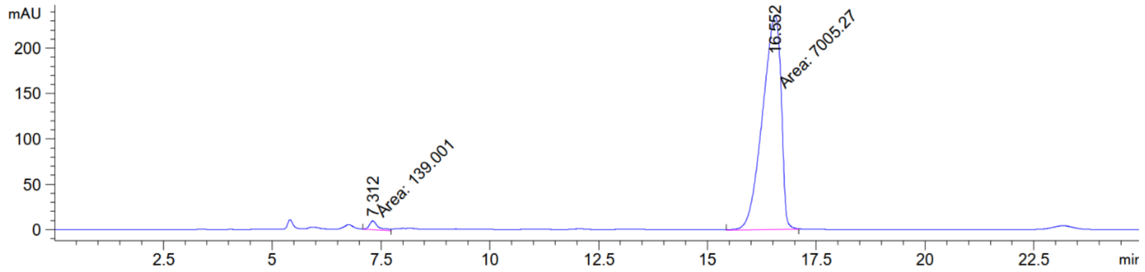

Peak #	RetTime [min]	Type	Width [min]	Area [mAU*s]	Height [mAU]	Area %
1	7.312	MM	0.2368	139.00089	9.78337	1.9456
2	16.552	MM	0.4946	7005.27002	236.05336	98.0544

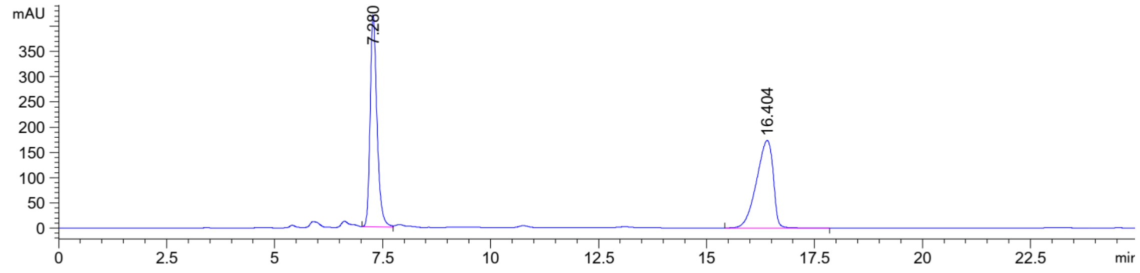

Peak #	RetTime [min]	Type	Width [min]	Area [mAU*s]	Height [mAU]	Area %
1	7.280	BV	0.1663	4611.31982	419.20218	49.8333
2	16.403	BB	0.4201	4642.16797	173.51149	50.1667

(*R*,*E*)-5-nitro-3-(2-phenylprop-1-en-1-yl)isoindolin-1-one (3fa)

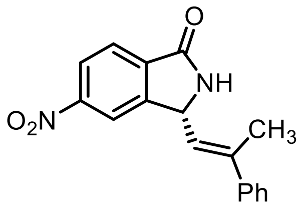

39% yield (11.5 mg, 0.039 mmol, *E/Z* > 20:1), pale brown colour solid. **R$_f$** = 0.15 (pentane/ethylacetate = 1/1). **^{1}H NMR** (400 MHz, Methanol-d$_4$): δ 8.39 (ddd, *J* = 8.3, 2.0, 0.6 Hz, 1H), 8.27 (dt, *J* = 1.7, 0.7 Hz, 1H), 7.98 (dd, *J* = 8.4, 0.6 Hz, 1H), 7.45 – 7.43 (m, 2H), 7.34 – 7.24 (m, 3H), 5.80 (d, *J* = 9.3 Hz, 1H), 5.48 (dq, *J* = 9.3, 1.4 Hz, 1H), 2.42 (d, *J* = 1.4 Hz, 3H). **^{13}C NMR** (101 MHz, Methanol-d$_4$): δ 170.5, 152.2, 150.2, 143.6, 143.1, 138.4, 129.4, 128.9, 127.0, 125.6, 125.1, 123.9, 112.0, 56.9, 16.9. **HRMS** (ESI/QTOF): *m/z* calcd. for $C_{17}H_{15}N_2O_3^+$ [M + H]$^+$: 295.1077; found: 295.1083. **IR** (ATR): 3233, 2923, 2853, 1702, 1530, 1345, 761, 735, 696 cm^{-1}. **[α]$_D$^{20}** = +115.6 (c = 0.5, $CHCl_3$). **Chiral HPLC:** Chiralpak IG, 4.6 x 250 mm; 20% i-PrOH/hexane, 1.0 mL/min, 254 nm; t$_R$ (major) = 10.16 min, t$_R$ (minor) = 11.07 min, 97.5:2.5 *er*

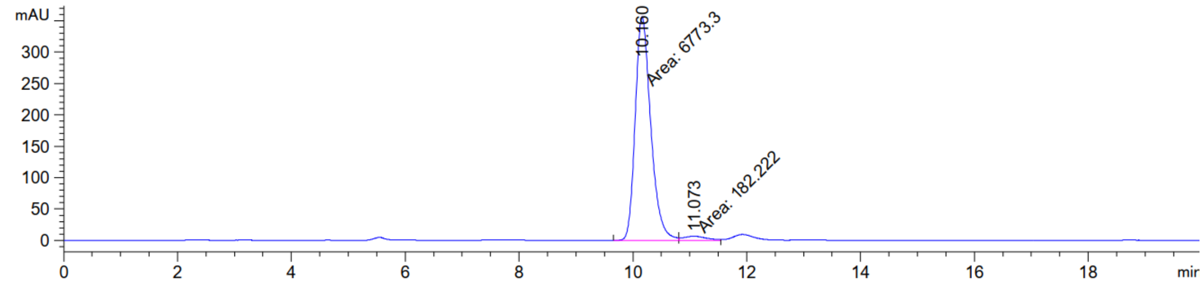

Peak #	RetTime [min]	Type	Width [min]	Area [mAU*s]	Height [mAU]	Area %
1	10.160	MF	0.3168	6773.29834	356.34131	97.3802
2	11.073	FM	0.4680	182.22153	6.48876	2.6198

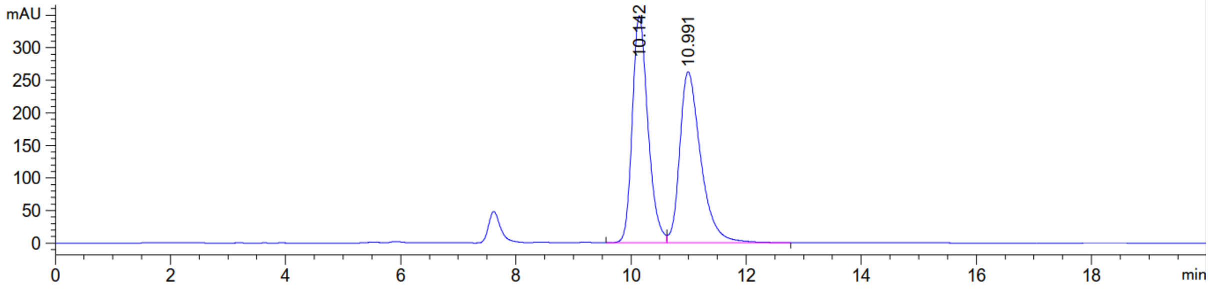

Peak #	RetTime [min]	Type	Width [min]	Area [mAU*s]	Height [mAU]	Area %
1	10.142	BV	0.2889	6597.09424	348.46585	49.7700
2	10.991	VB	0.3840	6658.08057	262.40308	50.2300

(*R*,*E*)-1-oxo-3-(2-phenylprop-1-en-1-yl)isoindoline-5-carbonitrile (3ga)

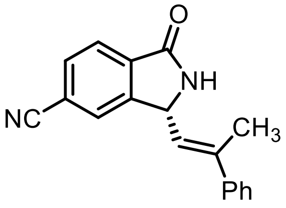

63% yield (17.4 mg, 0.063 mmol, *E/Z* > 20:1), pale yellow colour solid. **m.p.** = 166-168 ℃. **R$_f$** = 0.18 (pentane/ethylacetate = 1/1). **^{1}H NMR** (400 MHz, $CDCl_3$,): δ 7.96 (d, *J* = 7.7 Hz, 1H), 7.77 (dd, *J* = 7.8, 1.3 Hz, 1H), 7.70 (d, *J* = 1.2 Hz, 1H), 7.41 – 7.36 (m, 2H), 7.36 – 7.29 (m, 3H), 7.23 (s, 1H), 5.63 (d, *J* = 9.3 Hz, 1H), 5.53 – 5.48 (m, 1H), 2.39 (d, *J* = 1.3 Hz, 3H). **^{13}C NMR** (101 MHz, $CDCl_3$): δ 168.9, 147.5, 141.6, 141.5, 135.6, 132.4, 128.5, 128.1, 127.3, 125.9, 124.7, 122.8, 118.1, 115.7, 55.3, 16.8. **HRMS** (ESI/QTOF): *m/z* calcd. for $C_{18}H_{14}N_2NaO^+$ $[M + Na]^+$: 297.0998, found 297.0997. **IR** (ATR): 3219, 3081, 2924, 2231, 1701, 1421, 761, 696 cm^{-1}. **[α]$_D$ ^{20}** = +78.5 (c = 0.7, $CHCl_3$). **Chiral HPLC:** Chiralpak IG, 4.6 x 250 mm; 20% i-PrOH/hexane, 1.0 mL/min, 254 nm; t_R (major) = 11.02 min, t_R (minor) = 12.89 min, 89:11 *er*

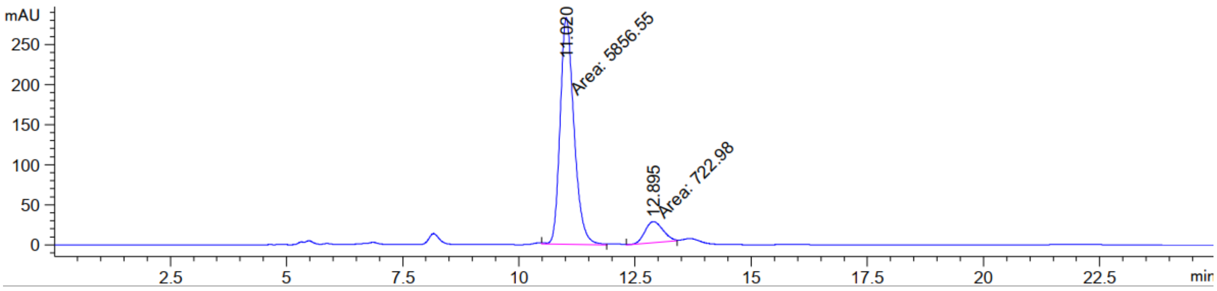

Peak #	RetTime [min]	Type	Width [min]	Area [mAU*s]	Height [mAU]	Area %
1	11.020	MM	0.3454	5856.55176	282.63638	89.0117
2	12.895	MM	0.4548	722.98047	26.49584	10.9883

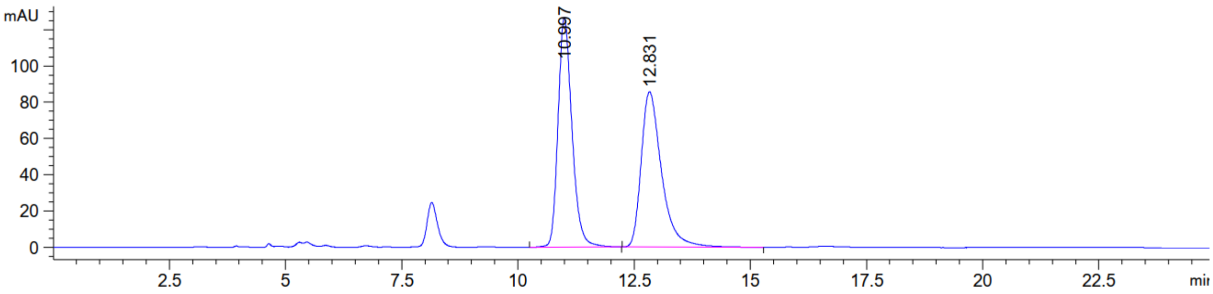

Peak #	RetTime [min]	Type	Width [min]	Area [mAU*s]	Height [mAU]	Area %
1	10.997	BB	0.3200	2651.18555	126.77229	50.3401
2	12.831	BB	0.4600	2615.36377	85.71484	49.6599

(*R*,*E*)-5-fluoro-3-(2-phenylprop-1-en-1-yl)isoindolin-1-one (3ha)

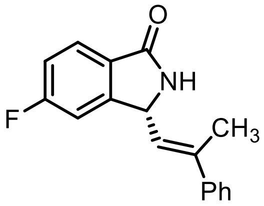

70% yield (18.8 mg, 0.070 mmol, *E/Z* > 20:1), white colour solid. **m.p. =** 153-155 ̊C. **R_f** = 0.28 (pentane/ethylacetate = 1/1). **^{1}H NMR** (400 MHz, Methanol-d$_4$): δ 7.81 (dd, *J* = 8.4, 5.0 Hz, 1H), 7.44 – 7.41 (m, 2H), 7.34 – 7.21 (m, 5H), 5.66 (d, *J* = 9.3 Hz, 1H), 5.45 (dd, *J* = 9.3, 1.4 Hz, 1H), 2.38 (d, *J* = 1.4 Hz, 3H). **^{13}C NMR** (101 MHz, Methanol-d$_4$): 171.9, 167.1 (d, $J_{F\text{-}C}$ = 251.5 Hz), 151.9 (d, $J_{F\text{-}C}$ = 10.1 Hz), 143.8, 142.3, 129.4, 129.2 (d, $J_{F\text{-}C}$ = 2.0 Hz), 128.7, 126.9, 126.5 (d, $J_{F\text{-}C}$ = 10.1 Hz), 124.8, 117.6 (d, $J_{F\text{-}C}$ = 24.2 Hz), 111.7 (d, $J_{F\text{-}C}$ = 24.2 Hz), 56.6 (d, $J_{F\text{-}C}$ = 2.0 Hz), 16.8. **^{19}F NMR** (376 MHz, Methanol-d$_4$): -108.1. **HRMS** (ESI/QTOF): *m/z* calcd. for $C_{17}H_{14}FNNaO^+$ [M + Na]$^+$: 290.0952; found: 290.0944. **IR** (ATR): 3228, 2923, 1696, 1623, 1479, 1266, 760, 696 cm^{-1}. **$[\alpha]_D^{20}$** = +12.2 (c = 0.5, $CHCl_3$). **Chiral HPLC:** Chiralpak IB, 4.6 x 250 mm; 15% i-PrOH/hexane, 1.0 mL/min, 254 nm; t_R (minor) = 8.11 min, t_R (major) = 18.77 min, 98:2 *er*

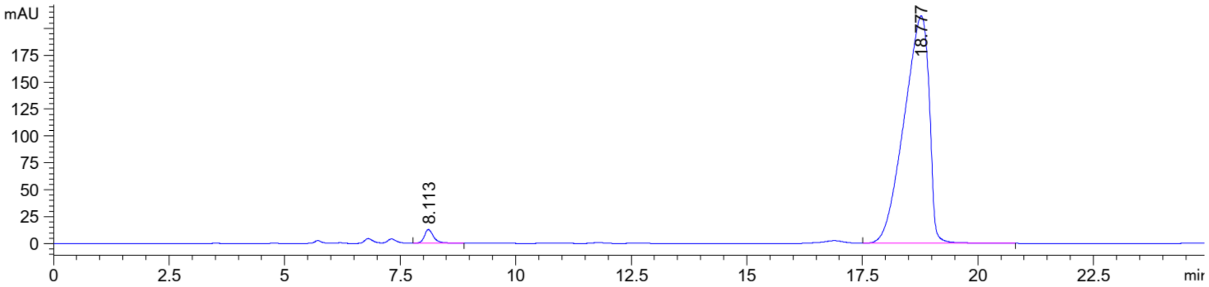

Peak #	RetTime [min]	Type	Width [min]	Area [mAU*s]	Height [mAU]	Area %
1	8.113	BB	0.2049	174.18947	12.62986	2.1358
2	18.777	BB	0.5844	7981.34863	211.45807	97.8642

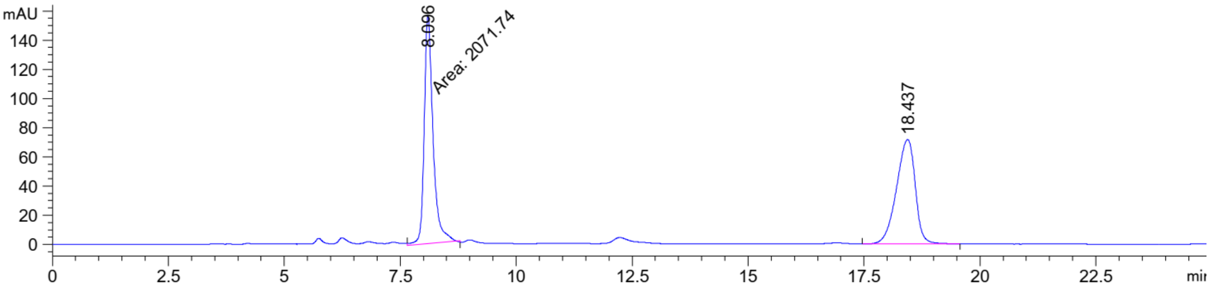

Peak #	RetTime [min]	Type	Width [min]	Area [mAU*s]	Height [mAU]	Area %
1	8.096	MM	0.2210	2071.74341	156.23068	50.7654
2	18.437	BB	0.4396	2009.27283	71.56451	49.2346

(*R*,*E*)-5-chloro-3-(2-phenylprop-1-en-1-yl)isoindolin-1-one (3ia)

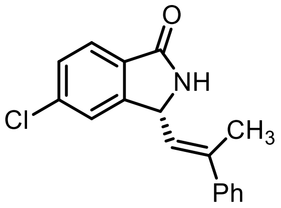

66% yield (18.8 mg, 0.066 mmol, *E/Z* = 20:1), white colour solid. **m.p.** = 157-161 ˚C. **R$_f$** = 0.34 (pentane/ethylacetate = 1/1). **^{1}H NMR** (400 MHz, Methanol-d$_4$): δ 7.75 (d, *J* = 8.1 Hz, 1H), 7.52 (dd, *J* = 8.1, 1.7 Hz, 1H), 7.48-7.47 (m, 1H), 7.42 – 7.39 (m, 2H), 7.33 – 7.28 (m, 2H), 7.27 – 7.22 (m, 1H), 5.65 (d, *J* = 9.3 Hz, 1H), 5.43 (dq, *J* = 9.4, 1.4 Hz, 1H), 2.37 (d, *J* = 1.4 Hz, 3H). **^{13}C NMR** (101 MHz, Methanol-d$_4$): δ 171.8, 150.9, 143.7, 142.3, 139.7, 131.7, 130.0, 129.4, 128.7, 126.9, 125.8, 124.8, 124.6, 56.6, 16.8. **HRMS** (ESI+/QTOF): *m/z* calcd. for $C_{17}H_{14}ClNNaO^+$ [M + Na]$^+$: 306.0656, found 306.0654. **IR** (ATR): 3221, 2922, 1693, 1610, 1421, 1191, 759, 696 cm^{-1}. **[α]$_D$ ^{20}** = +102.9 (c = 1.5, $CHCl_3$). **Chiral HPLC:** Chiralpak IB, 4.6 x 250 mm; 15% i-PrOH/hexane, 1.0 mL/min, 254 nm; t_R (minor) = 8.22 min, t_R (major) = 18.75 min, 98:2 *er*

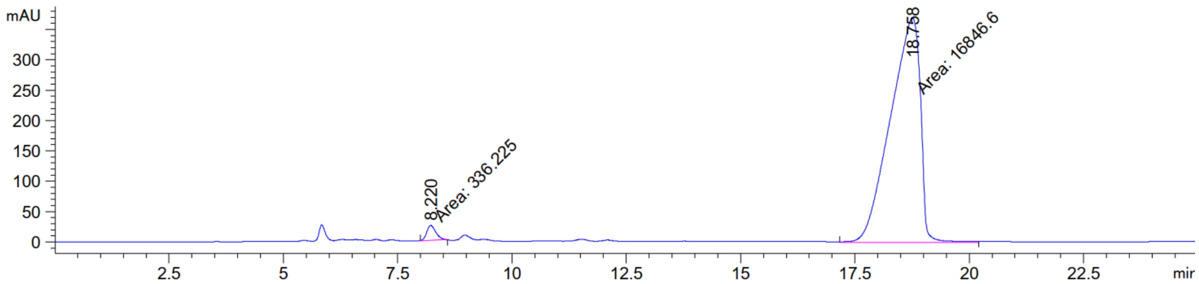

Peak #	RetTime [min]	Type	Width [min]	Area [mAU*s]	Height [mAU]	Area %
1	8.220	MM	0.2269	336.22455	24.69296	1.9568
2	18.758	MM	0.7581	1.68466e4	370.38498	98.0432

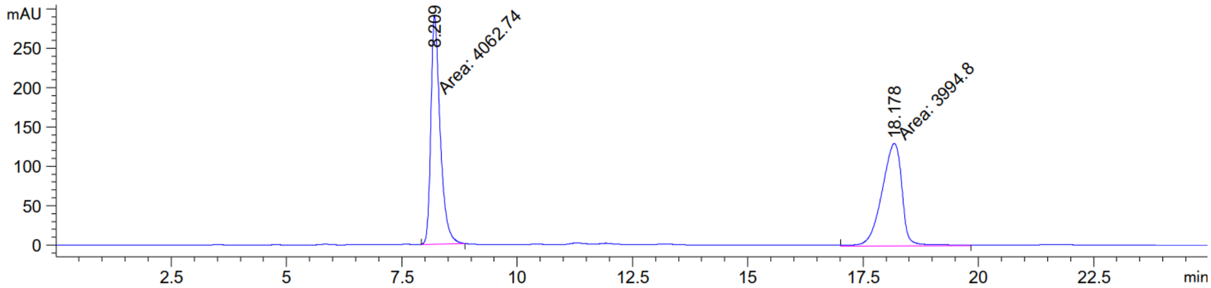

Peak #	RetTime [min]	Type	Width [min]	Area [mAU*s]	Height [mAU]	Area %
1	8.209	MM	0.2334	4062.74243	290.14890	50.4216
2	18.178	MM	0.5114	3994.80322	130.19563	49.5784

(*R*,*E*)-5-bromo-3-(2-phenylprop-1-en-1-yl)isoindolin-1-one (3ja)

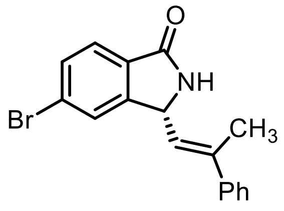

68% yield (22.3 mg, 0.068 mmol, *E/Z* = 18:1), white colour solid. **m.p.** = 184-187 ̊C. **R**$_f$ = 0.33 (pentane/ethylacetate = 1/1). **^{1}H NMR** (400 MHz, Methanol-d$_4$): δ 7.69 (m, 2H), 7.64 (m, 1H), 7.45 – 7.40 (m, 2H), 7.35 – 7.29 (m, 2H), 7.29 – 7.21 (m, 1H), 5.67 (dd, *J* = 9.3, 0.8 Hz, 1H), 5.47 – 5.41 (m, 1H), 2.37 (d, *J* = 1.4 Hz, 3H). **^{13}C NMR** (101 MHz, Methanol-d$_4$): δ 171.9, 151.1, 143.7, 142.4, 133.0, 132.2, 129.4, 128.8, 127.9, 127.9, 127.0, 126.0, 124.6, 56.6, 16.9. **HRMS** (ESI+/QTOF): *m/z* calcd. for $C_{17}H_{15}BrNO^+$ [M + H]$^+$: 328.0332; found: 328.0338. **IR** (ATR): 3210, 3080, 1694, 1606, 1417, 1141, 758, 696 cm^{-1}. **[α]$_D$ ^{20}** = +121.3 (c = 1.5, $CHCl_3$). **Chiral HPLC:** Chiralpak IB, 4.6 x 250 mm; 15% i-PrOH/hexane, 1.0 mL/min, 254 nm; t$_R$ (minor) = 7.86 min, t$_R$ (major) = 18.53 min, 98.5:1.5 *er*

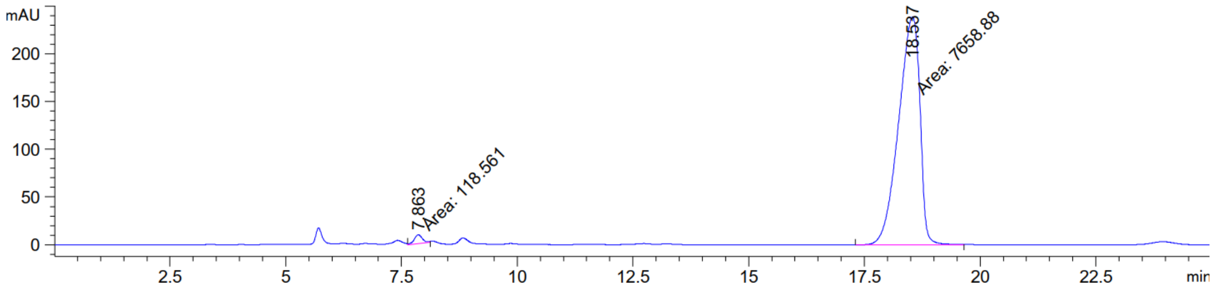

Peak #	RetTime [min]	Type	Width [min]	Area [mAU*s]	Height [mAU]	Area %
1	7.863	MM	0.2090	118.56075	9.45505	1.5244
2	18.537	MM	0.5340	7658.87988	239.03596	98.4756

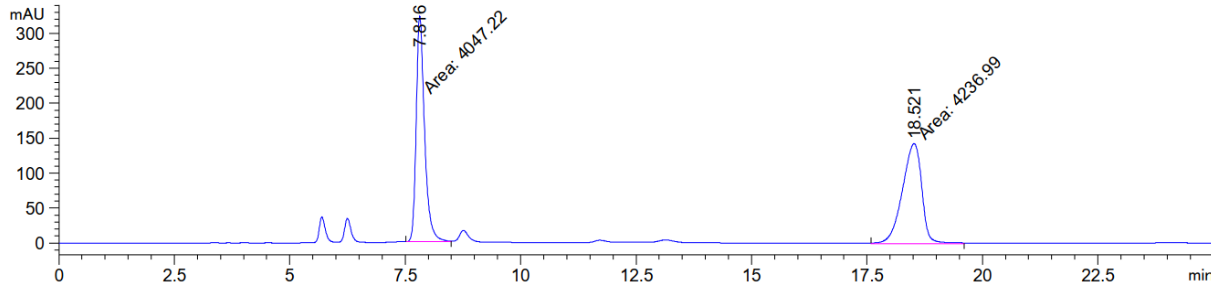

Peak #	RetTime [min]	Type	Width [min]	Area [mAU*s]	Height [mAU]	Area %
1	7.816	MM	0.2091	4047.21704	322.60056	48.8546
2	18.521	MM	0.4927	4236.98584	143.33292	51.1454

(*R*,*E*)-6-methyl-3-(2-phenylprop-1-en-1-yl)isoindolin-1-one (3ka)

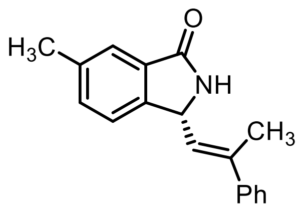

71% yield (18.6 mg, 0.071 mmol, *E/Z* > 20:1), white colour solid. **m.p.** = 198-200 ℃. **R$_f$** = 0.25 (pentane/ethylacetate = 1/1). **^{1}H NMR** (400 MHz, Methanol-d$_4$): δ 7.59 (s, 1H), 7.45 (dd, *J* = 7.6, 0.8 Hz, 1H), 7.41 – 7.38 (m, 2H), 7.34 (d, *J* = 7.8 Hz, 1H), 7.32 – 7.28 (m, 2H), 7.25 – 7.21 (m, 1H), 5.59 (d, *J* = 9.3 Hz, 1H), 5.42 (dq, *J* = 9.3, 1.4 Hz, 1H), 2.44 (s, 3H), 2.36 (d, *J* = 1.4 Hz, 3H). **^{13}C NMR** (101 MHz, Methanol-d$_4$): δ 173.2, 146.4, 143.9, 141.3, 139.8, 134.5, 133.0, 129.4, 128.6, 126.9, 125.7, 124.4, 124.2, 56.7, 21.3, 16.8. **HRMS** (ESI+/QTOF): *m/z* calcd. for $C_{18}H_{17}NNaO^+$ $[M + Na]^+$: 286.1202; found: 286.1202. **IR** (ATR): 3212, 3058, 2922, 1690, 1624, 1491, 1444, 759, 697 cm^{-1}. **[α]$_D$ ^{20}** = +16.3 (c = 1.0, $CHCl_3$). **Chiral HPLC:** Chiralpak IB, 4.6 x 250 mm; 15% i-PrOH/hexane, 1.0 mL/min, 254 nm; t$_R$ (minor) = 6.74 min, t$_R$ (major) = 14.03 min, 96.5:3.5 *er*

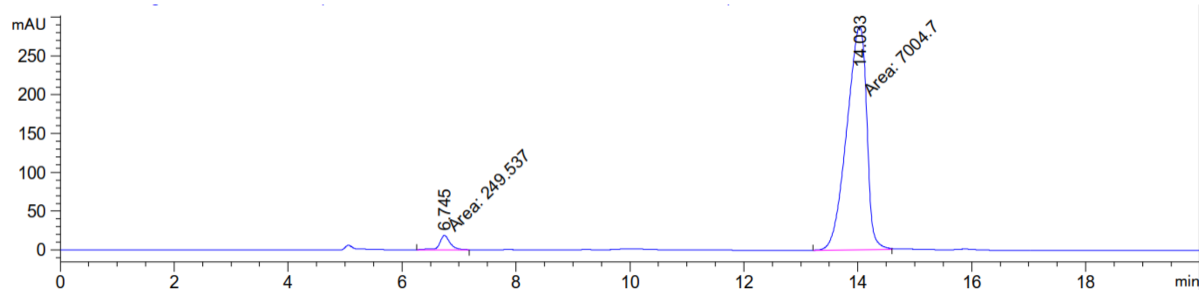

Peak #	RetTime [min]	Type	Width [min]	Area [mAU*s]	Height [mAU]	Area %
1	6.745	MM	0.2223	249.53748	18.71151	3.4399
2	14.033	MM	0.4056	7004.70313	287.83148	96.5601

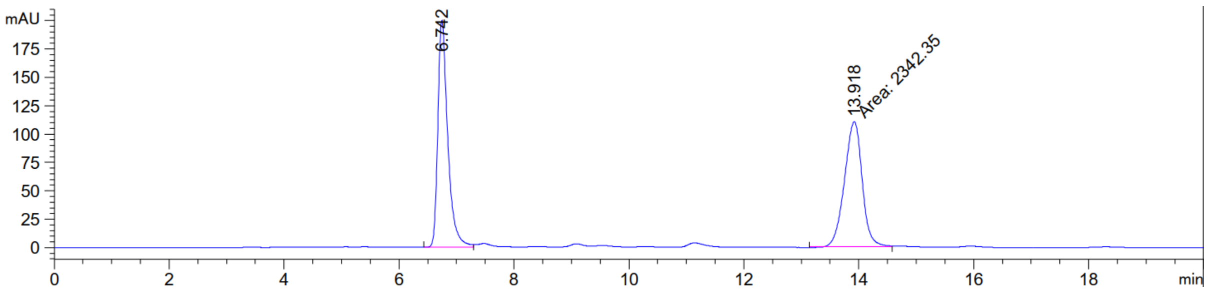

Peak #	RetTime [min]	Type	Width [min]	Area [mAU*s]	Height [mAU]	Area %
1	6.742	BV	0.1795	2389.96729	199.87906	50.5031
2	13.918	MM	0.3548	2342.35376	110.02644	49.4969

(*R*,*E*)-3-(2-phenylprop-1-en-1-yl)-6-(trifluoromethyl)isoindolin-1-one (3la)

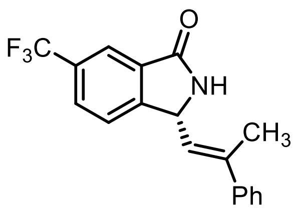

62% yield (19.7 mg, 0.062 mmol, *E/Z* > 20:1), white colour solid. **m.p. =** 169-172 ℃. **R**$_f$ = 0.47 (pentane/ethylacetate = 1/1). **^{1}H NMR** (400 MHz, Methanol-d$_4$): δ 8.06 (s, 1H), 7.93(dd, *J* = 8.0, 0.8 Hz, 1H), 7.69 (d, *J* = 8.0 Hz, 1H), 7.43 – 7.40 (m, 2H), 7.32 – 7.22 (m, 3H), 5.77 (d, *J* = 9.3 Hz, 1H), 5.46 (dq, *J* = 9.4, 1.4 Hz, 1H), 2.40 (d, *J* = 1.4 Hz, 3H). **^{13}C NMR** (101 MHz, Methanol-d$_4$): δ 171.3, 152.8, 143.7, 142.6, 132.1 (q, *J* = 32.8 Hz), 130.2 (q, *J* = 3.6 Hz), 129.4, 128.8, 126.9, 125.7, 125.4 (q, *J* = 272.6 Hz), 124.3, 124.04, 121.4 (q, *J* = 4.0 Hz), 57.0, 16.9. **^{19}F NMR** (376 MHz, Methanol-d$_4$): -63.7. **HRMS** (ESI+/QTOF): *m/z* calcd. for $C_{18}H_{14}F_3NNaO^+$ $[M + Na]^+$: 340.0920, found 340.0917. **IR** (ATR): 3220, 3062, 2924, 1694, 1632, 1316, 1268, 1167, 1124, 1097, 758, 696 cm^{-1}. **[α]$_D$^{20}** = +4.7 (c = 0.5, $CHCl_3$). **Chiral HPLC:** Chiralpak IB, 4.6 x 250 mm; 15% i-PrOH/hexane, 1.0 mL/min, 254 nm; t_R (minor) = 6.72 min, t_R (major) = 19.40 min, 95.5:4.5 *er*

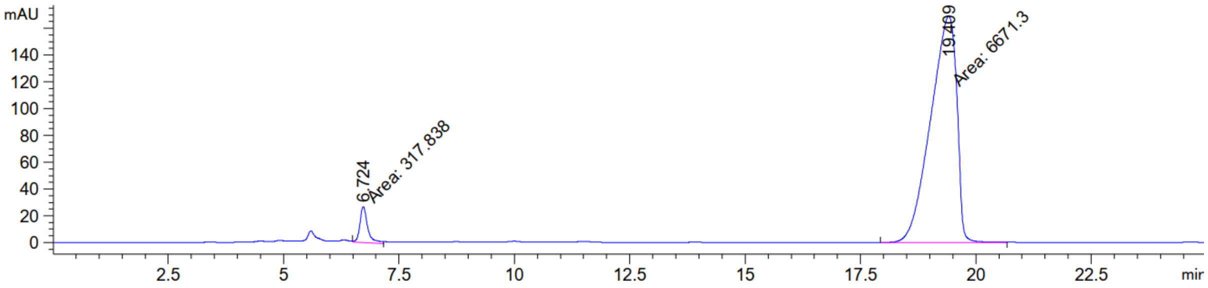

Peak #	RetTime [min]	Type	Width [min]	Area [mAU*s]	Height [mAU]	Area %
1	6.724	MM	0.1969	317.83771	26.90661	4.5476
2	19.409	MM	0.6575	6671.30469	169.09729	95.4524

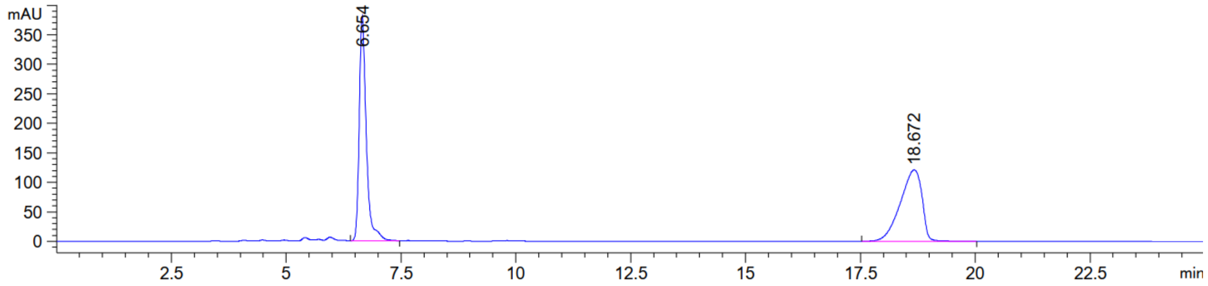

Peak #	RetTime [min]	Type	Width [min]	Area [mAU*s]	Height [mAU]	Area %
1	6.654	BB	0.1589	4079.07739	381.05536	50.6601
2	18.672	BB	0.5110	3972.78125	121.00914	49.3399

(*R*,*E*)-7-fluoro-3-(2-phenylprop-1-en-1-yl)isoindolin-1-one (3ma)

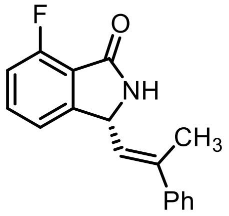

44% yield (11.80 mg, 0.044 mmol, *E/Z* > 20:1), white colour solid. **m.p.** = 190-193 ℃. **R$_f$** = 0.40 (pentane/ethylacetate = 1/1). **^{1}H NMR** (400 MHz, Methanol-d$_4$) δ 7.65 – 7.60 (m, 1H), 7.42 – 7.40 (m, 2H), 7.33 – 7.23 (m, 4H), 7.19 – 7.14 (m, 1H), 5.66 (d, *J* = 9.3 Hz, 1H), 5.45 (dq, *J* = 9.3, 1.4 Hz, 1H), 2.37 (d, *J* = 1.4 Hz, 3H). **^{13}C NMR** (101 MHz, Methanol-d$_4$): δ 169.6 (d, *J* = 2.2 Hz), 160.3 (d, *J* = 259.7 Hz), 152.1 (d, *J* = 2.4 Hz), 143.8, 142.0, 135.8 (d, *J* = 7.7 Hz), 129.4, 128.7, 126.9, 125.0, 120.7 (d, *J* = 4.2 Hz), 116.4 (d, *J* = 19.5 Hz), 56.7, 16.8. **^{19}F NMR** (376 MHz, Methanol-d$_4$): -120.9. **HRMS** (ESI+/QTOF): *m/z* calcd. for $C_{17}H_{14}FNNaO^+$ [M + Na]$^+$: 290.0952; found: 290.0958. **IR** (ATR): 3222, 3082, 1694, 1625, 1480, 1250, 758, 696 cm^{-1}. **[α]$_D$ ^{20}** = -5.8 (c = 0.8, $CHCl_3$). **Chiral HPLC:** Chiralpak IG, 4.6 x 250 mm; 20% i-PrOH/hexane, 1.0 mL/min, 254 nm; t$_R$ (minor) = 15.38 min, t$_R$ (major) = 20.00 min, 97.5:2.5 *er*

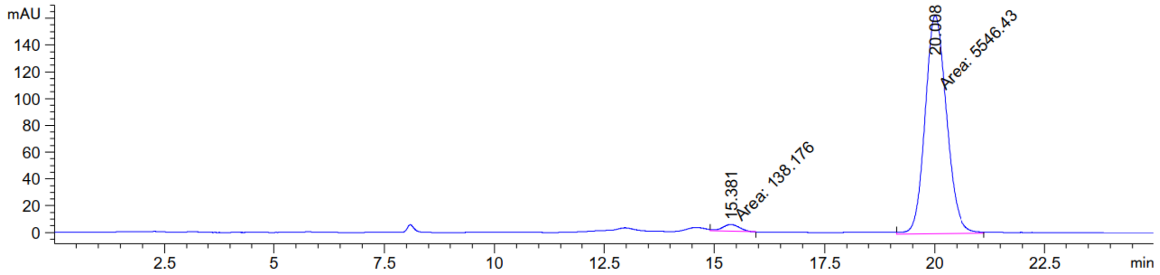

Peak #	RetTime [min]	Type	Width [min]	Area [mAU*s]	Height [mAU]	Area %
1	15.381	MM	0.4576	138.17555	5.03293	2.4307
2	20.008	MM	0.5673	5546.43262	162.94489	97.5693

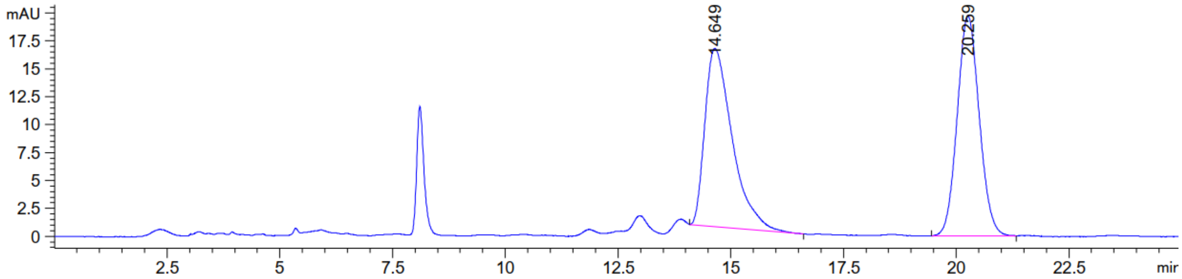

Peak #	RetTime [min]	Type	Width [min]	Area [mAU*s]	Height [mAU]	Area %
1	14.649	BB	0.6295	674.60291	15.94729	50.2270
2	20.259	BB	0.5271	668.50641	19.73866	49.7730

(*R*,*E*)-3-(2-phenylprop-1-en-1-yl)-2,3-dihydro-1H-benzo[*f*]isoindol-1-one (3na)

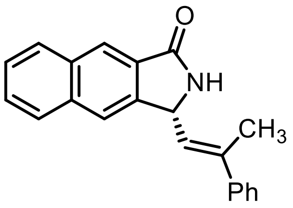

42% yield (12.60 mg, 0.042 mmol, *E/Z* > 20:1), white colour solid. **m.p.** = 265-270 ℃. **R$_f$** = 0.22 (pentane/ethylacetate = 1/1). **^{1}H NMR** (400 MHz, DMSO-d$_6$): δ 8.94 (s, 1H), 8.31 (s, 1H), 8.15 (d, *J* = 8.0 Hz, 1H), 8.06 (d, *J* = 8.0 Hz, 1H), 7.97 (s, 1H), 7.65 – 7.55 (m, 2H), 7.52 – 7.45 (m, 2H), 7.34 (t, *J* = 7.2, 2H), 7.28 (d, *J* = 7.2 Hz, 1H), 5.79 (d, *J* = 9.1 Hz, 1H), 5.63 (d, *J* = 9.1 Hz, 1H), 2.41 (s, 3H). **^{13}C NMR** (101 MHz, DMSO-d6): δ 169.0, 142.6, 141.8, 138.0, 134.9, 132.6, 130.5, 129.3, 128.4, 128.1, 127.50, 127.48, 126.25, 126.20, 125.6, 122.9, 122.1, 54.4, 16.2. **HRMS** (ESI+/QTOF): *m/z* calcd. for $C_{21}H_{17}NNaO^+$ [M + Na]$^+$: 322.1202, found 322.1203. **IR** (ATR): 3202, 2924, 1693, 1660, 1642, 1199, 760, 695 cm^{-1}. **[α]$_D$ ^{20}** = +123.3 (c = 0.2, $CHCl_3$). **Chiral HPLC:** Chiralpak IG, 4.6 x 250 mm; 30% i-PrOH/hexane, 1.0 mL/min, 254 nm; t$_R$ (major) = 17.60 min, t$_R$ (minor) = 25.04 min, 98:2 *er*

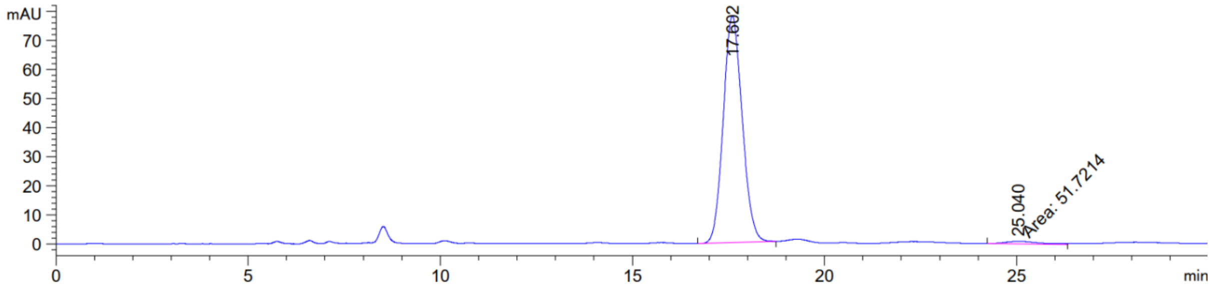

Peak #	RetTime [min]	Type	Width [min]	Area [mAU*s]	Height [mAU]	Area %
1	17.602	BB	0.5338	2668.32886	77.87314	98.0985
2	25.040	MM	1.0040	51.72141	8.58560e-1	1.9015

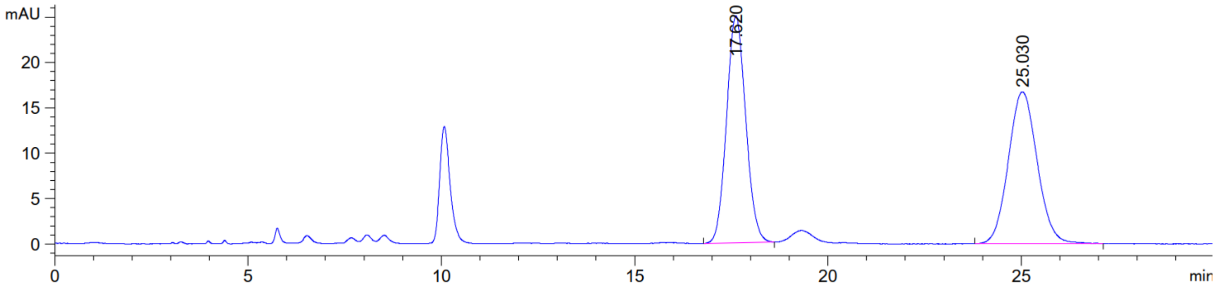

Peak #	RetTime [min]	Type	Width [min]	Area [mAU*s]	Height [mAU]	Area %
1	17.620	BB	0.5403	862.88422	25.02195	49.8666
2	25.030	BB	0.8070	867.49933	16.72809	50.1334

(*R*,*E*)-3-(2-(p-tolyl)prop-1-en-1-yl)isoindolin-1-one (3ab)

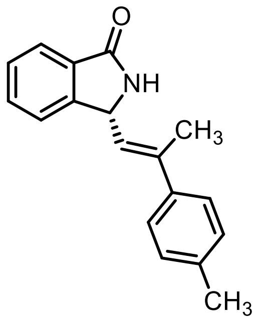

71% yield (18.60 mg, 0.071 mmol, *E/Z* > 20:1), white colour solid. **R$_f$** = 0.25 (pentane/ethylacetate = 1/1). **^{1}H NMR** (400 MHz, Methanol-d$_4$) δ 7.79 (dt, *J* = 7.5, 1.0 Hz, 1H), 7.62 (td, *J* = 7.5, 1.2 Hz, 1H), 7.50 (t, *J* = 7.4 Hz, 1H), 7.48 – 7.44 (m, 1H), 7.32 – 7.26 (m, 2H), 7.11 (d, *J* = 8.0 Hz, 2H), 5.63 (d, *J* = 9.3 Hz, 1H), 5.40 (dq, *J* = 9.3, 1.4 Hz, 1H), 2.35 (d, *J* = 1.4 Hz, 3H), 2.29 (s, 3H). **^{13}C NMR** (101 MHz, Methanol-d4) δ 173.0, 149.2, 141.4, 140.9, 138.5, 133.5, 132.9, 130.0, 129.4, 126.8, 124.6, 124.5, 124.3, 57.0, 21.1, 16.7. **HRMS** (ESI+/QTOF): *m/z* calcd. for $C_{18}H_{17}NNaO^{+}$ [M + Na]$^{+}$: 286.1202; found: 286.1202. **IR** (ATR): 3218, 2922, 1691, 1612, 1468, 814, 756, 693 cm^{-1}. **[α]$_D$^{20}** = +16.8 (c = 0.7, $CHCl_3$). **Chiral HPLC:** Chiralpak IB, 4.6 x 250 mm; 15% i-PrOH/hexane, 1.0 mL/min, 254 nm; t$_R$ (minor) = 7.14 min, t$_R$ (major) = 9.86 min, 96.5:3.5 *er*

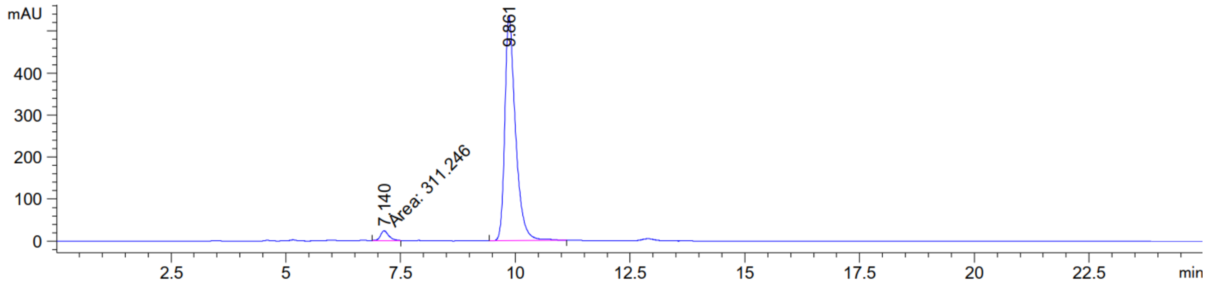

Peak #	RetTime [min]	Type	Width [min]	Area [mAU*s]	Height [mAU]	Area %
1	7.140	MM	0.2164	311.24557	23.97286	3.4660
2	9.861	BB	0.2386	8668.69922	536.12347	96.5340

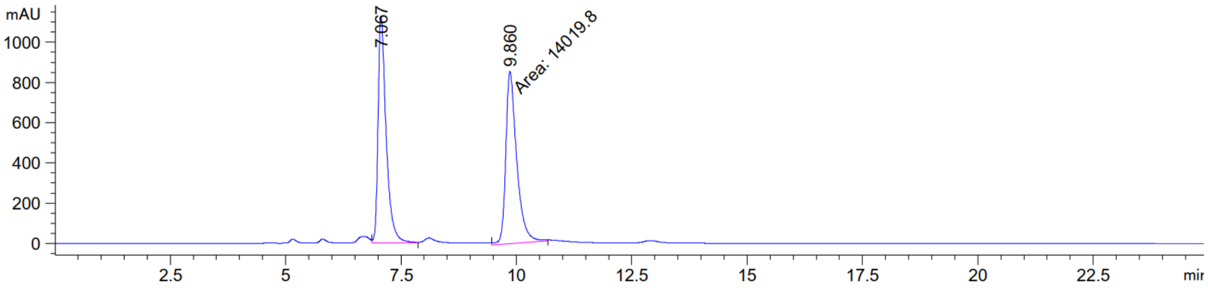

Peak #	RetTime [min]	Type	Width [min]	Area [mAU*s]	Height [mAU]	Area %
1	7.067	VV	0.1801	1.37355e4	1127.63940	49.4878
2	9.860	MM	0.2734	1.40198e4	854.78961	50.5122

(*R*,*E*)-3-(2-(4-fluorophenyl)prop-1-en-1-yl)isoindolin-1-one (3ac)

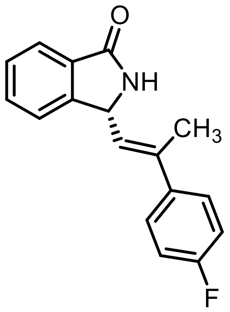

68% yield (18.20 mg, 0.068 mmol, *E/Z* > 20:1), white colour solid. **R$_f$** = 0.26 (pentane/ethylacetate = 1/1). **^{1}H NMR** (400 MHz, Methanol-d$_4$): δ 7.79 (d, *J* = 7.6 Hz, 1H), 7.62 (td, *J* = 7.5, 1.0 Hz, 1H), 7.53 – 7.41 (m, 4H), 7.06 – 7.00 (m, 2H), 5.64 (d, *J* = 9.3 Hz, 1H), 5.42 (dq, *J* = 9.4, 1.4 Hz, 1H), 2.37 (d, *J* = 1.4 Hz, 3H). **^{13}C NMR** (101 MHz, Methanol-d4): δ 173.0, 163.8 (d, *J* = 246.2 Hz), 149.1, 140.5, 140.1(d, *J* = 3.2 Hz), 133.5, 132.9, 129.5, 128.8 (d, *J* = 8.1 Hz), 128.7, 125.58, 125.57, 124.5, 124.3, 116.0 (d, *J* = 21.8 Hz), 56.9, 16.9. **^{19}F NMR** (376 MHz, Methanol-d$_4$): -117.2. **HRMS** (ESI+/QTOF): *m/z* calcd. for $C_{17}H_{14}FNNaO^+$ [M + Na]$^+$: 290.0952; found: 290.0952. **IR** (ATR): 3221, 3076, 2924, 1692, 1601, 1509, 1468, 1230, 830, 750, 693 cm^{-1}. **[α]$_D$ ^{20}** = +10.5 (c = 0.5, $CHCl_3$). **Chiral HPLC:** Chiralpak IB, 4.6 x 250 mm; 15% i-PrOH/hexane, 1.0 mL/min, 254 nm; t$_R$ (minor) = 7.72 min, t$_R$ (major) = 10.48 min, 96:4 *er*

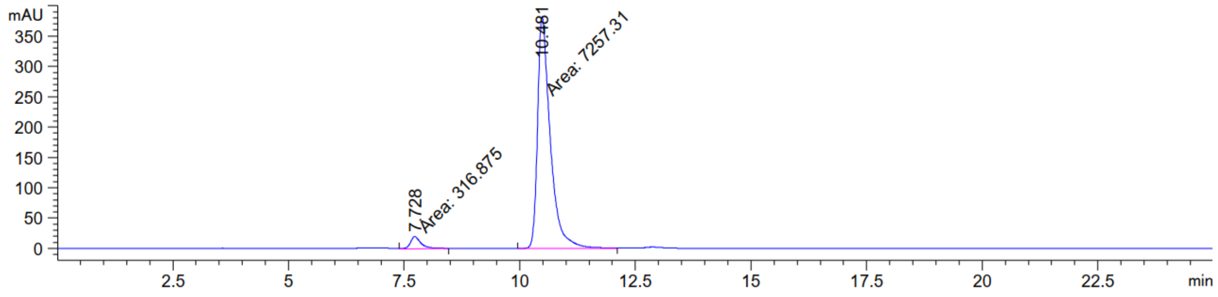

Peak #	RetTime [min]	Type	Width [min]	Area [mAU*s]	Height [mAU]	Area %
1	7.728	MM	0.2670	316.87506	19.77646	4.1836
2	10.481	MM	0.3170	7257.30664	381.54849	95.8164

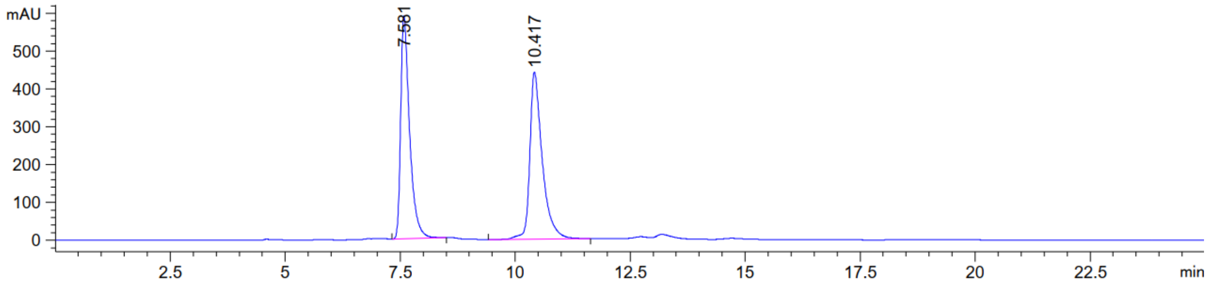

Peak #	RetTime [min]	Type	Width [min]	Area [mAU*s]	Height [mAU]	Area %
1	7.581	BB	0.1987	7924.89014	589.94366	48.8897
2	10.417	BB	0.2747	8284.85254	442.05597	51.1103

(*R*,*E*)-3-(2-(4-bromophenyl)prop-1-en-1-yl)isoindolin-1-one (3ad)

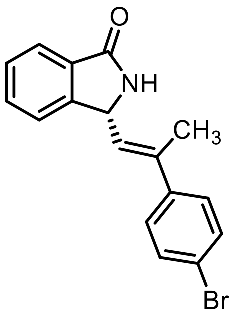

62% yield (20.20 mg, 0.062 mmol, *E/Z* > 20:1), white colour solid. **m.p. =** 170-173 ℃. **R$_f$** = 0.27 (pentane/ethylacetate = 1/1). **^{1}H NMR** (400 MHz, Methanol-d$_4$) δ 7.79 (dt, *J* = 7.6, 1.0 Hz, 1H), 7.62 (td, *J* = 7.5, 1.2 Hz, 1H), 7.51 (t, *J* = 7.4 Hz, 1H), 7.49 – 7.43 (m, 3H), 7.35 – 7.32 (m, 2H), 5.64 (d, *J* = 9.2 Hz, 1H), 5.48 (dq, *J* = 9.3, 1.4 Hz, 1H), 2.36 (d, *J* = 1.4 Hz, 3H). **^{13}C NMR** (101 MHz, Methanol-d$_4$) δ 173.0, 148.9, 142.9, 140.4, 133.5, 132.9, 132.5, 129.5, 128.8, 126.3, 124.5, 124.3, 122.4, 56.8, 16.6. **HRMS** (ESI+/QTOF): *m/z* calcd. for $C_{17}H_{14}BrNNaO^+$ [M + Na]$^+$: 350.0151; found: 350.0152. **IR** (ATR): 3208, 1691, 1485, 1468, 1007, 818, 748 cm^{-1}. **[α]$_D$ ^{20}** = +31.6 (c = 0.7, $CHCl_3$). **Chiral HPLC:** Chiralpak IB, 4.6 x 250 mm; 15% i-PrOH/hexane, 1.0 mL/min, 254 nm; t$_R$ (minor) = 8.54 min, t$_R$ (major) = 11.87 min, 96.5:3.5 *er*

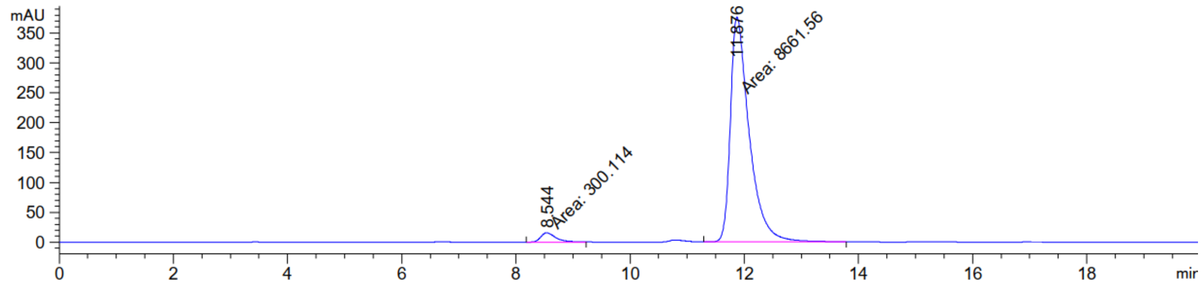

Peak #	RetTime [min]	Type	Width [min]	Area [mAU*s]	Height [mAU]	Area %
1	8.544	MM	0.3170	300.11365	15.78048	3.3489
2	11.876	MM	0.3833	8661.55762	376.63425	96.6511

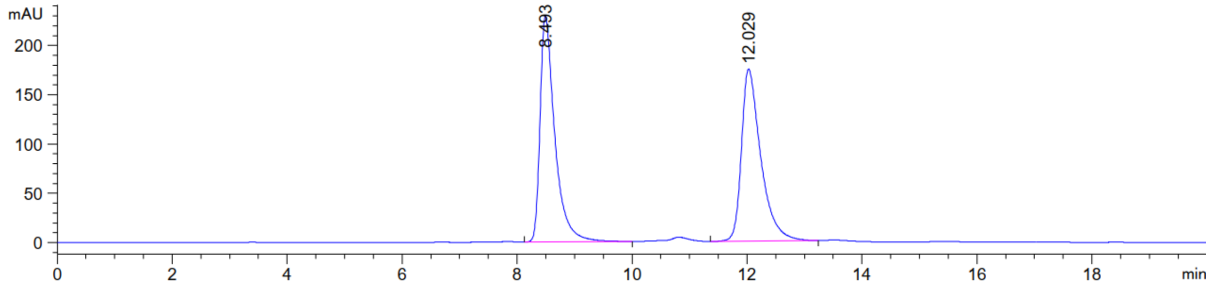

Peak #	RetTime [min]	Type	Width [min]	Area [mAU*s]	Height [mAU]	Area %
1	8.493	BB	0.2623	4096.02344	229.42836	50.0236
2	12.029	BB	0.3470	4092.16650	174.89574	49.9764

(*R*,*E*)-3-(2-(4-(trifluoromethyl)phenyl)prop-1-en-1-yl)isoindolin-1-one (3ae)

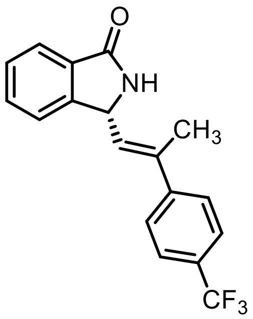

71% yield (20.50 mg, 0.071 mmol, *E/Z* > 20:1), white colour solid. **m.p. =** 164-166 ̊C. **R_f** = 0.26 (pentane/ethylacetate = 1/1). **^{1}H NMR** (600 MHz, Methanol-d_4) δ 7.80 (dt, *J* = 7.7, 1.0 Hz, 1H), 7.65-7.59 (m, 5H), 7.54 – 7.48 (m, 2H), 5.68 (d, *J* = 9.3 Hz, 1H), 5.58 (dq, *J* = 9.3, 1.4 Hz, 1H), 2.42 (d, *J* = 1.4 Hz, 3H). **^{13}C NMR** (151 MHz, Methanol-d4) δ 173.0, 148.7, 147.7, 140.3, 133.6, 132.9, 130.5 (q, *J* = 32.3 Hz), 129.6, 127.8, 127.5, 126.3 (q, *J* = 3.8 Hz), 125.7 (q, *J* = 271.1 Hz), 124.5, 124.4, 56.7, 16.6. **^{19}F NMR** (376 MHz, Methanol-d_4): -64.0. **HRMS** (ESI+/QTOF): *m/z* calcd. for $C_{18}H_{15}F_3NO^+$ [M + H]$^+$: 318.1100; found: 318.1104. **IR** (ATR): 3272, 2925, 1706, 1668, 1327, 1167, 1115, 835, 752 cm^{-1}. **[α]$_D$ ^{20}** = +13.7 (c = 0.5, $CHCl_3$). **Chiral HPLC:** Chiralpak IB, 4.6 x 250 mm; 15% i-PrOH/hexane, 1.0 mL/min, 254 nm; t_R (minor) = 7.47 min, t_R (major) = 10.93 min, 96:4 *er*

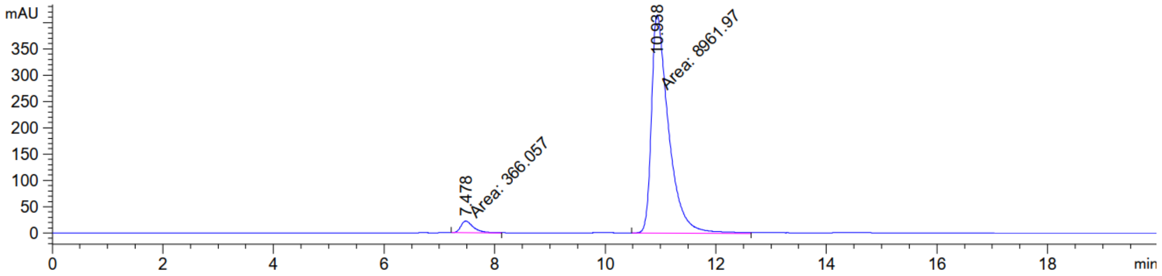

Peak #	RetTime [min]	Type	Width [min]	Area [mAU*s]	Height [mAU]	Area %
1	7.478	MM	0.2732	366.05746	22.33053	3.9243
2	10.938	MM	0.3606	8961.97070	414.21674	96.0757

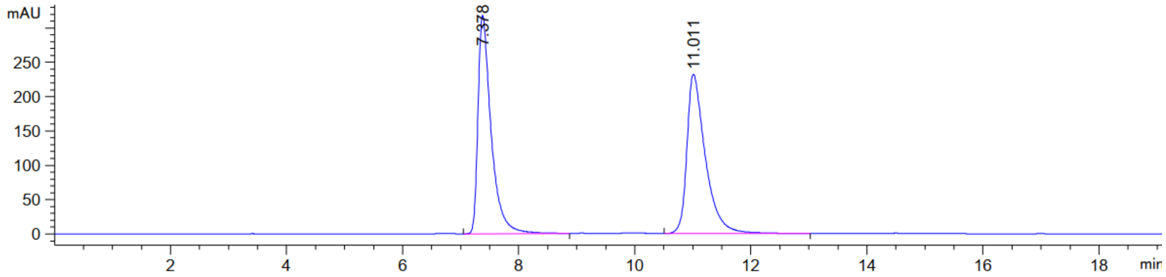

Peak #	RetTime [min]	Type	Width [min]	Area [mAU*s]	Height [mAU]	Area %
1	7.378	BB	0.2334	4995.71338	317.85464	49.3398
2	11.011	BB	0.3236	5129.41016	232.30771	50.6602

(*R*,*E*)-3-(2-([1,1'-biphenyl]-4-yl)prop-1-en-1-yl)isoindolin-1-one (3af)

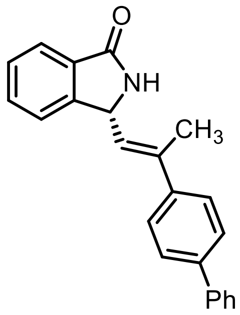

59% yield (19.20 mg, 0.059 mmol, *E/Z* > 20:1), white colour solid. **m.p. =** 235-238 ˚C. **R$_f$** = 0.27 (pentane/ethylacetate =1/1). **^{1}H NMR** (400 MHz, $CDCl_3$) δ 7.89 (dt, *J* = 7.5, 1.0 Hz, 1H), 7.60-7.55 (m, 5H), 7.52 – 7.40 (m, 6H), 7.37 – 7.32 (m, 1H), 6.71 (s, 1H), 5.64 (dd, *J* = 9.3, 1.3 Hz, 1H), 5.60 (d, *J* = 9.3 Hz, 1H), 2.41 (d, *J* = 1.3 Hz, 3H). **^{13}C NMR** (101 MHz, $CDCl_3$) δ 171.0, 147.2, 141.0, 140.7, 140.6, 139.3, 132.3, 131.7, 128.9, 128.6, 127.5, 127.2, 127.1, 126.4, 124.9, 124.0, 123.3, 55.5, 16.7. **HRMS** (ESI+/QTOF): *m/z* calcd. for $C_{23}H_{19}NNaO^+$ $[M + Na]^+$: 348.1359; found: 348.1344. **IR** (ATR): 3213, 3074, 3030, 1694, 1487, 1468, 833, 764, 750, 696 cm^{-1}. **[α]$_D$ ^{20}** = +59.8 (c = 1.0, $CHCl_3$). **Chiral HPLC:** Chiralpak IB, 4.6 x 250 mm; 15% i-PrOH/hexane, 1.0 mL/min, 280 nm; t_R (major) = 12.9 min, t_R (minor) = 18.8 min, 96.5:3.5 *er*

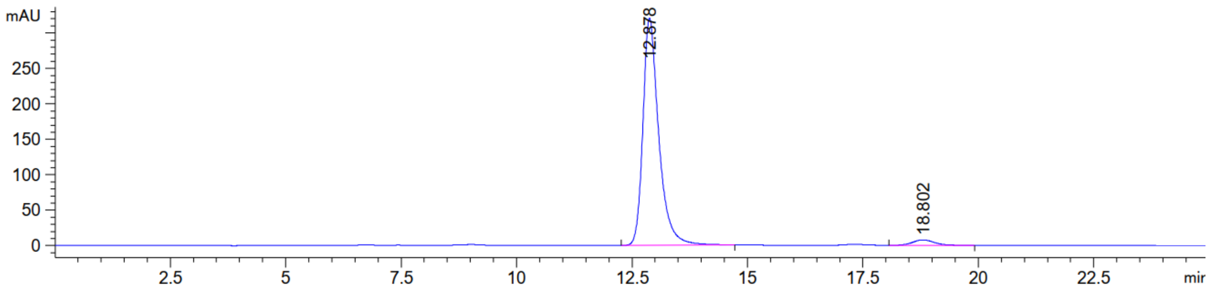

Peak #	RetTime [min]	Type	Width [min]	Area [mAU*s]	Height [mAU]	Area %
1	12.878	BB	0.3562	7644.44092	320.56281	96.6720
2	18.802	BB	0.4786	263.16263	7.81985	3.3280

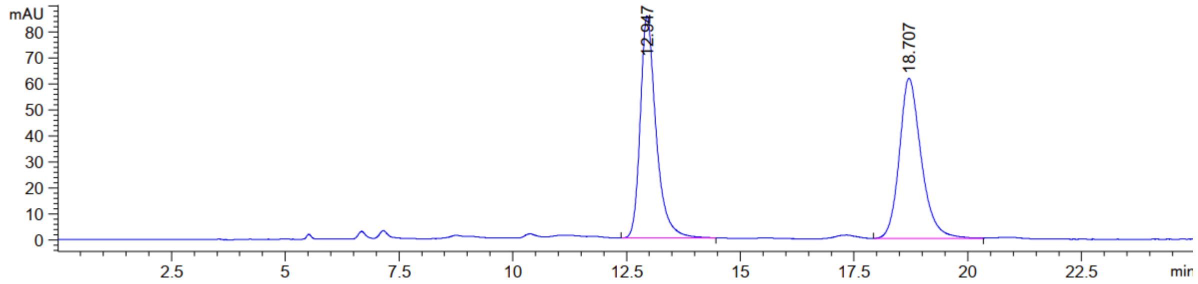

Peak #	RetTime [min]	Type	Width [min]	Area [mAU*s]	Height [mAU]	Area %
1	12.947	BB	0.3600	2067.04517	85.50692	50.0820
2	18.707	BB	0.4940	2060.27319	61.63074	49.9180

(*R*,*E*)-3-(2-(3-methoxyphenyl)prop-1-en-1-yl)isoindolin-1-one (3ag)

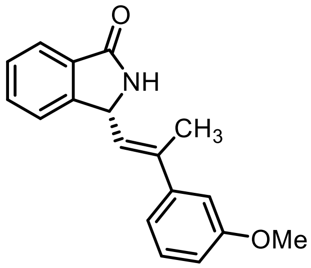

63% yield (17.50 mg, 0.063 mmol, *E/Z* > 20:1), white colour solid. **R$_f$** = 0.30 (pentane/ethylacetate = 1/1). **^{1}H NMR** (400 MHz, Methanol-d$_4$) δ 7.79 (dt, *J* = 7.6, 1.0 Hz, 1H), 7.63 (td, *J* = 7.5, 1.2 Hz, 1H), 7.54 – 7.47 (m, 2H), 7.22 (t, *J* = 8.0 Hz, 1H), 6.99 (ddd, *J* = 7.7, 1.7, 0.9 Hz, 1H), 6.93 (m, 1H), 6.82 (ddd, *J* = 8.2, 2.6, 0.9 Hz, 1H), 5.65 (d, *J* = 9.3 Hz, 1H), 5.45 (dq, *J* = 9.3, 1.4 Hz, 1H), 3.77 (s, 3H), 2.37 (d, *J* = 1.4 Hz, 3H). **^{13}C NMR** (101 MHz, Methanol-d$_4$) δ 173.1, 161.2, 149.1, 145.4 141.6, 133.5, 132.9, 130.4, 129.5, 125.6, 124.5, 124.3, 119.4, 114.0, 112.7, 56.9, 55.6, 16.9. **HRMS** (ESI+/QTOF): *m/z* calcd. for $C_{18}H_{17}NNaO_2^+$ [M + Na]$^+$: 302.1151; found: 302.1143. **IR** (ATR): 3221, 3076, 2927, 1693, 1605, 1578, 1468, 1254, 750, 694 cm^{-1}. **[α]$_D$ ^{20}** = +16.7 (c = 0.5, CHCl$_3$). **Chiral HPLC:** Chiralpak IA, 4.6 x 250 mm; 15% i-PrOH/hexane, 1.0 mL/min, 280 nm; t$_R$ (minor) = 8.19 min, t$_R$ (major) = 9.08 min, 95:5 *er*

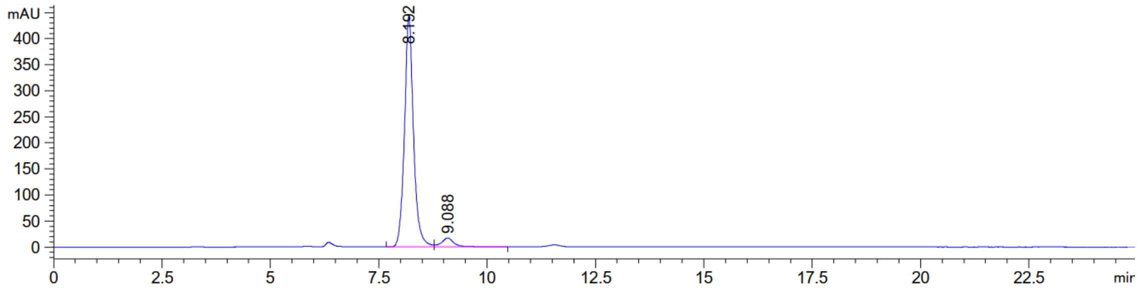

Peak #	RetTime [min]	Type	Width [min]	Area [mAU*s]	Height [mAU]	Area %
1	8.192	BV	0.2135	6429.19531	442.64264	94.7835
2	9.088	VB	0.2948	353.83884	17.28634	5.2165

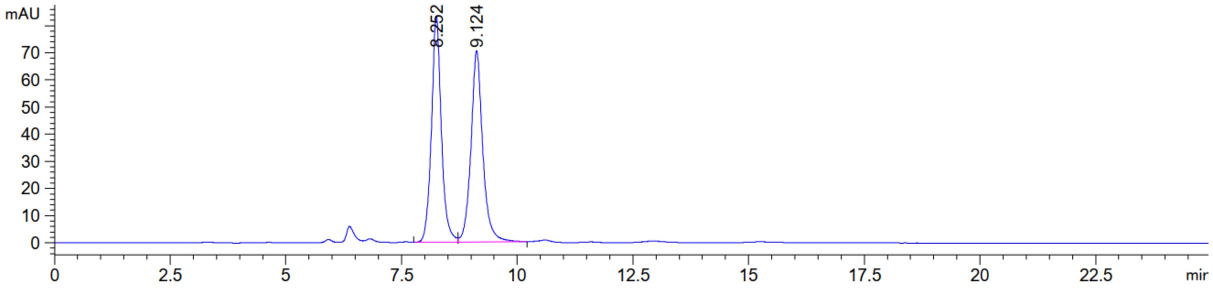

Peak #	RetTime [min]	Type	Width [min]	Area [mAU*s]	Height [mAU]	Area %
1	8.252	BV	0.2199	1243.24622	83.39200	49.7762
2	9.124	VB	0.2597	1254.42468	70.46989	50.2238

(*R*,*E*)-3-(2-(3-(trifluoromethyl)phenyl)prop-1-en-1-yl)isoindolin-1-one (3ah)

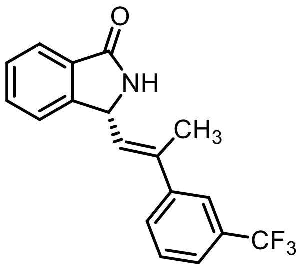

70% yield (22.10 mg, 0.070 mmol, *E/Z* = 10.6:1), white colour solid. **m.p. =** 110-113 ̊C. **R$_f$** = 0.50 (pentane/ethylacetate = 1/1). **^{1}H NMR** (600 MHz, Methanol-d$_4$) δ 7.80 (dt, *J* = 7.6, 1.0 Hz, 1H), 7.70 – 7.68 (m, 2H), 7.64 (td, *J* = 7.5, 1.1 Hz, 1H), 7.57 – 7.50 (m, 4H), 5.68 (d, *J* = 9.3 Hz, 1H), 5.54 (dd, *J* = 9.3, 1.4 Hz, 1H), 2.42 (d, *J* = 1.4 Hz, 3H). **^{13}C NMR** (151 MHz, Methanol-d$_4$) δ 173.1, 148.8, 144.9, 140.3, 133.6, 132.9, 131.8 (q, *J* = 32.0 Hz), 130.7, 130.3, 129.6, 127.5, 125.6 (q, *J* = 271.5 Hz), 125.2 (q, *J* = 3.9 Hz), 124.5, 124.4, 123.5 (q, *J* = 4.0 Hz), 56.8, 16.7. **^{19}F NMR** (565 MHz, Methanol-d$_4$): -64.2. **HRMS** (ESI+/QTOF): *m/z* calcd. for $C_{18}H_{14}F_3NNaO^+$ [M + Na]$^+$: 340.0920; found: 340.0916. **IR** (ATR): 3215, 3077, 2926, 1696, 1469, 1332, 1233, 1165, 1123, 1072, 801, 750, 698 cm^{-1}. **[α]$_D$^{20}** = -3.7 (c = 1.0, $CHCl_3$). **Chiral HPLC:** Chiralpak IB, 4.6 x 250 mm; 15% i-PrOH/hexane, 1.0 mL/min, 280 nm; t$_R$ (minor) = 6.53 min, t$_R$ (major) = 9.86 min, 94:6 *er*

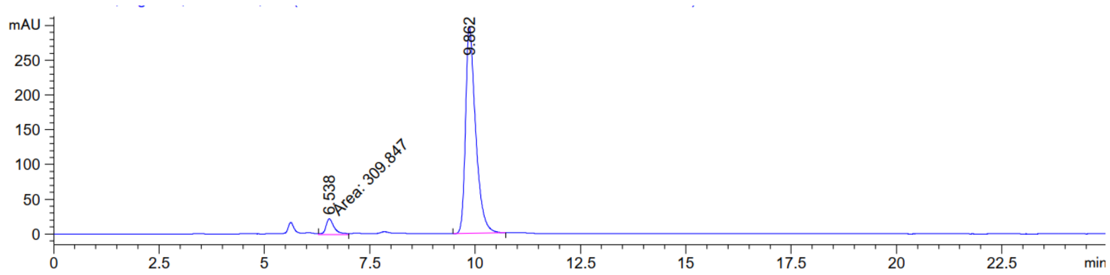

Peak #	RetTime [min]	Type	Width [min]	Area [mAU*s]	Height [mAU]	Area %
1	6.538	MM	0.2286	309.84711	22.59274	5.8757
2	9.862	BB	0.2464	4963.50293	297.93970	94.1243

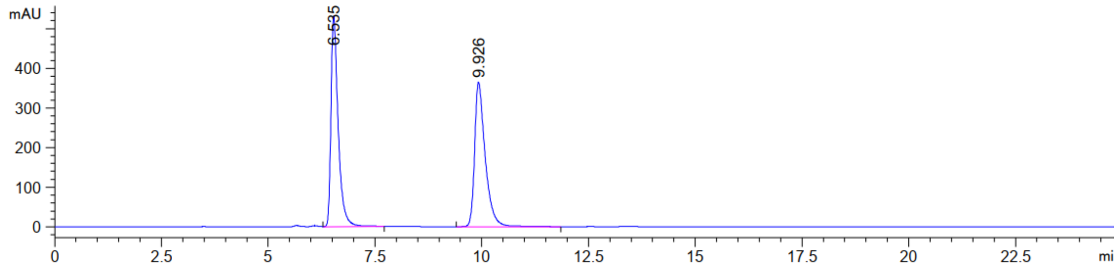

Peak #	RetTime [min]	Type	Width [min]	Area [mAU*s]	Height [mAU]	Area %
1	6.535	VB	0.1751	6239.53516	531.09003	49.8657
2	9.926	BB	0.2526	6273.15234	364.90103	50.1343

(*R*,*E*/*Z*)-3-(2-(2-chlorophenyl)prop-1-en-1-yl)isoindolin-1-one (3ai)

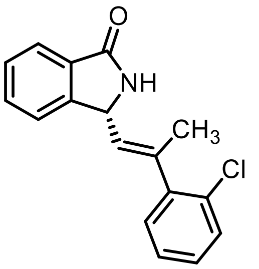

78% yield (22.13 mg, 0.078 mmol, *E*/*Z* > 1:0.85 (inseparable)), white colour solid. **R$_f$** = 0.3 (pentane/ethylacetate = 1/1). **^{1}H NMR** (400 MHz, Methanol-d$_4$) δ 7.78 (d, *J* = 7.6 Hz, 1H, *E*), 7.72 (d, *J* = 7.6 Hz, 0.85H, *Z*), 7.65 (td, *J* = 7.5, 1.1 Hz, 1H, *E*), 7.61 – 7.30 (m, 9H), 7.27-7.20 (m, 3H), 5.62 (d, *J* = 9.3 Hz, 1H, *E*), 5.26 (d, *J* = 9.5 Hz, 0.85H, *Z*), 5.07 (dq, *J* = 9.4, 1.6 Hz, 1H, *E*), 4.75-4.68 (m, 1H, *Z*), 2.31 (d, *J* = 1.4 Hz, 3H, *E*), 2.08 (d, *J* = 1.6 Hz, 2.55H, *Z*).**^{13}C NMR** (101 MHz, Methanol-d$_4$, for both *E* and *Z* isomers (1:0.8 *E*/*Z*)) δ 173.1, 172.8, 148.7, 148.5, 144.3, 142.8, 141.5, 140.8, 133.5, 133.3, 132.9, 132.8, 132.7, 131.7,131.3, 130.9, 130.8, 130.6, 130.4, 129.8, 129.5, 129.5, 128.8, 128.6, 128.1, 126.8, 124.7, 124.6, 124.3, 124.2, 57.7, 56.5, 24.4, 18.4. **HRMS** (ESI+/QTOF): *m/z* calcd. for $C_{18}H_{14}ClNNaO^+$ $[M + Na]^+$: 306.0656; found: 306.0646. **IR** (ATR): 3211, 3069, 1690, 1469, 1429, 1358, 1038, 747, 693 cm^{-1}. **[α]$_D$ ^{20}** = -36.6 (c = 1.5, $CHCl_3$). **Chiral HPLC:** Chiralpak IA, 4.6 x 250 mm; 5% i-PrOH/hexane, 1.0 mL/min, 254 nm; for (*E*-isomer) t_R (major) = 11.73 min, t_R (minor) = 15.43 min, 94:6 *er*; for (*Z*-isomer) t_R (major) = 13.63 min, t_R (minor) = 23.41 min, 90.5:9.5 *er*.

Chiral HPLC for *(E*-**3ai**)

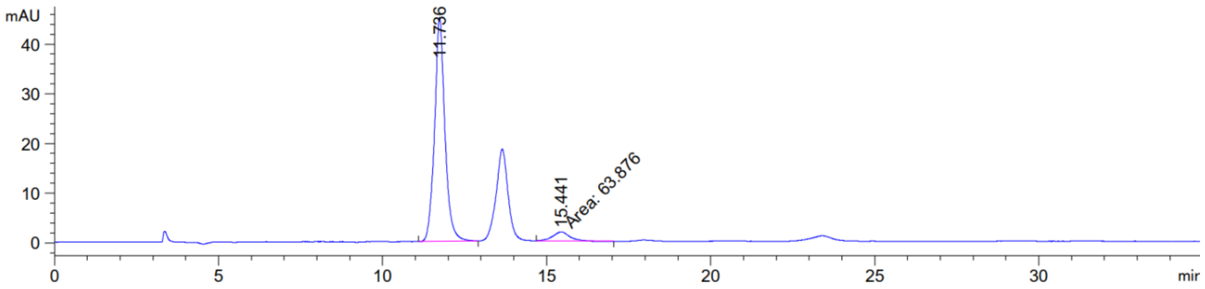

Peak #	RetTime [min]	Type	Width [min]	Area [mAU*s]	Height [mAU]	Area %
1	11.736	BB	0.3217	995.37299	45.07125	93.9697
2	15.441	MM	0.5856	63.87598	1.81812	6.0303

Chiral HPLC for (*Z*-**3ai**)

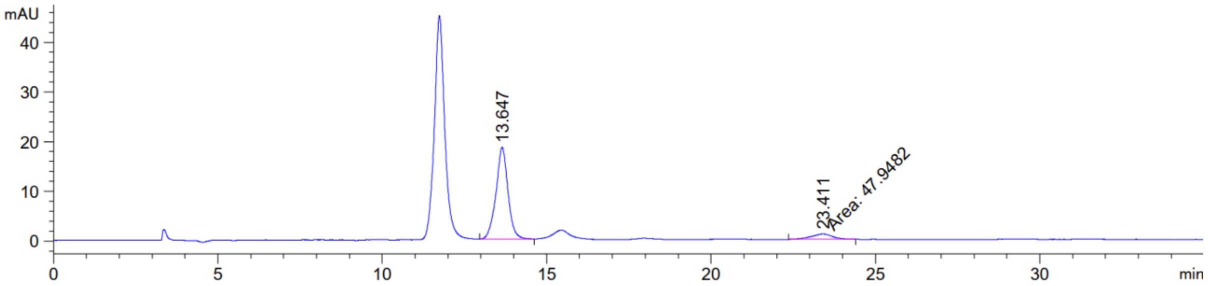

Peak #	RetTime [min]	Type	Width [min]	Area [mAU*s]	Height [mAU]	Area %
1	13.647	BB	0.3717	468.01758	18.46753	90.7071
2	23.411	MM	0.7299	47.94823	1.09490	9.2929

Chiral HPLC for (*rac*-**3ai**)

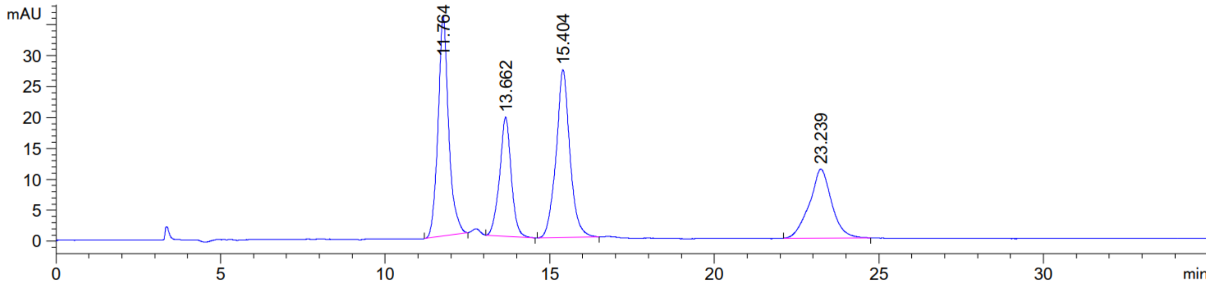

Peak #	RetTime [min]	Type	Width [min]	Area [mAU*s]	Height [mAU]	Area %
1	11.764	BB	0.3266	795.54529	35.61783	30.7824
2	13.662	BB	0.3630	473.72247	19.25484	18.3300
3	15.404	BB	0.4230	793.23578	27.13809	30.6931
4	23.239	BB	0.6482	521.91089	11.14796	20.1946

(*R*,*E*)-3-(2-(naphthalen-2-yl)prop-1-en-1-yl)isoindolin-1-one (3aj)

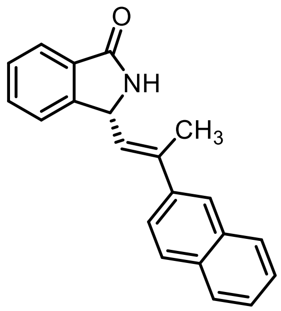

66% yield (19.70 mg, 0.066 mmol, *E/Z* > 20:1), white colour solid. **m.p.** = 217-219 °C. $\mathbf{R_f}$ = 0.35 (pentane/ethylacetate = 1/1). **^{1}H NMR** (400 MHz, $CDCl_3$): δ 7.89 (dt, *J* = 7.5, 1.0 Hz, 1H), 7.836 – 7.77 (m, 4H), 7.60 – 7.42 (m, 6H), 6.76 (s, 1H), 5.73 (dq, *J* = 9.3, 1.4 Hz, 1H), 5.63 (d, *J* = 9.3 Hz, 1H), 2.48 (d, *J* = 1.3 Hz, 3H). **^{13}C NMR** (400 MHz, $CDCl_3$): δ 171.0, 147.2, 139.6, 139.3, 133.4, 133.0, 132.3, 131.7, 128.6, 128.3, 128.1, 127.7, 126.5, 126.2, 125.4, 124.9, 124.1, 124.0, 123.4, 55.6, 16.8. **HRMS** (ESI+/QTOF): *m/z* calcd. for $C_{21}H_{17}NNaO^+$ $[M + Na]^+$: 322.1202; found: 322.1200. **IR** (ATR): 3211, 3056, 1693, 1468, 817, 749, 693 cm^{-1}. $[\alpha]_D^{20}$ = +19.3 (c = 0.7, $CHCl_3$). **Chiral HPLC:** Chiralpak IG, 4.6 x 250 mm; 30% i-PrOH/hexane, 1.0 mL/min, 254 nm; t_R (minor) = 12.87 min, t_R (major) = 17.03 min, 94.5:5.5 *er*.

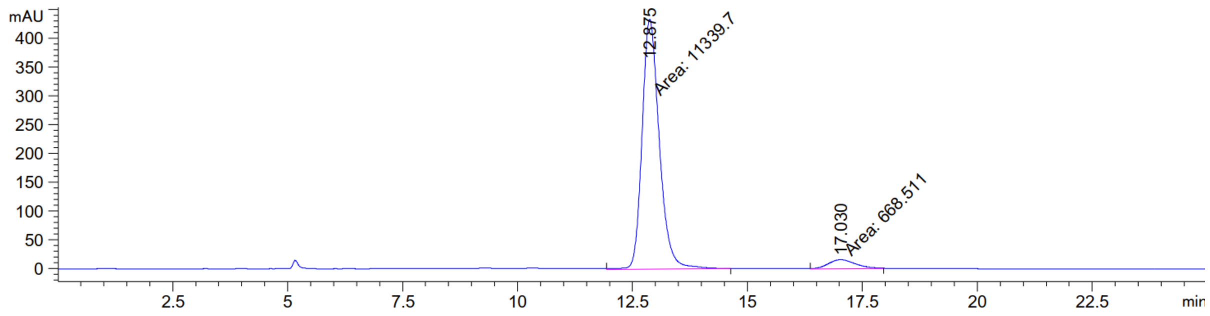

Peak #	RetTime [min]	Type	Width [min]	Area [mAU*s]	Height [mAU]	Area %
1	12.875	MM	0.4358	1.13397e4	433.71289	94.4329
2	17.030	MM	0.7035	668.51099	15.83847	5.5671

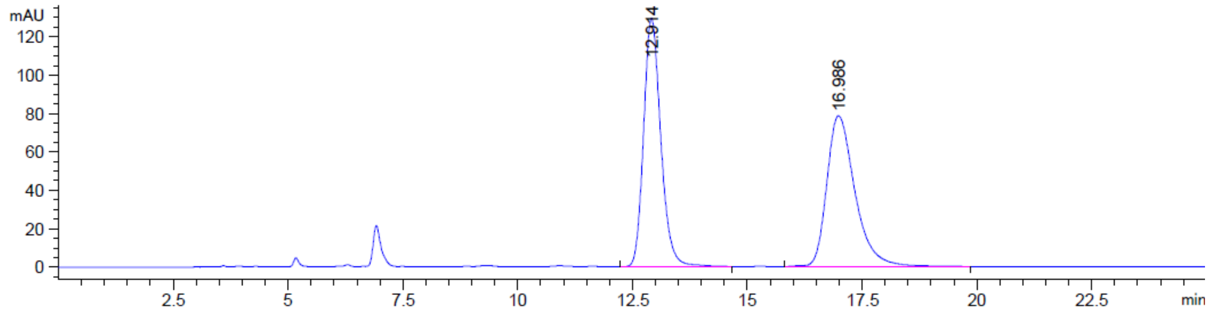

Peak #	RetTime [min]	Type	Width [min]	Area [mAU*s]	Height [mAU]	Area %
1	12.914	BB	0.4001	3372.21729	129.37572	50.1521
2	16.986	BB	0.6502	3351.76831	78.48563	49.8479

***(R,E)*-3-(2-(thiophen-2-yl)prop-1-en-1-yl)isoindolin-1-one (3ak)**

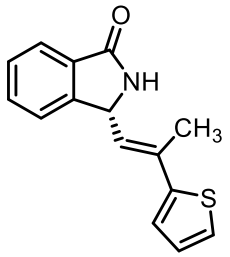

33% yield (8.4 mg, 0.033 mmol, *E/Z* > 20:1), brown colour sticky material. **R_f** = 0.50 (Pentane/ethylacetate =1/1). **^{1}H NMR** (400 MHz, Methanol-d_4): δ 7.80 (dt, *J* = 7.5, 1.0 Hz, 1H), 7.63 (td, *J* = 7.5, 1.2 Hz, 1H), 7.52 (t, *J* = 7.4 Hz, 1H), 7.46 (dd, *J* = 7.6, 1.0 Hz, 1H), 7.25 (dd, *J* = 5.1, 1.1 Hz, 1H), 7.16 (dd, *J* = 3.7, 1.2 Hz, 1H), 6.99 (dd, *J* = 5.1, 3.7 Hz, 1H), 5.65 (d, *J* = 9.3 Hz, 1H), 5.57 (dq, *J* = 9.4, 1.4 Hz, 1H), 2.40 (d, *J* = 1.3 Hz, 3H). **^{13}C NMR** (151 MHz, Methanol-d_4) δ 173.0, 148.9, 147.23 135.1, 133.6, 132.9, 129.6, 128.5, 125.5, 125.0, 124.6, 124.3, 123.5, 56.6, 16.6. **HRMS** (ESI+/QTOF): *m/z* calcd. for $C_{15}H_{13}NNaOS^+$ [M + Na]$^+$: 278.0610; found: 278.0609. **IR** (ATR): 3229, 1692, 1468, 1237, 751, 695 cm^{-1}. **$[\alpha]_D$ ^{20}** = +6.1 (c = 0.3, $CHCl_3$). **Chiral HPLC:** Chiralpak IB, 4.6 x 250 mm; 15% i-PrOH/hexane, 1.0 mL/min, 280 nm; t_R (minor) = 9.79 min, t_R (major) = 15.34 min, 93:7 *er*

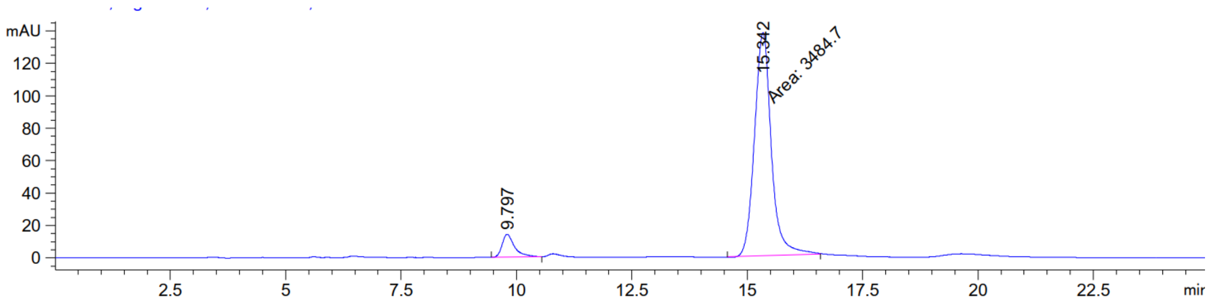

Peak #	RetTime [min]	Type	Width [min]	Area [mAU*s]	Height [mAU]	Area %
1	9.797	BB	0.2810	265.47571	14.13994	7.0790
2	15.342	MM	0.4220	3484.69653	137.61166	92.9210

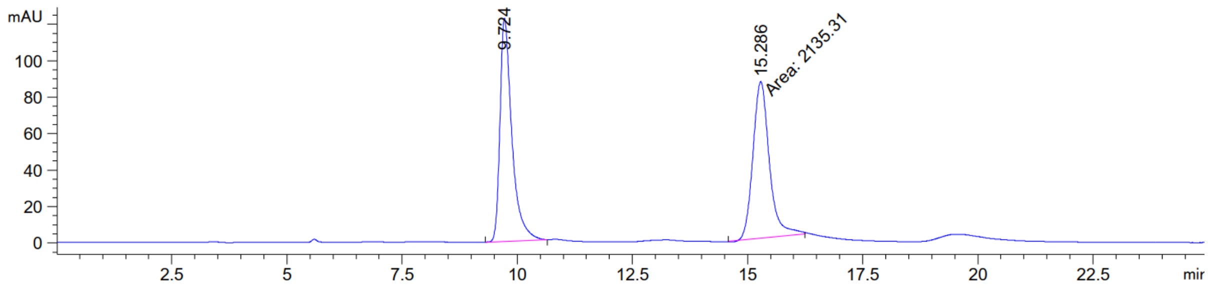

Peak #	RetTime [min]	Type	Width [min]	Area [mAU*s]	Height [mAU]	Area %
1	9.724	BB	0.2650	2193.42676	122.42822	50.6713
2	15.286	MM	0.4141	2135.30518	85.94790	49.3287

(*R*,*E*/*Z*)-3-(2-methyl-4-phenylbut-1-en-1-yl)isoindolin-1-one (**3al**)

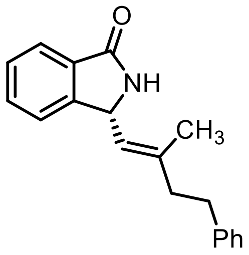

71% yield (19.7 mg, 0.071 mmol, *E*/*Z* = 1:0.55 (separable)) **(*E*)-isomer:** White colour solid, $\mathbf{R_f}$ = 0.39 (pentane/ethylacetate = 1:1). **^{1}H NMR** (400 MHz, Methanol-d$_4$): δ 7.72 (d, *J* = 7.6 Hz, 1H), 7.54 (td, *J* = 7.5, 1.2 Hz, 1H), 7.46 (t, *J* = 7.5 Hz, 1H), 7.2 – 7.20 (m, 2H), 7.18 – 7.12 (m, 4H), 5.39 (d, *J* = 9.3 Hz, 1H), 4.83 (dq, *J* = 9.3, 1.3 Hz, 1H), 2.83-2.71 (m, 2H), 2.40 (t, *J* = 7.6 Hz, 2H), 1.99 (d, *J* = 1.3 Hz, 3H). **^{13}C NMR** (101 MHz, Methanol-d$_4$) δ 173.0, 149.4, 142.8, 142.0, 133.3, 132.7, 129.5, 129.3, 129.2, 126.9, 124.5, 124.0, 123.7, 56.5, 42.5, 35.1, 16.8. **HRMS** (ESI+/QTOF): *m/z* calcd. for $C_{19}H_{19}NNaO^+$ [M + Na]$^+$: 300.1359; found: 300.1354. **IR** (ATR):3241, 3026, 2926, 1693, 1469, 1454, 748, 698 cm^{-1}. **[α]$_D$ ^{20}** = -21.5 (c = 1.0, $CHCl_3$). **Chiral HPLC:** Chiralpak IB, 4.6 x 250 mm; 15% i-PrOH/hexane, 1.0 mL/min, 254 nm; t_R (minor) = 7.18 min, t_R (major) = 7.63 min, 98:2 *er*.

(*Z*)-isomer: Colourless sticky material, $\mathbf{R_f}$ = 0.66 (pentane/ethylacetate = 1/1). **^{1}H NMR** (400 MHz, Methanol-d$_4$): δ 7.70 (dt, *J* = 7.4, 1.0 Hz, 1H), 7.50 (td, *J* = 7.5, 1.3 Hz, 1H), 7.44 (ddd, *J* = 8.0, 7.3, 1.1 Hz, 1H), 7.34 – 7.27 (m, 4H), 7.26 – 7.21 (m, 1H), 6.91 (dd, *J* = 7.5, 1.0 Hz, 1H), 5.21 (d, *J* = 9.7 Hz, 1H), 4.83 (d, *J* = 9.7 Hz, 1H), 2.94-2.87 (m, 2H), 2.75 (dt, *J* = 13.2, 7.6 Hz, 1H), 2.66 – 2.58 (m, 1H), 1.87 (d, *J* = 1.4 Hz, 3H). **^{13}C NMR** (101 MHz, Methanol-d$_4$) δ 172.8, 149.3, 143.0, 142.2, 133.2, 132.6, 129.8, 129.6, 129.2, 127.2, 124.6, 124.0, 123.9, 56.3, 35.31, 35.29, 23.6. **HRMS** (ESI+/QTOF): *m/z* calcd. for $C_{19}H_{20}NO^+$ [M + H]$^+$: 278.1539; found: 278.1547. **IR** (ATR): 2925, 1695, 1673, 1214, 759, 696, 541 cm^{-1}. **[α]$_D$ ^{20}** = -16.7 (c = 0.15, $CHCl_3$). **Chiral HPLC:** Chiralpak IB, 4.6 x 250 mm; 15% i-PrOH/hexane, 1.0 mL/min, 210 nm; t_R (minor) = 7.70 min, t_R (major) = 8.28 min, 87:13 *er*.

Chiral HPLC for (*E*-**3al**)

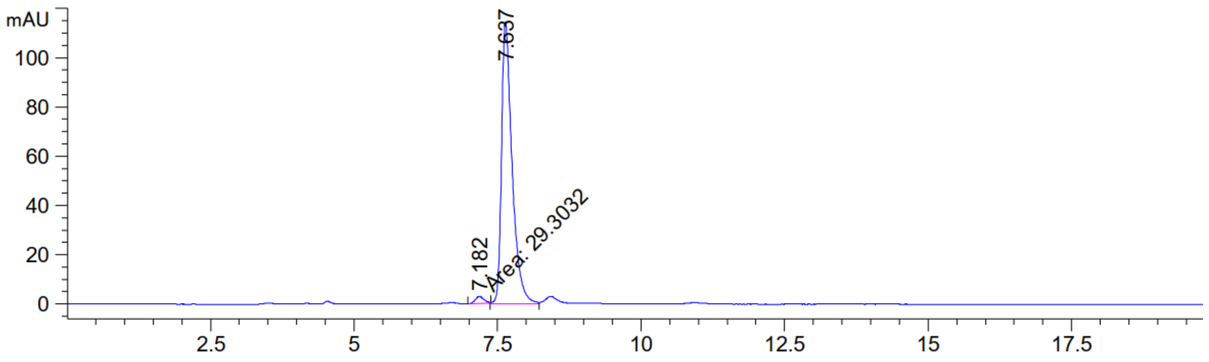

Peak #	RetTime [min]	Type	Width [min]	Area [mAU*s]	Height [mAU]	Area %
1	7.182	MM	0.1739	29.30320	2.80831	1.9726
2	7.637	VV	0.1885	1456.23938	114.38805	98.0274

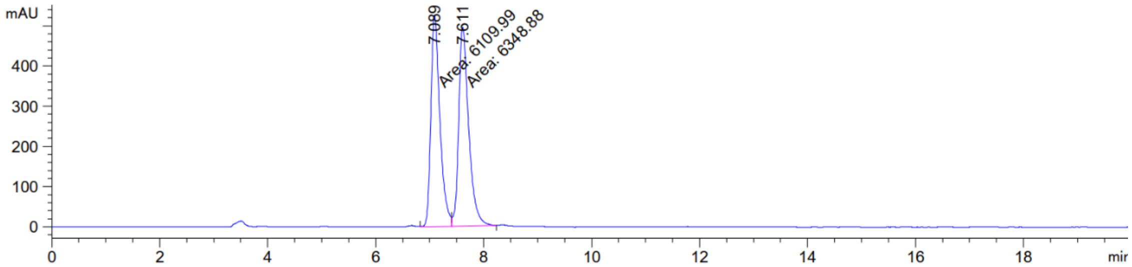

Peak #	RetTime [min]	Type	Width [min]	Area [mAU*s]	Height [mAU]	Area %
1	7.089	MF	0.1940	477.31296	41.00091	49.4765
2	7.611	FM	0.2113	487.41287	38.43727	50.5235

<u>Chiral HPLC for *(Z*-**3al**)</u>

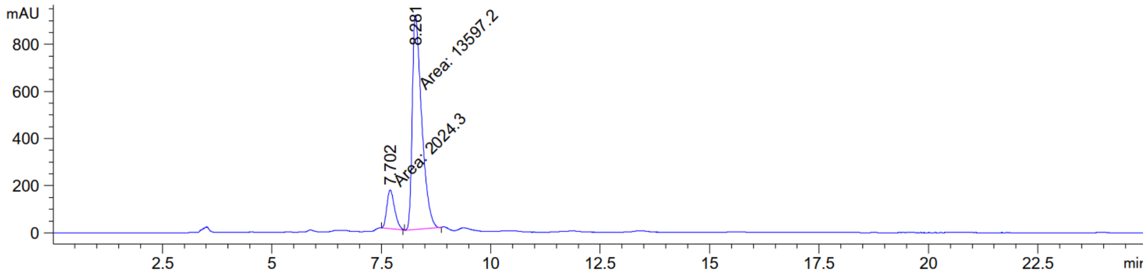

Peak #	RetTime [min]	Type	Width [min]	Area [mAU*s]	Height [mAU]	Area %
1	7.702	MM	0.2041	2024.30115	165.33768	12.9585
2	8.281	MM	0.2491	1.35972e4	909.91974	87.0415

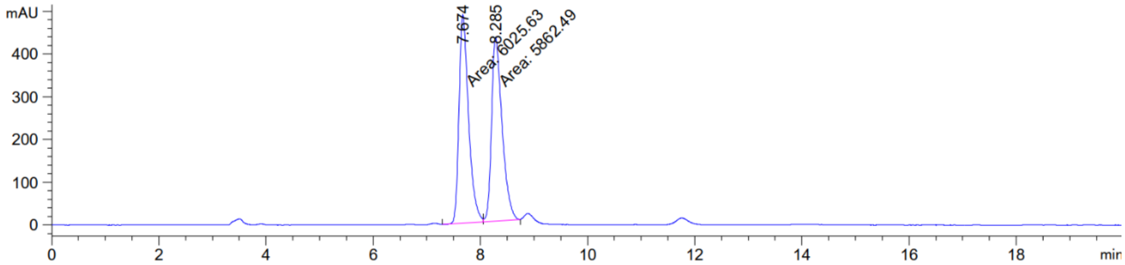

Peak #	RetTime [min]	Type	Width [min]	Area [mAU*s]	Height [mAU]	Area %
1	7.674	MF	0.2060	6025.63135	487.62210	50.6862
2	8.285	FM	0.2269	5862.48975	430.64728	49.3138

(*R*,*E*)-3-(2-phenylbut-1-en-1-yl)isoindolin-1-one (3am)

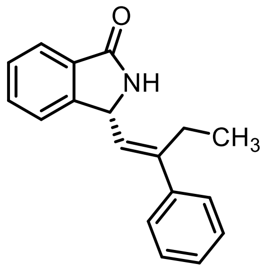

65% yield (17.0 mg, 0.065 mmol, *E*/*Z* = 15:1), white colour solid. **m.p. =** 202-204 ̊C. **R$_f$** = 0.35 (pentane/ethylacetate = 1:1). **^{1}H NMR** (400 MHz, $CDCl_3$) δ 7.87 (d, *J* = 7.5 Hz, 1H), 7.58 (td, *J* = 7.5, 1.2 Hz, 1H), 7.49 (t, *J* = 7.5 Hz, 1H), 7.41 (d, *J* = 7.7 Hz, 1H), 7.37 – 7.26 (m, 5H), 6.56 (s, 1H), 5.54 (d, *J* = 9.6 Hz, 1H), 5.37 (d, *J* = 9.5 Hz, 1H), 2.88 – 2.77 (m, 2H), 1.18 (t, *J* = 7.5 Hz, 3H). **^{13}C NMR** (101 MHz, $CDCl_3$) δ 170.9, 147.13, 147.12, 141.3, 132.3, 131.7, 128.5, 127.8, 126.6, 124.3, 124.0, 123.3, 55.2, 23.7, 14.8. **HRMS** (ESI+/QTOF): *m/z* calcd. for $C_{18}H_{18}NO^+$ $[M + H]^+$: 264.1383; found: 264.1386. **IR** (ATR): 3215, 2967, 2930, 4694, 1468, 1346, 750, 696 cm^{-1}. **$[\alpha]_D^{20}$** = +14.5 (c = 0.5, $CHCl_3$). **Chiral HPLC:** Chiralpak IB, 4.6 x 250 mm; 15% i-PrOH/hexane, 1.0 mL/min, 254 nm; t_R (minor) = 6.68 min, t_R (major) = 10.23 min, 95:5 *er*.

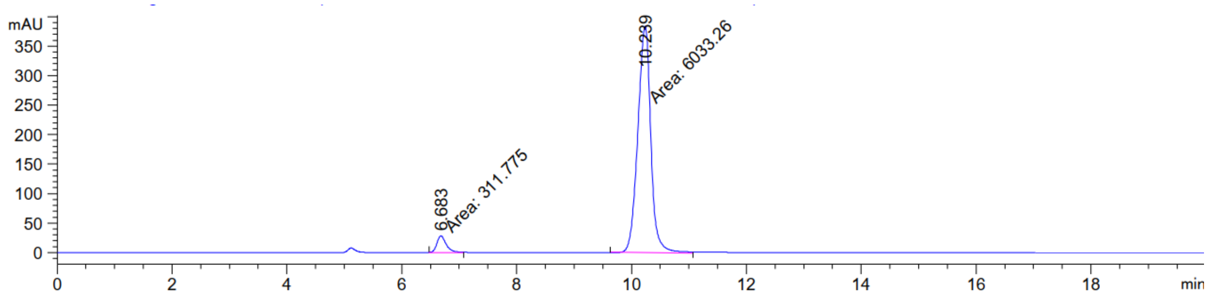

Peak #	RetTime [min]	Type	Width [min]	Area [mAU*s]	Height [mAU]	Area %
1	6.683	MM	0.1866	311.77545	27.84092	4.9137
2	10.239	MM	0.2620	6033.26123	383.80457	95.0863

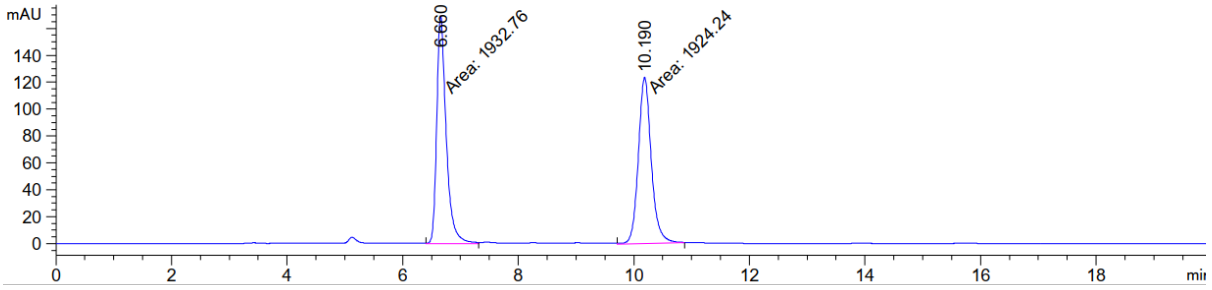

Peak #	RetTime [min]	Type	Width [min]	Area [mAU*s]	Height [mAU]	Area %
1	6.660	MM	0.1901	1932.76196	169.49504	50.1105
2	10.190	MM	0.2596	1924.23547	123.52203	49.8895

(*R*,*E*)-3-(2-phenylhex-1-en-1-yl)isoindolin-1-one (3an)

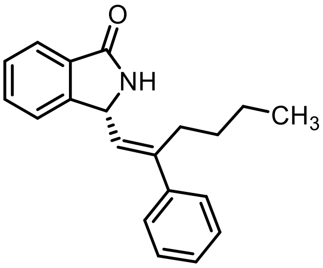

74% yield (21.50 mg, 0.074 mmol, *E/Z* = 9:1), white colour solid. **R_f** = 0.45 (pentane/ethylacetate = 1/1). **^{1}H NMR** (400 MHz, Methanol-d_4) δ 7.79 (dt, *J* = 7.8, 1.0 Hz, 1H), 7.64 (td, *J* = 7.5, 1.2 Hz, 1H), 7.54 – 7.47 (m, 2H), 7.38 – 7.23 (m, 5H), 5.62 (d, *J* = 9.6 Hz, 1H), 5.25 (d, *J* = 9.6 Hz, 1H), 2.91 – 2.79 (m, 2H), 1.53-1.31 (m, 4H), 0.93 (t, *J* = 7.1 Hz, 3H). **^{13}C NMR** (101 MHz, Methanol-d4) δ 173.1, 149.0, 147.3, 143.3, 133.5, 132.9, 129.5, 129.4, 128.6, 127.6, 125.7, 124.5, 124.3, 56.7, 32.7, 30.9, 23.7, 14.3. **HRMS** (ESI+/QTOF): *m/z* calcd. for $C_{20}H_{22}NO^+$ $[M + H]^+$: 292.1696; found: 292.1694. **IR** (ATR): 3210, 2955, 2928, 1694, 1467, 1347, 748, 697 cm^{-1}. **$[\alpha]_D$ ^{20}** = -3.4 (c = 1.0, $CHCl_3$). **Chiral HPLC:** Chiralpak IB, 4.6 x 250 mm; 5% i-PrOH/hexane, 1.0 mL/min, 254 nm; t_R (minor) = 8.97 min, t_R (major) = 14.7 min, 95:5 *er*.

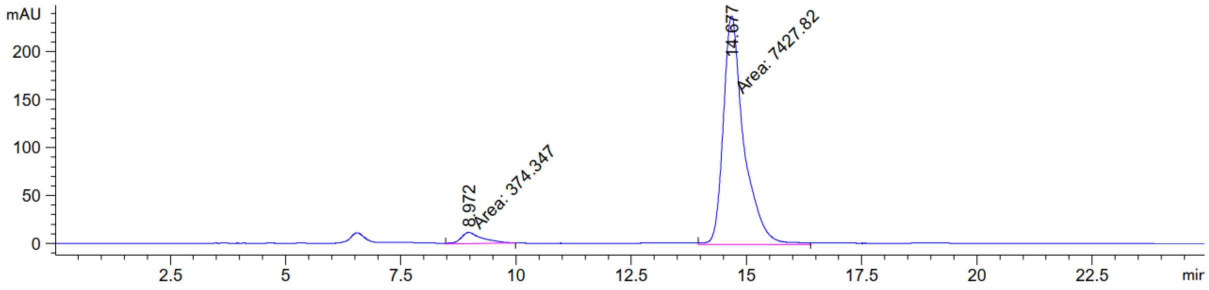

Peak #	RetTime [min]	Type	Width [min]	Area [mAU*s]	Height [mAU]	Area %
1	8.972	MM	0.5371	374.34747	11.61620	4.7980
2	14.677	MM	0.5195	7427.82324	238.29944	95.2020

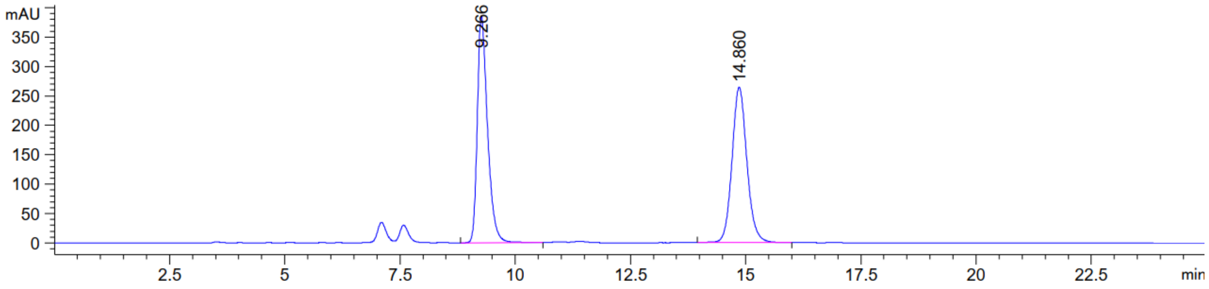

Peak #	RetTime [min]	Type	Width [min]	Area [mAU*s]	Height [mAU]	Area %
1	9.266	BB	0.2322	5953.69824	385.48764	49.9671
2	14.860	BB	0.3452	5961.53125	264.29572	50.0329

(*R*,*E*)-3-(2,3-diphenylprop-1-en-1-yl)isoindolin-1-one (3ao)

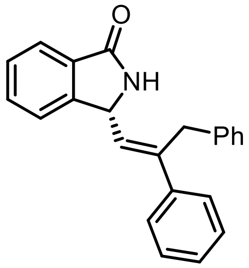

73% yield (23.40 mg, 0.073 mmol, *E/Z* = 16:1), pale yellow solid. **m.p.** = 97-99 ℃. **R$_f$** = 0.32 (pentane/ethylacetate = 1/1). **^{1}H NMR** (400 MHz, Methanol-d$_4$) δ 7.78 (dt, *J* = 7.5, 1.0 Hz, 1H), 7.55 (td, *J* = 7.5, 1.3 Hz, 1H), 7.48 (td, *J* = 7.5, 1.0 Hz, 1H), 7.37 – 7.33 (m, 2H), 7.28 – 7.13 (m, 9H), 5.64 (d, *J* = 9.6 Hz, 1H), 5.48 (d, *J* = 9.6 Hz, 1H), δ_A = 4.24, δ_B = 4.19 (AB, *J* = 16.0, 4.4 Hz 2H). **^{13}C NMR** (101 MHz, Methanol-d$_4$) δ 173.0, 148.7, 144.7, 143.0, 140.8, 133.4, 132.9, 129.6, 129.5, 129.4, 128.7, 127.8, 127.5, 127.3, 124.6, 124.3, 56.9, 36.8. **HRMS** (ESI+/QTOF): *m/z* calcd. for $C_{23}H_{19}NNaO^+$ [M + Na]$^+$: 348.1359; found: 348.1361. **IR** (ATR): 3210, 3060, 3026, 1693, 1493, 1468, 749, 696 cm^{-1}. **[α]$_D$ ^{20}** = -3.0 (c = 0.5, CHCl$_3$). **Chiral HPLC:** Chiralpak IB, 4.6 x 250 mm; 15% i-PrOH/hexane, 1.0 mL/min, 254 nm; t_R (minor) = 7.98 min, t_R (major) = 13.83 min, 95:5 *er*.

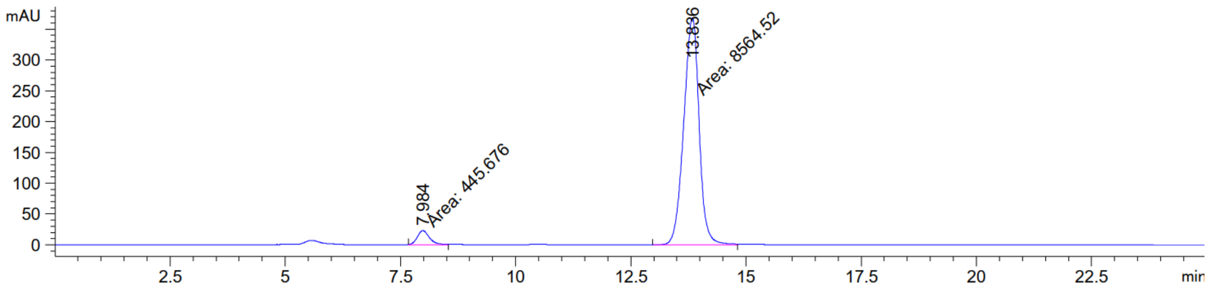

Peak #	RetTime [min]	Type	Width [min]	Area [mAU*s]	Height [mAU]	Area %
1	7.984	MM	0.3171	445.67587	23.42413	4.9464
2	13.836	MM	0.3872	8564.51758	368.67422	95.0536

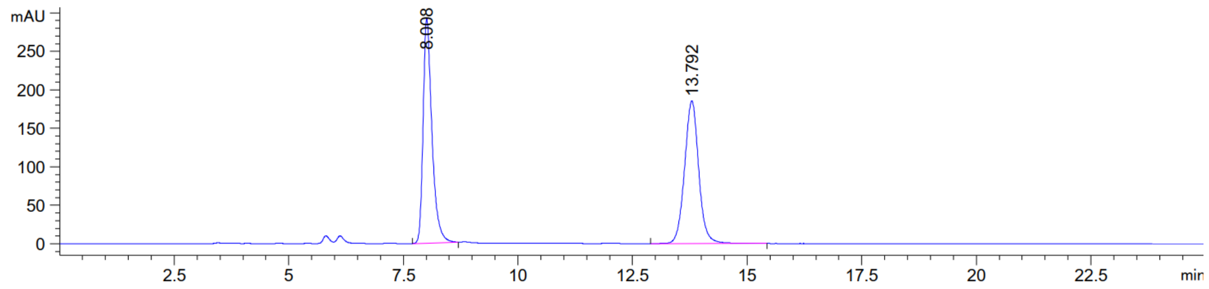

Peak #	RetTime [min]	Type	Width [min]	Area [mAU*s]	Height [mAU]	Area %
1	8.008	BB	0.2000	3907.53760	292.15463	49.5303
2	13.792	BB	0.3286	3981.64893	185.37520	50.4697

(*R*)-3-(3-methyl-2-phenylbut-1-en-1-yl)isoindolin-1-one (3ap)

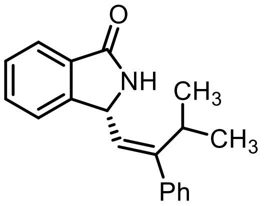

71% yield (19.68 mg, 0.071 mmol, *E/Z* = 1:2.6), White colour solid. **(*E*)-isomer: R$_f$** = 0.39 (pentane/ethylacetate = 1/1).**^{1}H NMR** (400 MHz, Methanol-d$_4$): δ 7.80 – 7.76 (m, 1H), 7.66 (td, *J* = 7.5, 1.2 Hz, 1H), 7.54-7.51 (m, 2H), 7.31 – 7.24 (m, 3H), 7.19-7.17 (m, 2H), 5.71 (d, *J* = 9.7 Hz, 1H), 4.88 (d, *J* = 9.9 Hz, 1H), 3.44 (hept, *J* = 6.9 Hz, 1H), 1.25 (d, *J* = 6.9 Hz, 3H), 1.17 (d, *J* = 6.9 Hz, 3H). **^{13}C NMR** (101 MHz, Methanol-d$_4$) δ 173.1, 153.7, 149.0, 142.9, 133.5, 130.0, 129.5, 129.5, 128.8, 128.0, 126.3, 124.5, 124.3, 55.9, 31.0, 23.0, 22.4. **HRMS** (ESI+/QTOF): *m/z* calcd. for $C_{19}H_{20}NO^+$ $[M + H]^+$: 278.1539; found: 278.1548. **IR** (ATR): 3209, 2963, 2927, 1695, 1468, 749, 702 cm^{-1}. **$[\alpha]_D$ ^{20}** = -17.9 (c = 0.5, $CHCl_3$). **Chiral HPLC:** Chiralpak IB, 4.6 x 250 mm; 15% i-PrOH/hexane, 1.0 mL/min, 254 nm; t_R (minor) = 5.59 min, t_R (major) = 6.64 min, 86.5:13.5 *er*

<u>Chiral HPLC for *(E*-**3ap**)</u>

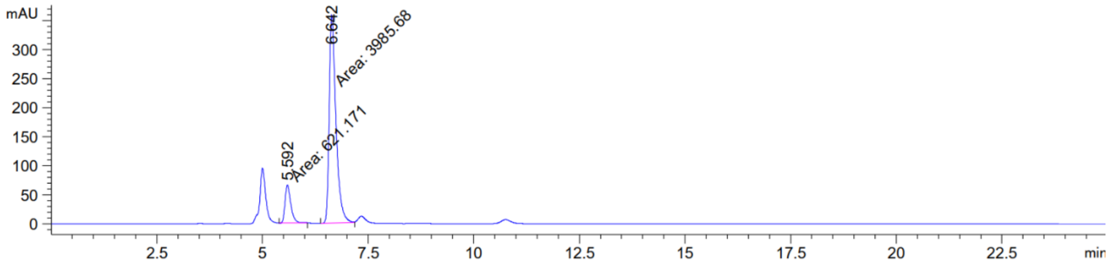

Peak #	RetTime [min]	Type	Width [min]	Area [mAU*s]	Height [mAU]	Area %
1	5.592	MM	0.1593	621.17072	64.97524	13.4836
2	6.642	MM	0.1853	3985.67529	358.42444	86.5164

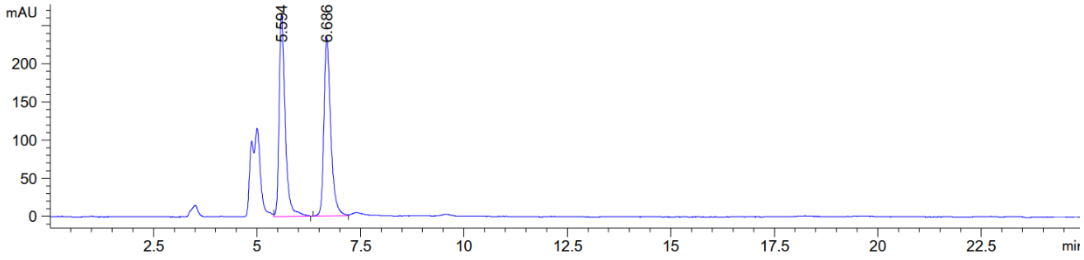

Peak #	RetTime [min]	Type	Width [min]	Area [mAU*s]	Height [mAU]	Area %
1	5.594	VB	0.1524	2689.59058	265.11081	50.1639
2	6.686	BV	0.1712	2672.01636	234.08310	49.8361

(Z)-isomer: White colour solid, **m.p.** = 118-121 ̊C. **R_f** = 0.42 (pentane/ethylacetate = 1/1). **^{1}H NMR** (400 MHz, Methanol-d$_4$): δ 7.73 (dt, *J* = 7.6, 0.9 Hz, 1H), 7.60 (td, *J* = 7.5, 1.1 Hz, 1H), 7.49 – 7.39 (m, 4H), 7.36 – 7.32 (m, 3H), 5.11 (dd, *J* = 9.7, 1.2 Hz, 1H), 4.88 (d, *J* = 9.7 Hz, 1H), 2.65 (m, 1H), 1.05 (d, *J* = 2.7 Hz, 3H), 1.03 (d, *J* = 2.6 Hz, 3H). **^{13}C NMR** (101 MHz, Methanol-d$_4$) δ 172.9, 155.1, 149.0, 140.8, 133.4, 132.8, 130.0, 129.4, 129.4, 128.4, 124.4, 124.2, 122.7, 57.5, 37.4, 21.9, 21.7. **HRMS** (ESI+/QTOF): *m/z* calcd. for $C_{19}H_{19}NNaO^+$ [M + Na]$^+$: 300.1359; found: 300.1362. **IR** (ATR): 3209, 2961, 2927, 2871, 1691, 1467, 750, 734, 704 cm^{-1}. $[\alpha]_D^{20}$ = -94.7 (c = 1.0, $CHCl_3$). **Chiral HPLC:** Chiralpak IG, 4.6 x 250 mm; 20% i-PrOH/hexane, 1.0 mL/min, 254 nm; t_R (major) = 8.4 min, t_R (minor) = 12.2 min, 85:15 *er*.

Chiral HPLC for (*Z*-**3ap**)

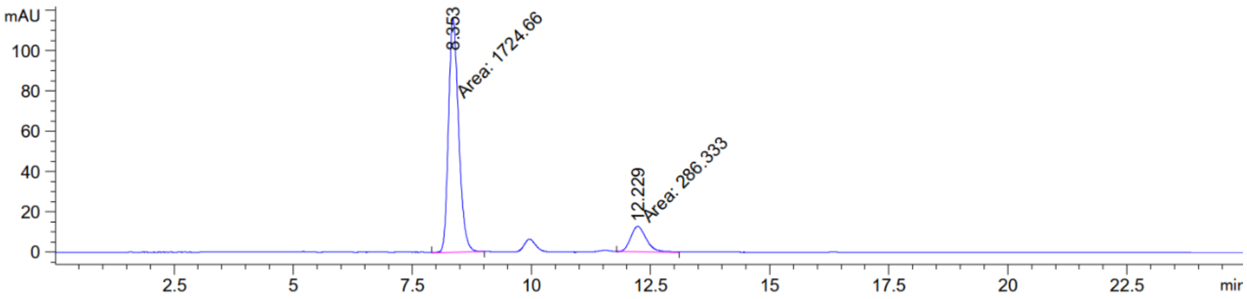

Peak #	RetTime [min]	Type	Width [min]	Area [mAU*s]	Height [mAU]	Area %
1	8.354	MM	0.2545	1.39415e4	913.15747	84.9219
2	12.231	MM	0.3806	2475.34229	108.39735	15.0781

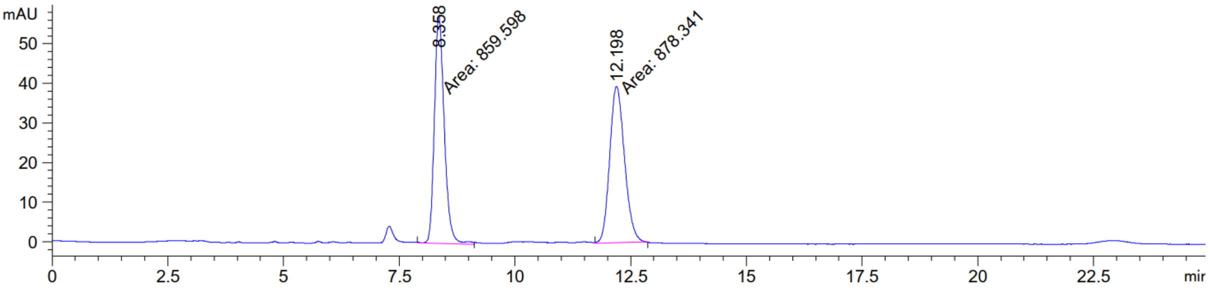

Peak #	RetTime [min]	Type	Width [min]	Area [mAU*s]	Height [mAU]	Area %
1	8.358	MM	0.2492	859.59821	57.50134	49.4608
2	12.198	MM	0.3708	878.34058	39.47744	50.5392

(*R*,*E*)-3-(3-methoxy-2-phenylprop-1-en-1-yl)isoindolin-1-one (3aq)

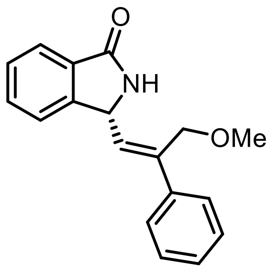

58% yield (16.20 mg, 0.058 mmol, *Z*/*E* = 17:1), white colour solid. **m.p. =** 166-168 ̊C. **R$_f$** = 0.3 (pentane/ethylacetate = 1/1). ^{1}**H NMR** (400 MHz, Methanol-d$_4$) δ 7.80 (d, *J* = 7.5 Hz, 1H), 7.64 (td, *J* = 7.5, 1.1 Hz, 1H), 7.58 – 7.50 (m, 2H), 7.44 – 7.42 (m, 2H), 7.34 – 7.24 (m, 3H), 5.74 (d, *J* = 9.6 Hz, 1H), 5.56 (d, *J* = 9.6 Hz, 1H), 4.67 (d, *J* = 11.5 Hz, 1H), 4.58 (d, *J* = 11.5 Hz, 1H), 3.49 (s, 3H). ^{13}**C NMR** (101 MHz, Methanol-d$_4$) δ 173.0, 148.5, 142.5, 141.9, 133.5, 132.9, 130.5, 129.6, 129.5, 128.8, 127.4, 125.0, 124.3, 70.9, 58.6, 56.5. **HRMS** (ESI+/QTOF): *m/z* calcd. for $C_{18}H_{17}NNaO_2^+$ [M + Na]$^+$: 302.1151; found: 302.1148. **IR** (ATR): 3238, 2927, 1692, 1468, 1191, 1094, 750, 695 cm^{-1}. **[α]$_D$^{20}** = +3.8 (c = 0.7, $CHCl_3$). **Chiral HPLC:** Chiralpak IB, 4.6 x 250 mm; 15% i-PrOH/hexane, 1.0 mL/min, 254 nm; t$_R$ (minor) = 8.20 min, t$_R$ (major) = 13.86 min, 93:7 *er*.

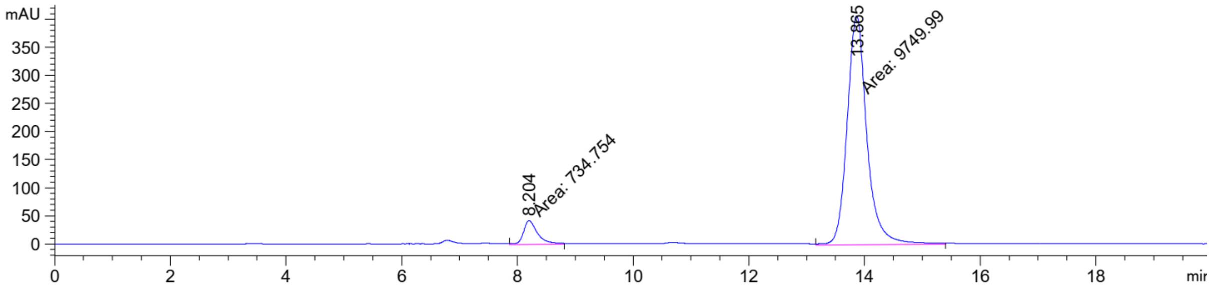

Peak #	RetTime [min]	Type	Width [min]	Area [mAU*s]	Height [mAU]	Area %
1	8.204	MM	0.2871	734.75397	42.65244	7.0078
2	13.865	MM	0.3985	9749.99121	407.80029	92.9922

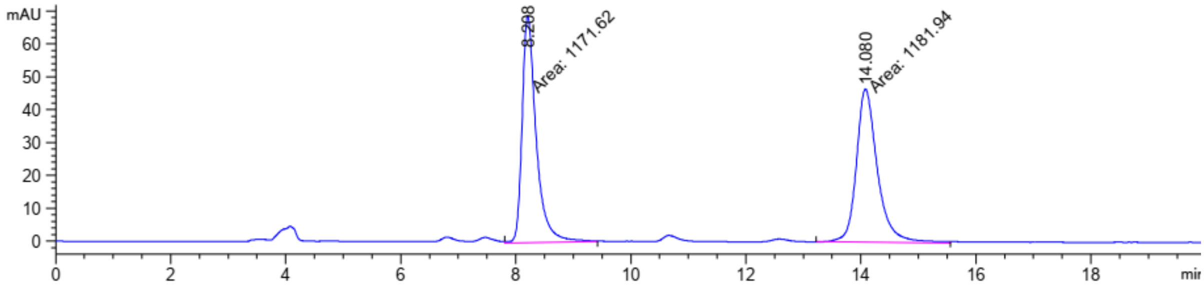

Peak #	RetTime [min]	Type	Width [min]	Area [mAU*s]	Height [mAU]	Area %
1	8.208	MM	0.2804	1159.63696	68.93148	49.8485
2	14.080	MM	0.4182	1166.68347	46.49996	50.1515

Cp^x vs Cp* Ligand Effect on the Reactivity and the Selectivity of the Annulation Process.

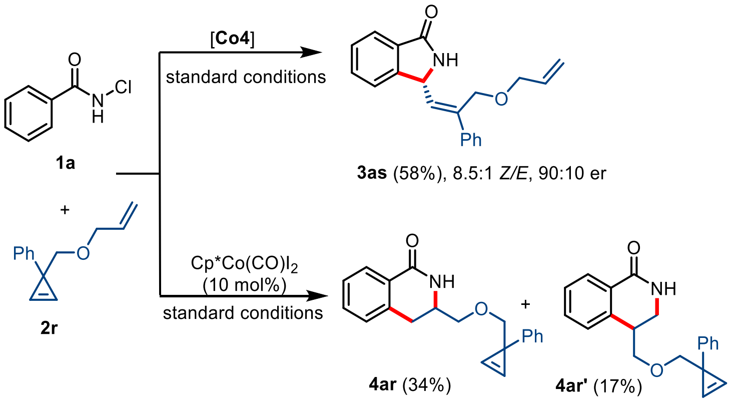

(*R*,*Z*)-3-(3-(allyloxy)-2-phenylprop-1-en-1-yl)isoindolin-1-one (3ar)

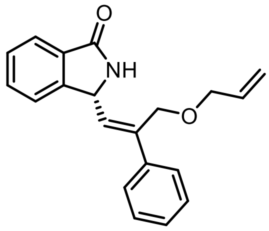

58% yield (17.70 mg, 0.058 mmol, *Z/E* = 8.5:1), white colour solid. **m.p. =** 135-137 ̊C. **R$_f$** = 0.29 (pentane/ethylacetate = 1/1). **^{1}H NMR** (400 MHz, Methanol-d$_4$) δ 7.79 (d, *J* = 7.5 Hz, 1H), 7.64 – 7.58 (m, 2H), 7.52 (td, *J* = 7.3, 1.4 Hz, 1H), 7.46 – 7.41 (m, 2H), 7.33 – 7.21 (m, 3H), 6.04-5.94 (m, 1H), 5.73 (d, *J* = 9.5 Hz, 1H), 5.55 (d, *J* = 9.5 Hz, 1H), 5.33 (dq, *J* = 17.3, 1.7 Hz, 1H), 5.21 (dq, *J* = 10.5, 1.5 Hz, 1H), 4.71 (d, *J* = 11.5 Hz, 1H), 4.61 (d, *J* = 11.5 Hz, 1H), 4.16 (dq, *J* = 5.8, 1.2 Hz, 2H). **^{13}C NMR** (101 MHz, Methanol-d$_4$) δ 173.0, 148.5, 142.5, 142.0, 135.9, 133.5, 132.9, 130.5, 129.6, 129.4, 128.8, 127.5, 125.1, 124.3, 117.9, 72.7, 68.5, 56.5. **HRMS** (ESI+/QTOF): *m/z* calcd. for $C_{20}H_{19}NNaO_2^+$ [M + Na]$^+$: 328.1308; found: 328.1298. **IR** (ATR): 3221, 3079, 2854, 1690, 1468, 1354, 1075, 750, 696 cm^{-1}. **[α]$_D$ ^{20}** = -8.9 (c = 1.0, $CHCl_3$). **Chiral HPLC:** Chiralpak IB, 4.6 x 250 mm; 15% i-PrOH/hexane, 1.0 mL/min, 254 nm; t$_R$ (minor) = 7.4 min, t$_R$ (major) = 12.2 min, 90:10 *er*.

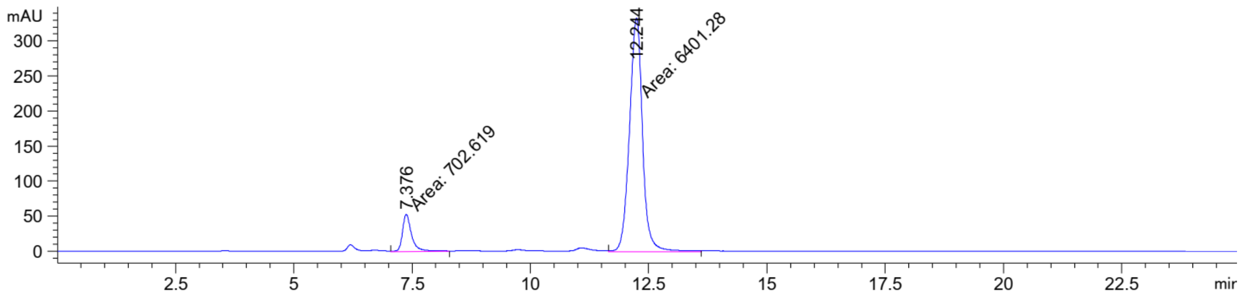

Peak #	RetTime [min]	Type	Width [min]	Area [mAU*s]	Height [mAU]	Area %
1	7.376	MM	0.2204	702.61890	53.13080	9.8906
2	12.244	MM	0.3199	6401.27783	333.46536	90.1094

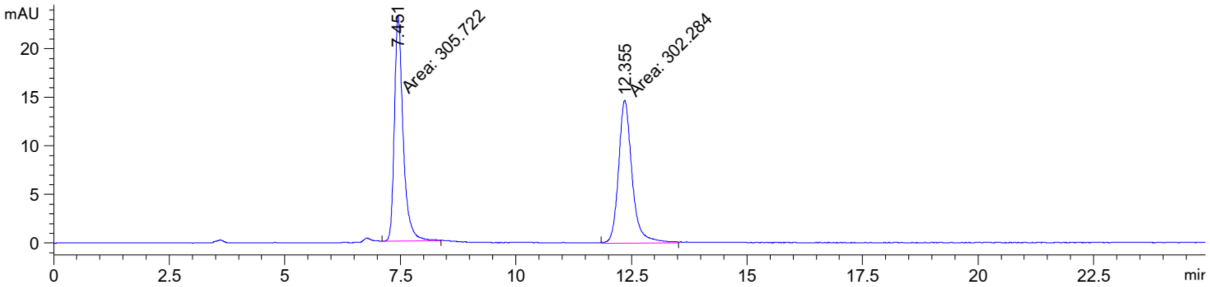

Peak #	RetTime [min]	Type	Width [min]	Area [mAU*s]	Height [mAU]	Area %
1	7.451	MM	0.2194	305.72232	23.22906	50.2828
2	12.355	MM	0.3421	302.28403	14.72539	49.7172

(±)-3-(((1-phenylcycloprop-2-en-1-yl)methoxy)methyl)-3,4-dihydroisoquinolin-1(2H)-one (4ar)

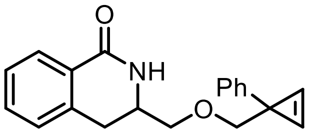

34% yield (10.2 mg, 0.034 mmol), white solid. **m.p. =** 101-103 ℃. **R$_f$** = 0.42 (pentane/ethylacetate =1/1). **^{1}H NMR** (400 MHz, CDCl$_3$) δ 8.07 (dd, *J* = 7.6, 1.4 Hz, 1H), 7.44 (td, *J* = 7.4, 1.5 Hz, 1H), 7.37 – 7.28 (m, 5H), 7.23-7.16 (m, 4H), 6.14 (s, 1H), 3.98– 3.90 (m, 3H), 3.62 (dd, *J* = 9.3, 4.4 Hz, 1H), 3.48 (*t*, *J* = 9.0 Hz, 1H), 2.91– 2.80 (m, 2H). **^{13}C NMR** (101 MHz, CDCl$_3$) δ 165.7, 146.2, 137.3, 132.4, 128.8, 128.3, 128.2, 127.6, 127.3, 126.4, 125.9, 112.9, 112.8, 78.7, 72. 9, 50.8, 30.7, 26.8. **HRMS** (APCI/QTOF): *m/z* calcd. for $C_{20}H_{19}NNaO_2^+$ [M + Na]$^+$: 328.1308; found: 328.1308. **IR** (ATR): 2857, 1669, 1605, 1465, 1112, 750, 699 cm^{-1}.

(±)4-(((1-phenylcycloprop-2-en-1-yl)methoxy)methyl)-3,4-dihydroisoquinolin-1(2H)-one (4ar')

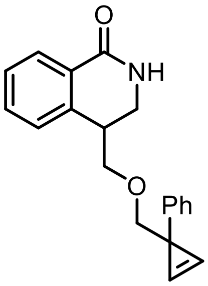

17% yield (5.1 mg, 0.017 mmol), colourless sticky material solid. **R$_f$** = 0.37 (pentane/ethylacetate =1/1). **^{1}H NMR** (400 MHz, CDCl$_3$) δ δ 8.07 (dd, *J* = 8.0, 1.4 Hz, 1H), 7.46 (td, *J* = 7.5, 1.4 Hz, 1H), 7.37 (td, *J* = 7.6, 1.0 Hz, 1H), 7.32 – 7.26 (m, 4H), 7.24 – 7.15 (m, 4H), 5.53 (s, 1H), 3.98 (d, *J* = 10.6 Hz, 1H), 3.87 (d, *J* = 10.6 Hz, 1H), 3.67 (t, *J* = 9.9 Hz, 1H), 3.60 – 3.47 (m, 3H), 3.16-3.10 (m, 1H). **^{13}C NMR** (101 MHz, CDCl$_3$) δ 165.7, 146.3, 139.2, 132.5, 128.8, 128.4, 128.2, 127.8, 127.7, 126.6, 125.8, 113.2, 113.1, 78.5, 70.4, 41.1, 38.7, 26.8.

HRMS (APCI/QTOF): *m/z* calcd. for $C_{20}H_{19}NNaO_2^+$ $[M + Na]^+$: 328.1308; found: 328.1304. **IR** (ATR): 2923, 2857, 1670, 1604, 1478, 1097, 1077, 761, 700 cm^{-1}.

Enantioselective Rh(III)-catalyzed [4+2] annulation of benzamides with cyclopropenes

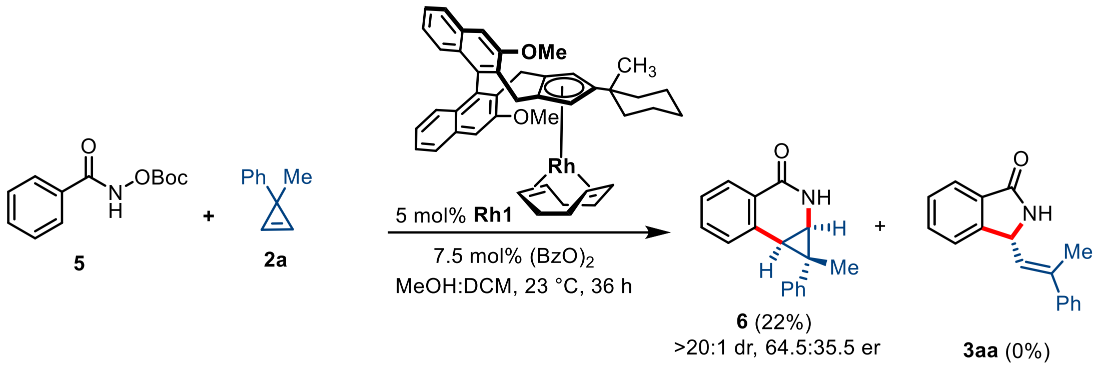

Following the procedure reported by Waldmann *et al.*,[13] without any protection from air, catalyst **Rh1** (5 mol%) and $(BzO)_2$ (7.5 mol%) were weighed in a 5 mL microwave vial. Benzohydroxymate **5** (0.1 mmol, 1.0 equiv) was added and the mixture was dissolved in a mixture of MeOH/DCM (1:1). Cyclopropene **2a** (1.5 equiv) was added to the stirred mixture at RT, and the mixture was further stirred at the same temperature for 36 h. Then the solvent was removed under reduced pressure and the crude mixture was directly submitted to silica gel column chromatography using pentane/ethyl acetate as an eluent to afford the desired [4+2] annulation product **6**.

Yield: 22% (5.5 mg), white colour solid. The spectra match with the reported literature values.[14] **1H NMR** (400 MHz, $CDCl_3$) δ 8.20 (dd, J = 7.9, 1.3 Hz, 1H), 7.52 (td, J = 7.5, 1.5 Hz, 1H), 7.45 – 7.31 (m, 6H), 7.28-7.24 (m, 1H), 6.72 (s, 1H), 3.49 (dd, J = 8.9, 3.4 Hz, 1H), 2.73 (d, J = 8.9 Hz, 1H), 1.14 (s, 3H). **R_f:** 0.44 (pentane/ethylacetate = 1/1). **IR** (ATR): 3201, 2926, 1665, 1601, 1478, 1446, 1342, 761, 699 cm^{-1}. $[\alpha]_D^{20}$ = +37.8 (c = 0.3, $CHCl_3$). **Chiral HPLC:** Chiralpak IA, 4.6 x 250 mm; 15% i-PrOH/hexane, 1.0 mL/min, 254 nm; t_R (minor) = 6.53 min, t_R (major) = 7.72 min, 64.5:35.5 *er*.

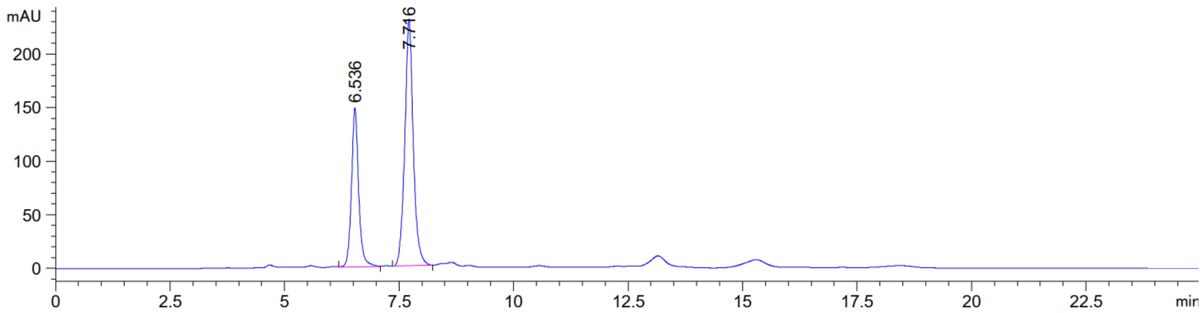

Peak #	RetTime [min]	Type	Width [min]	Area [mAU*s]	Height [mAU]	Area %
1	6.536	VV	0.1632	1619.08374	148.44254	35.2840
2	7.716	BB	0.1906	2969.64038	230.10126	64.7160

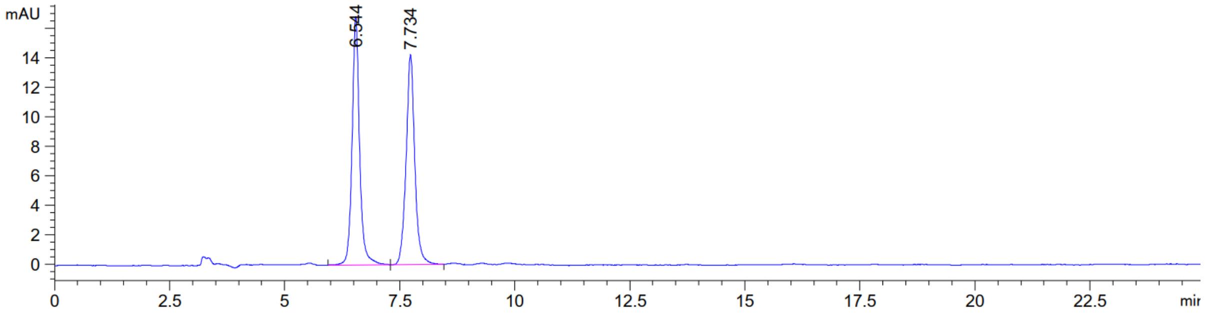

Peak #	RetTime [min]	Type	Width [min]	Area [mAU*s]	Height [mAU]	Area %
1	6.544	BB	0.1687	191.56036	16.83803	50.6174
2	7.734	BB	0.1933	186.88707	14.22665	49.3826

KIE Experiment: Parallel reactions

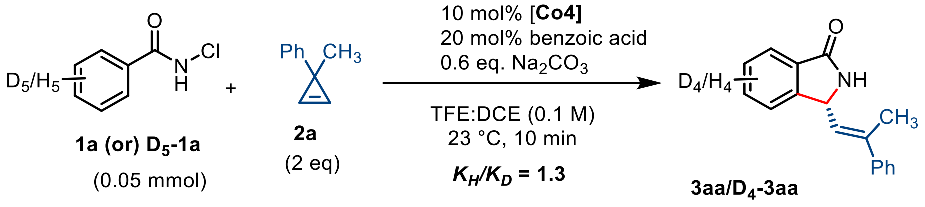

To an oven dried 5 mL microwave tube, *N*-chlorobenzamide **1a** (0.05 mmol, 1 eq.), cobalt catalyst **Co4** (10 mol%), benzoic acid (20 mol%) and Na_2CO_3 (0.6 eq.) were added. The reaction tube was connected to Schlenk line and evacuated with vacuum and then refilled with nitrogen (3 cycles). In another separate 5 mL microwave tube, deuterated *N*-chlorobenzamide[4] **1a**-d5 (0.05 mmol, 1 eq.), cobalt catalyst **Co4** (10 mol%), benzoic acid (20 mol%) and Na_2CO_3 (0.6 eq.) were added. The reaction tube was connected to Schlenk line and evacuated with vacuum and then refilled with nitrogen (3 cycles). Under nitrogen atmosphere, for each separate reaction vessel, degassed trifluoroethanol (0.25 mL) was added and stirred for 5 -10 min. Cyclopropene (2 eq.) was dissolved in degassed dichloroethane (0.25 mL) and added to the above reaction mixture. The resulting reaction mixtures were stirred in parallel at room temperature for 10 min. Each reaction mixture was quenched by addition of acetic acid (0.1 mL) and ethylacetate (0.5 mL), stirred for 10 min at 23 °C. At the end, both reaction mixtures were combined and filtered through a pad of silica gel. The filtrate was concentrated on rotavapor. NMR yield: 22% (using 1,3,5-trimethoxybenzene as an internal standard). The crude reaction mixture was purified by preparative TLC (penate:EtOAc = 1:1) to obtain the isoindolinone **3aa /3aa-D**$_4$. The primary KIE value was determined by ^{1}H NMR analysis (K_H/K_D = 1.3) .

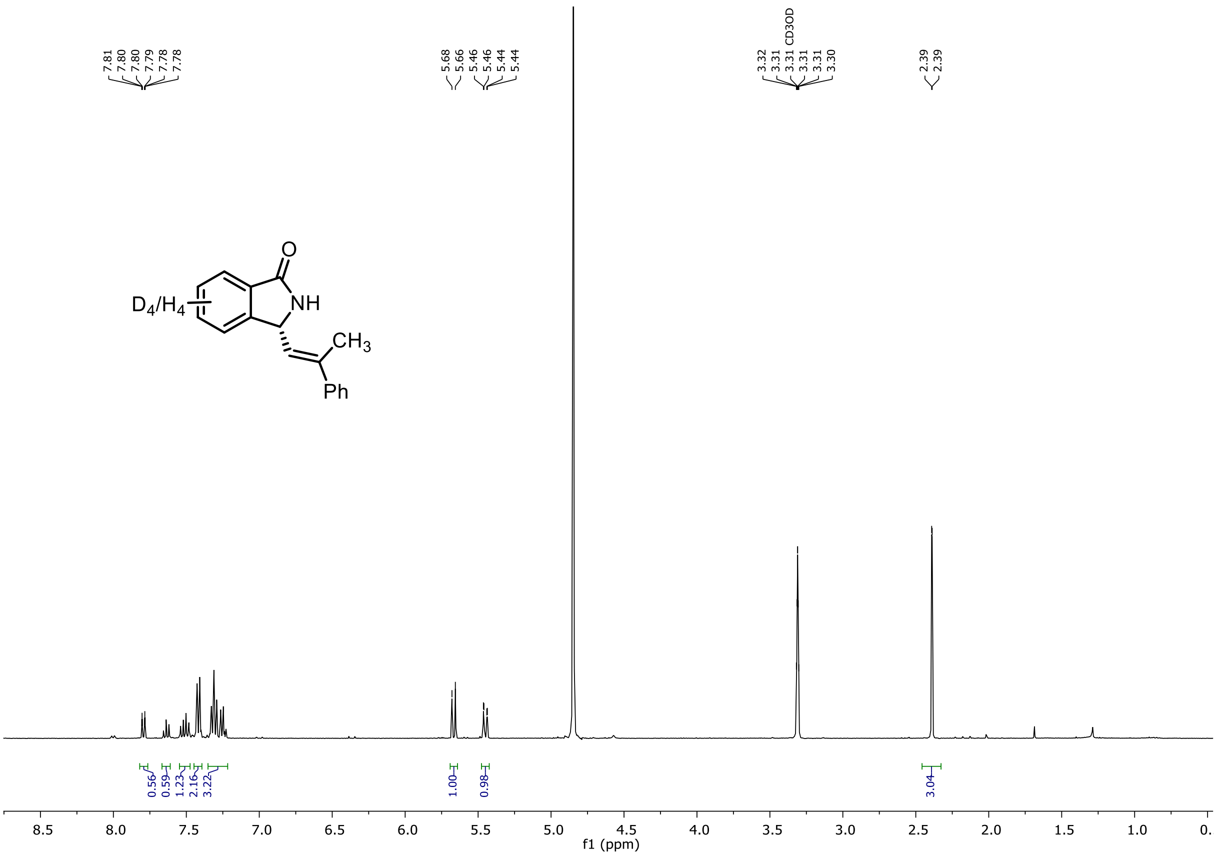

Figure S1. ^{1}H NMR for KIE experiment (**Parallel reactions**).

KIE Experiment: Competitive reaction

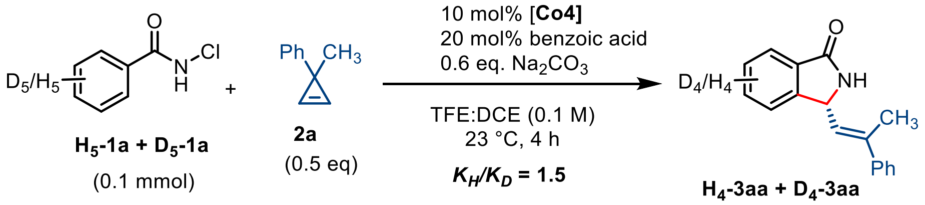

To an oven dried 5 mL microwave tube, *N*-chlorobenzamide **1a** (0.05 mmol, 0.5 eq.), deuterated *N*-chlorobenzamide **1a**-d5 (0.05 mmol, 0.5 eq.), cobalt catalyst **Co4** (10 mol%), benzoic acid (20 mol%) and Na_2CO_3 (0.6 eq.) were added. The reaction tube was connected to Schlenk line and evacuated with vacuum and then refilled with nitrogen (3 cycles). Under nitrogen atmosphere, degassed trifluoroethanol (0.5 mL) was added and stirred for 5 -10 min. Cyclopropene (0.5 eq.) was dissolved in degassed dichloroethane (0.5 mL) and added to the above reaction mixture. The resulting reaction mixtures was stirred at room temperature for 4 hours. The reaction mixture was quenched by addition of acetic acid (0.2 mL) and ethylacetate (1.0 mL), stirred for 10 min at 23 °C. The reaction mixture was filtered through a pad of silica gel and flushed with ethyl acetate. The filtrate was concentrated on rotavapor. NMR yield:

20% (using 1,3,5-trimethoxybenzene as an internal standard). The crude reaction mixture was purified by preparative TLC (penate:EtOAc = 1:1) to obtain the isoindolinone **3aa /3aa-D4**. The KIE value was determined by ^{1}H NMR analysis (K_H/K_D = 1.5) .

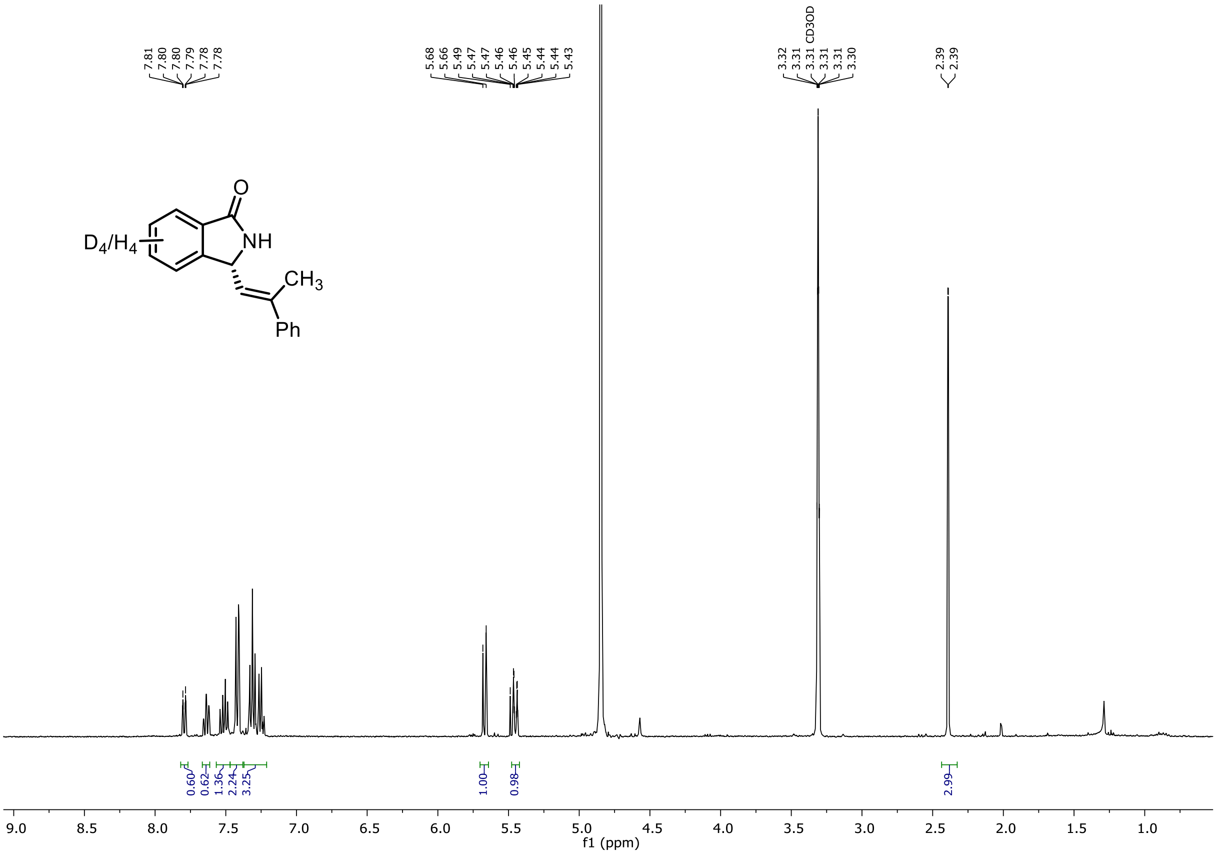

Figure S2. ^{1}H NMR for KIE experiment (**Intramolecular competitive reaction**).

X-ray crystallography data for 3aa

(*R*,*E*)-3-(2-phenylprop-1-en-1-yl)isoindolin-1-one (3aa)

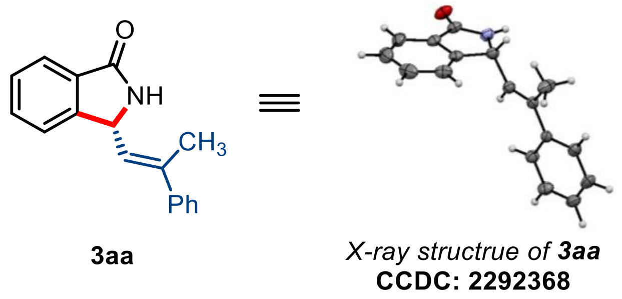

Experimental. Single clear pale colourless irregular-shaped crystals of **lve62** were used as supplied. A suitable crystal with dimensions 0.41 × 0.20 × 0.15 mm^3 was selected and mounted on a SuperNova, Dual, Cu at home/near, AtlasS2 diffractometer. The crystal was kept at a steady T = 140.00(10) K during data collection. The structure was solved with the **ShelXT** 2018/2 (Sheldrick, 2018) solution program using dual methods and by using **Olex2** 1.5 (Dolomanov et al., 2009) as the graphical interface. The model was refined with **ShelXL** 2019/3 (Sheldrick, 2015) using full matrix least squares minimisation on $\boldsymbol{F}^2$.

Crystal Data: $C_{17}H_{15}NO$, M_r = 249.30, orthorhombic, $P2_12_12_1$ (No. 19), a = 5.93229(5) Å, b = 11.01109(8) Å, c = 20.65454(15) Å, $\alpha = \beta = \gamma$ = 90°, V = 1349.175(17) Å^3, T = 140.00(10) K, Z = 4, Z' = 1, μ(Cu K$_\alpha$) = 0.597, 14448 reflections measured, 2804 unique (R_{int} = 0.0166) which were used in all calculations. The final wR_2 was 0.0658 (all data) and R_1 was 0.0255 (I≥2 σ(I)).

**Compound**	**3aa**
Formula	$C_{17}H_{15}NO$
$D_{calc.}$/ g cm^{-3}	1.227
μ/mm^{-1}	0.597
Formula Weight	249.30
Colour	clear pale colourless
Shape	irregular-shaped
Size/mm^3	0.41×0.20×0.15
T/K	140.00(10)
Crystal System	orthorhombic
Flack Parameter	0.02(5)
Hooft Parameter	0.02(5)
Space Group	$P2_12_12_1$
a/Å	5.93229(5)
b/Å	11.01109(8)
c/Å	20.65454(15)
α/°	90
β/°	90
γ/°	90
V/Å^3	1349.175(17)
Z	4
Z'	1
Wavelength/Å	1.54184
Radiation type	Cu K$_\alpha$
Θ_{min}/°	4.281
Θ_{max}/°	76.062
Measured Refl's.	14448
Indep't Refl's	2804
Refl's I≥2 σ(I)	2776
R_{int}	0.0166
Parameters	233
Restraints	0
Largest Peak	0.165
Deepest Hole	-0.110
GooF	1.069
wR_2 (all data)	0.0658
wR_2	0.0656
R_1 (all data)	0.0257
R_1	0.0255

Structure Quality Indicators

**Reflections:**	d min (Cu\a) 2Θ=152.1° 0.79	I/σ(I) 92.4	Rint m=5.17 1.66%	Full 135.4° 99% to 152.1° 100	
**Refinement:**	Shift 0.000	Max Peak 0.2	Min Peak -0.1	GooF 1.069	Hooft .02(5)

A clear pale colourless irregular-shaped crystal with dimensions 0.41 × 0.20 × 0.15 mm^3 was mounted. Data were collected using a SuperNova, Dual, Cu at home/near, AtlasS2 diffractometer operating at T = 140.00(10) K.

Data were measured using ω scans with Cu K_α radiation. The diffraction pattern was indexed and the total number of runs and images was based on the strategy calculation from the program CrysAlisPro 1.171.42.84a (Rigaku OD, 2023). The maximum resolution that was achieved was Θ = 76.062° (0.79 Å).

The unit cell was refined using CrysAlisPro 1.171.42.84a (Rigaku OD, 2023) on 12127 reflections, 84% of the observed reflections.

Data reduction, scaling and absorption corrections were performed using CrysAlisPro 1.171.42.84a (Rigaku OD, 2023). The final completeness is 100.00 % out to 76.062° in Θ. A gaussian absorption correction was performed using CrysAlisPro 1.171.42.84a (Rigaku Oxford Diffraction, 2023). The numerical absorption correction was based on gaussian integration over a multifaceted crystal model. The empirical absorption correction was done using spherical harmonics, implemented in SCALE3 ABSPACK scaling algorithm. The absorption coefficient μ of this crystal is 0.597 mm^{-1} at this wavelength (λ = 1.54184Å) and the minimum and maximum transmissions are 0.408 and 1.000.

The structure was solved and the space group $P2_12_12_1$ (# 19) determined by the ShelXT 2018/2 (Sheldrick, 2018) structure solution program using dual methods and refined by full matrix least squares minimisation on $\boldsymbol{F^2}$ using version 2019/3 of **ShelXL** (Sheldrick, 2015). All non-hydrogen atoms were refined anisotropically. Hydrogen atom positions were calculated geometrically and refined freely.

There is a single molecule in the asymmetric unit, which is represented by the reported sum formula. In other words: Z is 4 and Z' is 1.

The Flack parameter was refined to 0.02(5). Determination of absolute structure using Bayesian statistics on Bijvoet differences using the Olex2 results in 0.02(5). Note: The Flack parameter is used to determine chirality of the crystal studied, the value should be near 0, a value of 1 means that the stereochemistry is wrong, and the model should be inverted. A value of 0.5 means that the crystal consists of a racemic mixture of the two enantiomers.

Ortep-represention of (*R*,*E*)-**3aa** (thermal ellipsoids set at 50% probability). CCDC **2292368** contains the crystallographic data for (*R*,*E*)-**3aa**. These data can be obtained free of charge from The Cambridge Crystallographic Data Centre via www.ccdc.cam.ac.uk/data_request/cif.

Datablock: lve62

Bond precision:	C-C = 0.0020 A		Wavelength=1.54184
Cell:	a=5.93229(5)	b=11.01109(8)	c=20.65454(15)
	alpha=90	beta=90	gamma=90
Temperature:	140 K		

	Calculated	Reported
Volume	1349.175(18)	1349.175(17)
Space group	P 21 21 21	P 21 21 21
Hall group	P 2ac 2ab	P 2ac 2ab
Moiety formula	C17 H15 N O	C17 H15 N O
Sum formula	C17 H15 N O	C17 H15 N O
Mr	249.30	249.30
Dx,g cm-3	1.227	1.227
Z	4	4
Mu (mm-1)	0.597	0.597
F000	528.0	528.0
F000'	529.47	
h,k,lmax	7,13,26	7,13,25
Nref	2823[1658]	2804
Tmin,Tmax	0.865,0.912	0.408,1.000
Tmin'	0.781	

Correction method= # Reported T Limits: Tmin=0.408 Tmax=1.000
AbsCorr = GAUSSIAN

Data completeness= 1.69/0.99   Theta(max)= 76.062

R(reflections)= 0.0255(2776)   wR2(reflections)= 0.0658(2804)

S = 1.069   Npar= 233

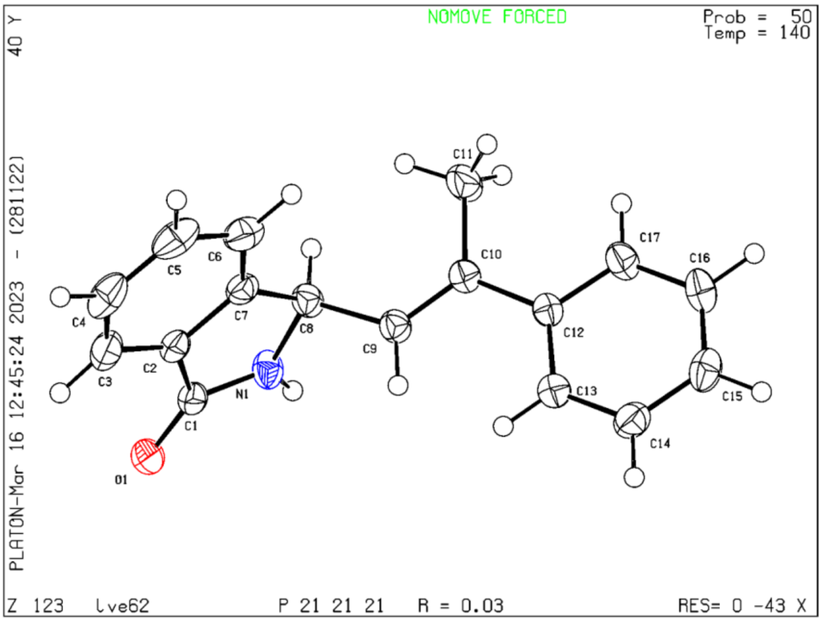

Computational Details

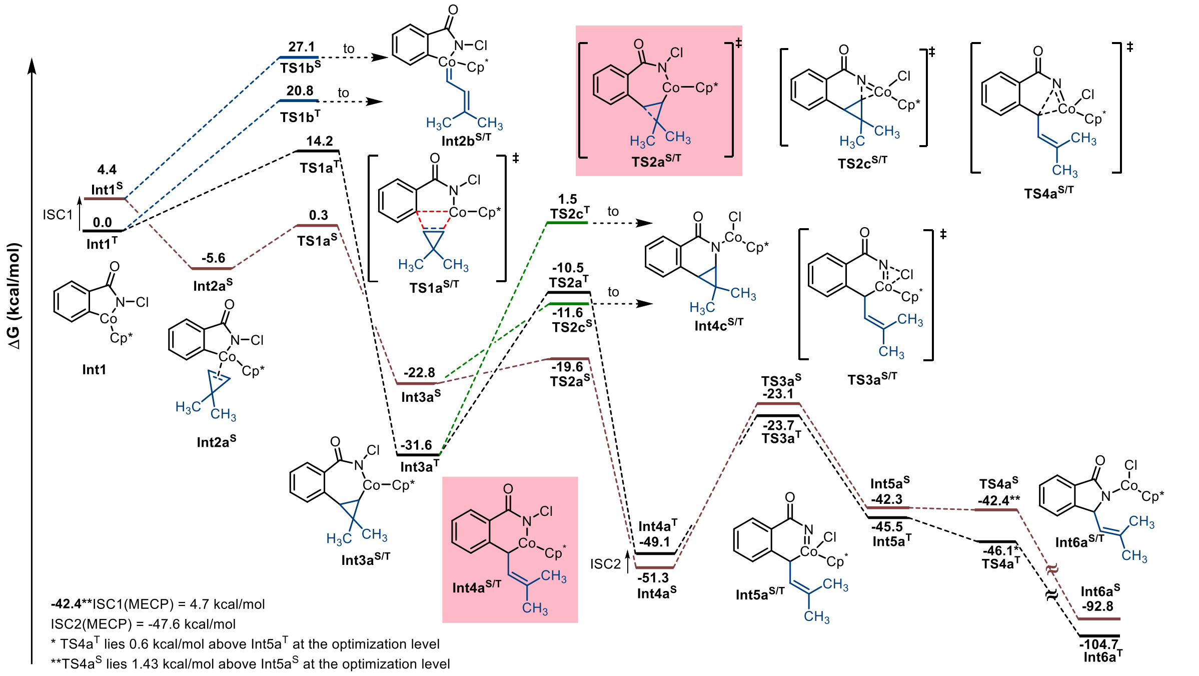

Figure S3. Singlet and triplet potential energy surfaces for C−H activation/cyclopropene insertion processes by Co computed at the B3PW91-D3(BJ)/def2-TZVP//B3PW91-D3(BJ)/def2-SVP theoretical level in implicit TFE solvent.

Table S7. Computed electronic energies, free energy corrections, single point energies, total free energies (each in Hartree), as well as relative free energies (in kcal/mol) for relevant cobalt species.

Species	B3PW91-D3(BJ)/def2-SVP Electronic Energy	B3PW91-D3(BJ)/def2-SVP Free Energy Correction	B3PW91-D3(BJ)/def2-TZVP Single Point Electronic Energy	Total Free Energy	Relative Free Energy (kcal/mol)
dimethylcyclopropene	-195.063790	0.088970	-195.273588	-195.184618	--
Int1-Triplet	-2631.093424	0.271360	-2632.300937	-2632.029577	0.0
Int1-Singlet	-2631.090092	0.274523	-2632.297013	-2632.022490	4.4
Int1-MECP	-2631.088472	0.273749	-2632.295898	-2632.022149	4.7
Int2a-Singlet	-2826.197807	0.383937	-2827.607095	-2827.223158	-5.6
TS1a-Singlet	-2826.192659	0.386108	-2827.599755	-2827.213647	0.3
TS1a-Triplet	-2826.161738	0.378529	-2827.570108	-2827.191579	14.2
TS1b-Singlet	-2826.139370	0.381152	-2827.552186	-2827.171034	27.1
TS1b-Triplet	-2826.142199	0.374649	-2827.555721	-2827.181072	20.8
Int3a-Singlet	-2826.230768	0.387427	-2827.638027	-2827.250600	-22.8
Int3a-Triplet	-2826.241125	0.382897	-2827.647384	-2827.264487	-31.6
Int3a-MECP	-2826.225668	0.386085	-2827.634847	-2827.248762	-21.7
TS2a-Singlet	-2826.219825	0.385646	-2827.631155	-2827.245509	-19.6
TS2a-Triplet	-2826.200081	0.378953	-2827.609845	-2827.230892	-10.5
TS2c-Singlet	-2826.213422	0.385019	-2827.617098	-2827.232079	-11.2
TS2c-Triplet	-2826.193184	0.383091	-2827.594922	-2827.211831	1.5
Int4a-Singlet	-2826.277136	0.388119	-2827.684059	-2827.295940	-51.3
Int4a-Triplet	-2826.264752	0.380950	-2827.673319	-2827.292369	-49.1
Int4a-MECP	-2826.263743	0.384882	-2827.675000	-2827.290118	-47.6
TS3a-Singlet	-2826.226504	0.381421	-2827.632487	-2827.251066	-23.1

TS3a-Triplet	-2826.222291	0.380030	-2827.632021	-2827.251991	-23.7
Int5a-Singlet	-2826.255265	0.384385	-2827.665922	-2827.281537	-42.3
Int5a-Triplet	-2826.258267	0.380851	-2827.667600	-2827.286749	-45.5
Int5a-MECP	-2826.252504	0.383269	-2827.663155	-2827.279886	-41.2
TS4a-Singlet	-2826.252984	0.384531	-2827.666240	-2827.281709	-42.4
TS4a-Triplet	-2826.257299	0.379560	-2827.667274	-2827.287714	-46.1
Int6a-Singlet	-2826.339583	0.387940	-2827.749986	-2827.362046	-92.8
Int6a-Triplet	-2826.355704	0.382058	-2827.763130	-2827.381072	-104.7
Int6a-MECP	-2826.336604	0.386508	-2827.748688	-2827.362180	-92.9

Table S8. Computed electronic energies, free energy corrections, single point energies, total free energies (each in Hartree), as well as relative free energies (in kcal/mol) for relevant rhodium species.

Species	B3PW91-D3(BJ)/def2-SVP Electronic Energy	B3PW91-D3(BJ)/def2-SVP Free Energy Correction	B3PW91-D3(BJ)/def2-TZVP Single Point Electronic Energy	Total Free Energy	Relative Free Energy (kcal/mol)
dimethylcyclopropene	-195.063790	0.088970	-195.273588	-195.184618	--
Int1	-1320.326329	0.404996	-1321.692076	-1321.287080	0.0
Int2	-1515.434707	0.513981	-1517.001325	-1516.487344	-9.8
TS1	-1515.413557	0.513772	-1516.980901	-1516.467129	2.9
Int3	-1515.449733	0.514889	-1517.018014	-1516.503125	-19.7
Int4	-1515.475476	0.519094	-1517.038408	-1516.519314	-29.9
TS2a	-1515.444338	0.513956	-1517.019594	-1516.505638	-21.3
TS2b	-1515.442384	0.514462	-1517.011011	-1516.496549	-15.6
Int5a	-1515.455051	0.514461	-1517.0307	-1516.516239	-27.9
TS3	-1515.452503	0.515901	-1517.027032	-1516.511131	-24.7
Int6	-1515.561925	0.516541	-1517.135028	-1516.618487	-92.1

Table S9. Computed electronic energies of singlet cobalt complexes using restricted, restricted open shell, and unrestricted formalisms. Computed total spin is included for the unrestricted computation as a test for spin contamination. Energies in Hartree.

Species	RB3PW91-D3(BJ)/def2-SVP Electronic Energy	ROB3PW91-D3(BJ)/def2-SVP Electronic Energy	UB3PW91-D3(BJ)/def2-SVP Electronic Energy
Int1-Singlet	-2631.090092	-2631.090092	-2631.090846 (S^2=0.27)
Int2a-Singlet	-2826.197807	-2826.197807	-2826.197807 (S^2=0)
TS1a-Singlet	-2826.192659	-2826.192659	-2826.192659 (S^2=0)
TS1b-Singlet	-2826.139370	-2826.139370	-2826.139370 (S^2=0)
Int3a-Singlet	-2826.230768	-2826.230768	-2826.230768 (S^2=0)
TS2a-Singlet	-2826.219825	-2826.219825	-2826.219825 (S^2=0)
TS2c-Singlet	-2826.213422	-2826.213422	-2826.213422 (S^2=0)
Int4a-Singlet	-2826.277136	-2826.277136	-2826.277136 (S^2=0)
TS3a-Singlet	-2826.226504	-2826.226504	-2826.226504 (S^2=0)
Int5a-Singlet	-2826.255265	-2826.255265	-2826.255840 (S^2=0.26)
TS4a-Singlet	-2826.252984	-2826.252984	-2826.253417 (S^2=0.26)
Int6a-Singlet	-2826.339583	-2826.339583	-2826.339583 (S^2=0)

Table S10. Computed total spin for triplet cobalt complexes.

Species	UB3PW91-D3(BJ)/def2-SVP Computed Spin (S^2)	UB3PW91-D3(BJ)/def2-SVP Computed Annihilated Spin (S^2A)
Int1-Triplet	2.15	2.00
TS1a-Triplet	2.10	2.00
TS1b-Triplet	2.08	2.00

Int3a-Triplet	2.04	2.00
TS2a-Triplet	2.11	2.00
TS2c-Triplet	2.12	2.01
Int4a-Triplet	2.07	2.00
TS3a-Triplet	2.19	2.01
Int5a-Triplet	2.20	2.01
TS4a-Triplet	2.40	2.04
Int6a-Triplet	2.09	2.00

Table S11. Localized orbital bonding analysis computed oxidation state of the transition metal in the catalytic cycle.

Species	Co-Singlet	Co-Triplet	Rh
Int1	+3	+3	+3
Int2a/Int2	+3	NA	+3
Int3a/Int3	+3	+3	+3
Int4a/Int4	+3	+3	+3
Int5a/Int5	+5	+3	+5
Int6a/Int6	+3	+3	+3

Table S12. Computed singlet/triplet electronic energy differences for cobalt species using the B3PW91-D3(BJ) functional with selected basis sets on B3PW91-D3(BJ)/def2-SVP optimized geometries. Deviations, given in parenthesis, calculated using the aug-cc-pVTZ basis set as reference. MAD = mean absolute deviation, MUD = mean unsigned deviation. Basis set employed in this work shown in bold. All values in kcal/mol.

	def2-SVP	6-311+G(d,p)-H,C,N,O,Cl LANL2TZ-Co	6-311+G(d,p)-H,C,N,O,Cl SDD-Co	**def2-TZVP**	def2-TZVPPD	aug-cc-pVTZ
Int1	-2.09 (0.13)	-6.01 (-3.78)	-6.85 (-4.63)	**-2.46 (-0.24)**	-2.45 (-0.22)	-2.22
Int3a	-6.50 (-0.72)	-8.07 (-2.29)	-9.67 (-3.89)	**-5.87 (-0.09)**	-5.90 (-0.12)	-5.78
Int4a	7.77 (0.84)	4.69 (-2.24)	2.98 (-3.95)	**6.74 (-0.19)**	6.78 (-0.15)	6.93
Int5a	-1.88 (-0.81)	-2.63 (-1.56)	-3.98 (-2.90)	**-1.05 (0.02)**	-1.11 (-0.04)	-1.07
Int6a	-10.12 (-1.50)	-9.35 (-0.74)	-11.61 (-3.00)	**-8.25 (0.37)**	-8.44 (0.17)	-8.61
TS1a	19.40 (0.82)	16.41 (-2.18)	15.09 (-3.49)	**18.60 (0.02)**	18.52 (-0.07)	18.59
TS1b	-1.78 (0.38)	-5.00 (-2.85)	-6.17 (-4.02)	**-2.22 (-0.06)**	-2.23 (-0.07)	-2.15
TS2a	12.39 (-1.33)	11.20 (-2.53)	9.60 (-4.13)	**13.37 (-0.35)**	13.48 (-0.25)	13.72
TS2c	12.70 (-1.20)	12.05 (-1.85)	10.64 (-3.25)	**13.92 (0.02)**	13.81 (-0.09)	13.90
TS3a	2.64 (2.40)	-1.34 (-1.59)	-1.73 (-1.97)	**0.29 (0.05)**	0.16 (-0.08)	0.24
TS4a	-2.71 (-2.14)	-2.97 (-2.40)	-4.38 (-3.81)	**-0.65 (-0.08)**	-0.66 (-0.09)	-0.57
MAD	1.12	2.18	3.55	**0.13**	0.12	--
MUD	-0.28	-2.18	-3.55	**-0.05**	-0.09	--

Table S13. Computed CM5 charges for key atoms for Int3a (cobalt) and Int4 (rhodium). Computations at the B3PW91-D3(BJ)/def2-SVP level.

Co ($Int3a^S$)	Rh (Int4)
+0.34 (Co)	+0.58 (Rh)
+0.06 (Cl)	-0.30 (O_{56})

Table S14. Computed single point electronic energies (on B3PW91-D3(BJ)/def2-SVP optimized geometries) of cobalt species with various basis sets. Values in Hartree.

	6-311+G(d,p)-H,C,N,O,Cl LANL2TZ-Co	6-311+G(d,p)-H,C,N,O,Cl SDD-Co	def2-TZVPPD	aug-cc-pVTZ
Int1-Singlet	-1394.553308	-1395.299108	-2632.308761	-2632.326464
Int1-Triplet	-1394.562884	-1395.310027	-2632.312662	-2632.330009
Int3a-Singlet	-1589.879920	-1590.624745	-2827.653040	-2827.666241
Int3a-Triplet	-1589.892781	-1590.640156	-2827.662444	-2827.675457
Int4a-Singlet	-1589.926382	-1590.670164	-2827.698860	-2827.712054
Int4a-Triplet	-1589.918902	-1590.665412	-2827.688060	-2827.701008
Int5a-Singlet	-1589.911055	-1590.655889	-2827.680735	-2827.694685
Int5a-Triplet	-1589.915253	-1590.662224	-2827.682506	-2827.696396
Int6a-Singlet	-1589.995128	-1590.740803	-2827.764889	-2827.778888
Int6a-Triplet	-1590.010027	-1590.759306	-2827.778341	-2827.792614
TS1a-Singlet	-1589.842403	-1590.585757	-2827.614673	-2827.627791
TS1a-Triplet	-1589.816255	-1590.561703	-2827.585159	-2827.598172
TS1b-Singlet	-1589.795184	-1590.538597	-2827.567024	-2827.580181
TS1b-Triplet	-1589.803157	-1590.548433	-2827.570574	-2827.583613
TS2a-Singlet	-1589.873710	-1590.617911	-2827.646105	-2827.659530
TS2a-Triplet	-1589.855866	-1590.602617	-2827.624629	-2827.637661
TS2c-Singlet	-1589.860181	-1590.605577	-2827.631894	-2827.645993
TS2c-Triplet	-1589.840984	-1590.588615	-2827.609883	-2827.623845
TS3a-Singlet	-1589.876735	-1590.621582	-2827.647090	-2827.661068
TS3a-Triplet	-1589.878874	-1590.624335	-2827.646828	-2827.660679
TS4a-Singlet	-1589.910813	-1590.656208	-2827.681152	-2827.695238
TS4a-Triplet	-1589.915543	-1590.663185	-2827.682196	-2827.696144

References

1. Ozols, K.; Jang, Y. S.; Cramer, N. Chiral Cyclopentadienyl Cobalt(III) Complexes Enable Highly Enantioselective 3d-Metal-Catalyzed C-H Functionalizations. *J. Am. Chem. Soc.* **2019**, *141*, 5675−5680.
2. Herraiz, A. G.; Cramer, N. Cobalt(III)- Catalyzed Diastereo- and Enantioselective Three-Component C-H Functionalization. *ACS Catal*. **2021**, *11*, 11938–11944.
3. Duchemin, C.; Cramer, N. Enantioselective $Cp^{x}Rh^{III}$-Catalyzed Carboaminations of Acrylates. *Angew. Chem., Int. Ed.* **2020**, *59*, 14129−14133.
4. (a) Yu, X.; Chen, K.; Guo, S.; Shi, P.; Song, C.; Zhu, J. Direct Access to Cobaltacycles via C−H Activation: *N*-Chloroamide-Enabled Room-Temperature Synthesis of Heterocycles. *Org. Lett.* **2017**, *19*, 5348-5351. (b) Muniraj, N.; Prabhu, K. R. Cobalt(III)-Catalyzed [4 + 2] Annulation of *N*-Chlorobenzamides with Maleimides. *Org. Lett.* **2019**, *21*, 1068-1072.
5. Phan, D. H. T.; Kou, K. G. M.; Dong, V. M. Enantioselective Desymmetrization of Cyclopropenes by Hydroacylation. *J. Am. Chem. Soc.* **2010**, *132*, 16354−16355.
6. Parra, A.; Amenós, L.; Guisán-Ceinos, M.; López, A.; Ruano, J. L. G.; Tortosa, M. Copper-Catalyzed Diastereo- and Enantioselective Desymmetrization of Cyclopropenes: Synthesis of Cyclopropylboronates. *J. Am. Chem. Soc.* **2014**, *136*, 15833−15836.
7. Guo, P.; Sun, W.; Liu, Y.; Li, Y.-X.; Loh, T.-P.; Jiang, Y. Stereoselective Synthesis of Vinylcyclopropa[b]indolines via a Rh-Migration Strategy. *Org. Lett*. **2020**, *22*, 5978−5983.
8. Li, Z.; Zhao, J.; Sun, B.; Zhou, T.; Liu, M.; Liu, S.; Zhang, M.; Zhang, Q. Asymmetric Nitrone Synthesis via Ligand-Enabled Copper-Catalyzed Cope-Type Hydroamination of Cyclopropene with Oxime. *J. Am. Chem. Soc.* **2017**, *139*, 11702−11705.
9. Nie, S.; Lu, A.; Kuker, E. L.; Dong, V. M. Enantioselective Hydrothiolation: Diverging Cyclopropenes through Ligand Control. *J. Am. Chem. Soc.* **2021**, *143*, 6176−6184.
10. Rubina, M.; Rubin, M.; Gevorgyan, V. Catalytic Enantioselective Hydrostannation of Cyclopropenes. *J. Am. Chem. Soc.* **2004**, *126*, 3688−3689.
11. González, M. J.; González, J.; López, L. A.; Vicente, R. Zinc-Catalyzed Alkene Cyclopropanation through Zinc Vinyl Carbenoids Generated from Cyclopropenes. *Angew. Chem., Int. Ed.* **2015**, *54*, 12139−12143.
12. Liu, D.; Liu, C.; Li, H.; Lei, A. Copper Catalysed Oxidative C–H/C–H Coupling between Olefins and Simple Ethers. *Chem. Comm.* **2014,** *50*, 3623-3626.
13. Shaaban, S., Li, H., Merten, C., Antonchick, A. P. Waldmann, H., Rhodium (III)-Catalyzed Enantioselective Benzamidation of Cyclopropenes. *Synthesis*. **2021**, *53,* 2192-2200.
14. Semakul, N.; Jackson, K. E.; Paton, R. S.; Rovis, T. Heptamethyl indenyl (Ind*) Enables Diastereoselective Benzamidation of Cycloproenes via Rh(III)-Catalyzed C−H Activation. *Chem. Sci.* **2017**, *8*, 1015−1020.

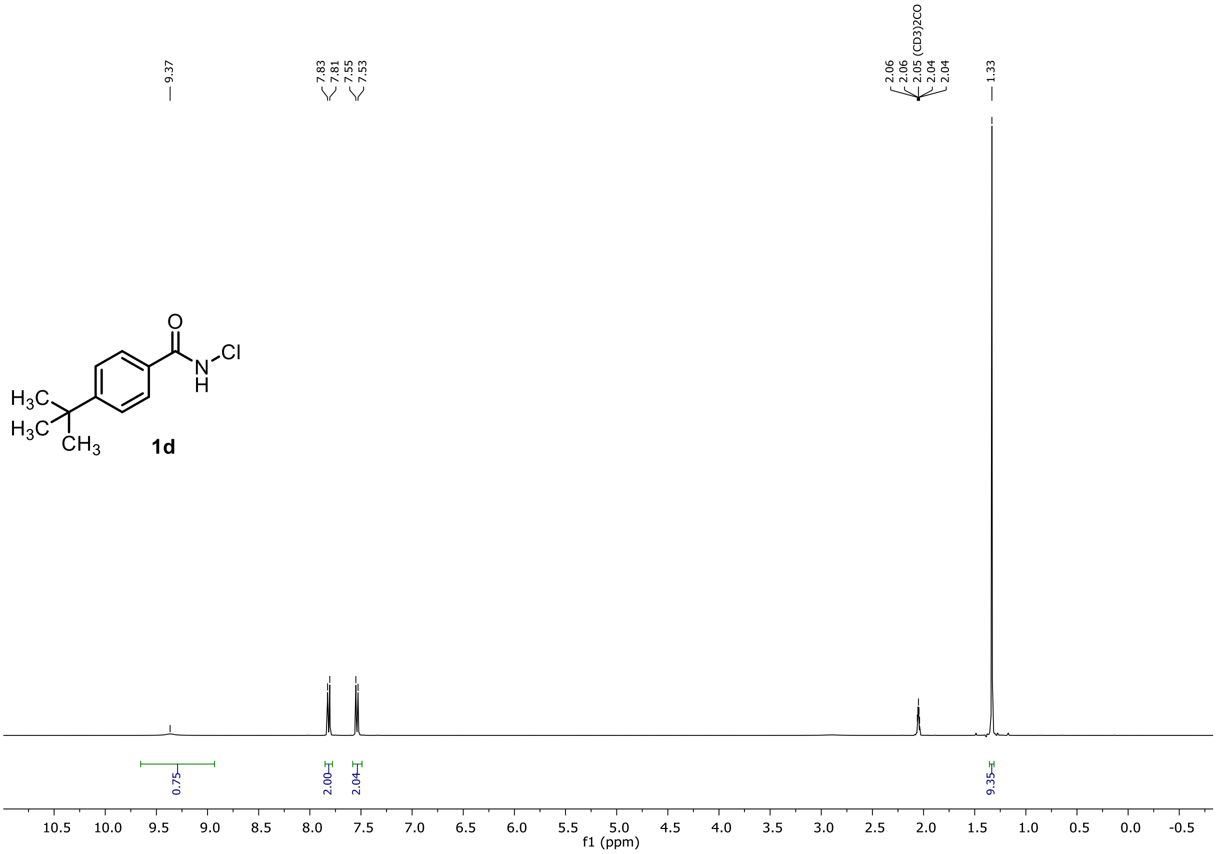

^{1}H NMR of **1d** (400 MHz, Acetone-d_6)

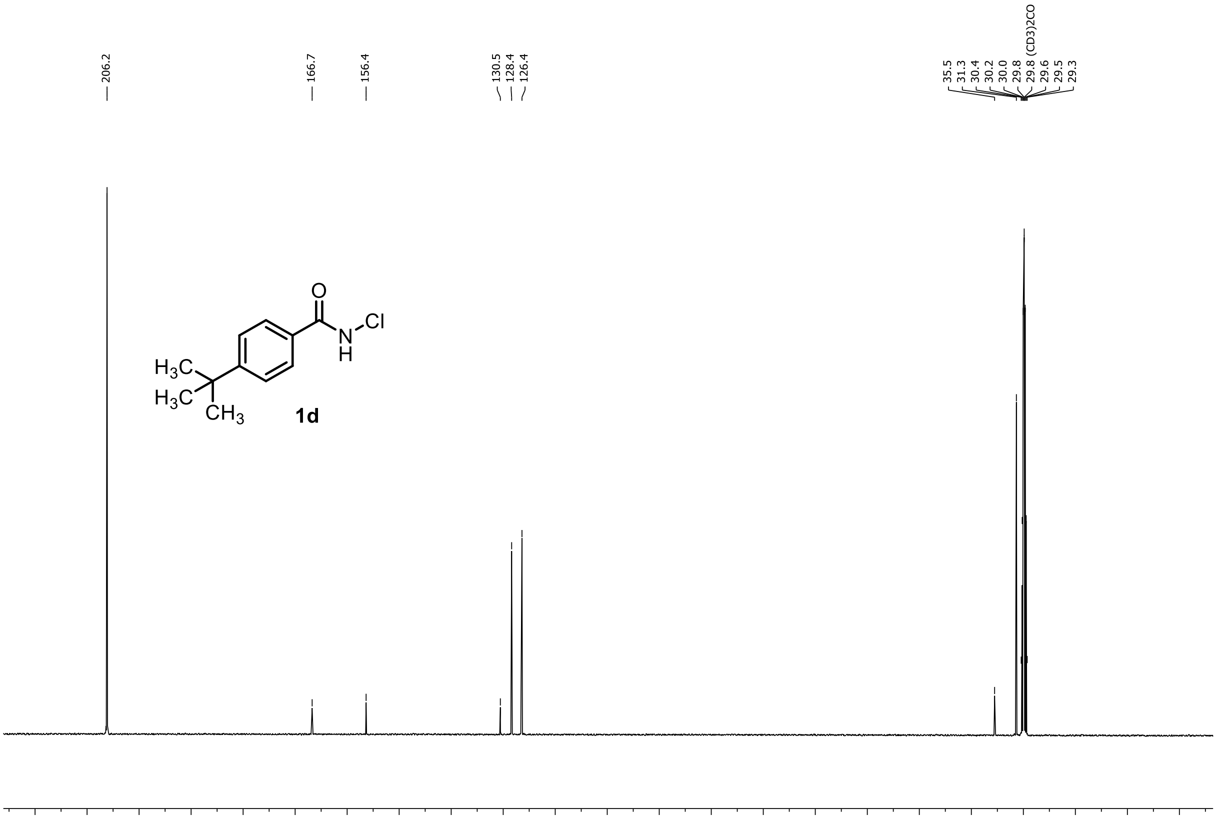

^{13}C NMR of **1d** (101 MHz, Acetone-d_6)

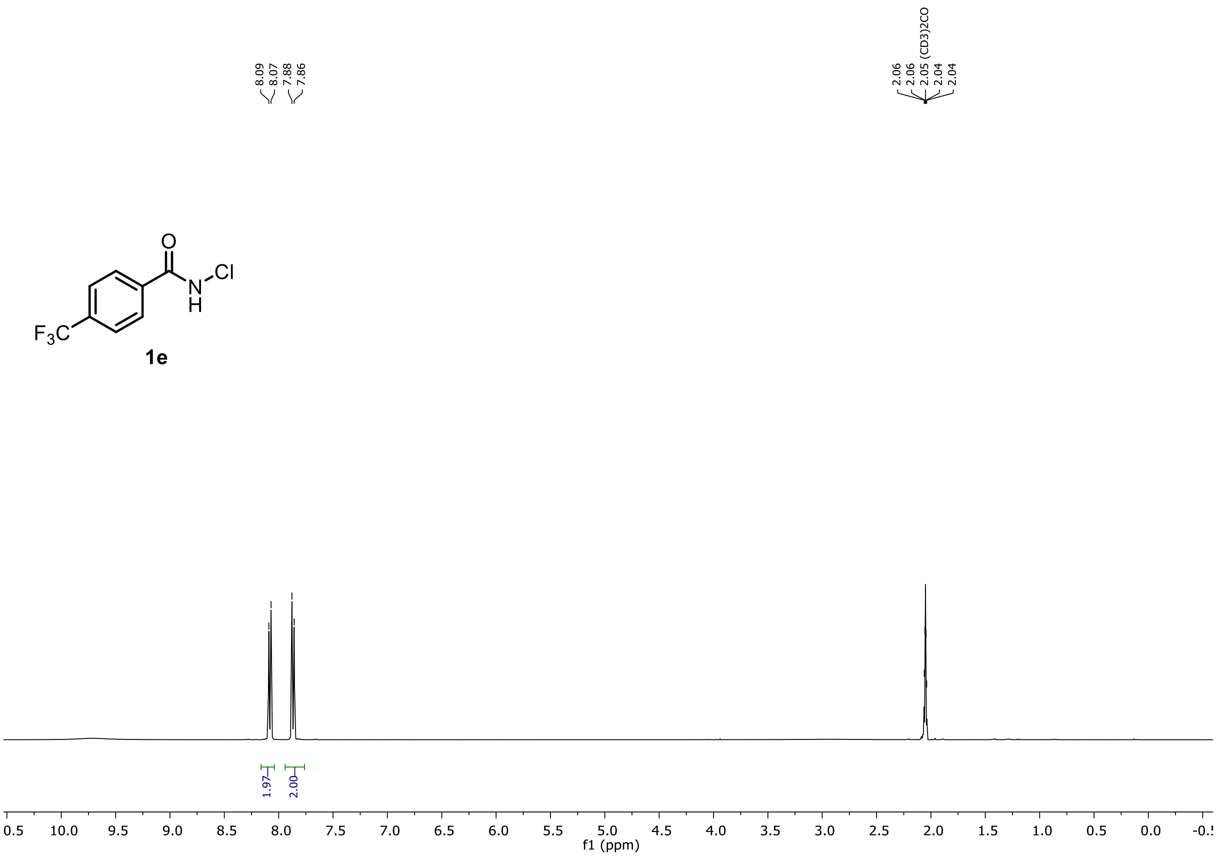

^{1}H NMR of **1e** (400 MHz, Acetone-d$_6$)

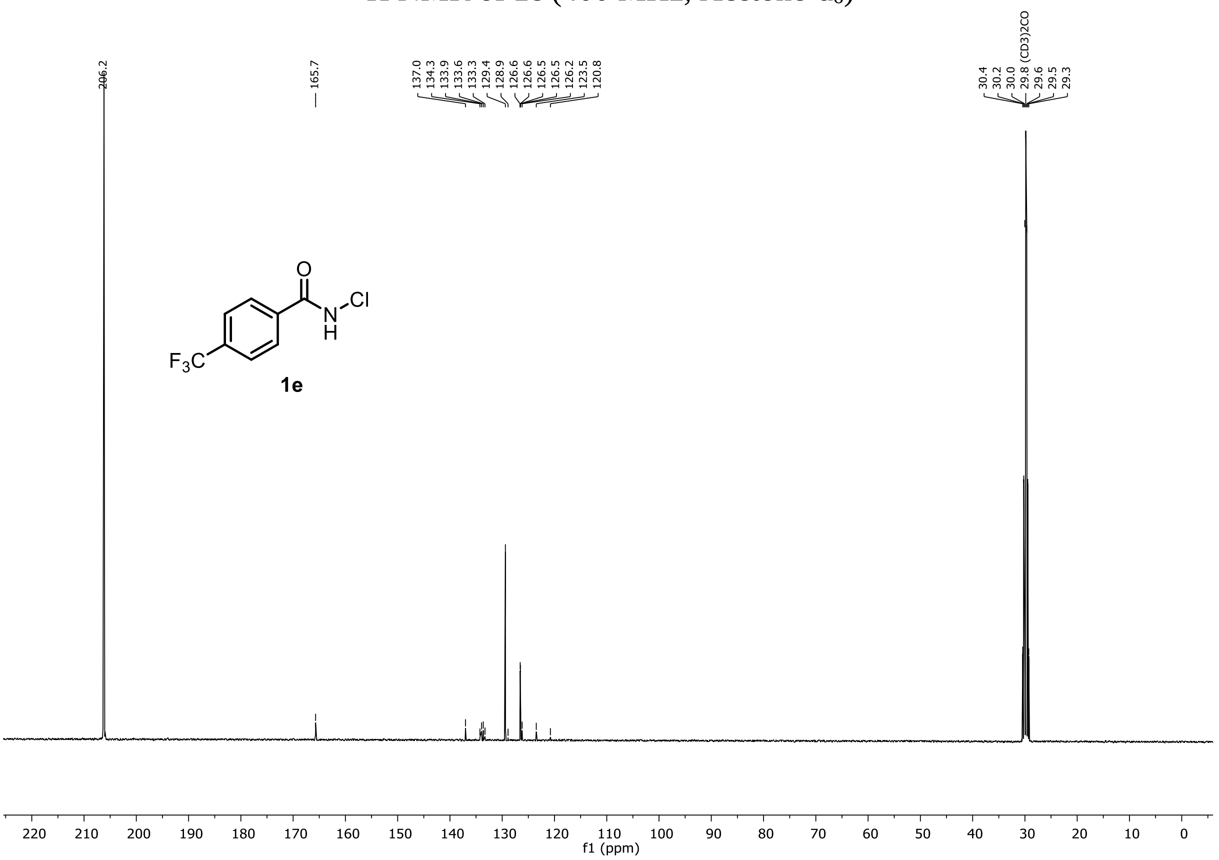

^{13}C NMR of **1e** (101 MHz, Acetone-d$_6$)

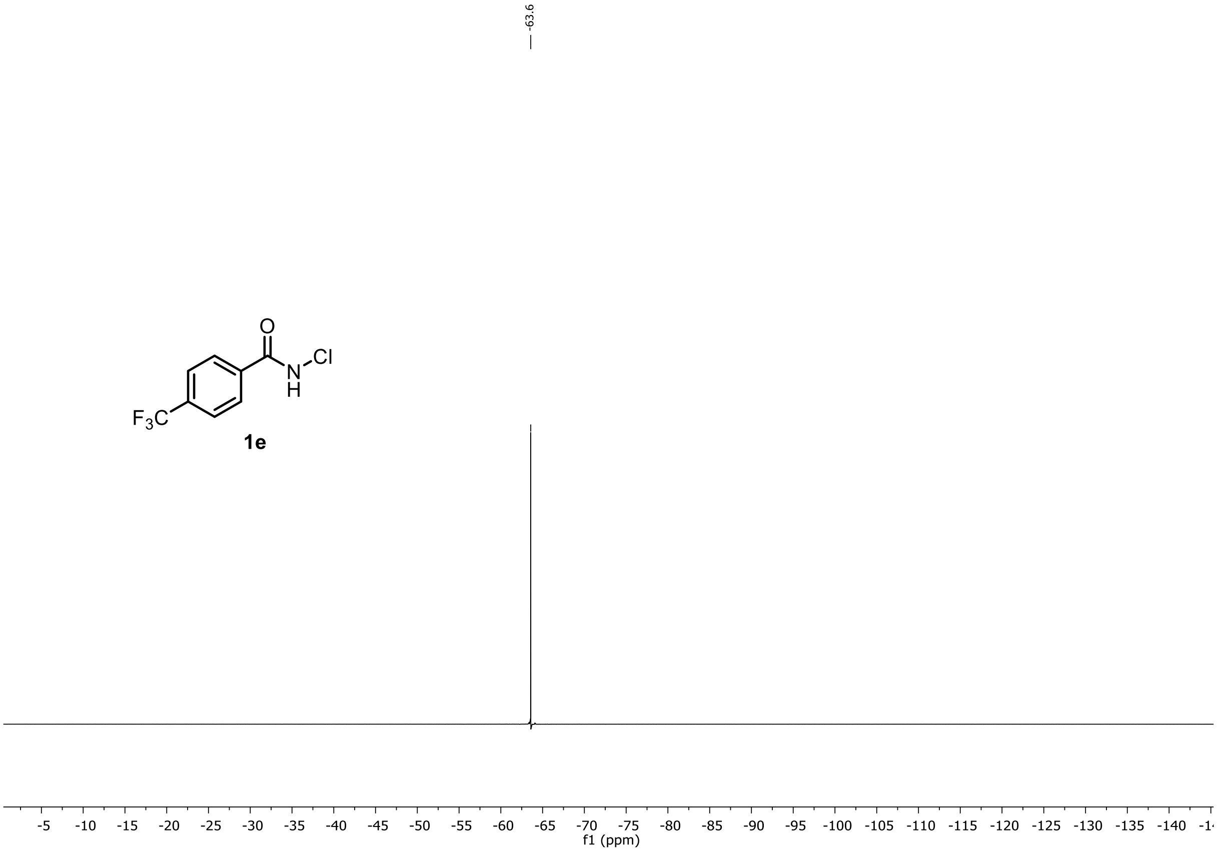

^{19}F NMR of **1e** (376 MHz, Acetone-d$_6$)

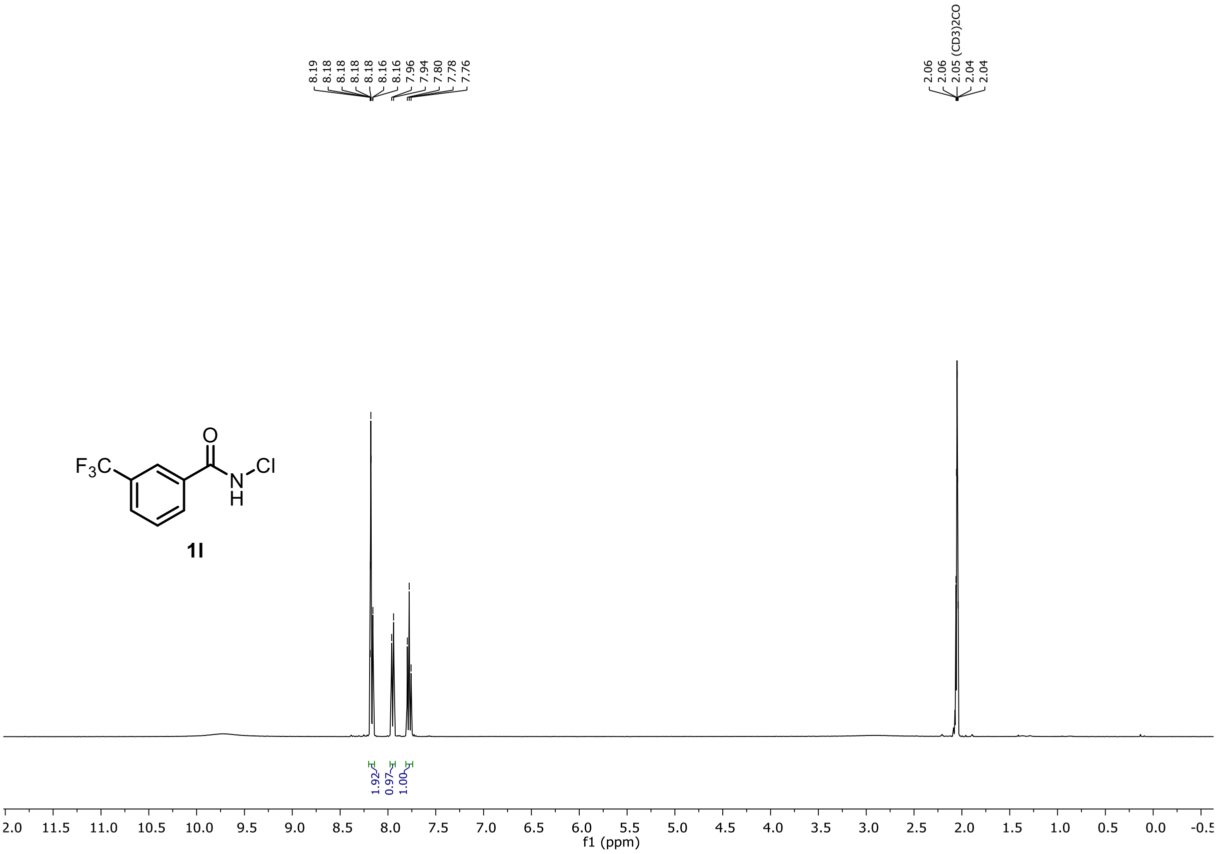

^{1}H NMR of **1l** (400 MHz, Acetone-d$_6$)

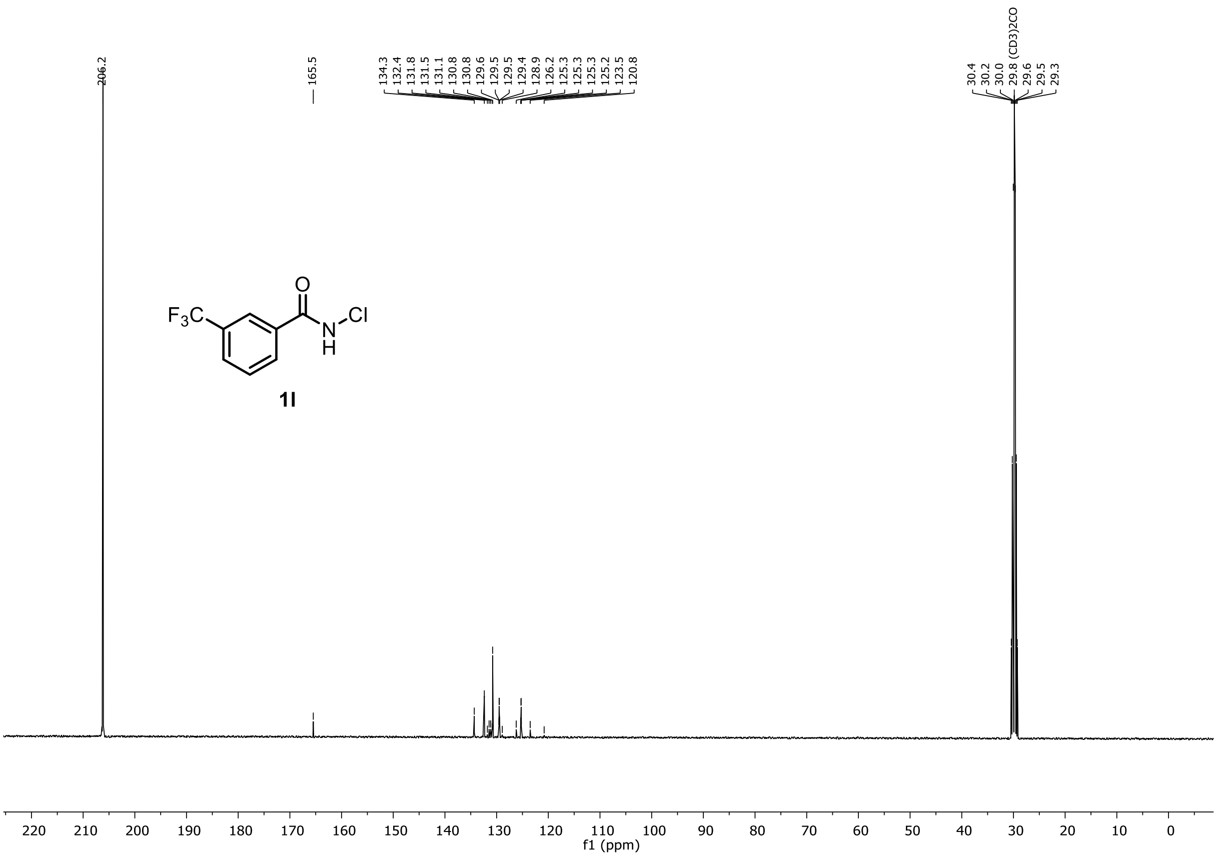

^{13}C NMR of **1l** (101 MHz, Acetone-d$_6$)

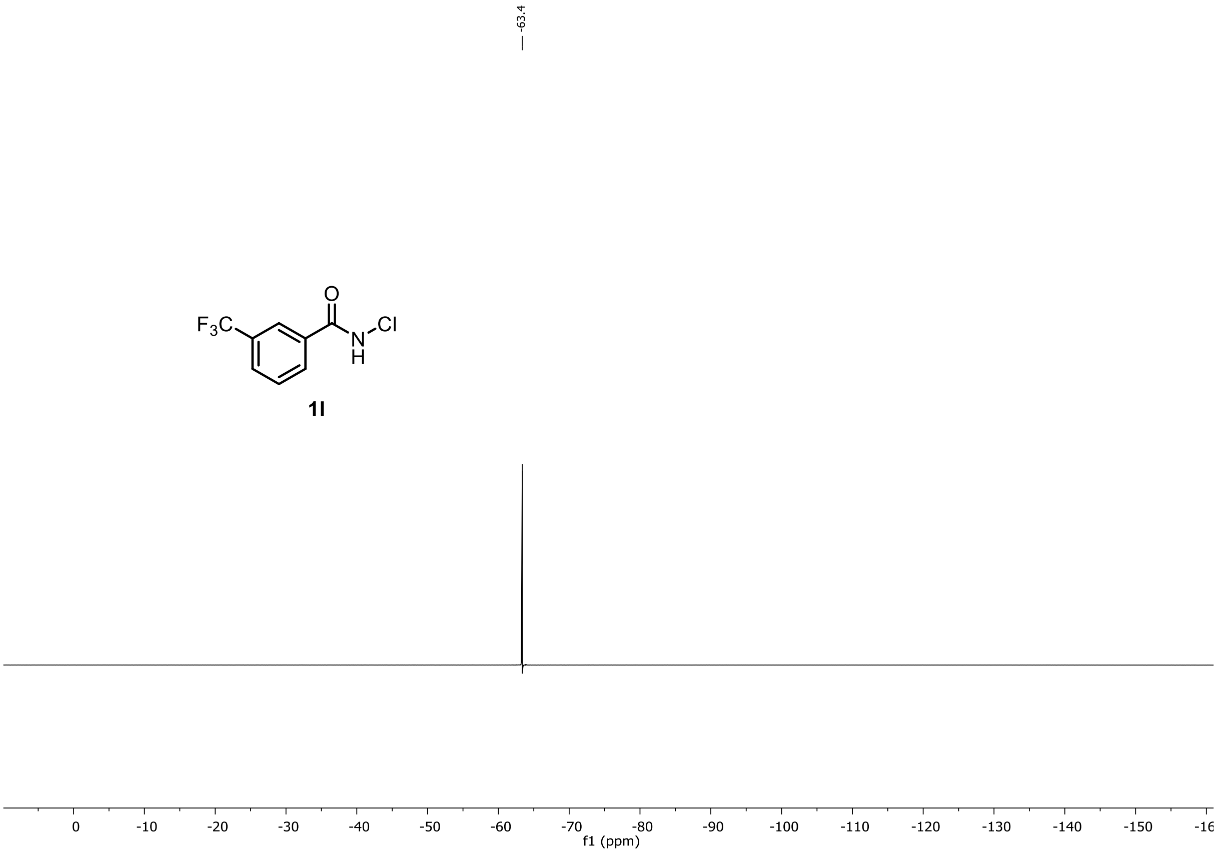

^{19}F NMR of **1l** (376 MHz, Acetone-d$_6$)

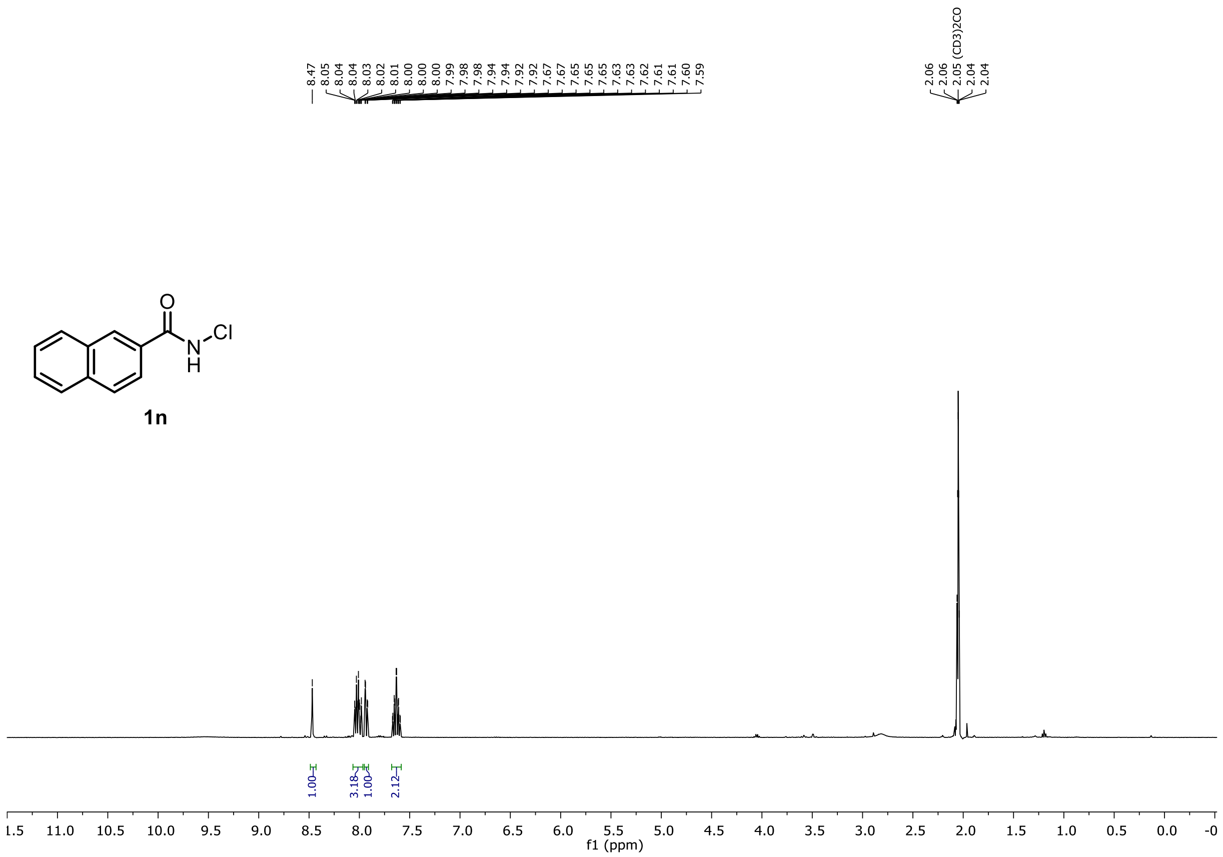

^{1}H NMR of **1n** (400 MHz, Acetone-d$_6$)

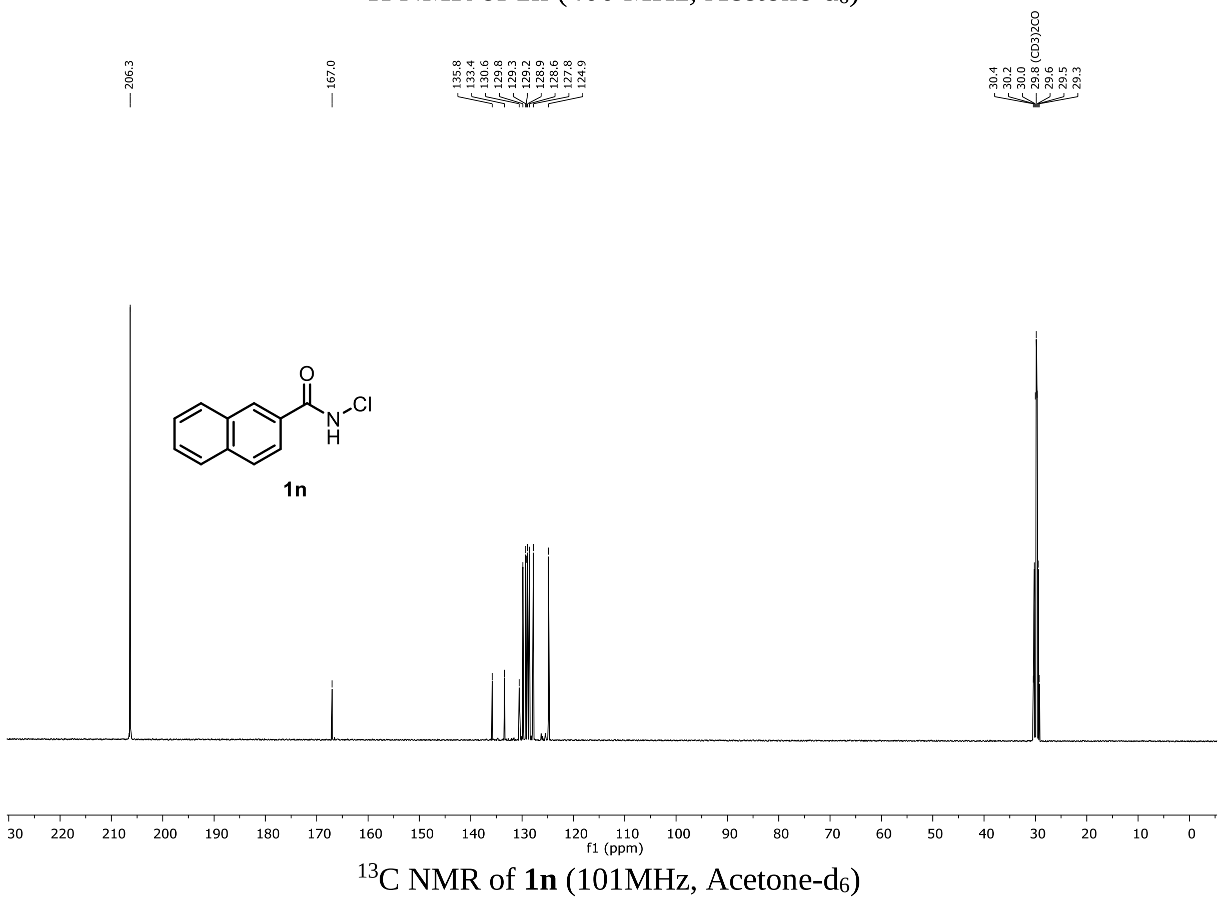

^{13}C NMR of **1n** (101MHz, Acetone-d$_6$)

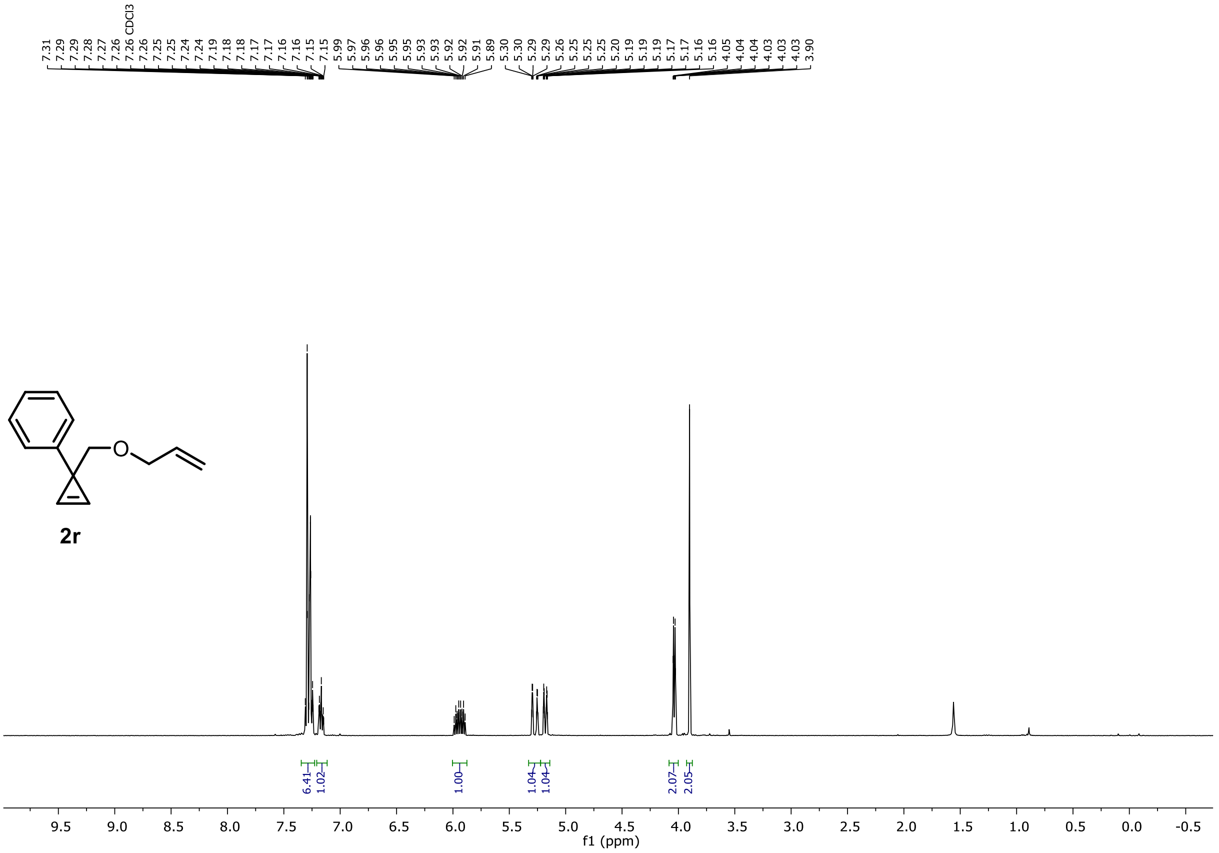

^{1}H NMR of **2r** (400 MHz, $CDCl_3$)

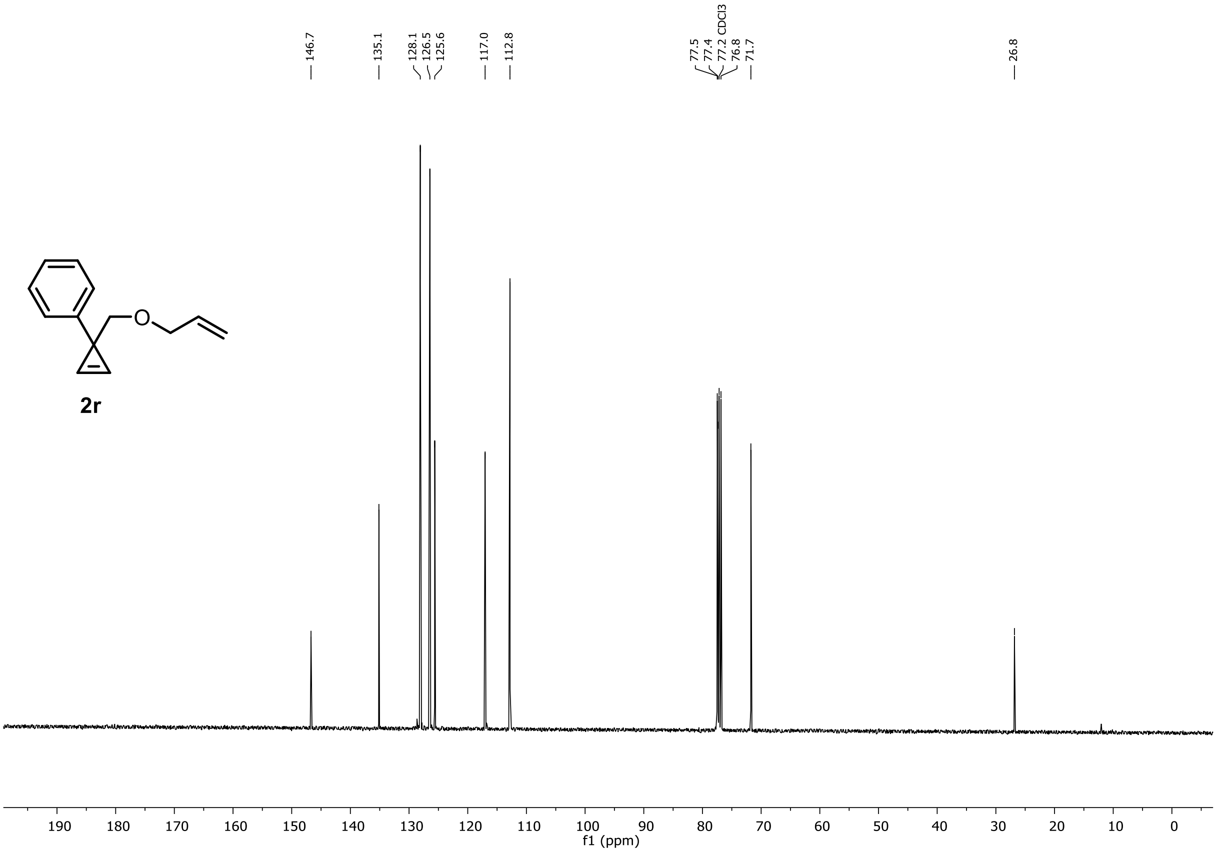

^{13}C NMR of **2r** (101 MHz, $CDCl_3$)

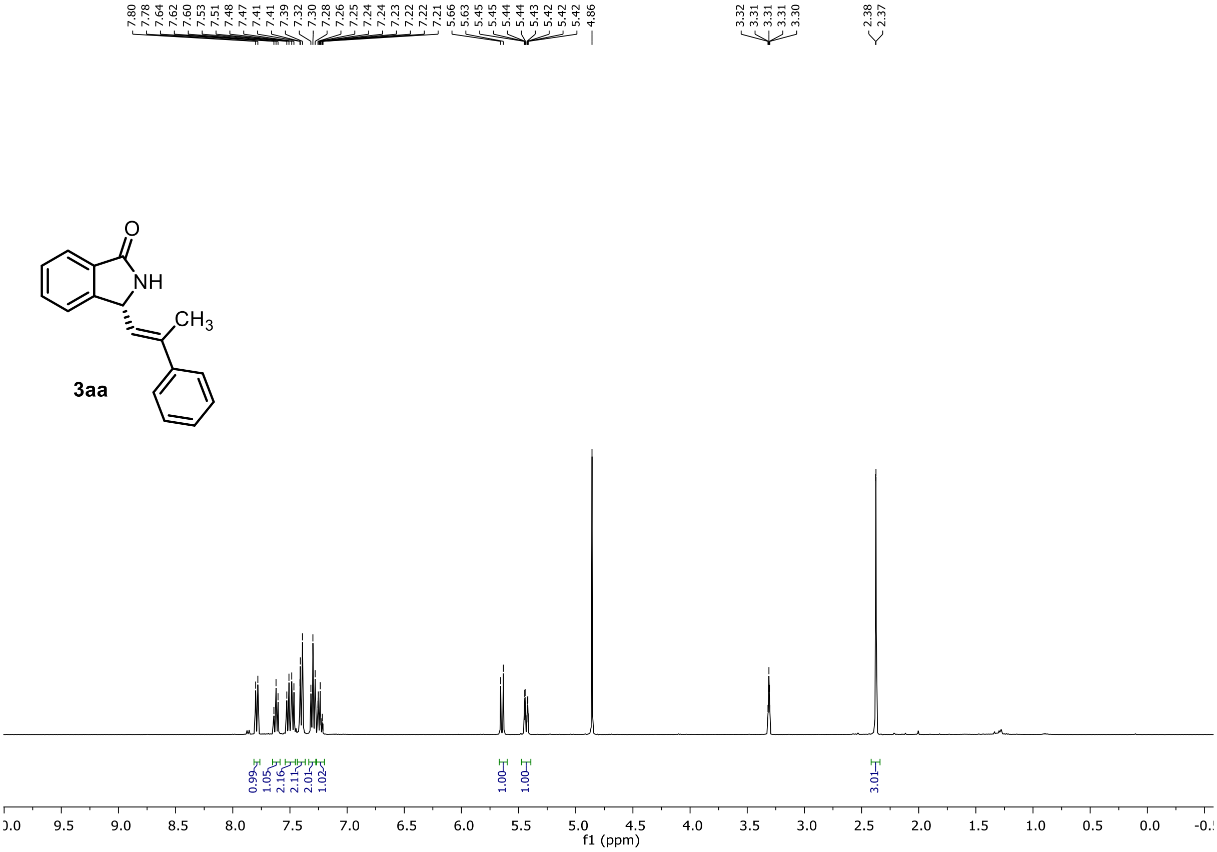

^{1}H NMR of **3aa** (400 MHz, Methanol-d$_4$)

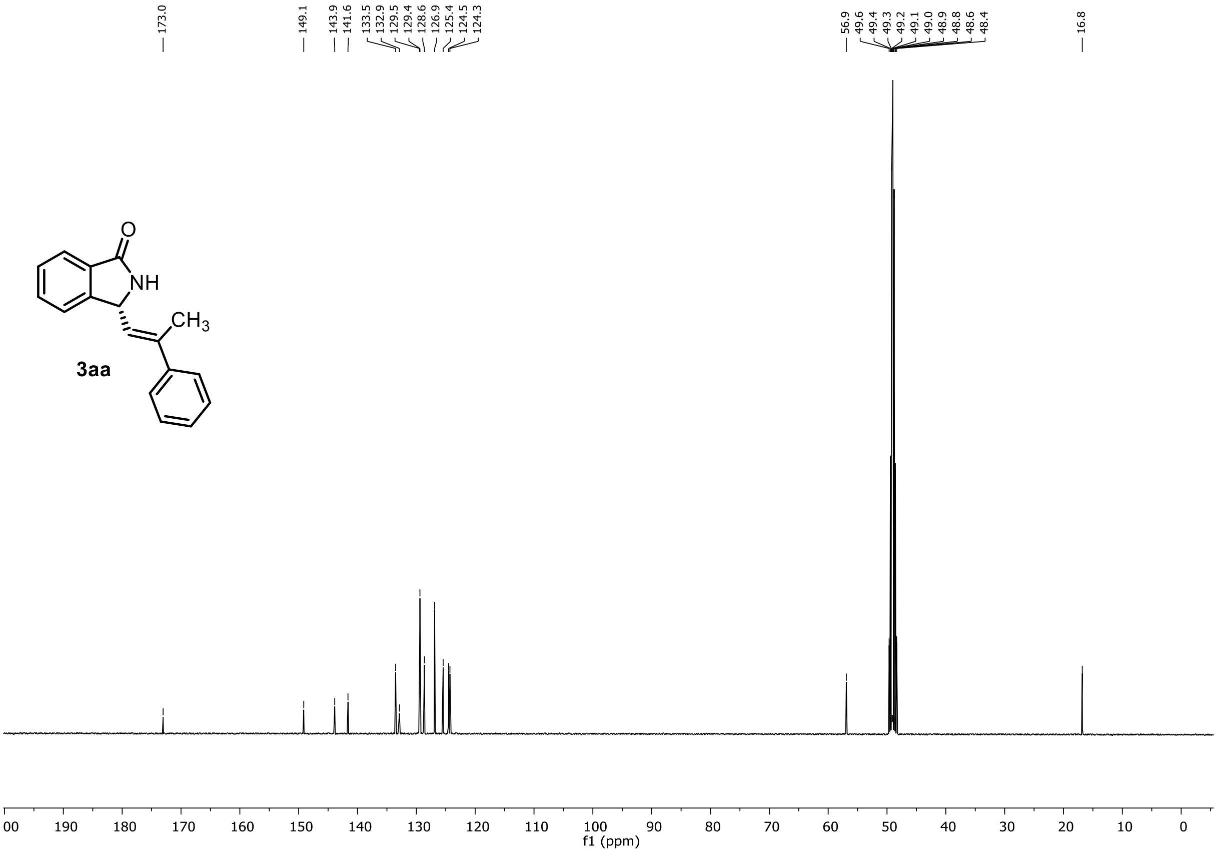

^{13}C NMR of **3aa** (101 MHz, Methanol-d$_4$)

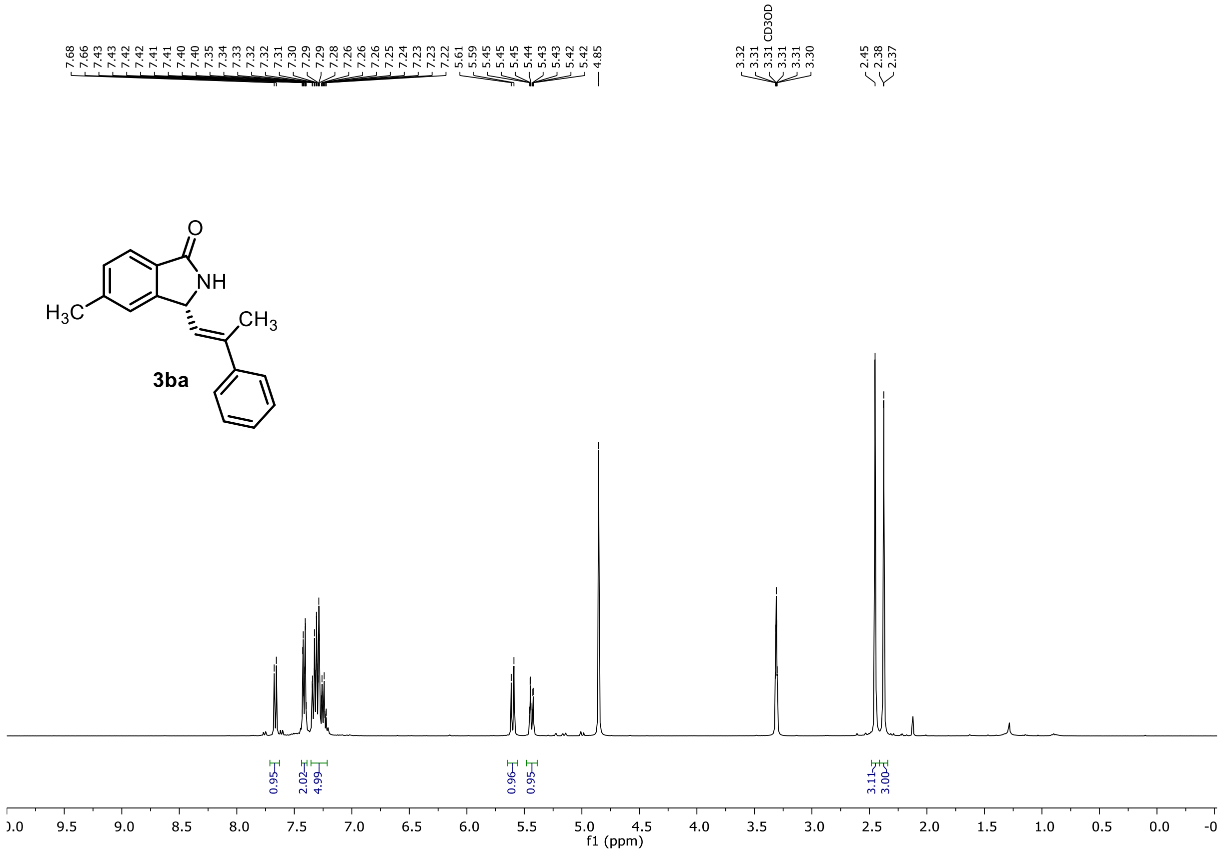

^{1}H NMR of **3ba** (400 MHz, Methanol-d$_4$)

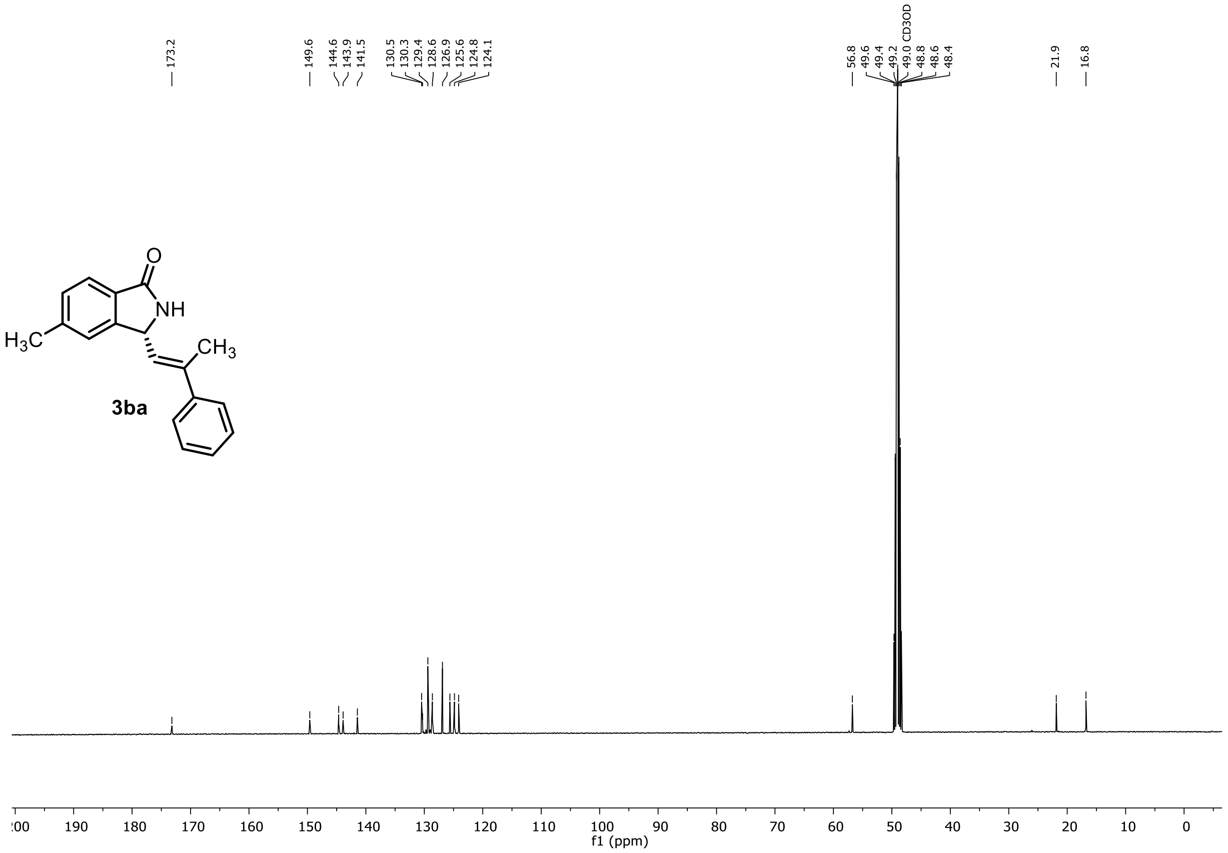

^{13}C NMR of **3ba** (101 MHz, Methanol-d$_4$)

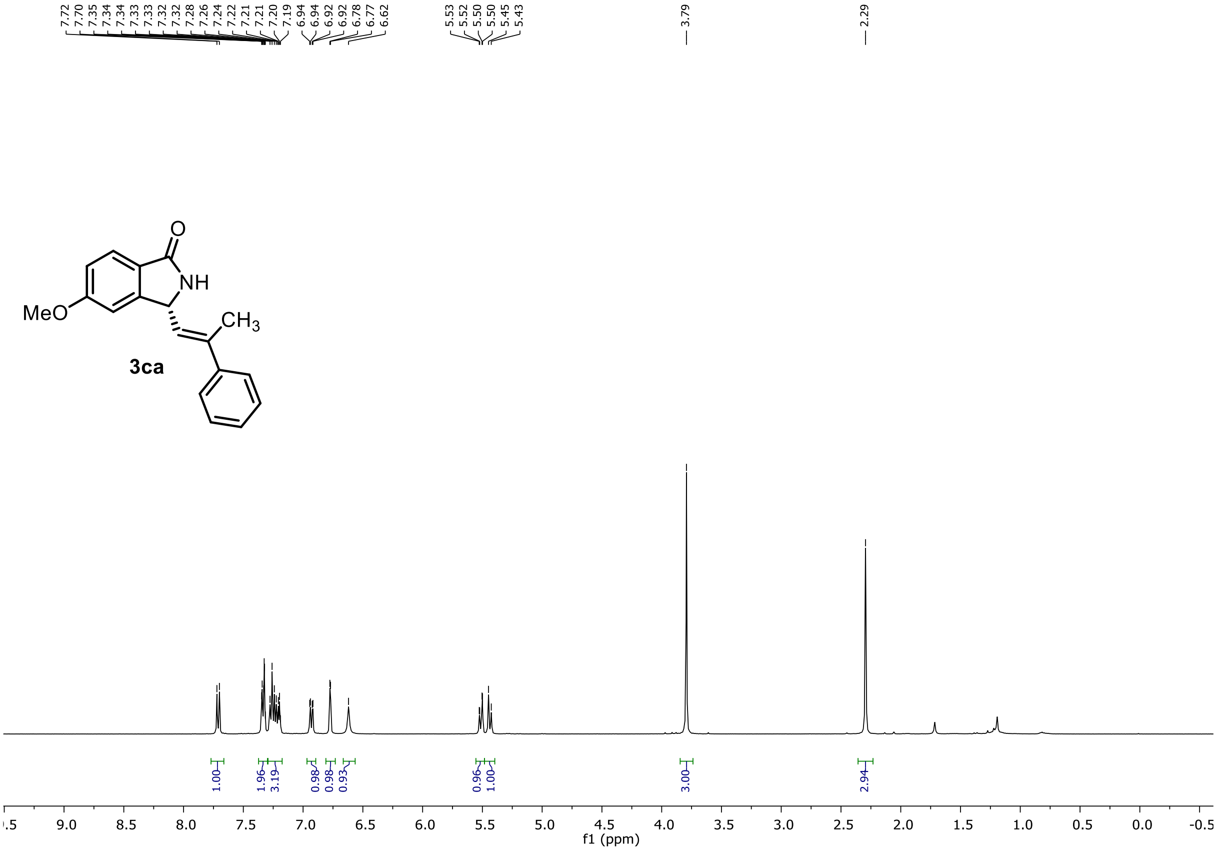

^{1}H NMR of **3ca** (400 MHz, $CDCl_3$)

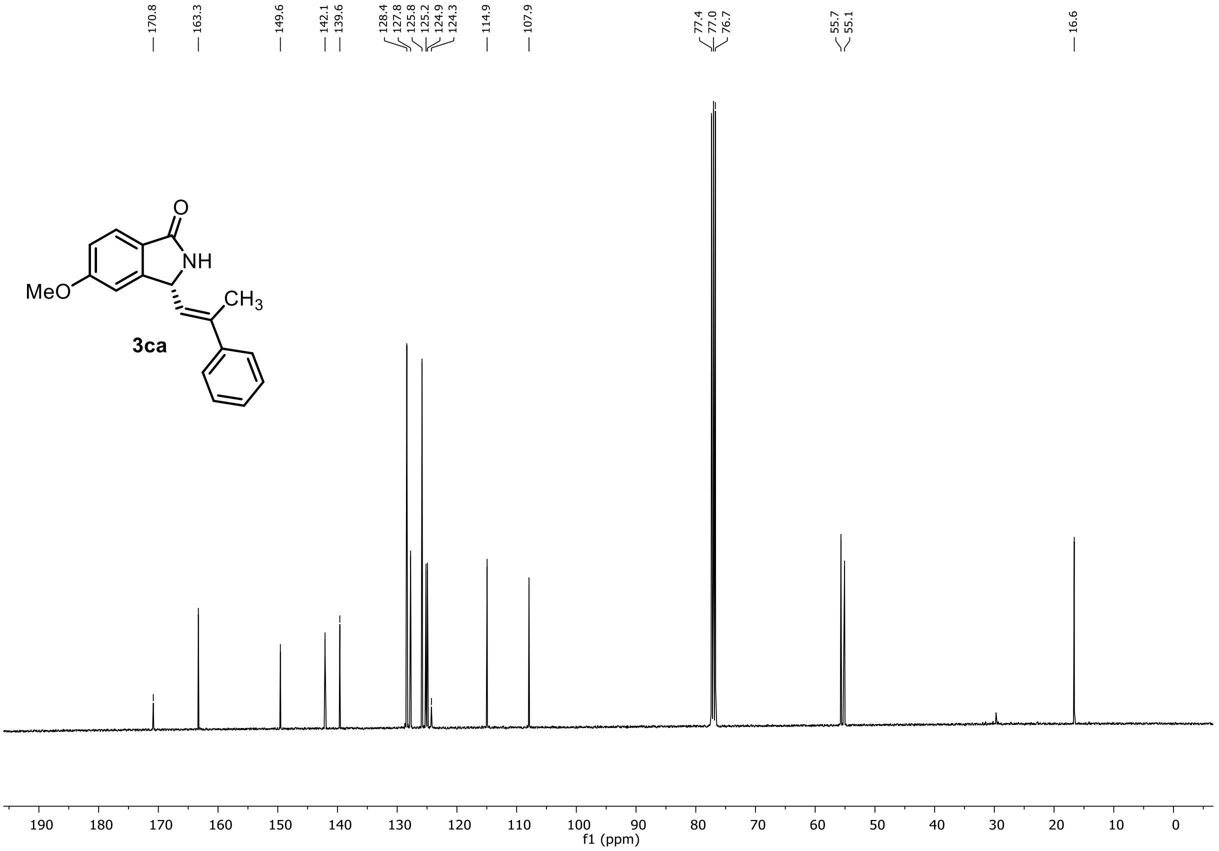

^{13}C NMR of **3ca** (101 MHz, $CDCl_3$)

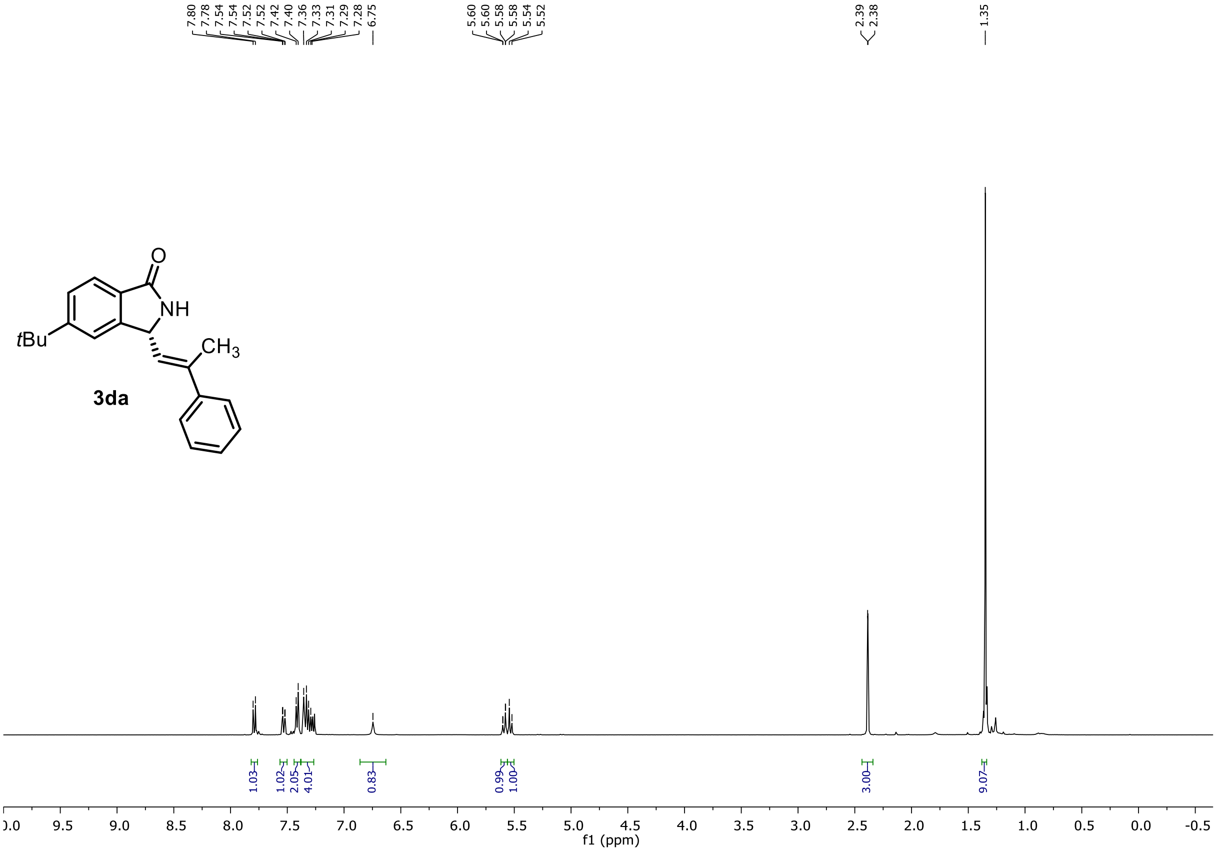

^{1}H NMR of **3da** (400 MHz, $CDCl_3$)

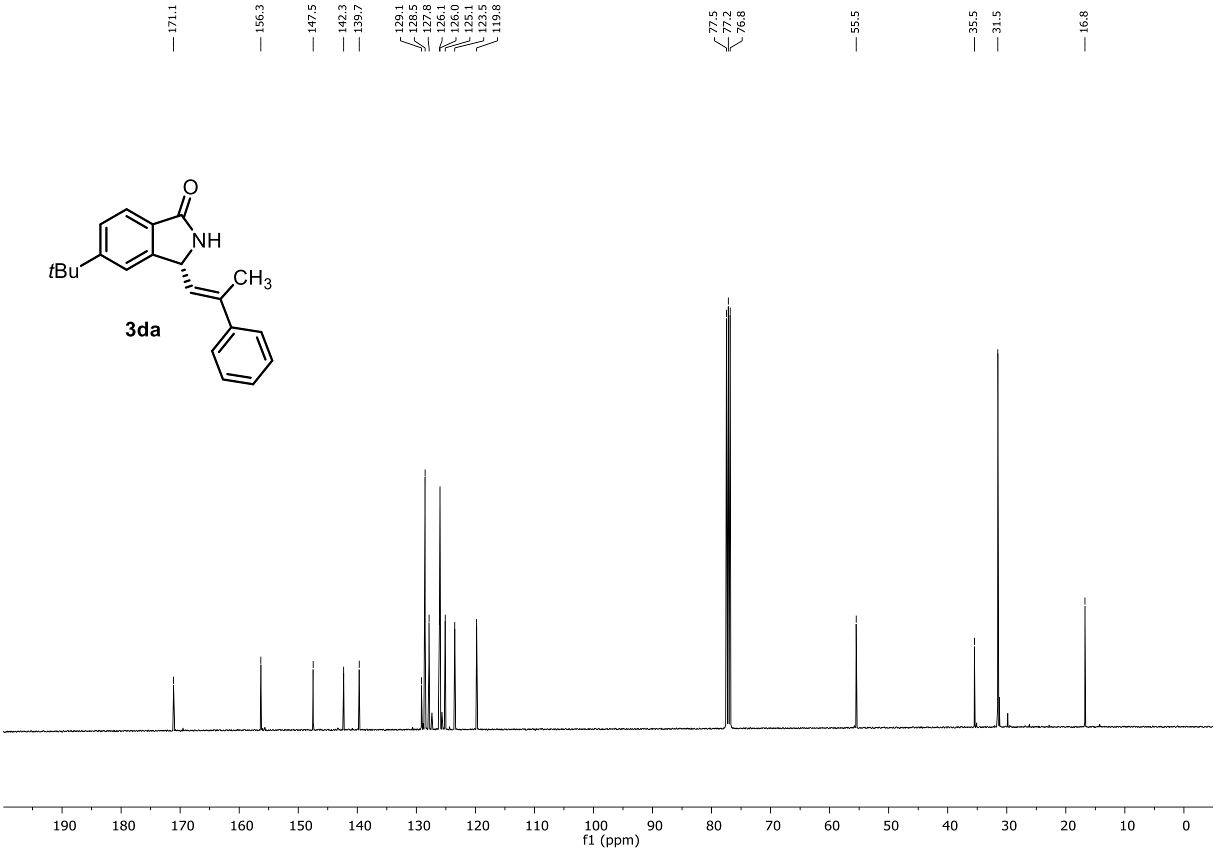

^{13}C NMR of **3da** (101 MHz, $CDCl_3$)

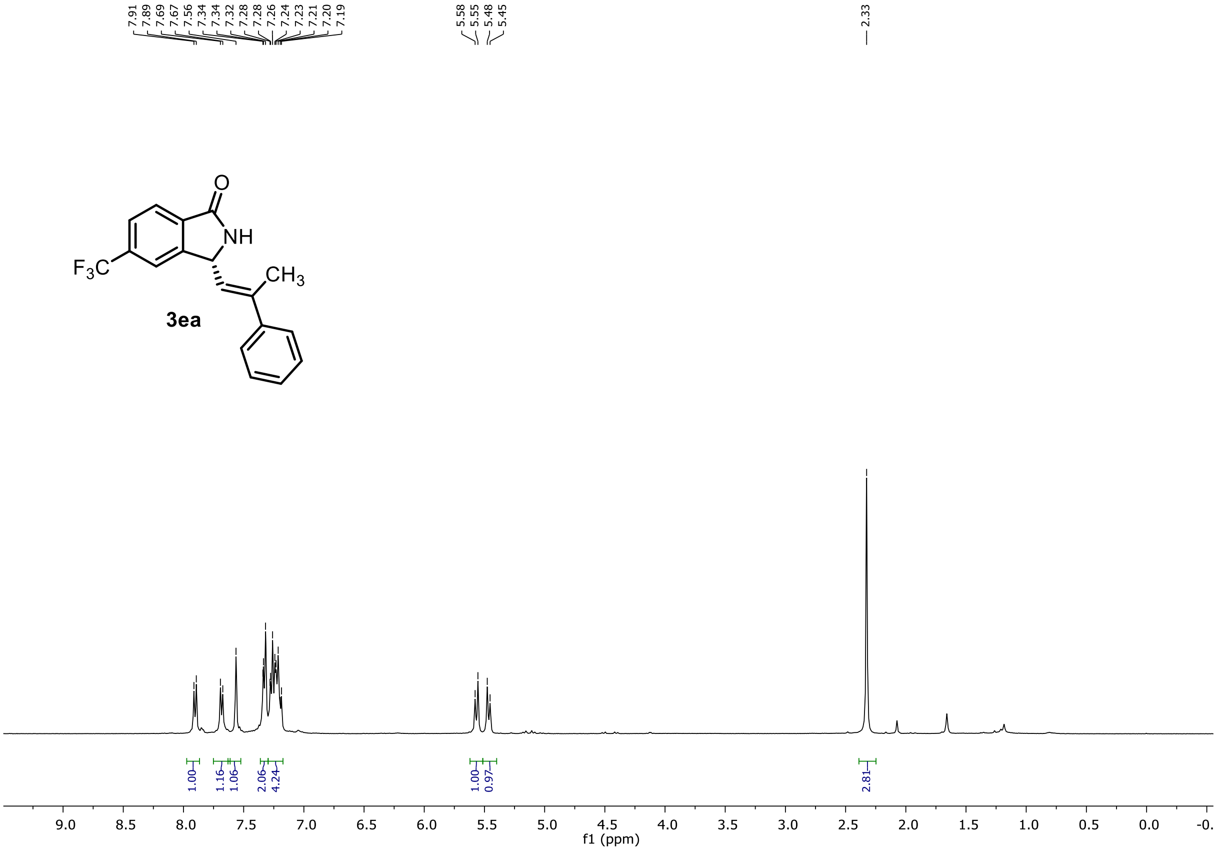

^{1}H NMR of **3ea** (400 MHz, $CDCl_3$)

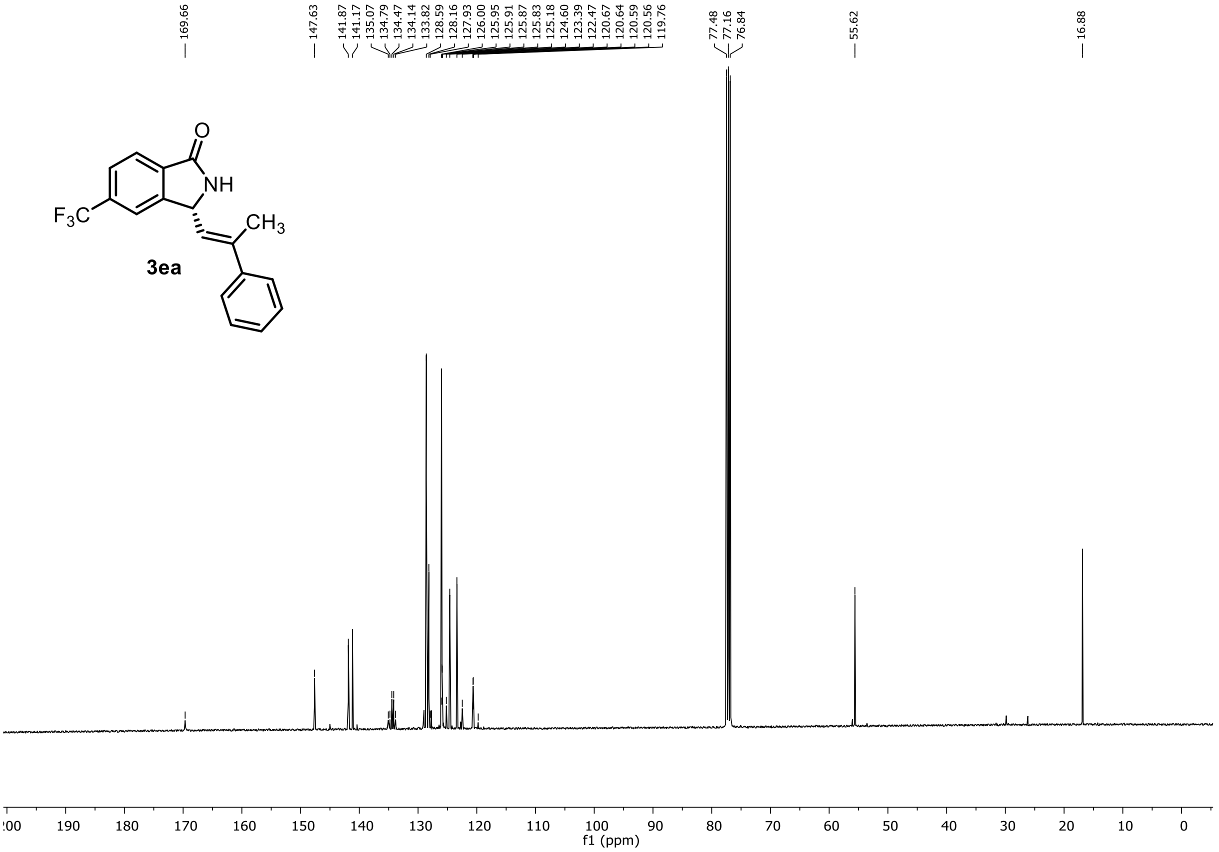

^{13}C NMR of **3ea** (101MHz, $CDCl_3$)

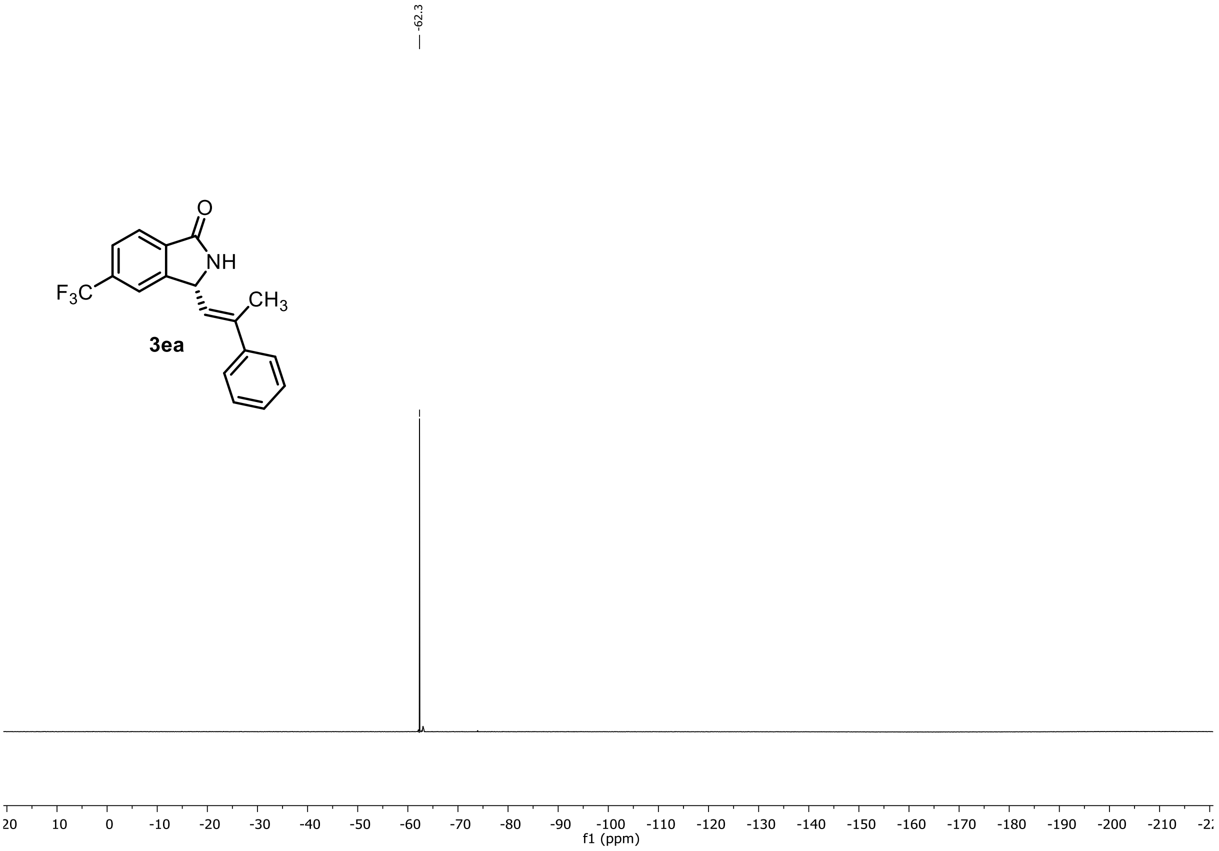

^{19}F NMR of **3ea** (376 MHz, $CDCl_3$)

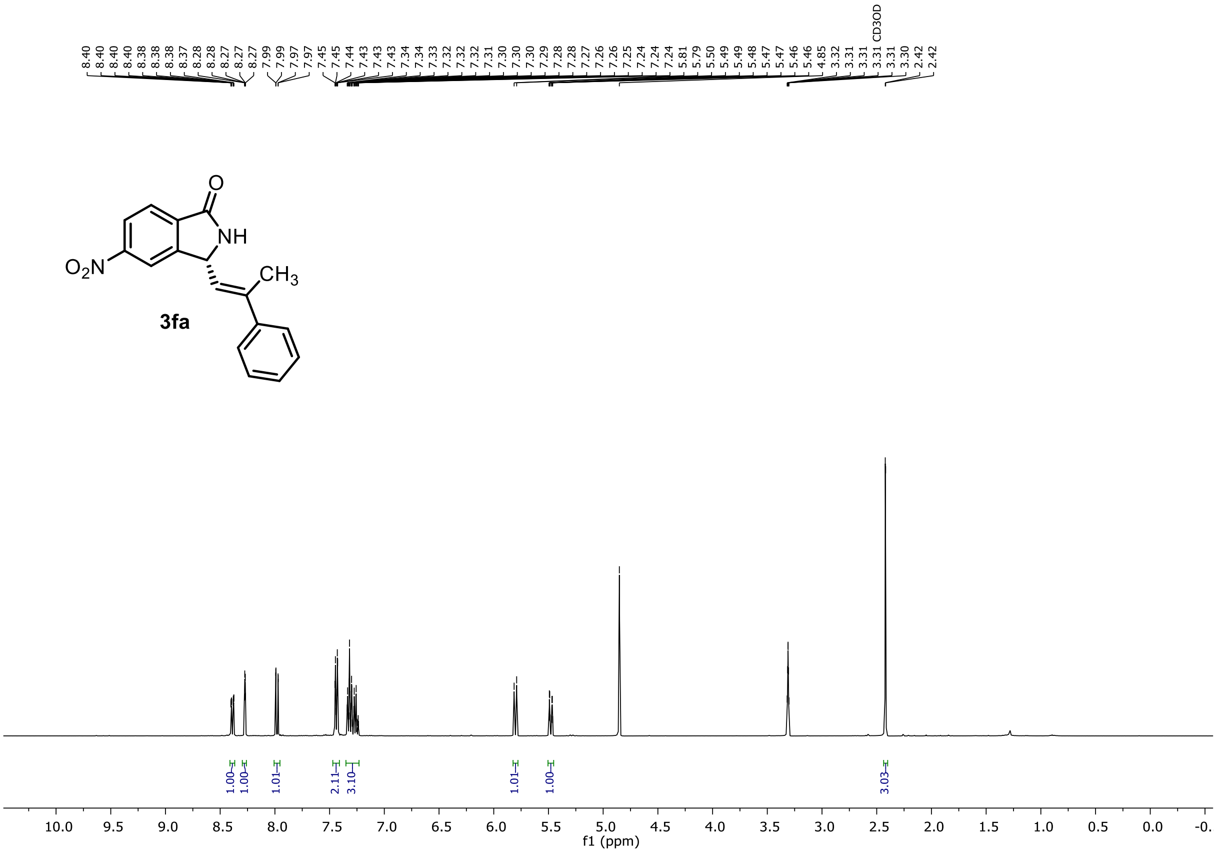

^{1}H NMR of **3fa** (400 MHz, Methanol-d_4)

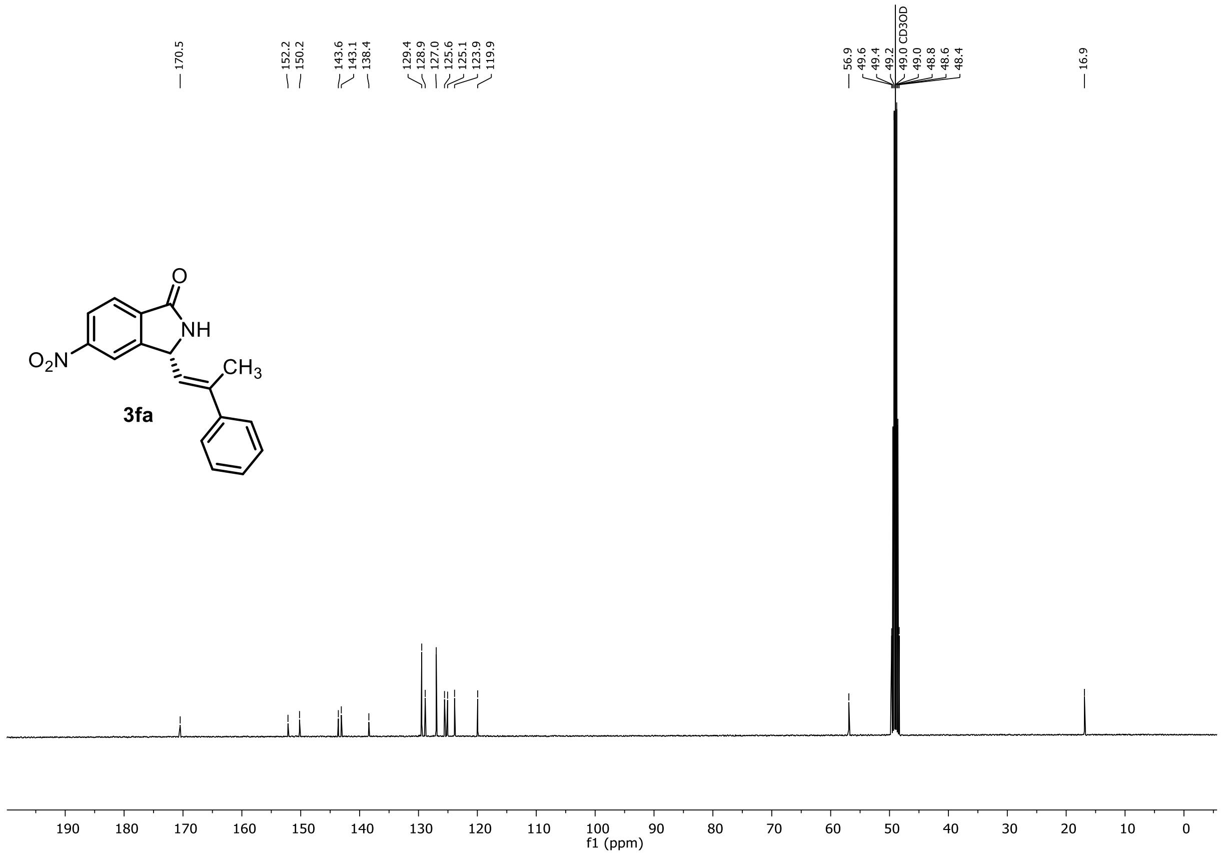

^{13}C NMR of **3fa** (101 MHz, Methanol-d$_4$)

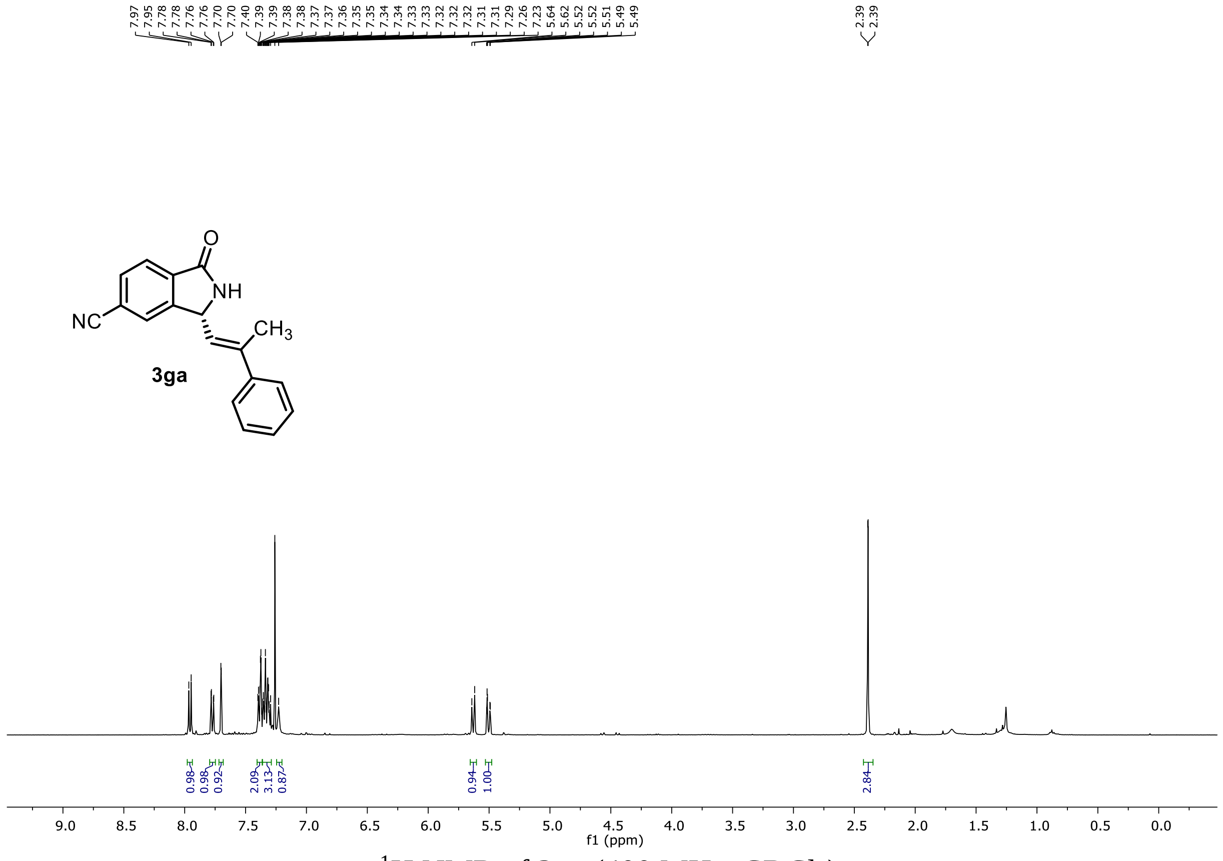

^{1}H NMR of **3ga** (400 MHz, $CDCl_3$)

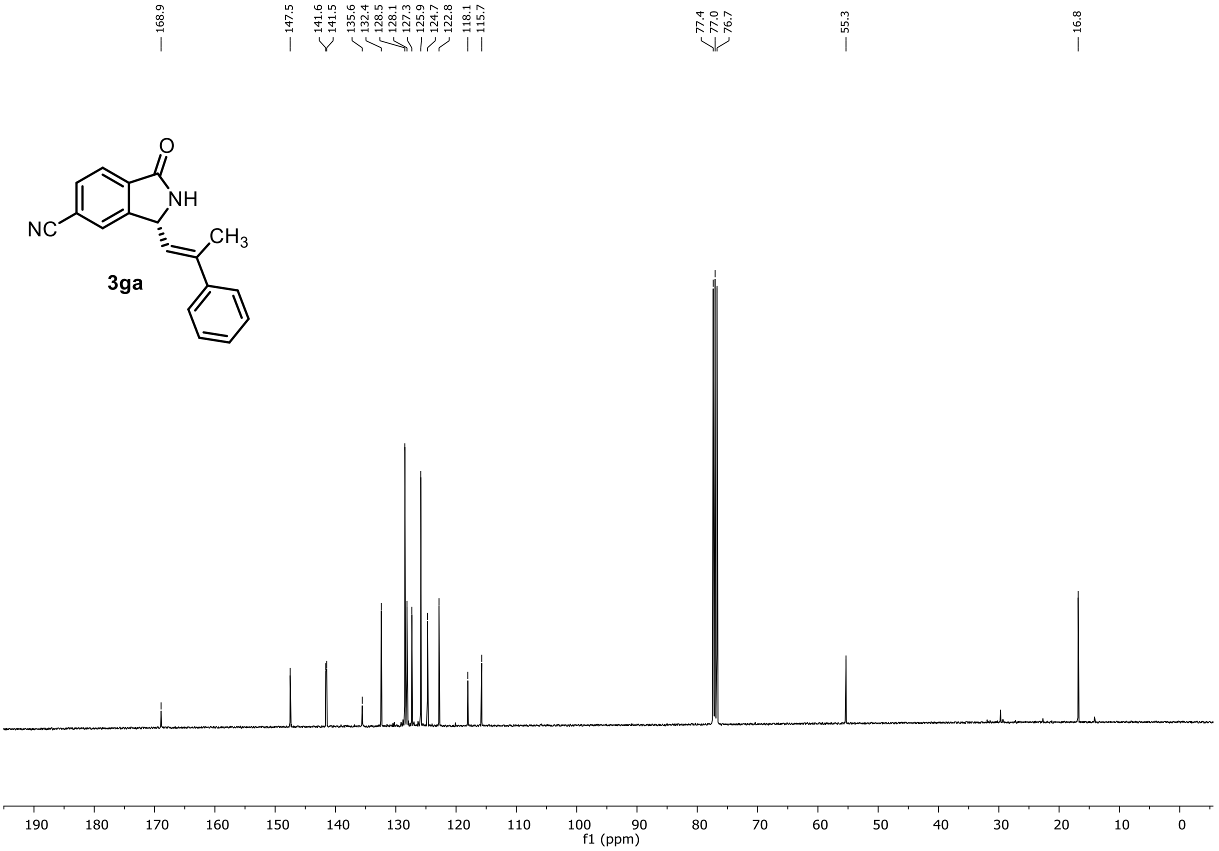

^{13}C NMR of **3ga** (101 MHz, $CDCl_3$)

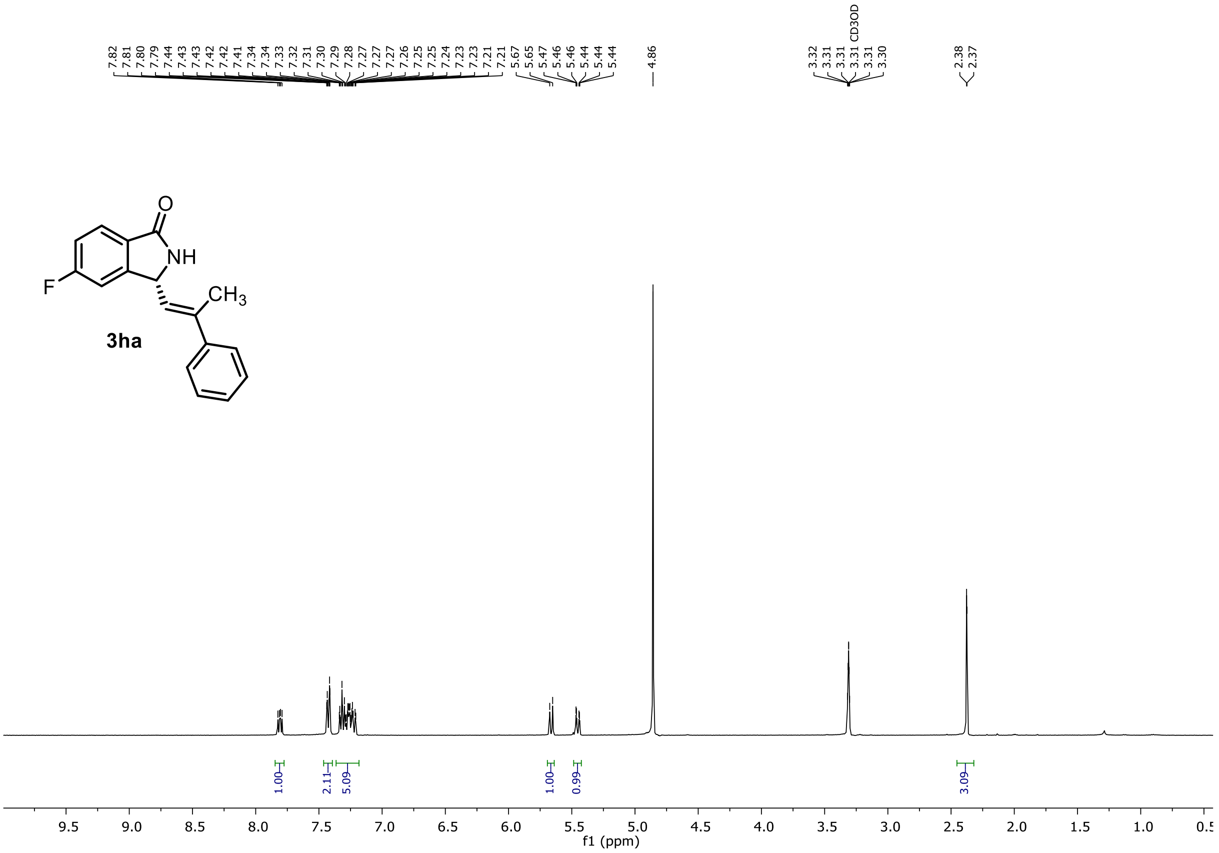

^{1}H NMR of **3ha** (400 MHz, Methanol-d_4)

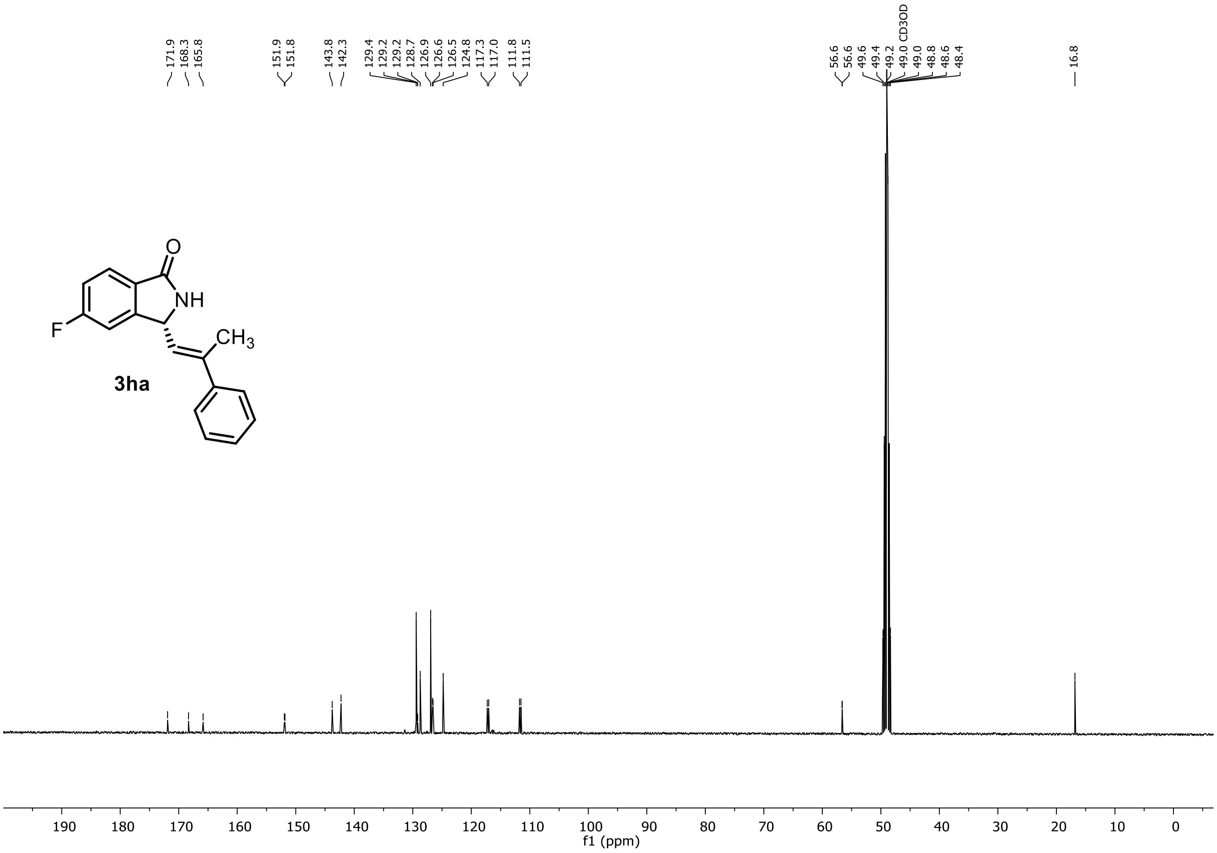

^{13}C NMR of **3ha** (101 MHz, Methanol-d$_4$)

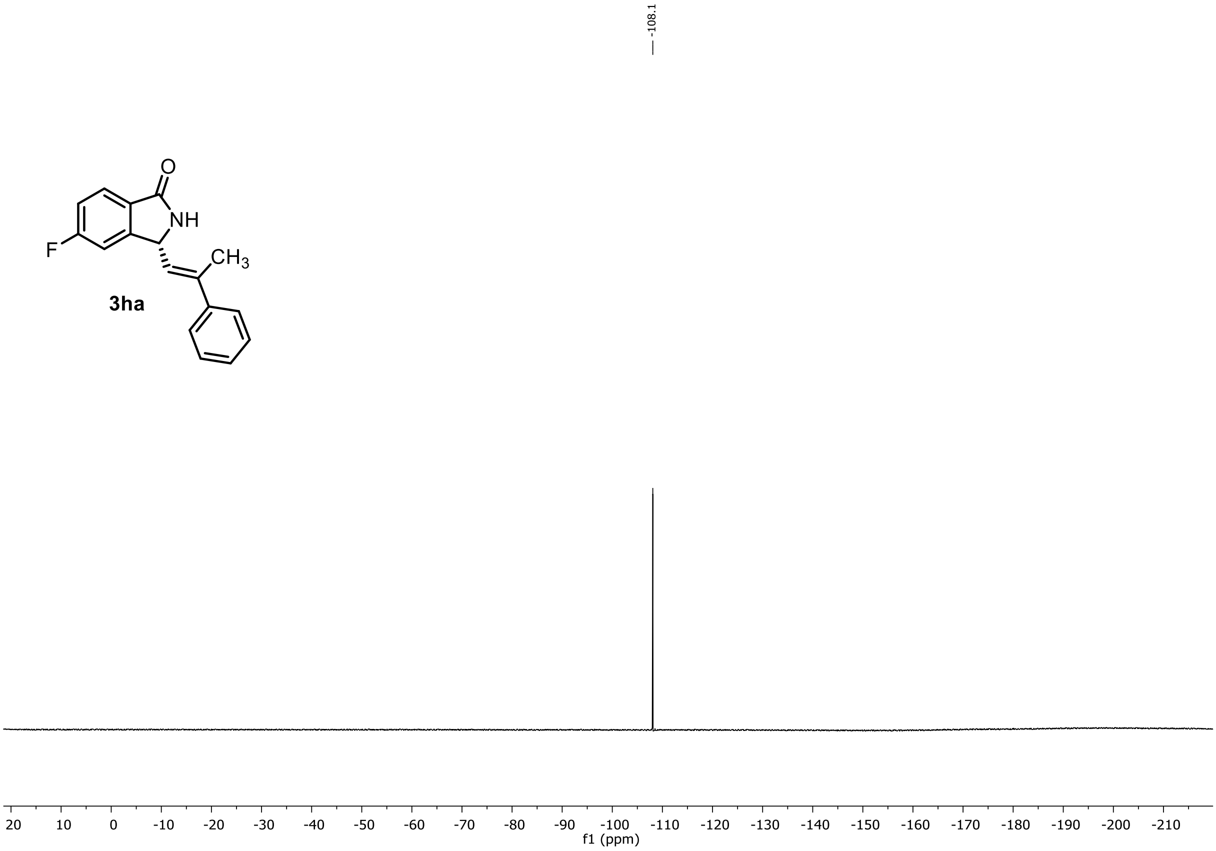

^{19}F NMR of **3ha** (376 MHz, Methanol-d$_4$)

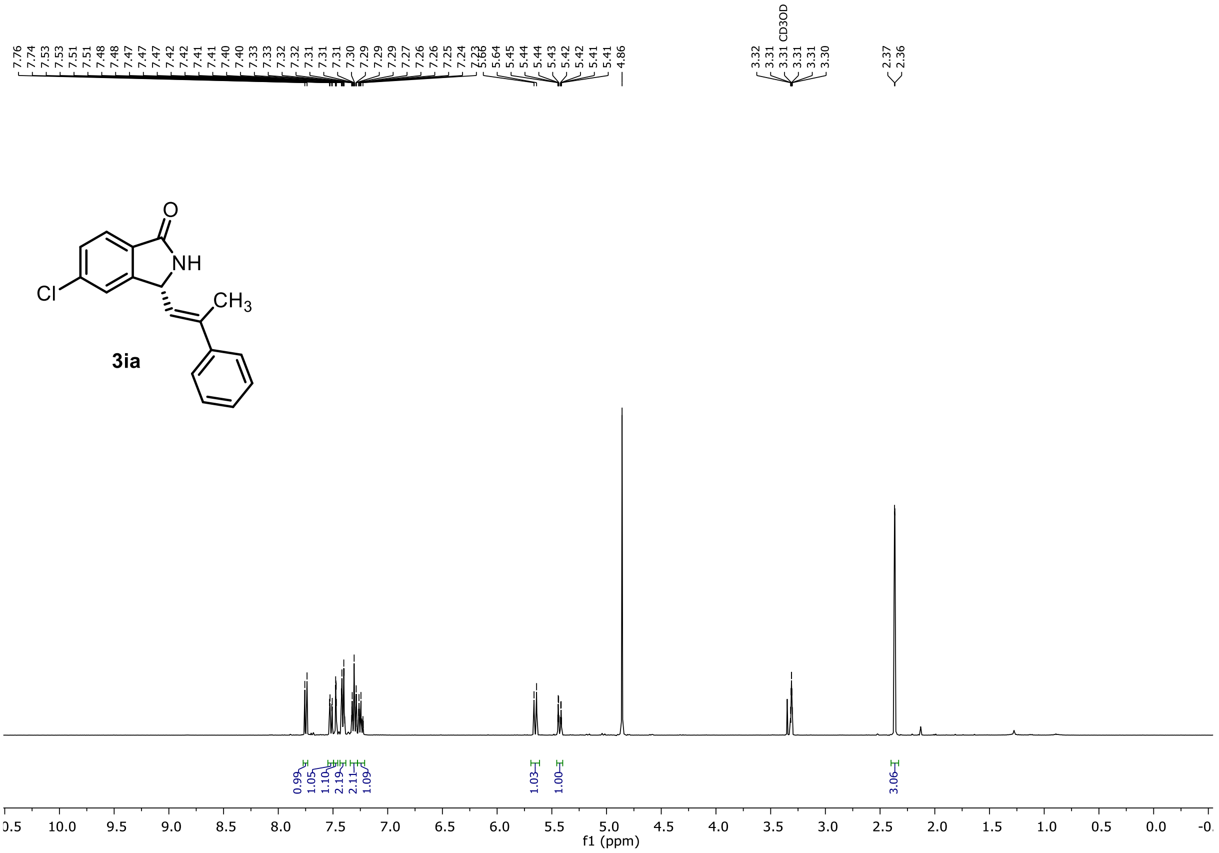

^{1}H NMR of **3ia** (400 MHz, Methanol-d_4)

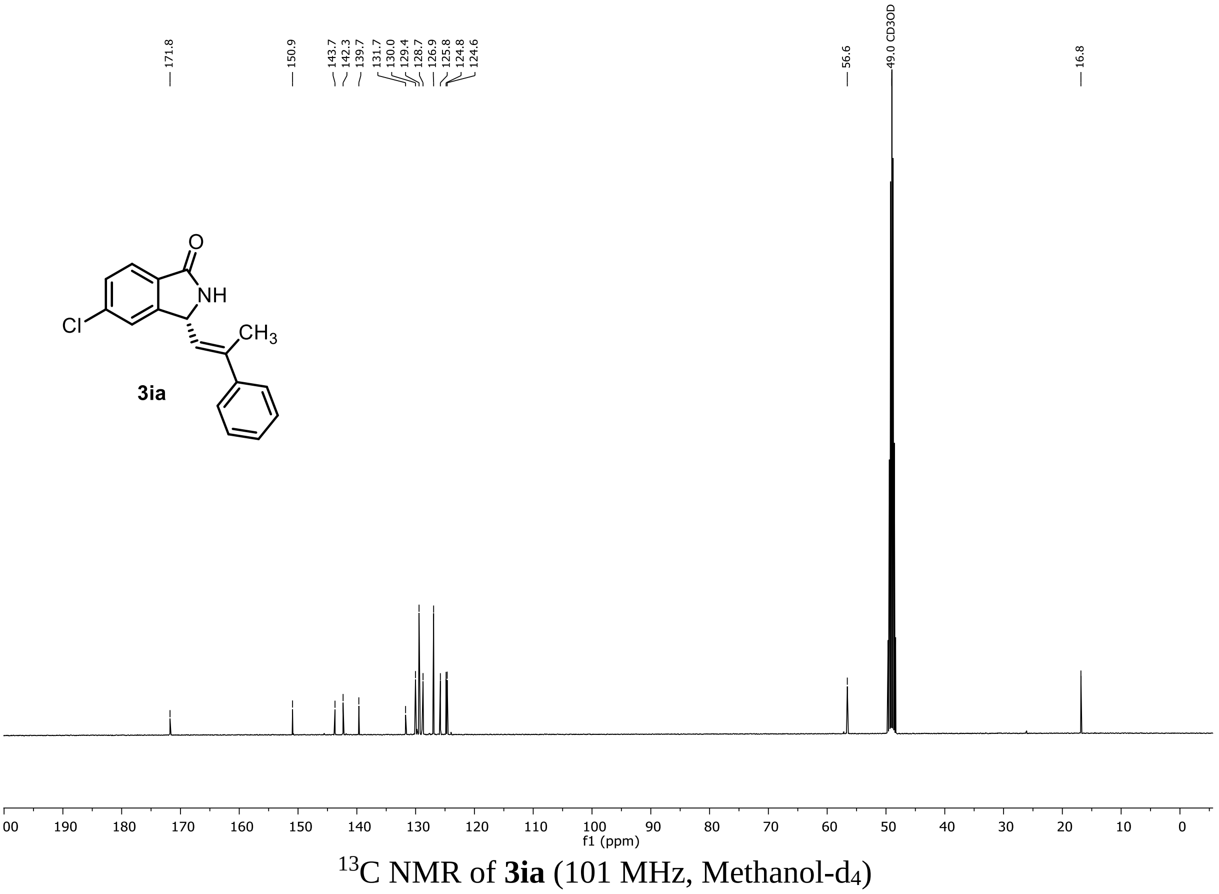

^{13}C NMR of **3ia** (101 MHz, Methanol-d_4)

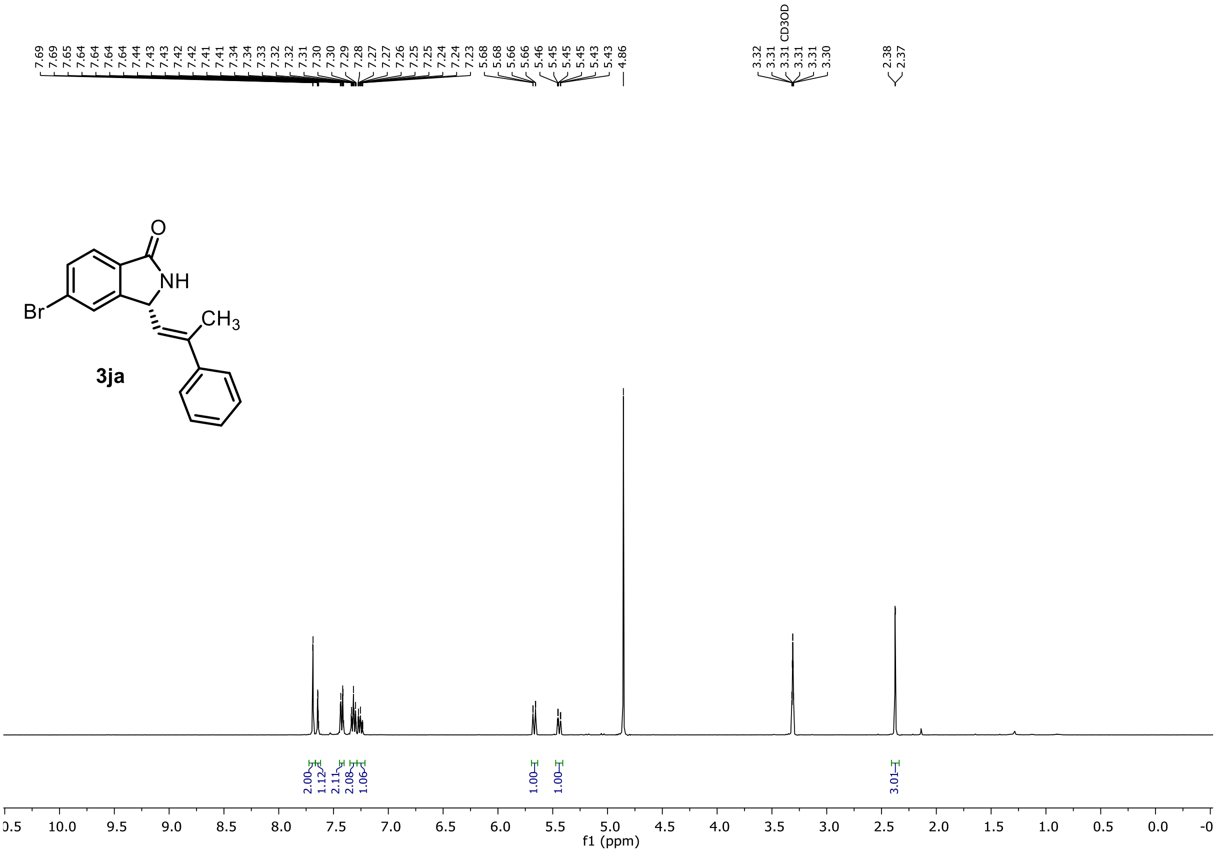

^{1}H NMR of **3ja** (400 MHz, Methanol-d$_4$)

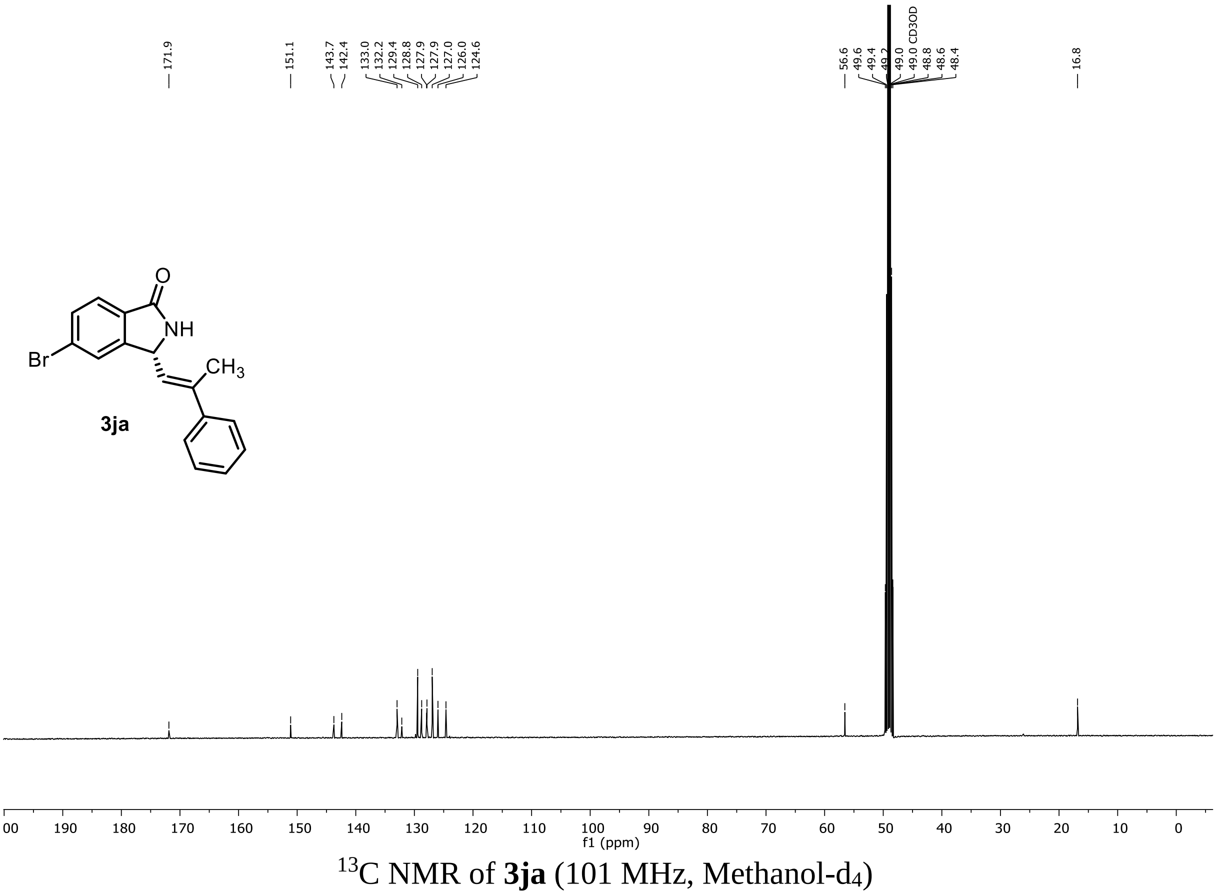

^{13}C NMR of **3ja** (101 MHz, Methanol-d$_4$)

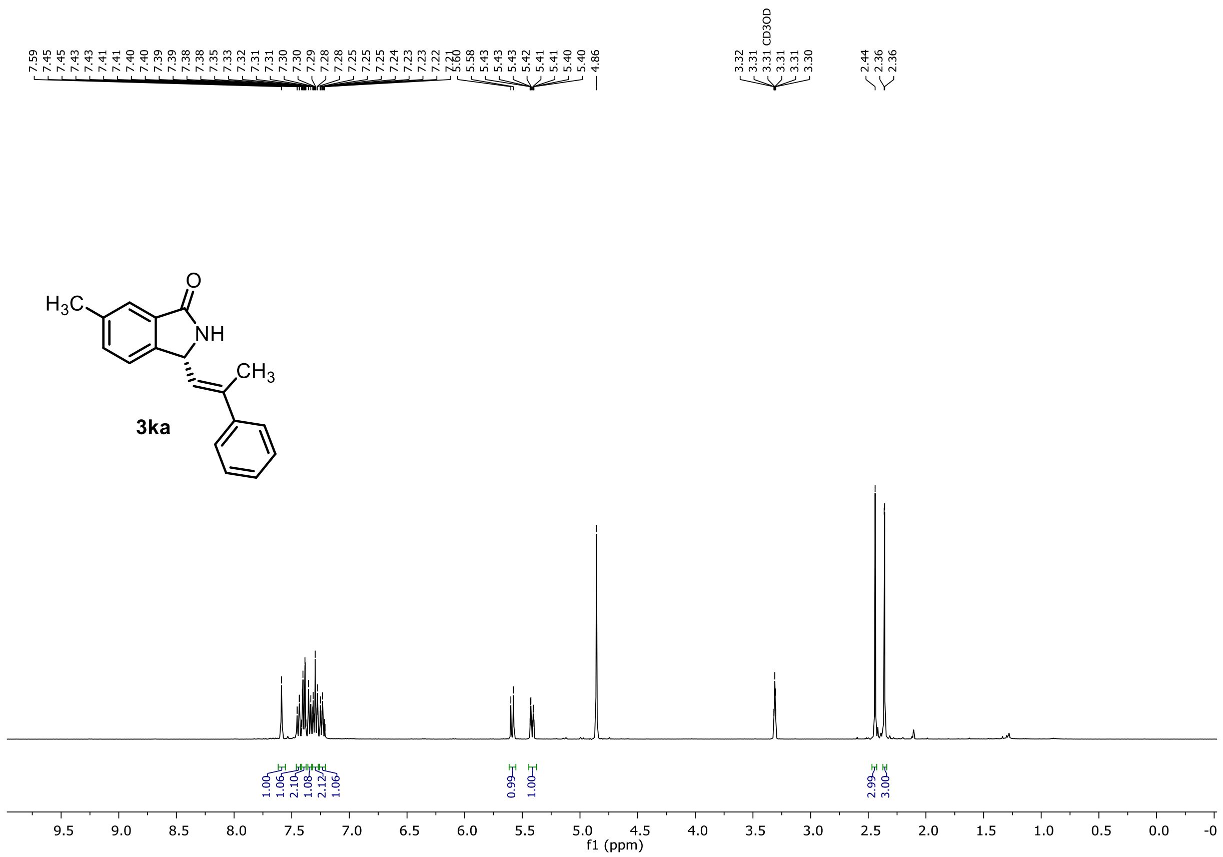

^{1}H NMR of **3ka** (400 MHz, Methanol-d$_4$)

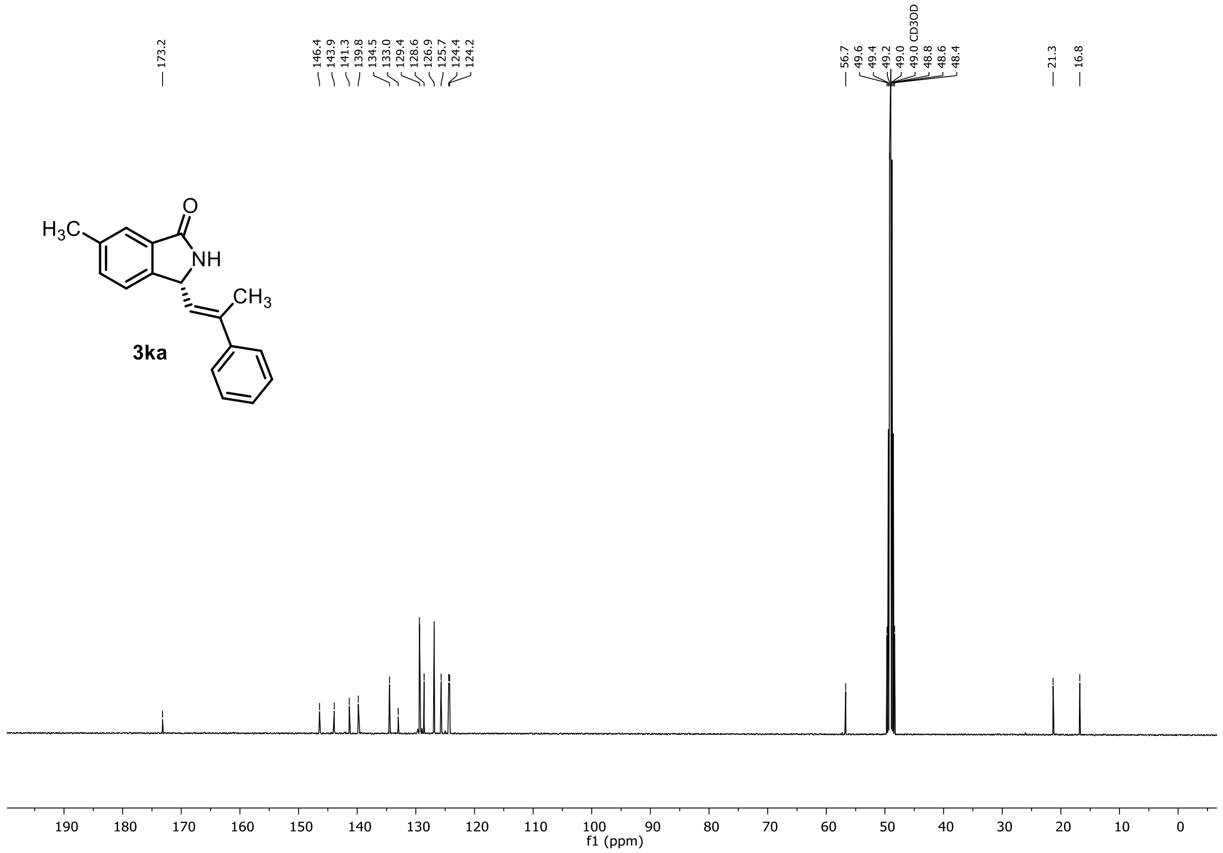

^{13}C NMR of **3ka** (101 MHz, Methanol-d$_4$)

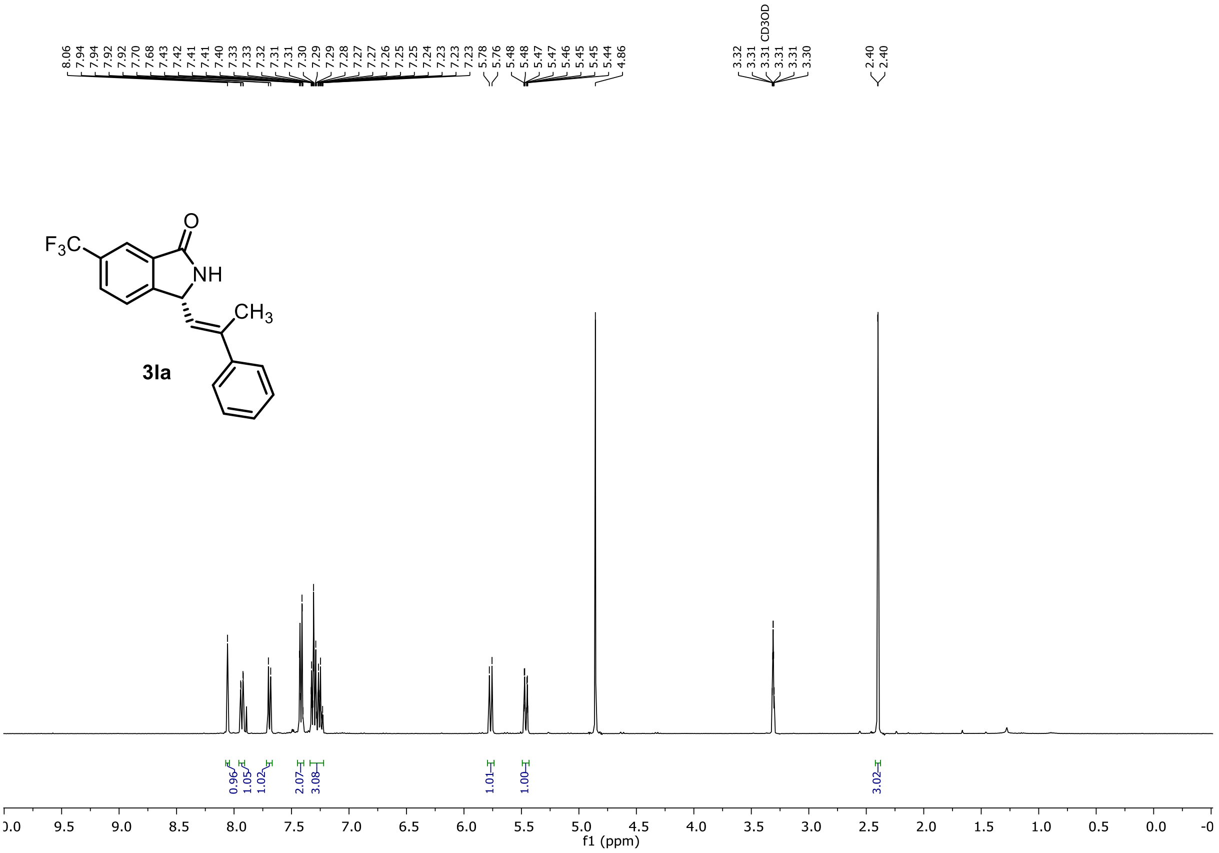

^{1}H NMR of **3la** (400 MHz, Methanol-d$_4$)

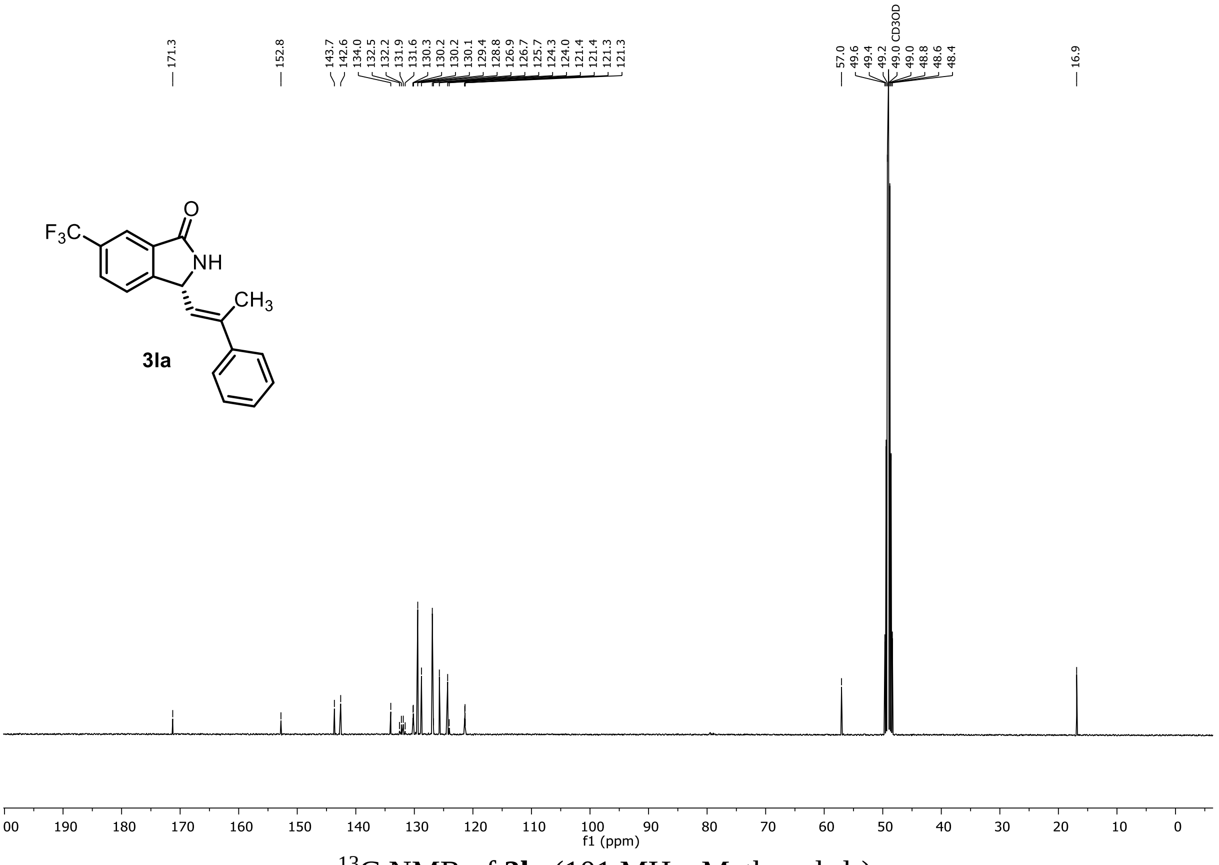

^{13}C NMR of **3la** (101 MHz, Methanol-d$_4$)

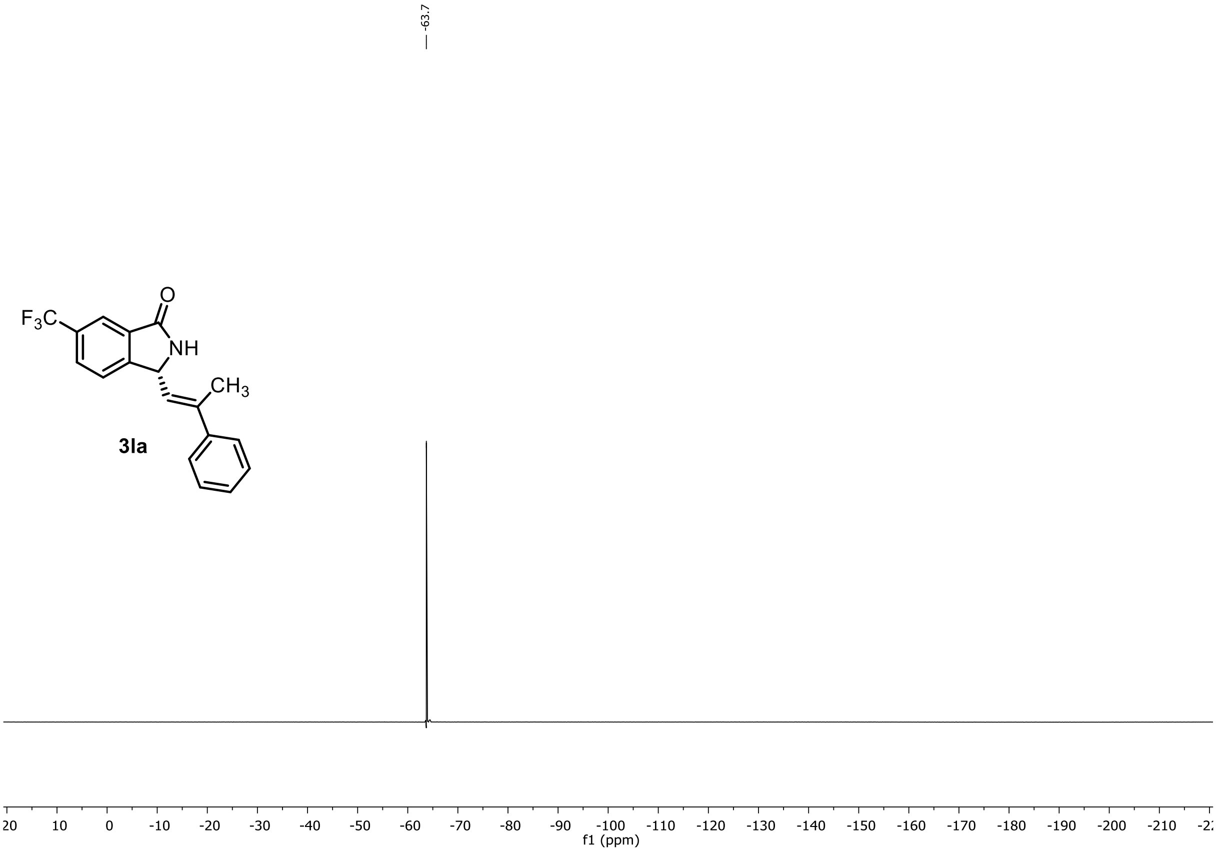

^{19}F NMR of **3la** (376 MHz, Methanol-d$_4$)

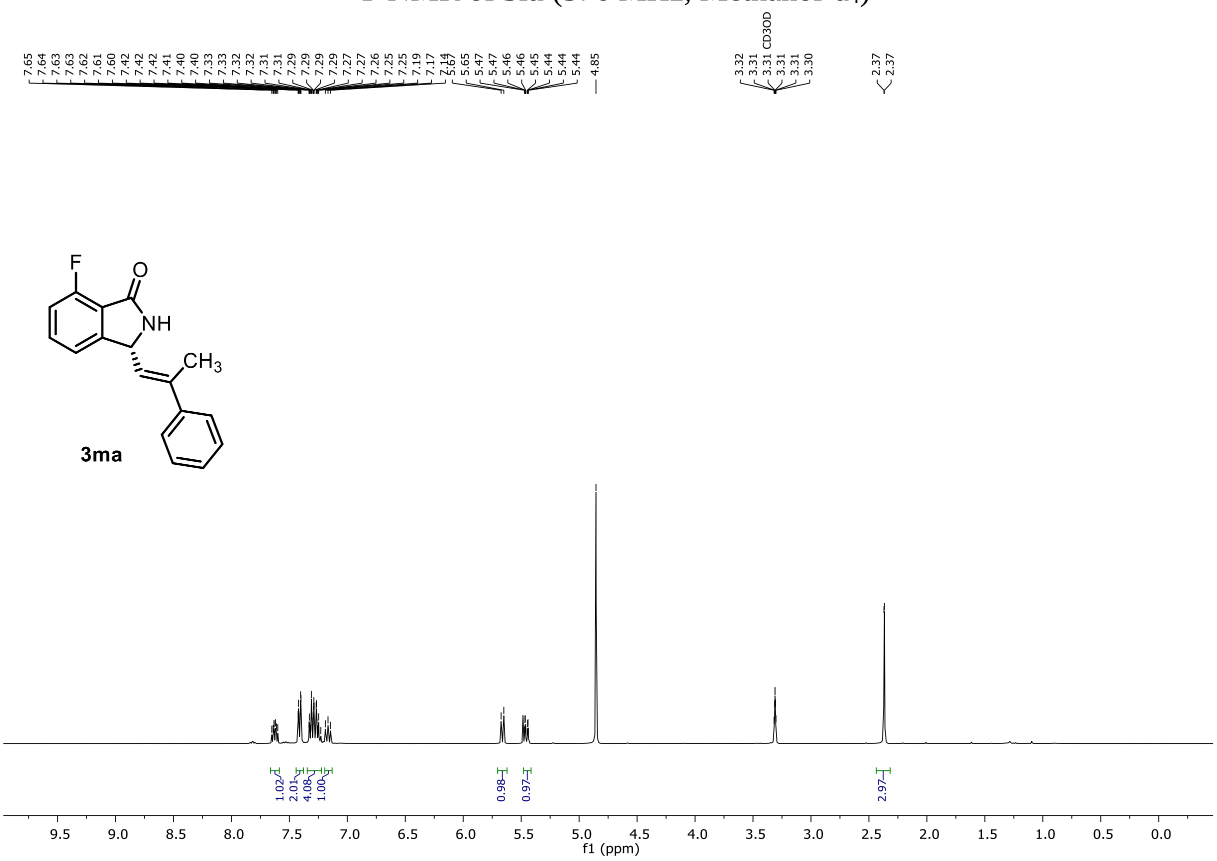

^{1}H NMR of **3ma** (400 MHz, Methanol-d$_4$)

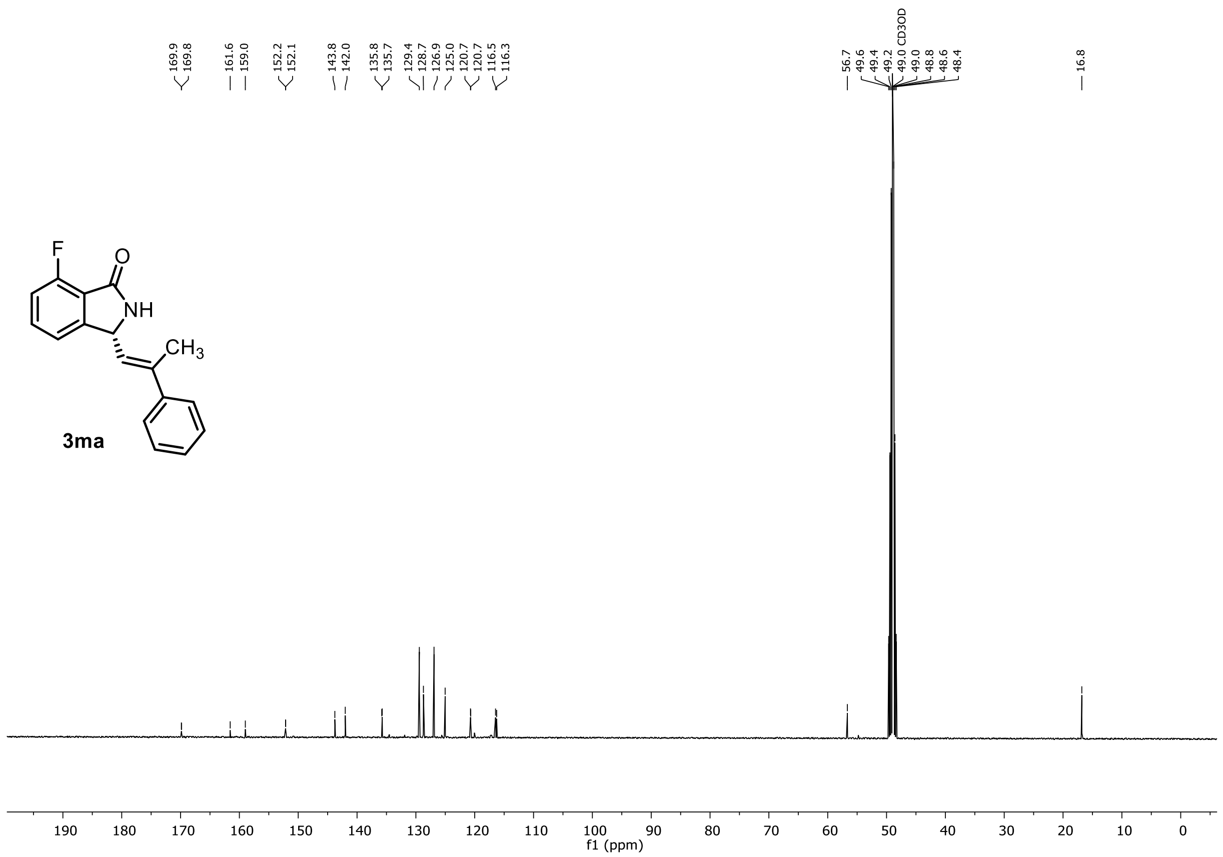

^{13}C NMR of **3ma** (101 MHz, Methanol-D_4)

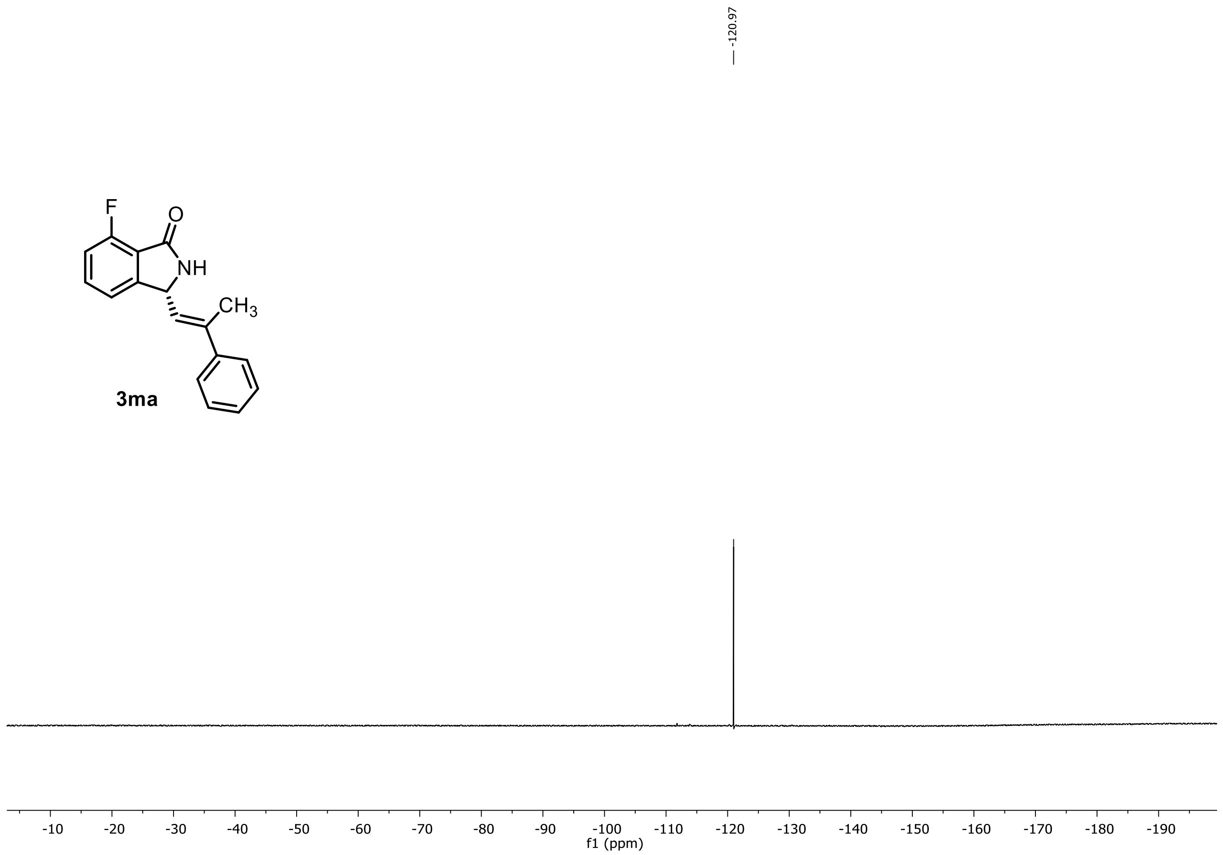

^{19}F NMR of **3ma** (376 MHz, Methanol-D_4)

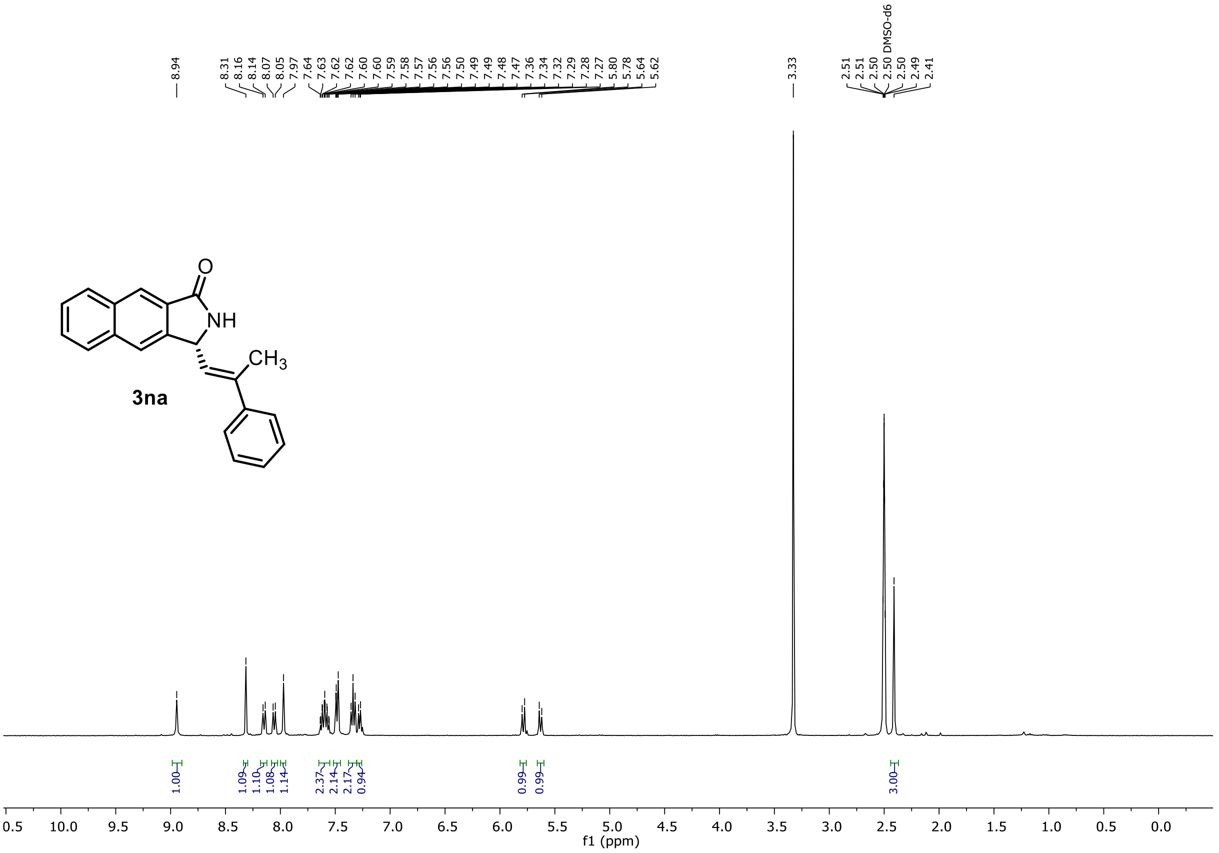

^{1}H NMR of **3na** (400 MHz, DMSO-d_6)

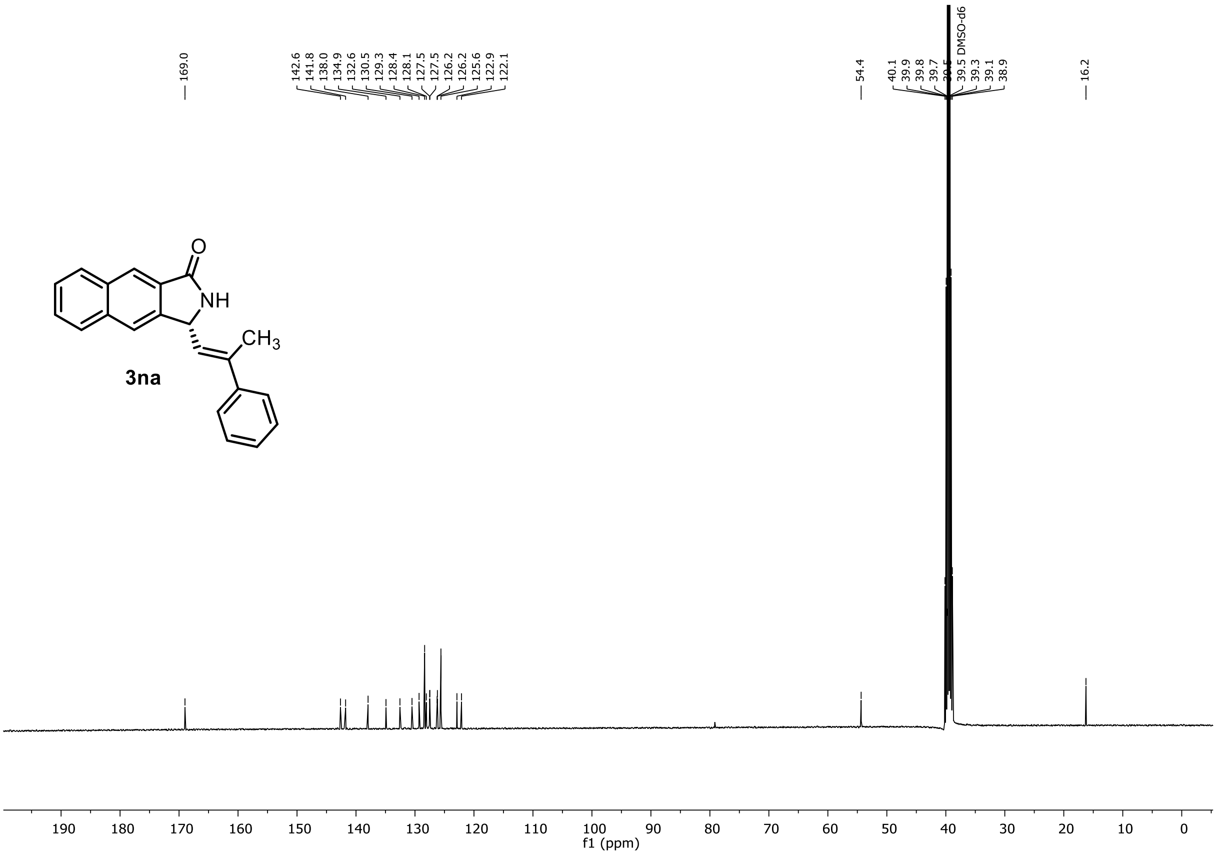

^{13}C NMR of **3na** (101 MHz, DMSO-d_6)

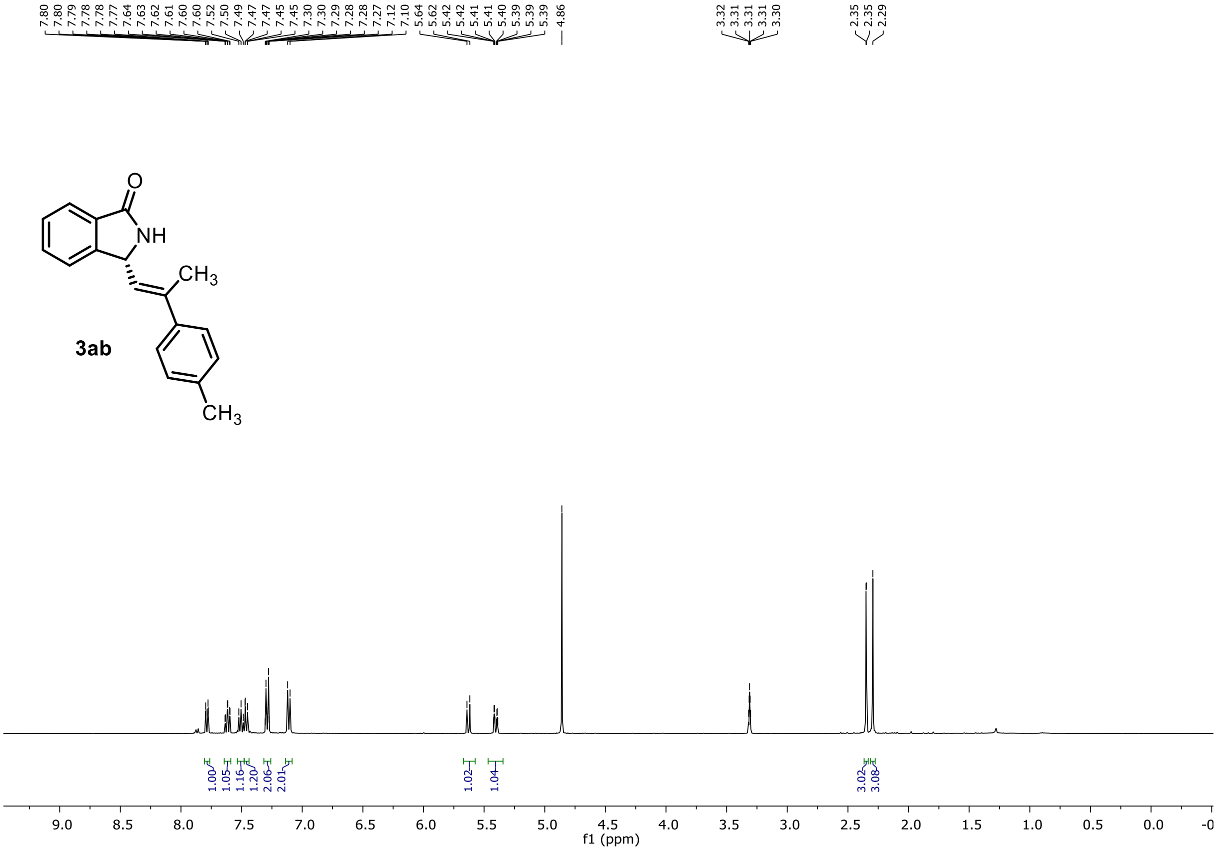

^{1}H NMR of **3ab** (400 MHz, Methanol-d$_4$)

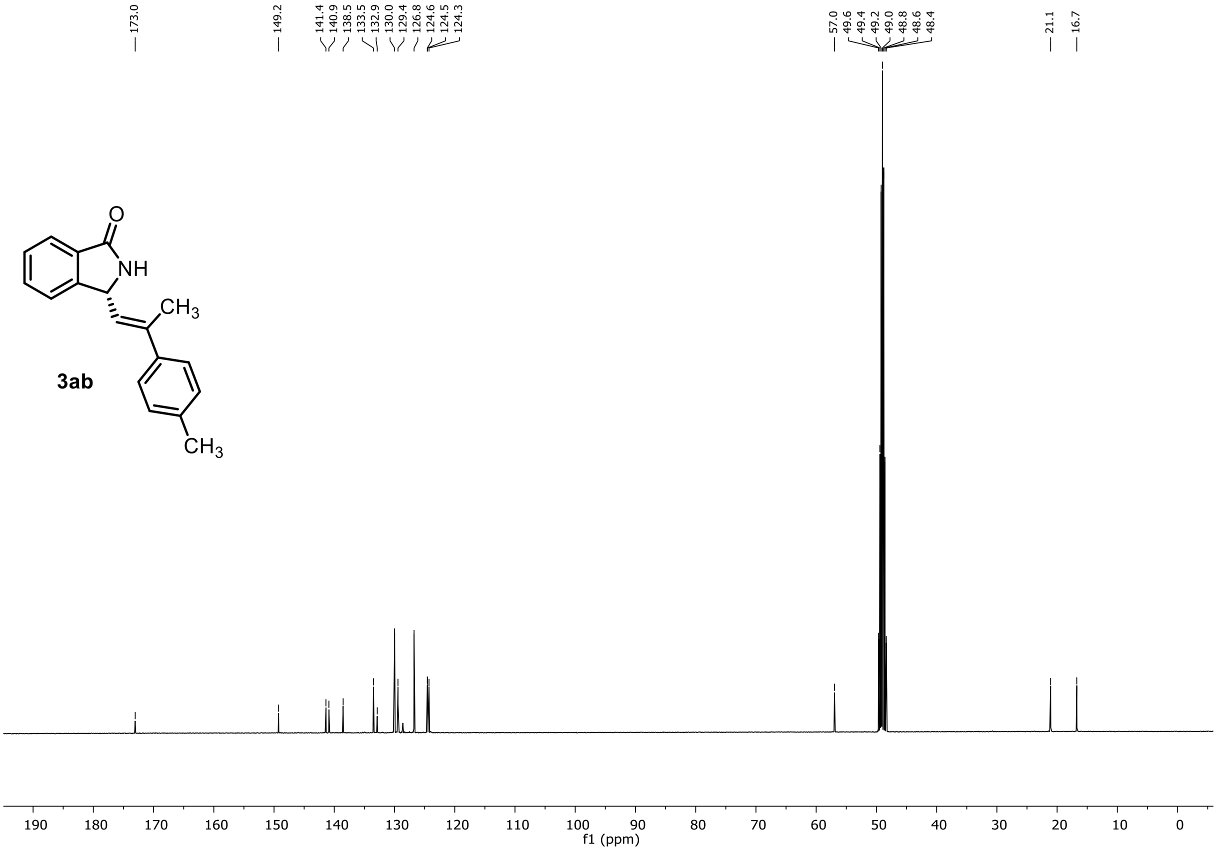

^{13}C NMR of **3ab** (101 MHz, Methanol-d$_4$)

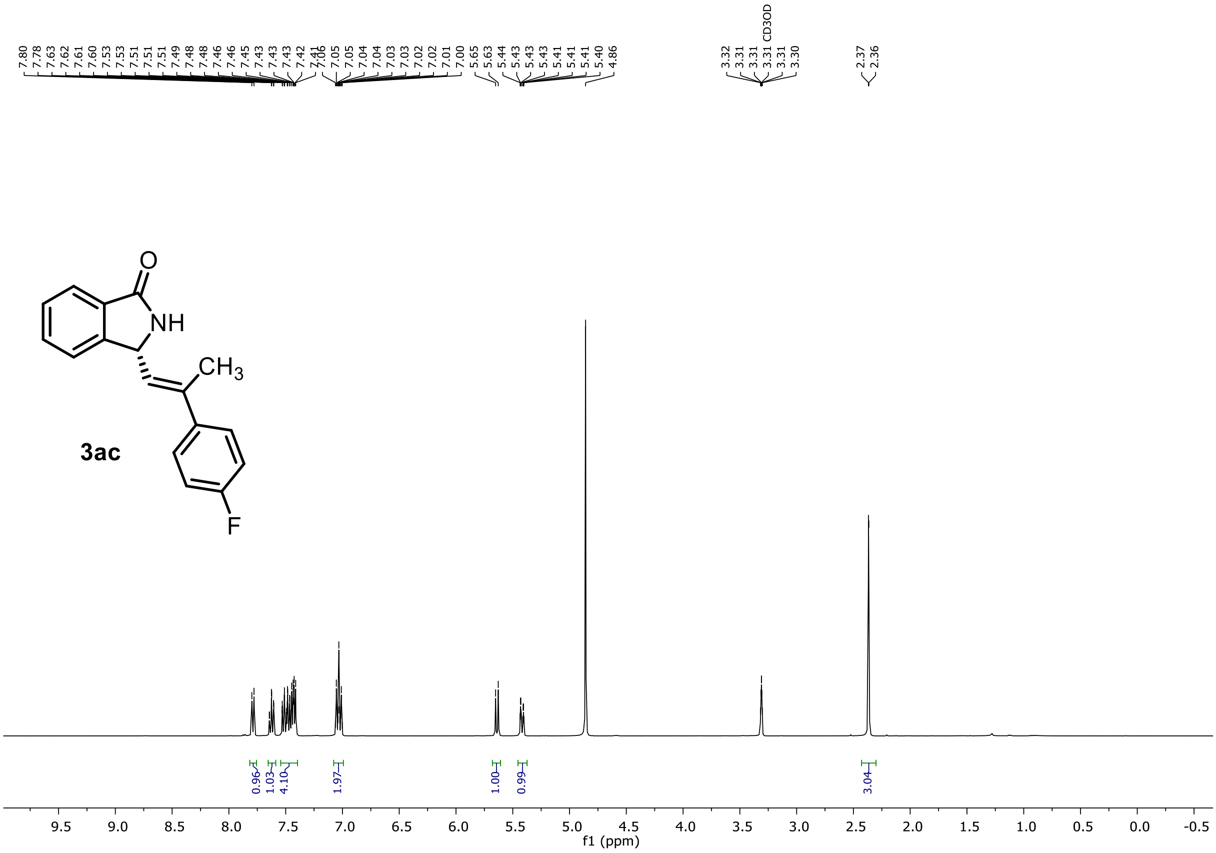

^{1}H NMR of **3ac** (400 MHz, Methanol-d$_4$)

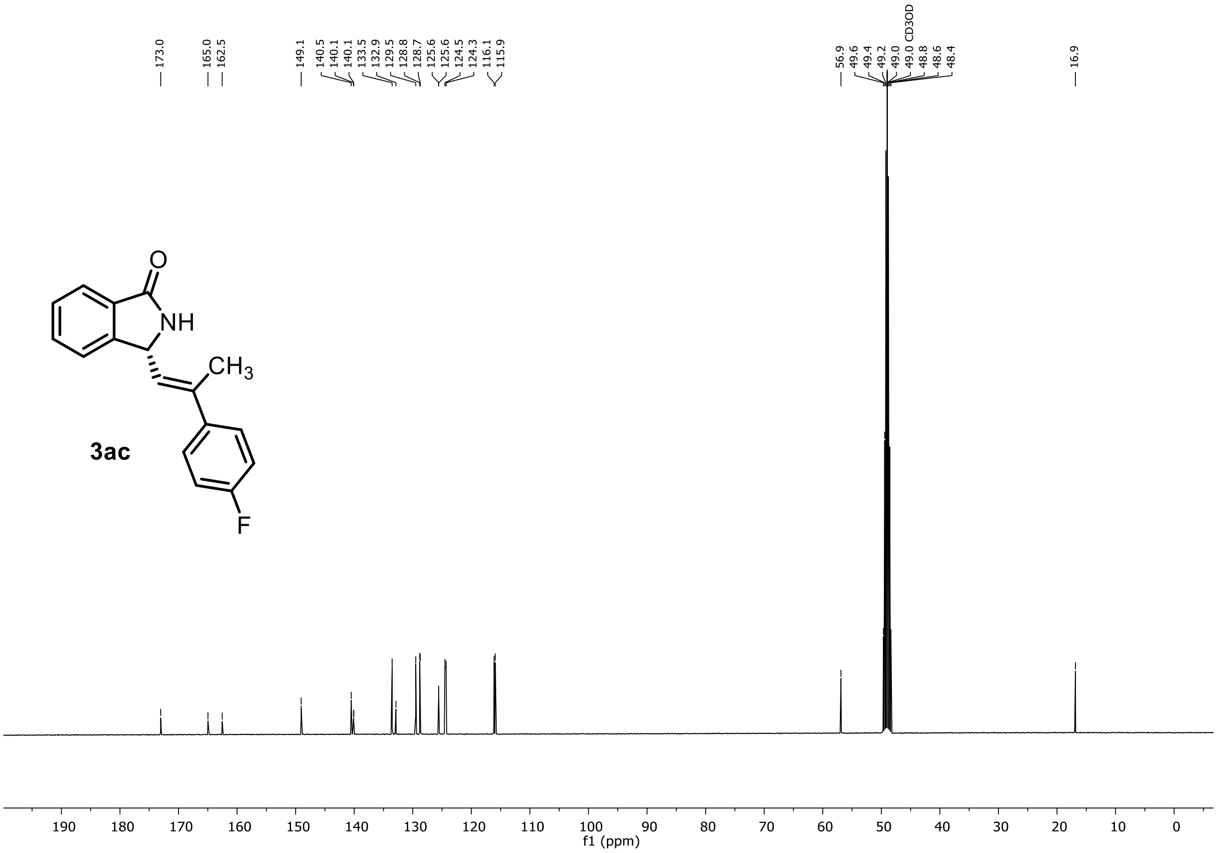

^{13}C NMR of **3ac** (101 MHz, Methanol-d$_4$)

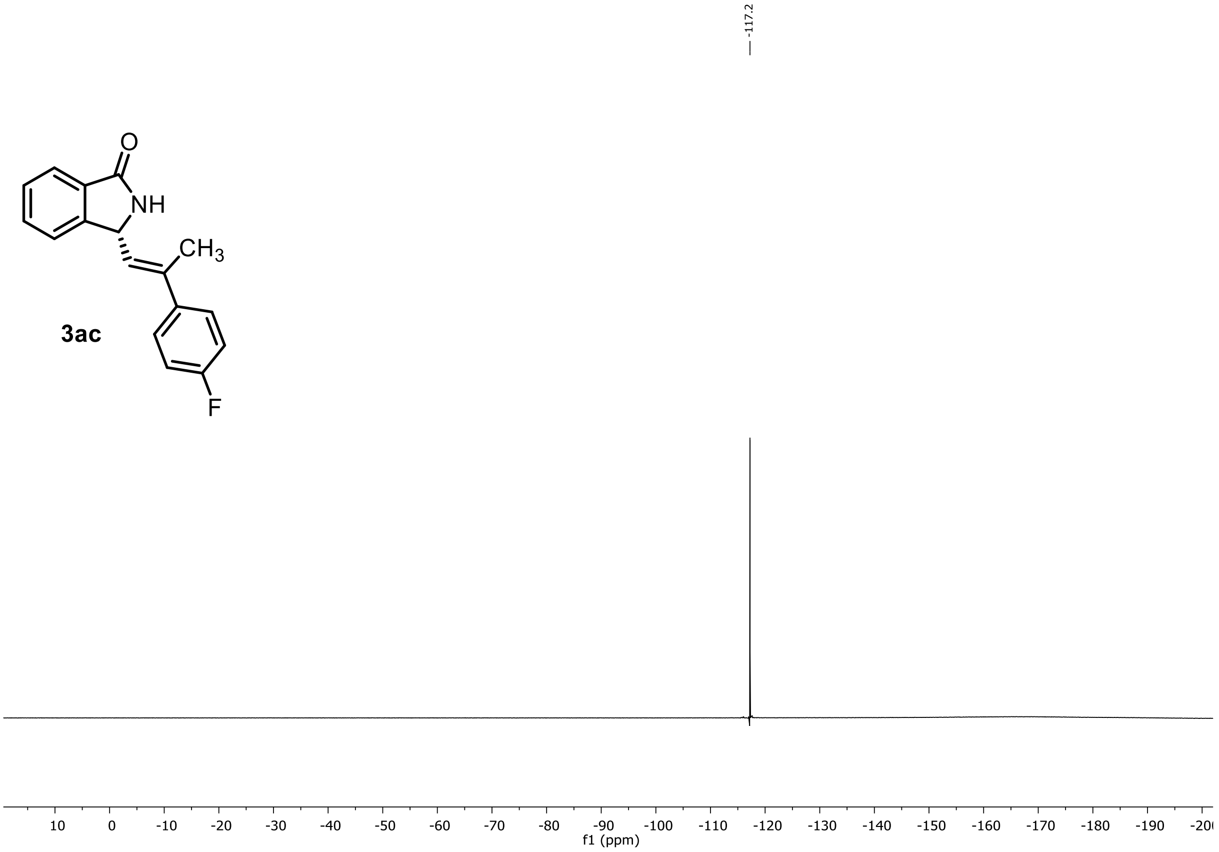

^{19}F NMR of **3ac** (376 MHz, Methanol-d$_4$)

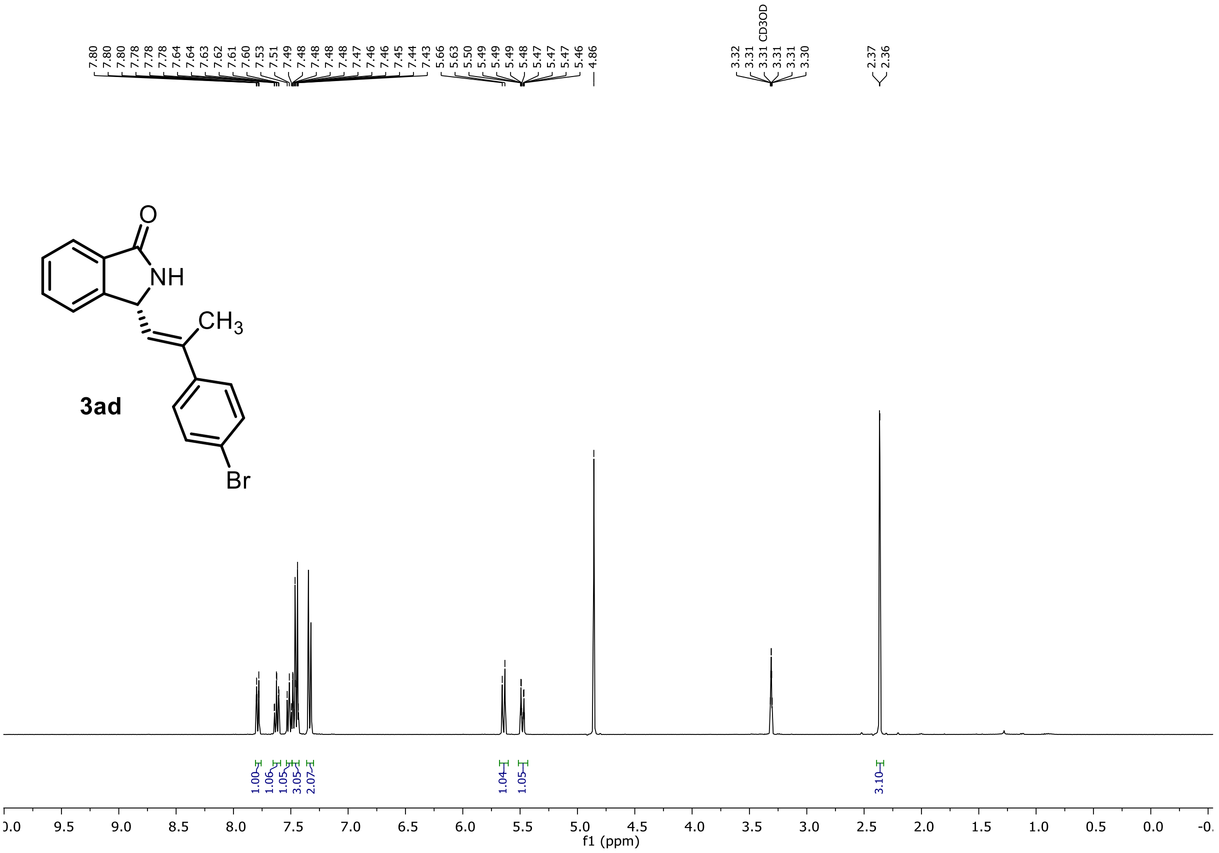

^{1}H NMR of **3ad** (400 MHz, Methanol-d$_4$)

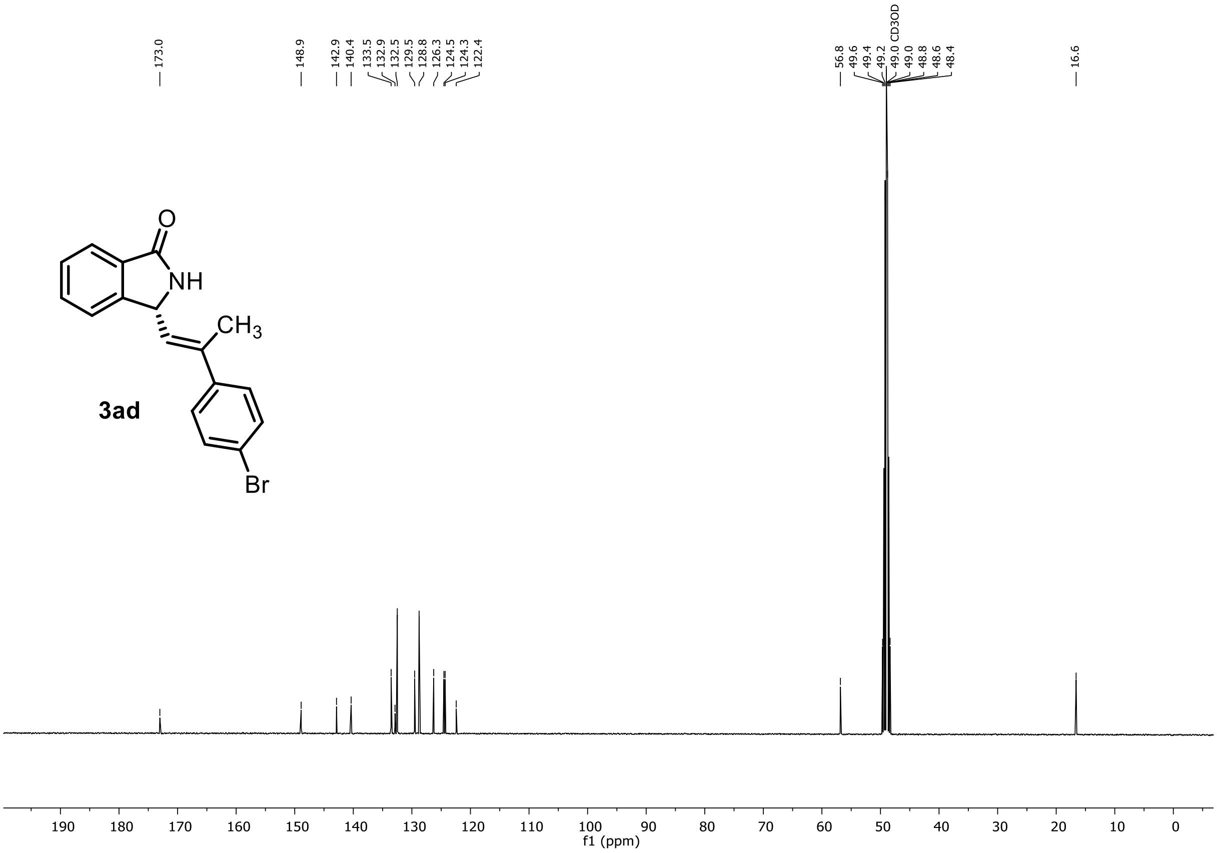

^{13}C NMR of **3ad** (101 MHz, Methanol-d$_4$)

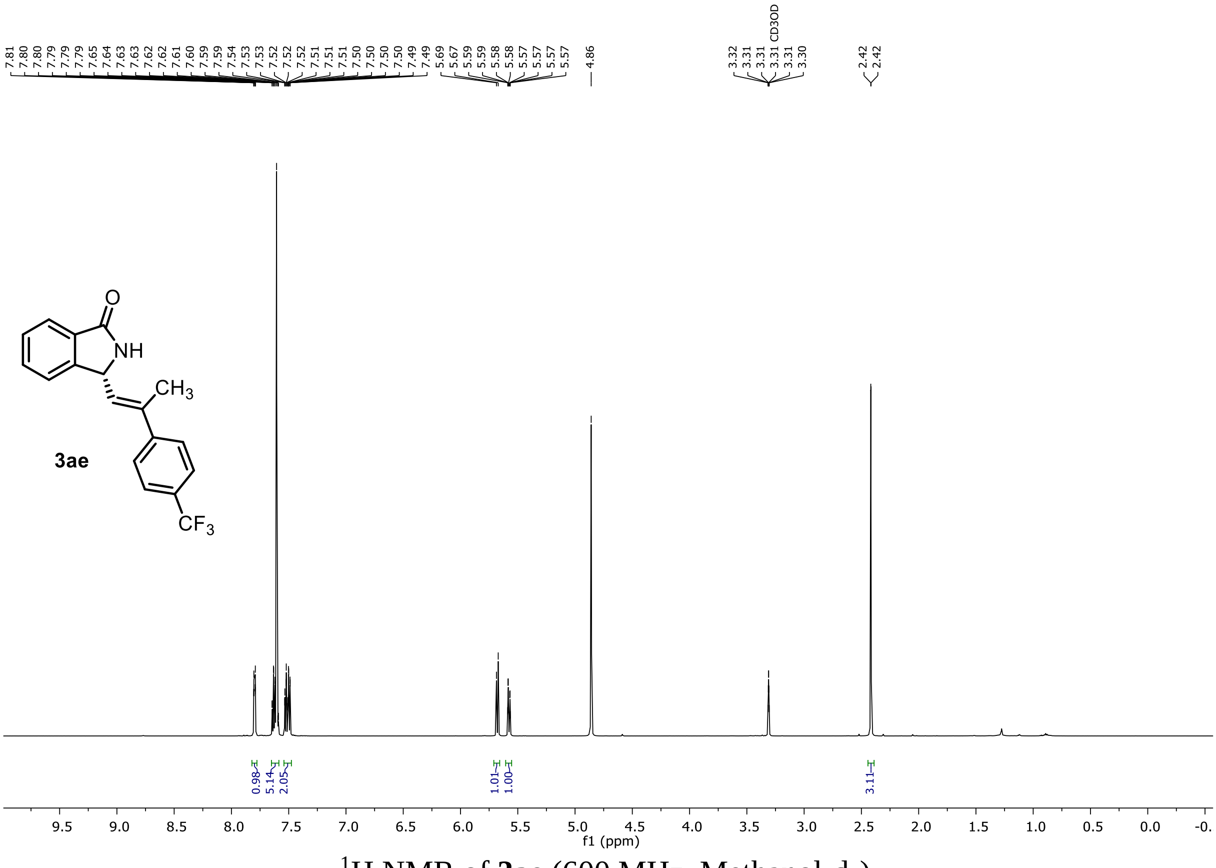

^{1}H NMR of **3ae** (600 MHz, Methanol-d$_4$)

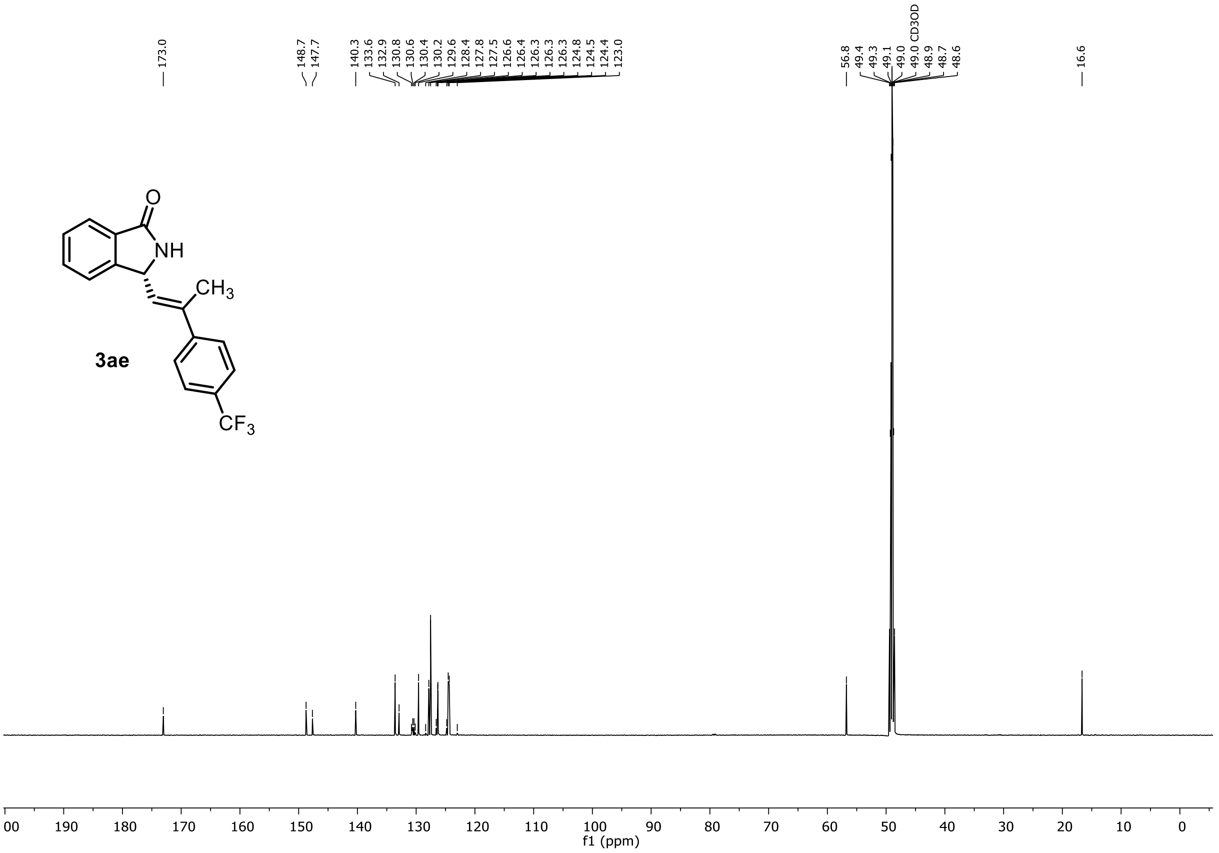

^{13}C NMR of **3ae** (151 MHz, Methanol-d$_4$)

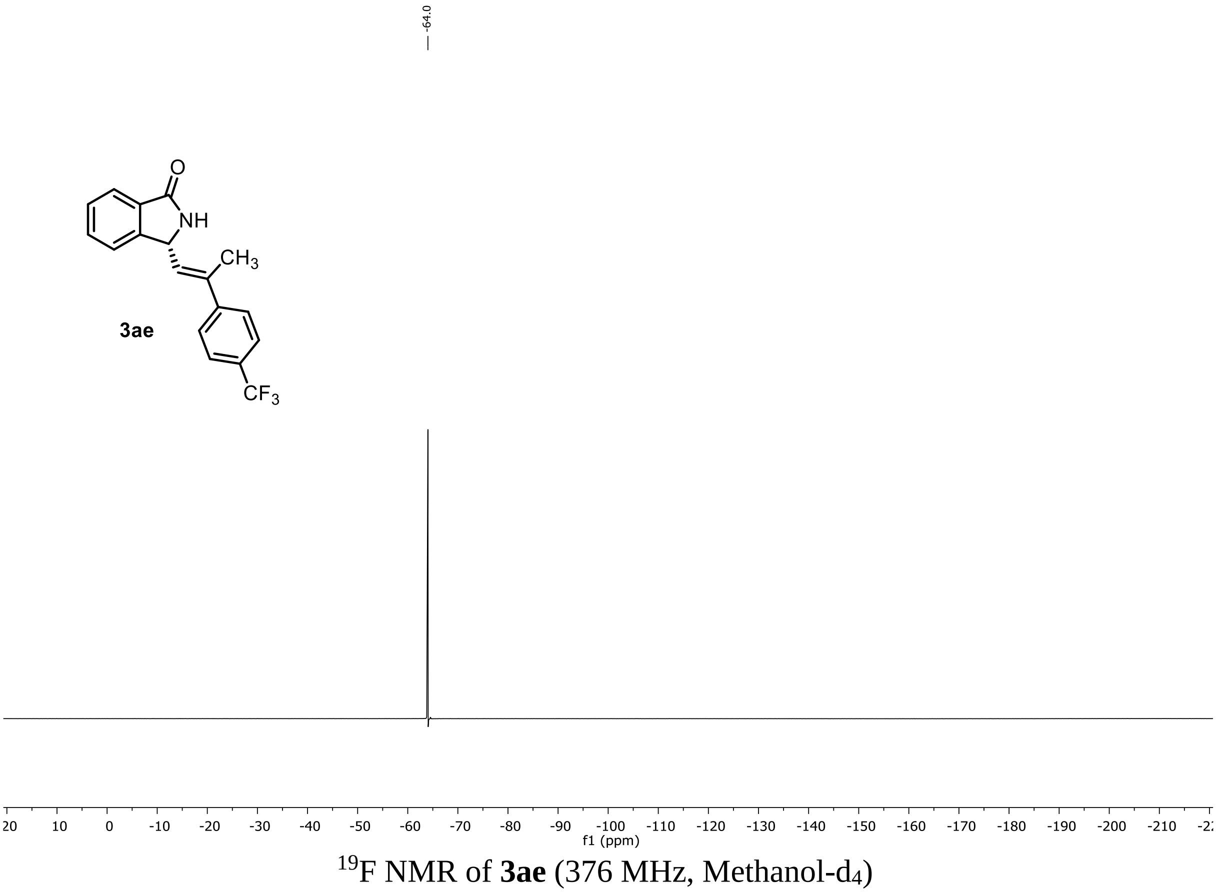

^{19}F NMR of **3ae** (376 MHz, Methanol-d$_4$)

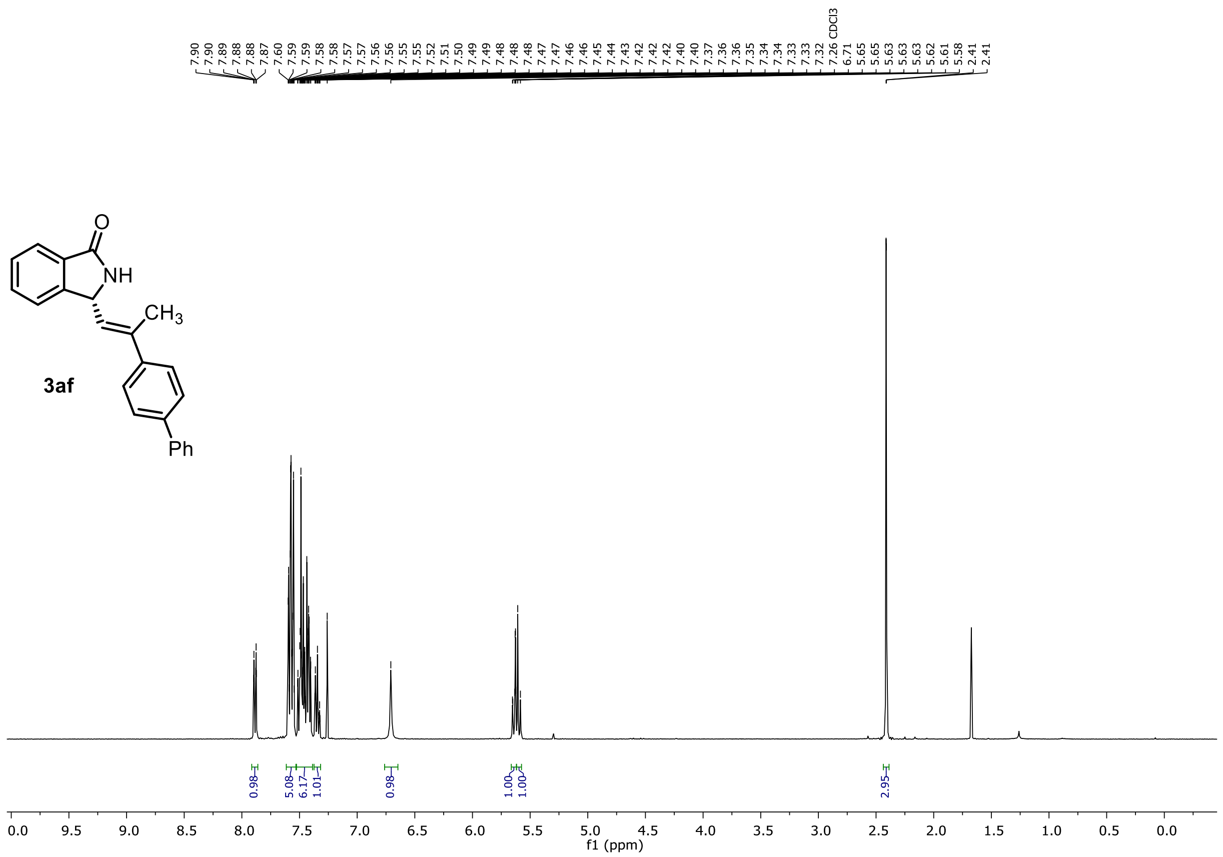

^{1}H NMR of **3af** (400 MHz, $CDCl_3$)

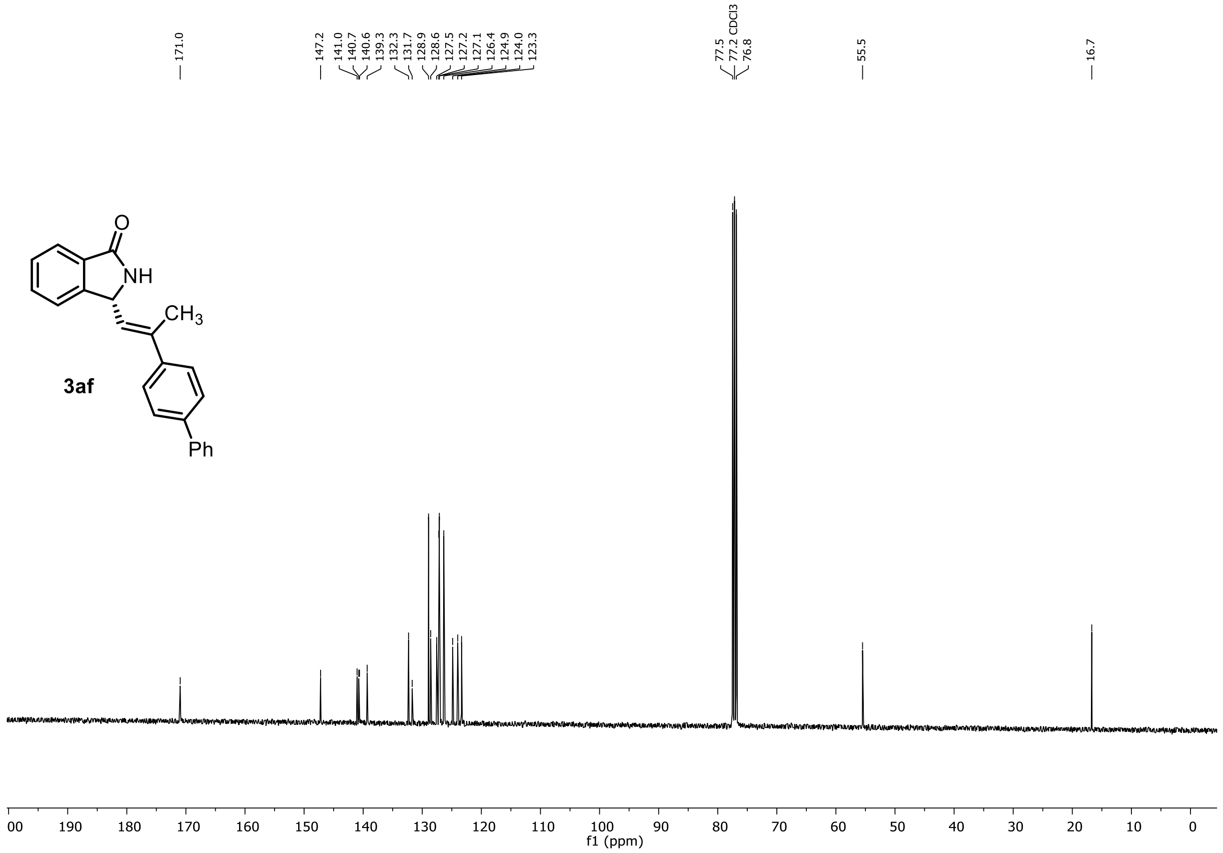

^{13}C NMR of **3af** (101 MHz, $CDCl_3$)

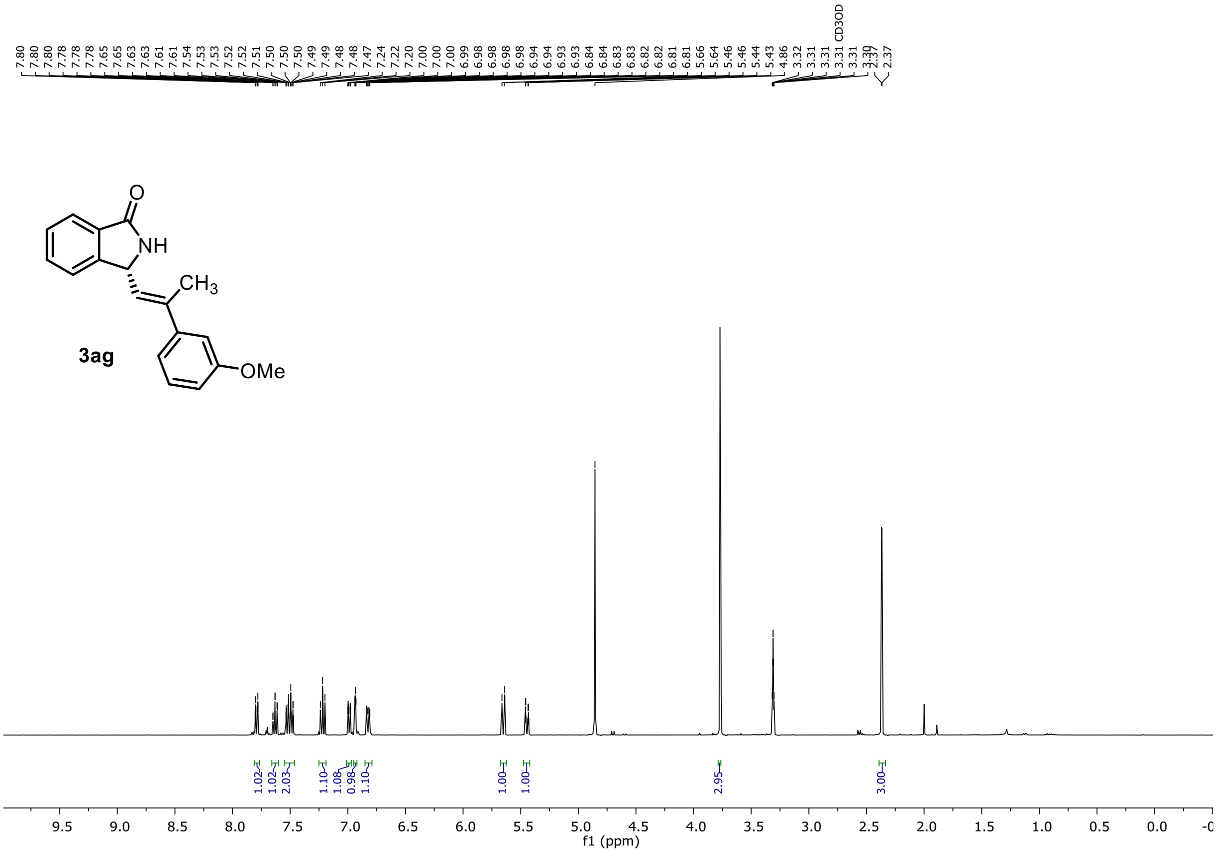

^{1}H NMR of **3ag** (400 MHz, Methanol-d$_4$)

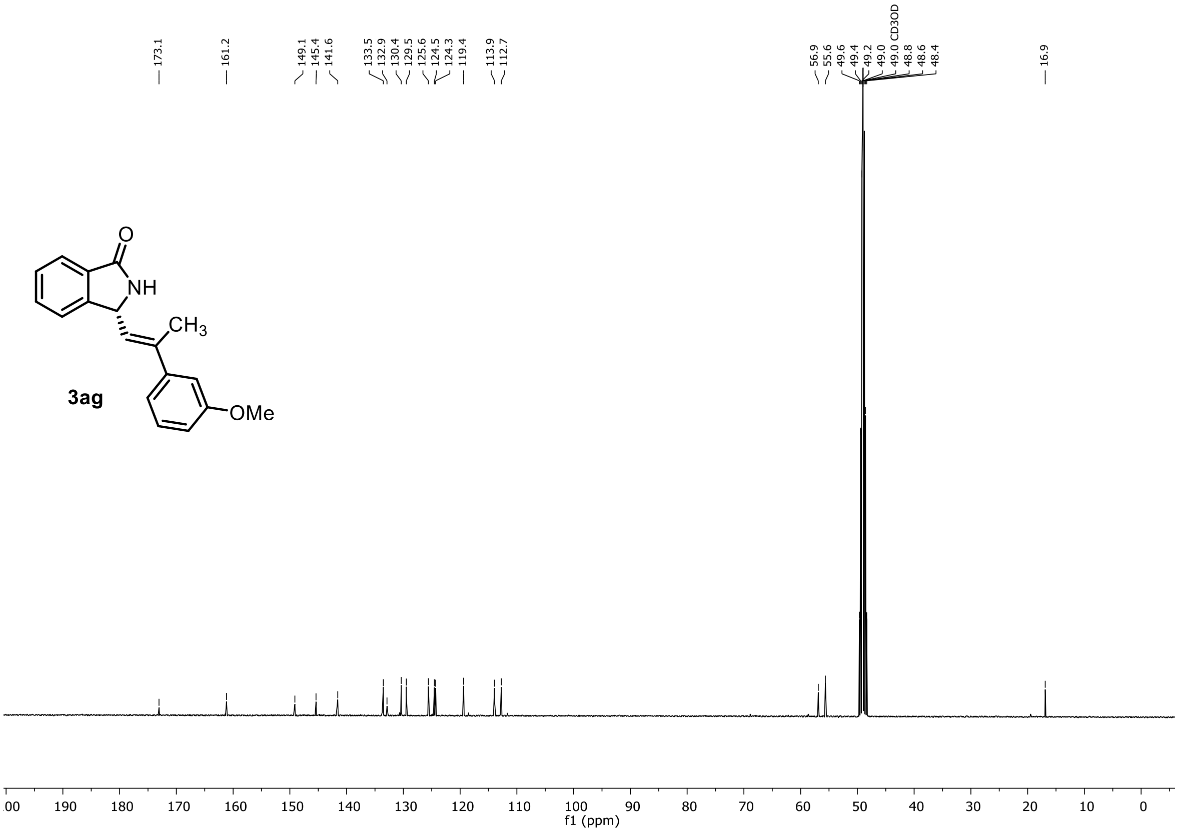

^{13}C NMR of **3ag** (101 MHz, Methanol-d$_4$)

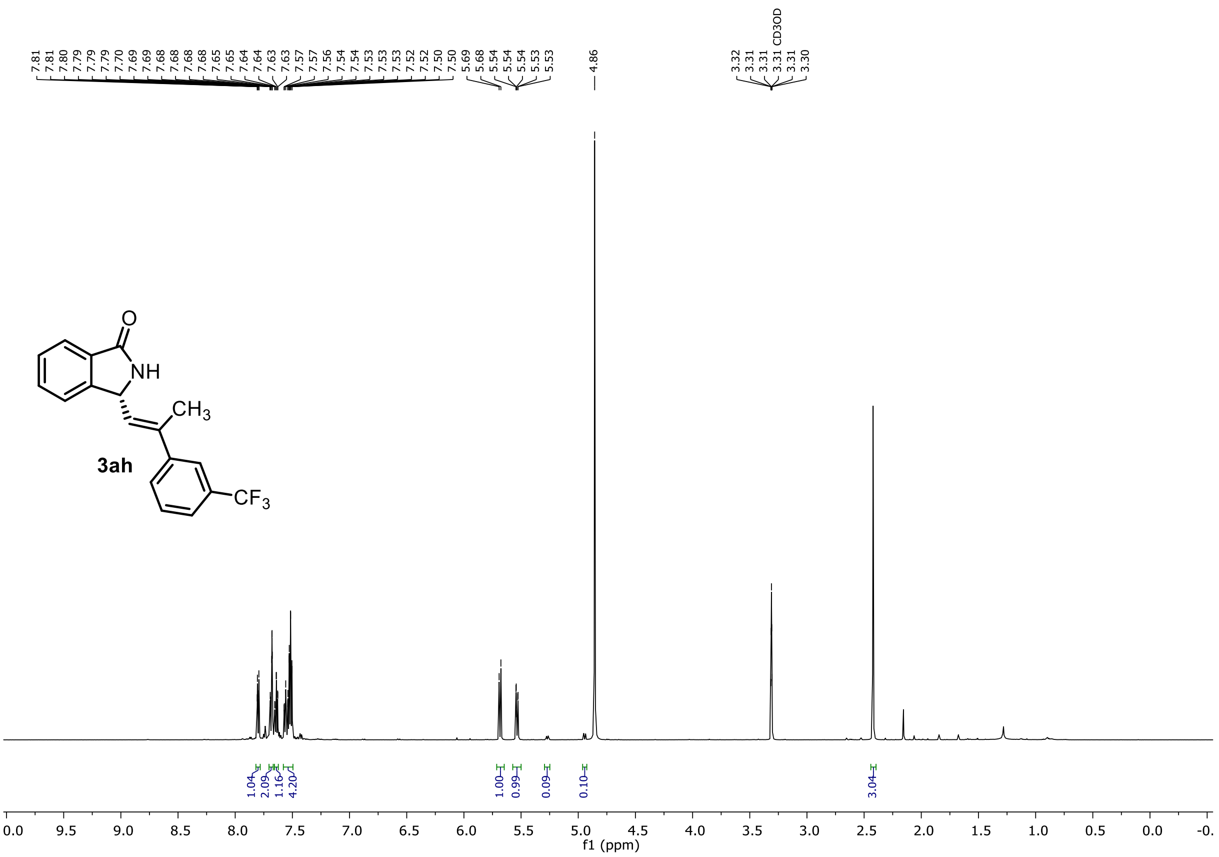

^{1}H NMR of **3ah** (600 MHz, Methanol-d$_4$)

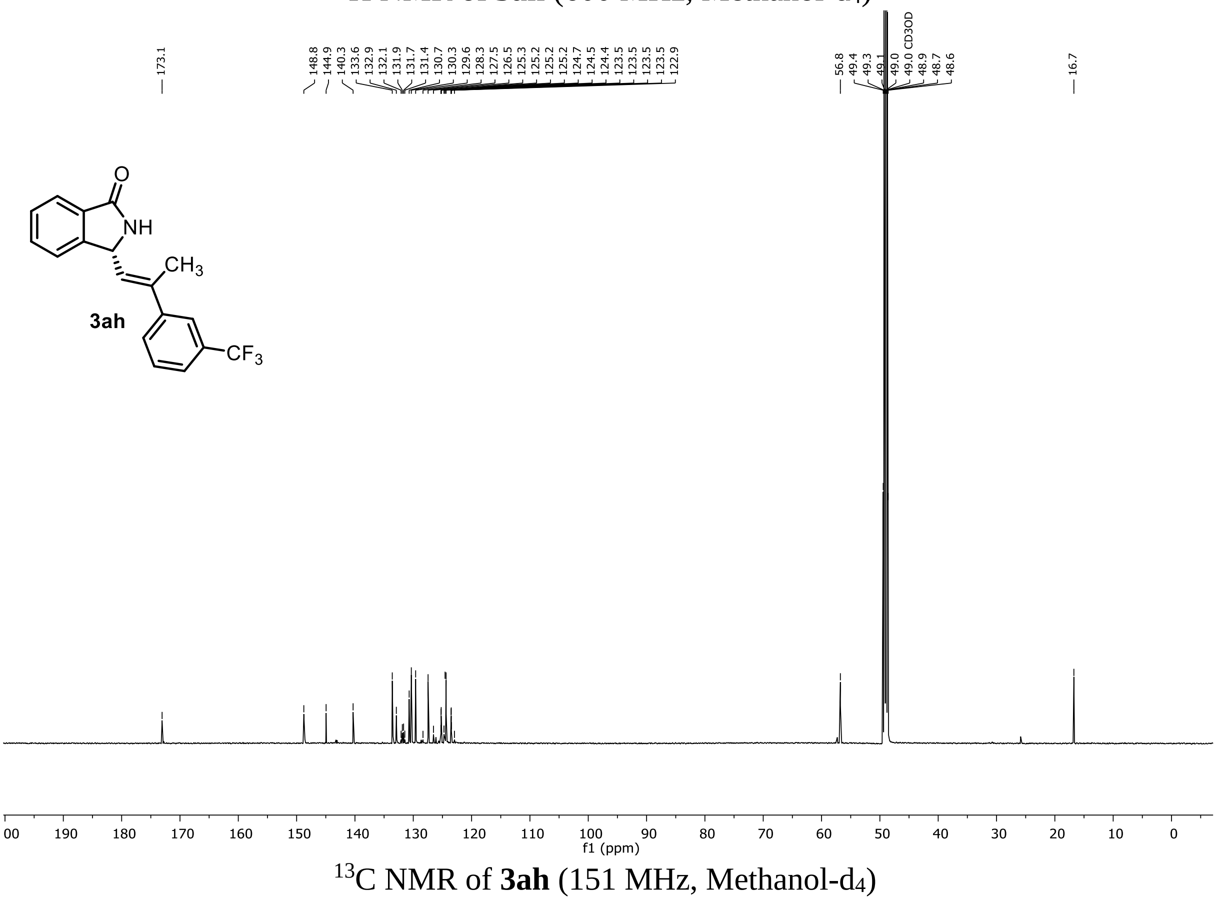

^{13}C NMR of **3ah** (151 MHz, Methanol-d$_4$)

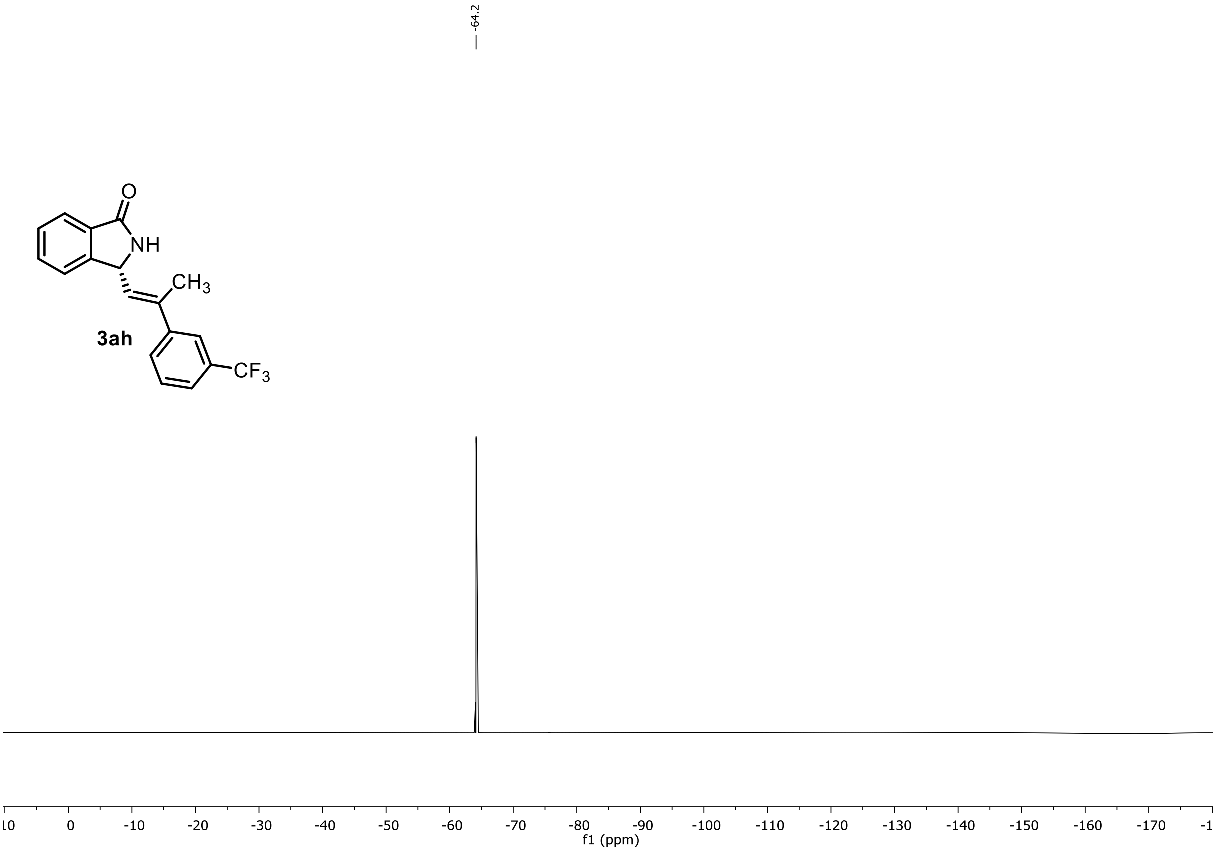

^{19}F NMR of **3ah** (564 MHz, Methanol-d$_4$)

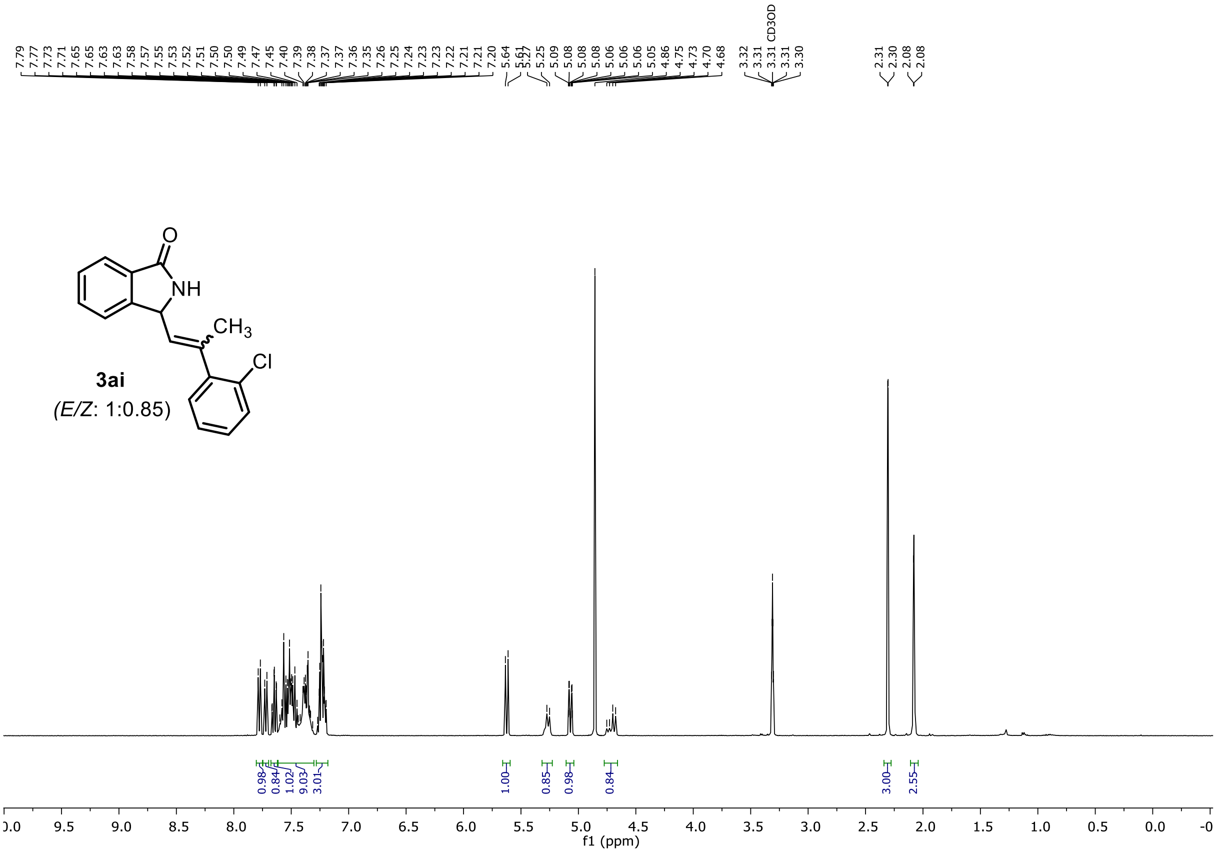

^{1}H NMR of **3ai** (400 MHz, Methanol-d$_4$)

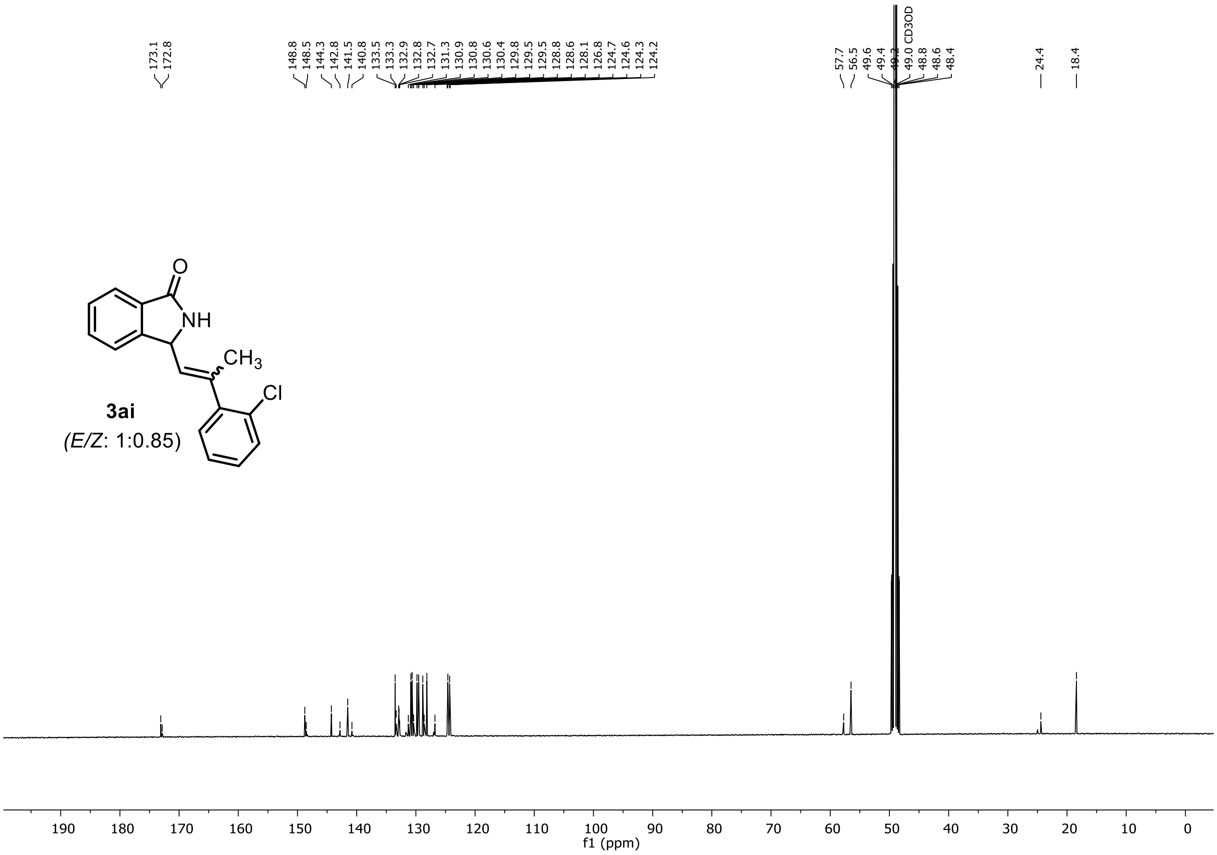

^{13}C NMR of **3ai** (101 MHz, Methanol-d$_4$)

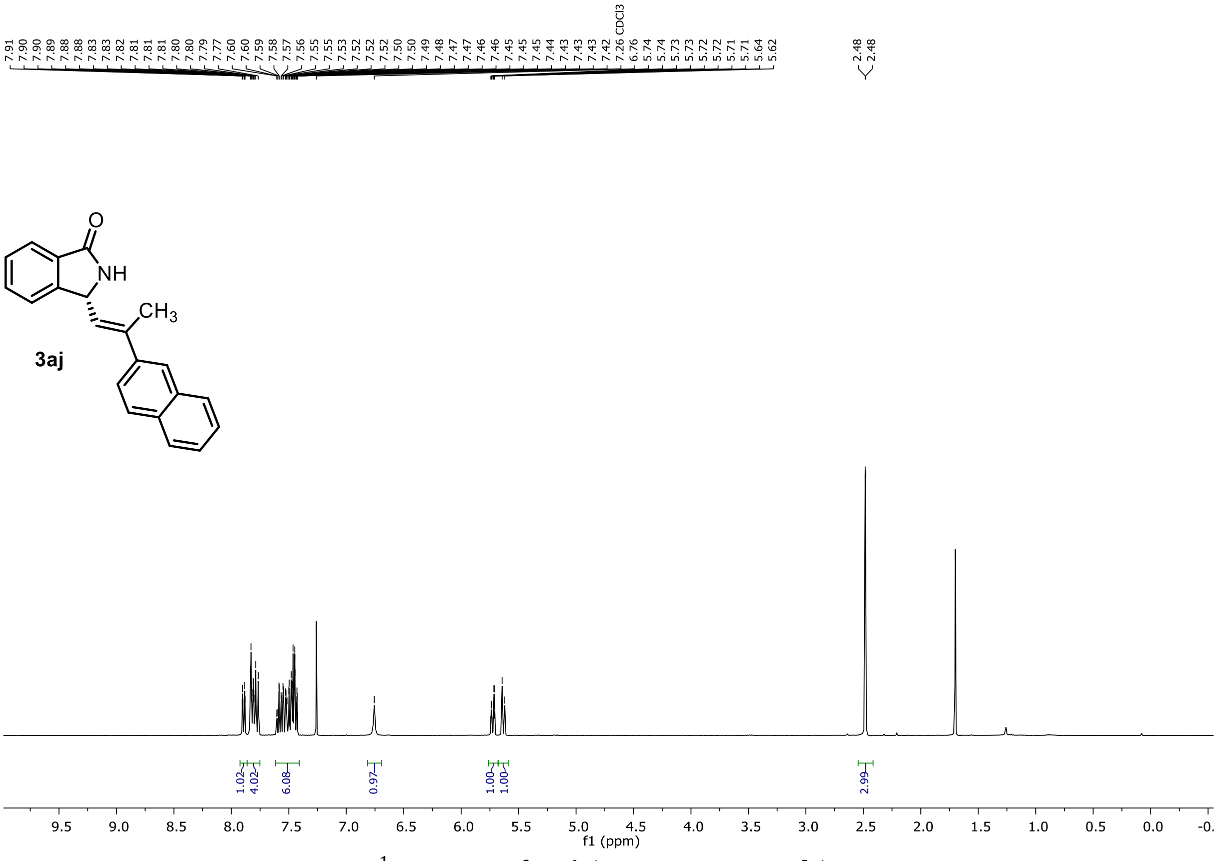

^{1}H NMR of **3aj** (400 MHz, $CDCl_3$)

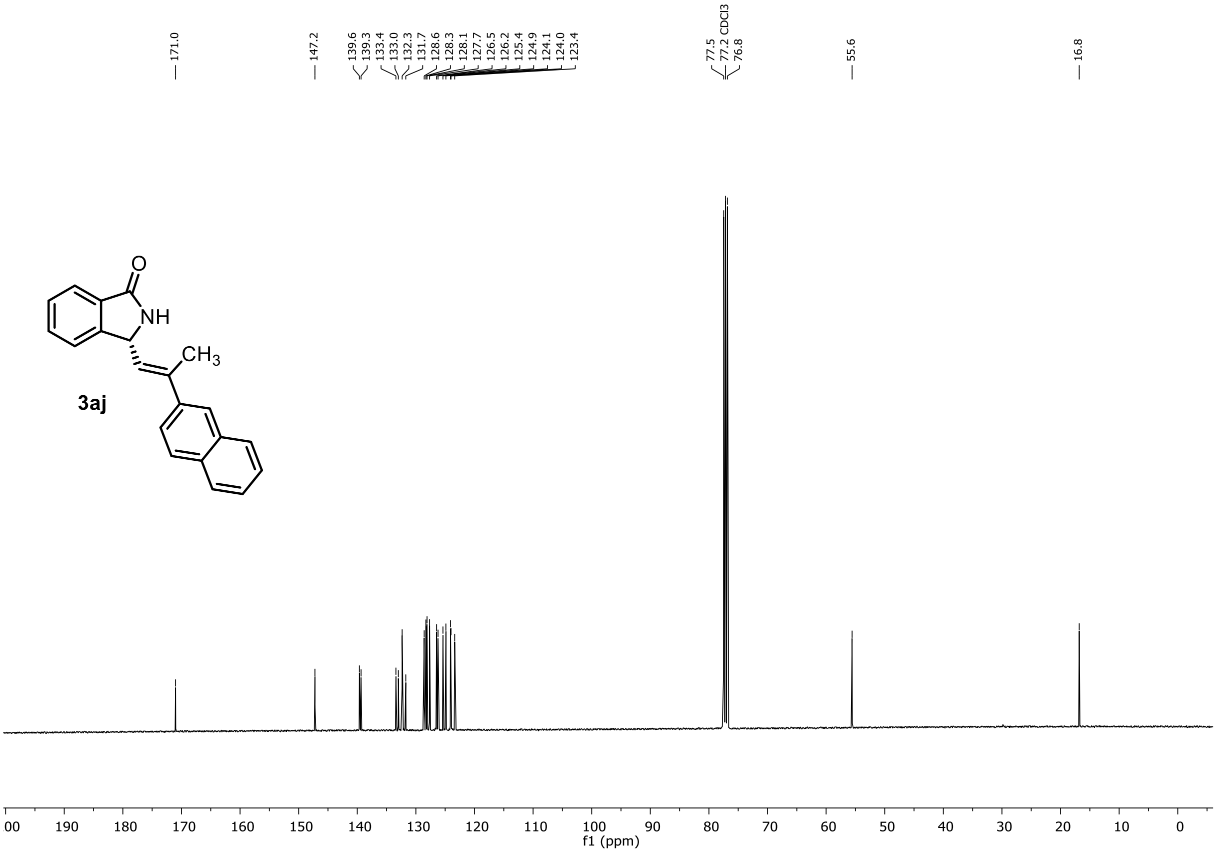

^{13}C NMR of **3aj** (101 MHz, $CDCl_3$)

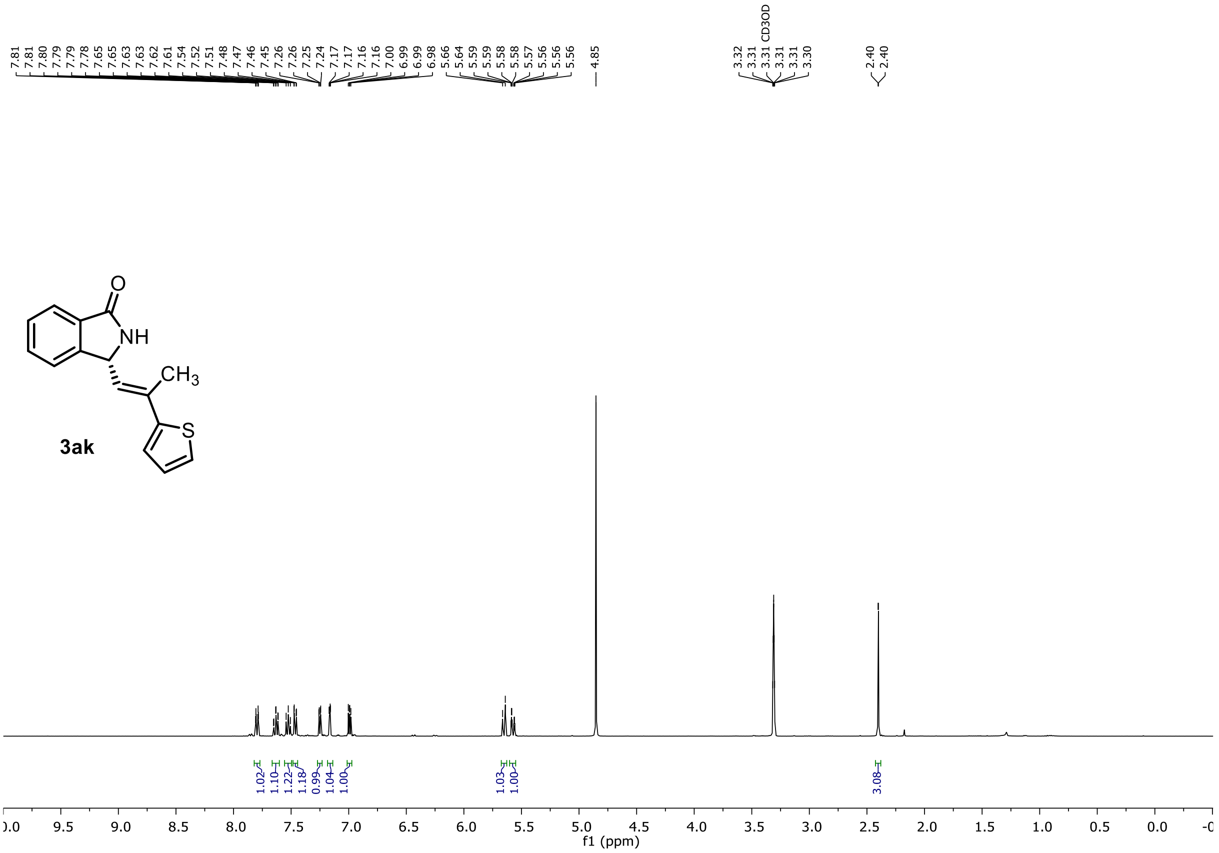

^{1}H NMR of **3ak** (400 MHz, Methanol-d$_4$)

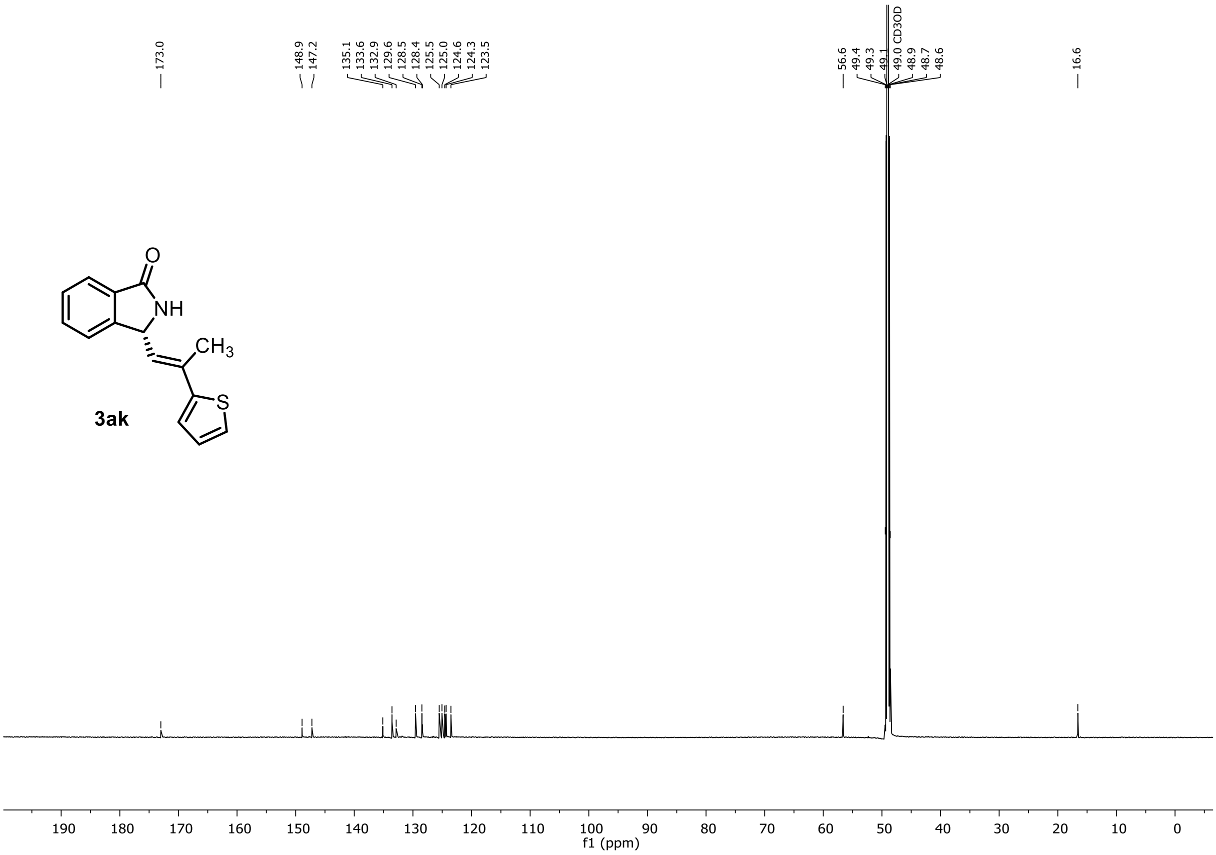

^{1}H NMR of **3ak** (151 MHz, Methanol-d$_4$)

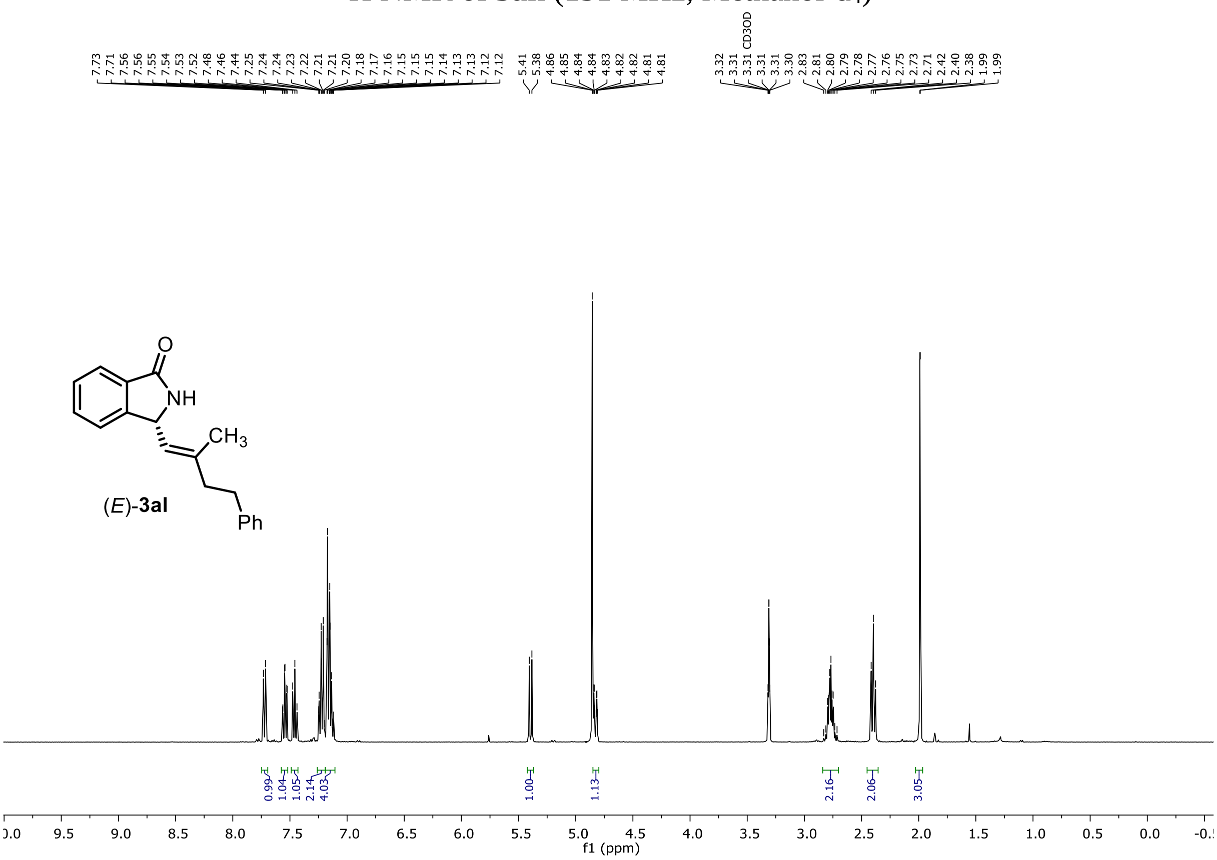

^{1}H NMR of (*E*)-**3al** (400 MHz, Methanol-d$_4$)

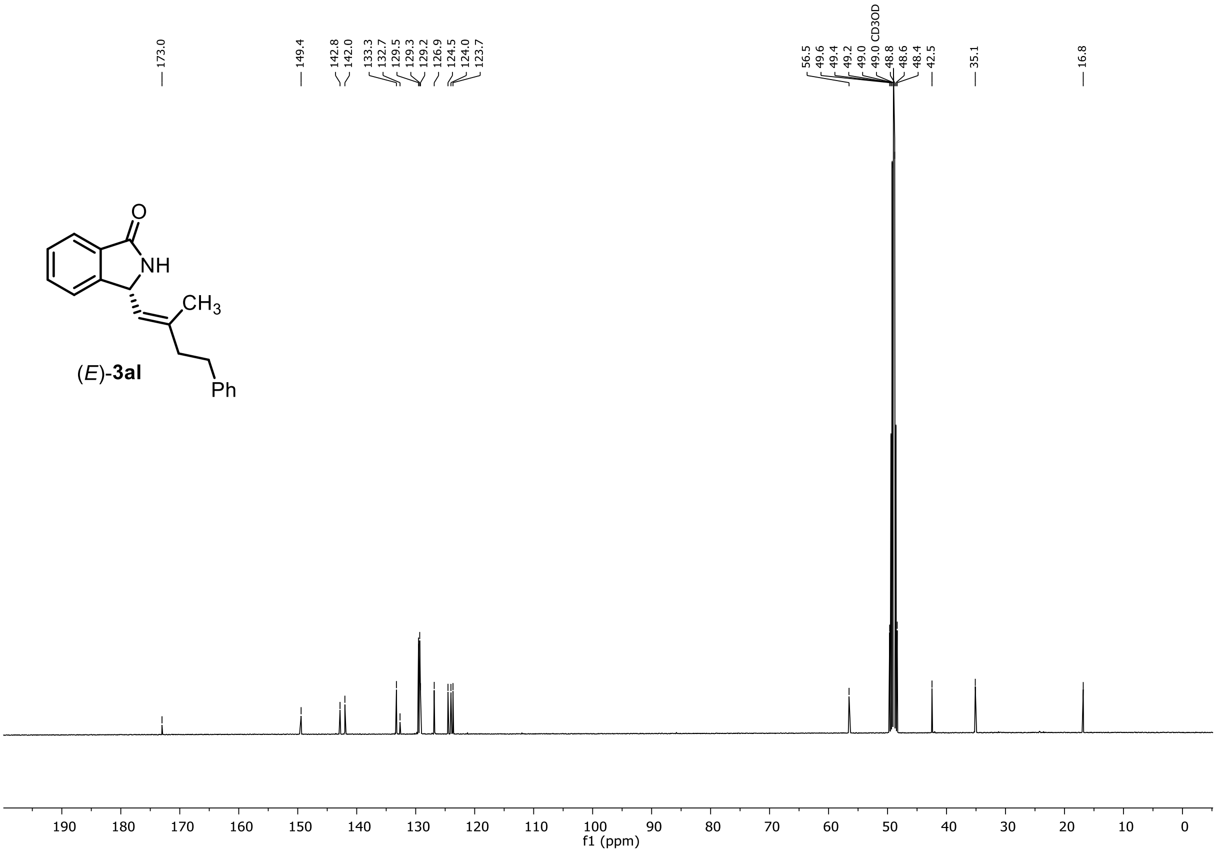

^{13}C NMR of (*E*)-**3al** (101MHz, Methanol-d$_4$)

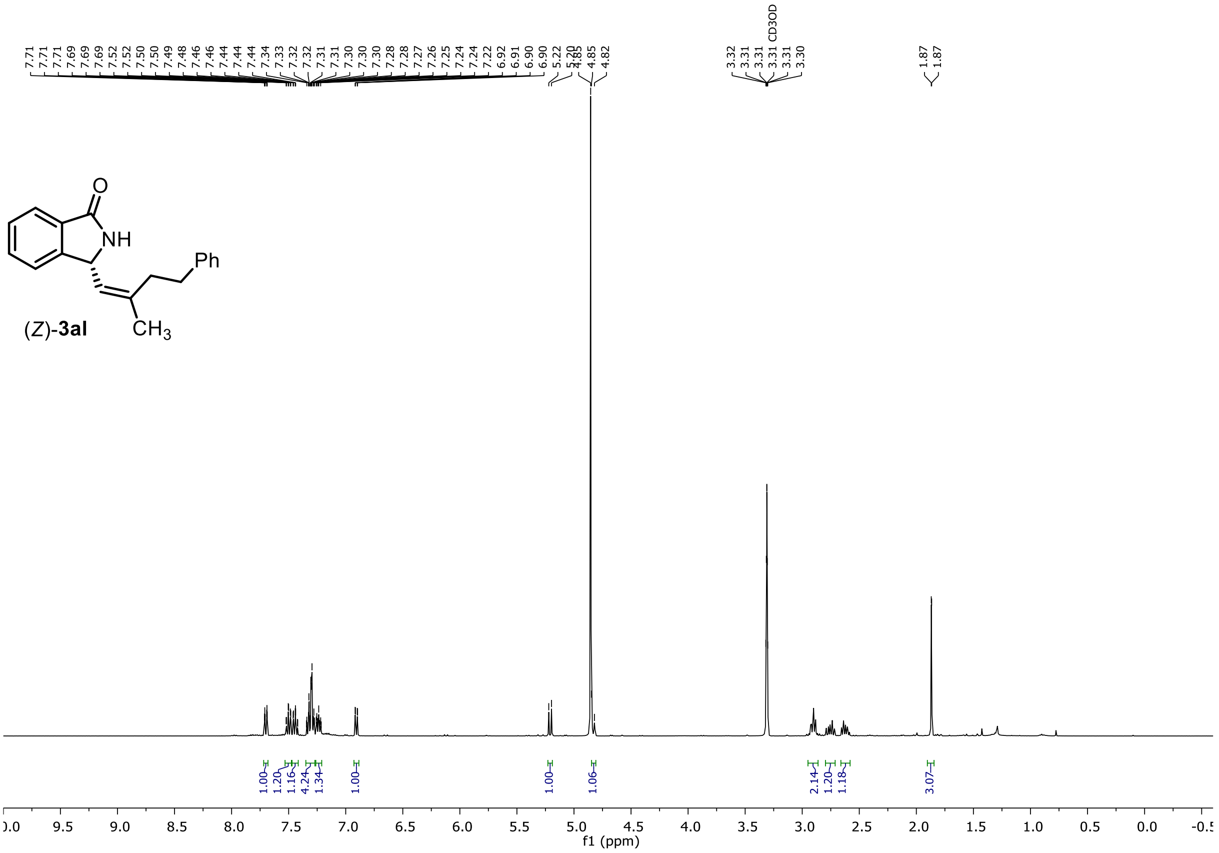

^{1}H NMR of (*Z*)-**3al** (400 MHz, Methanol-d$_4$)

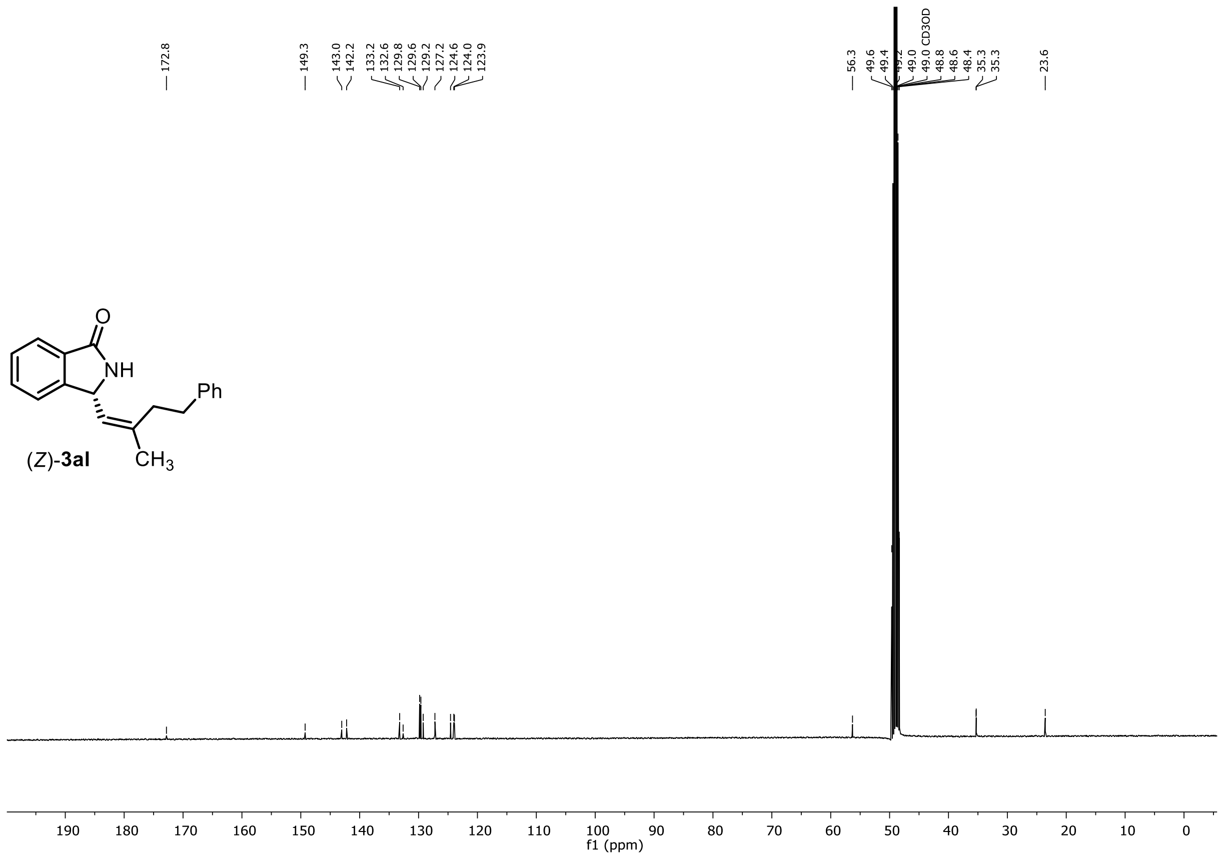

^{13}C NMR of (*Z*)-**3al** (101MHz, Methanol-d$_4$)

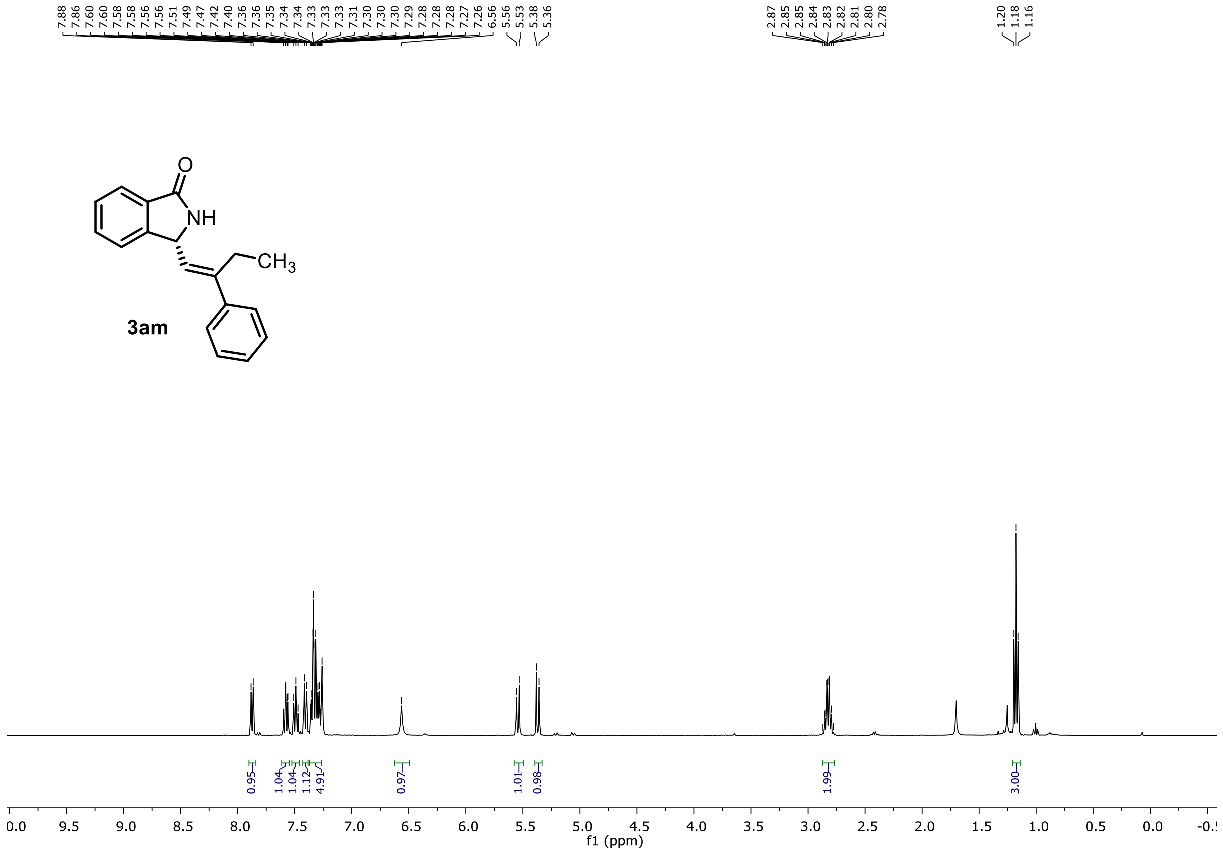

^{1}H NMR of **3am** (400 MHz, $CDCl_3$)

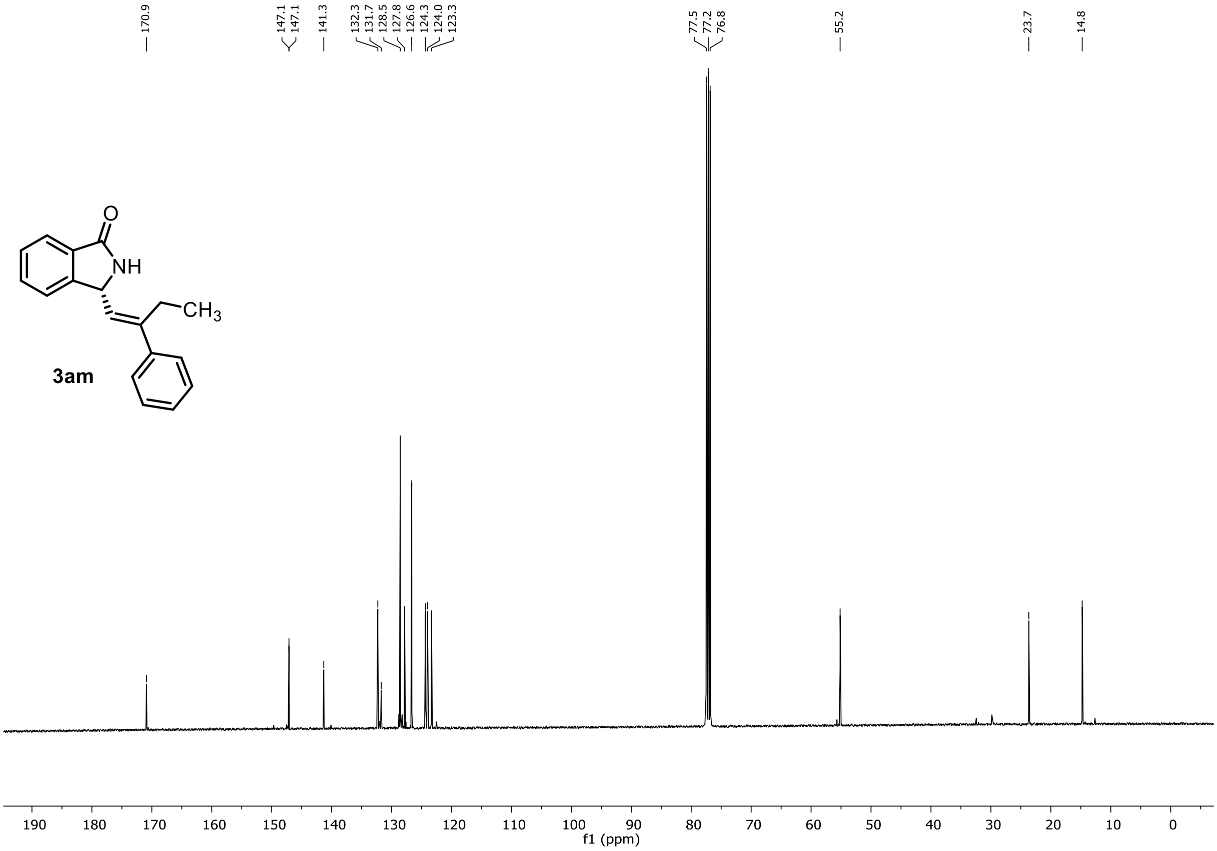

^{13}C NMR of **3am** (101 MHz, $CDCl_3$)

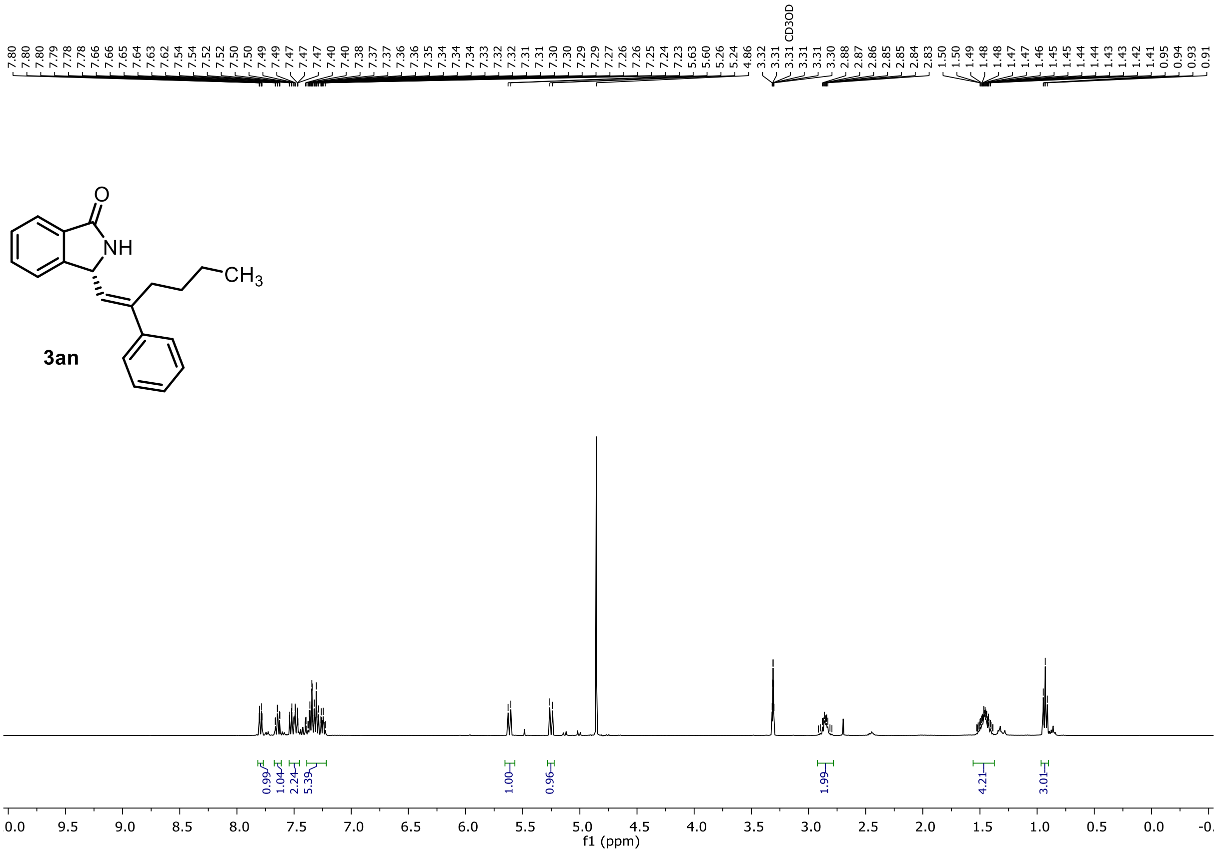

^{1}H NMR of **3an** (400 MHz, Methanol-d_4)

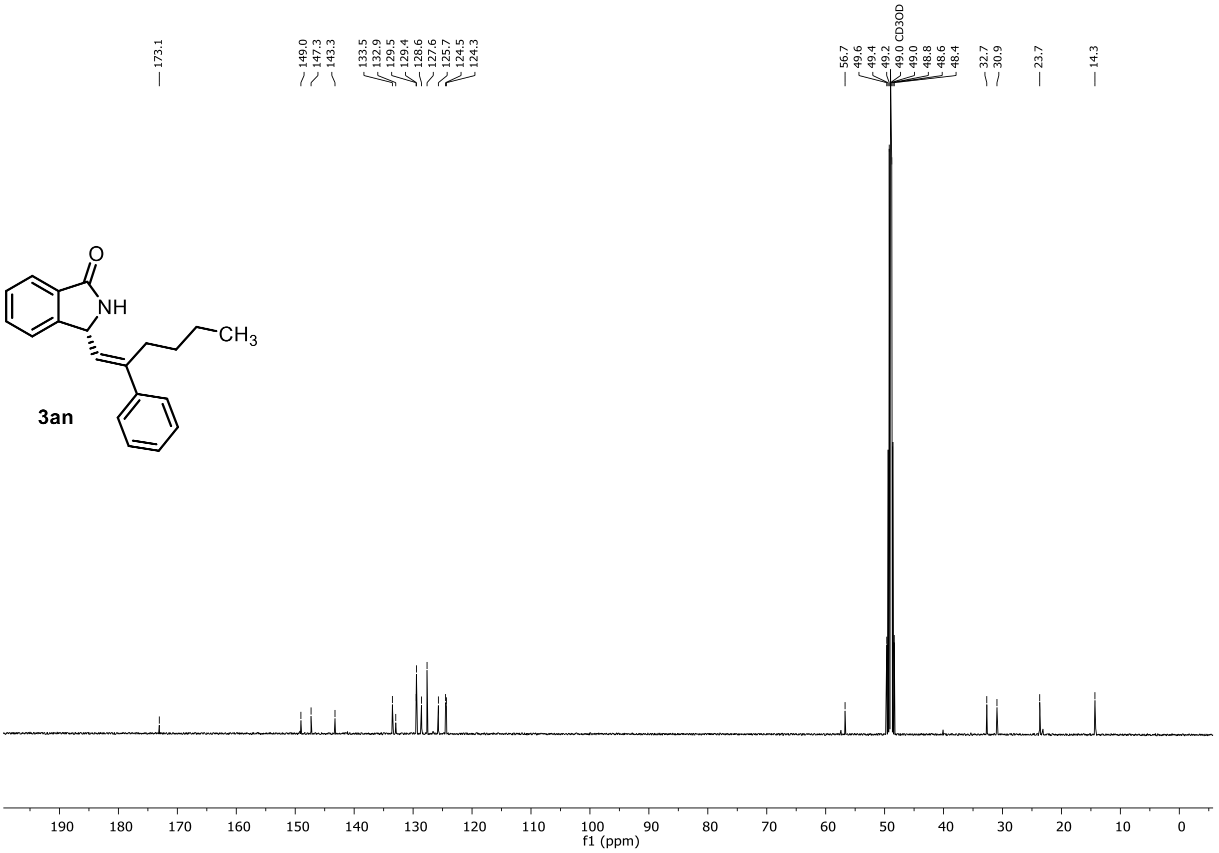

^{13}C NMR of **3an** (101 MHz, Methanol-d$_4$)

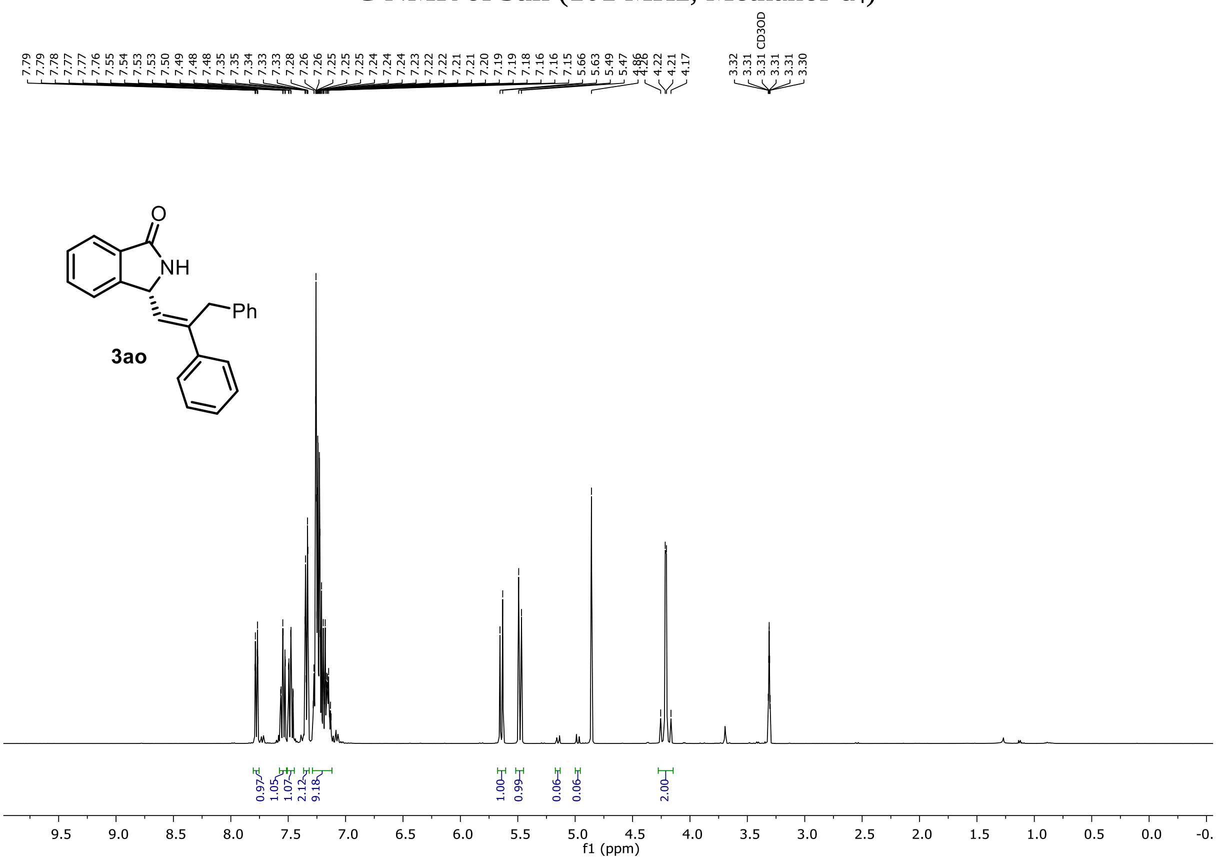

^{1}H NMR of **3ao** (400 MHz, Methanol-d$_4$)

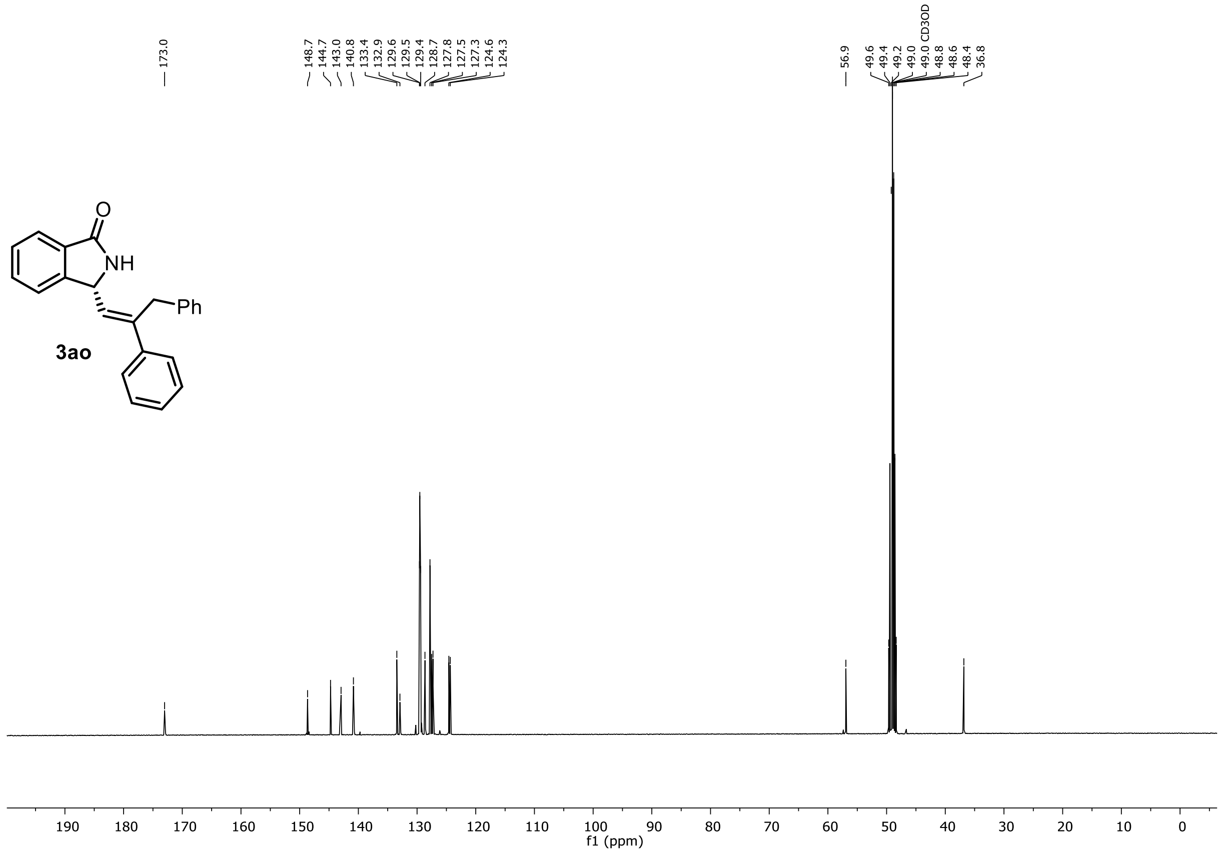

^{13}C NMR of **3ao** (101MHz, Methanol-d$_4$)

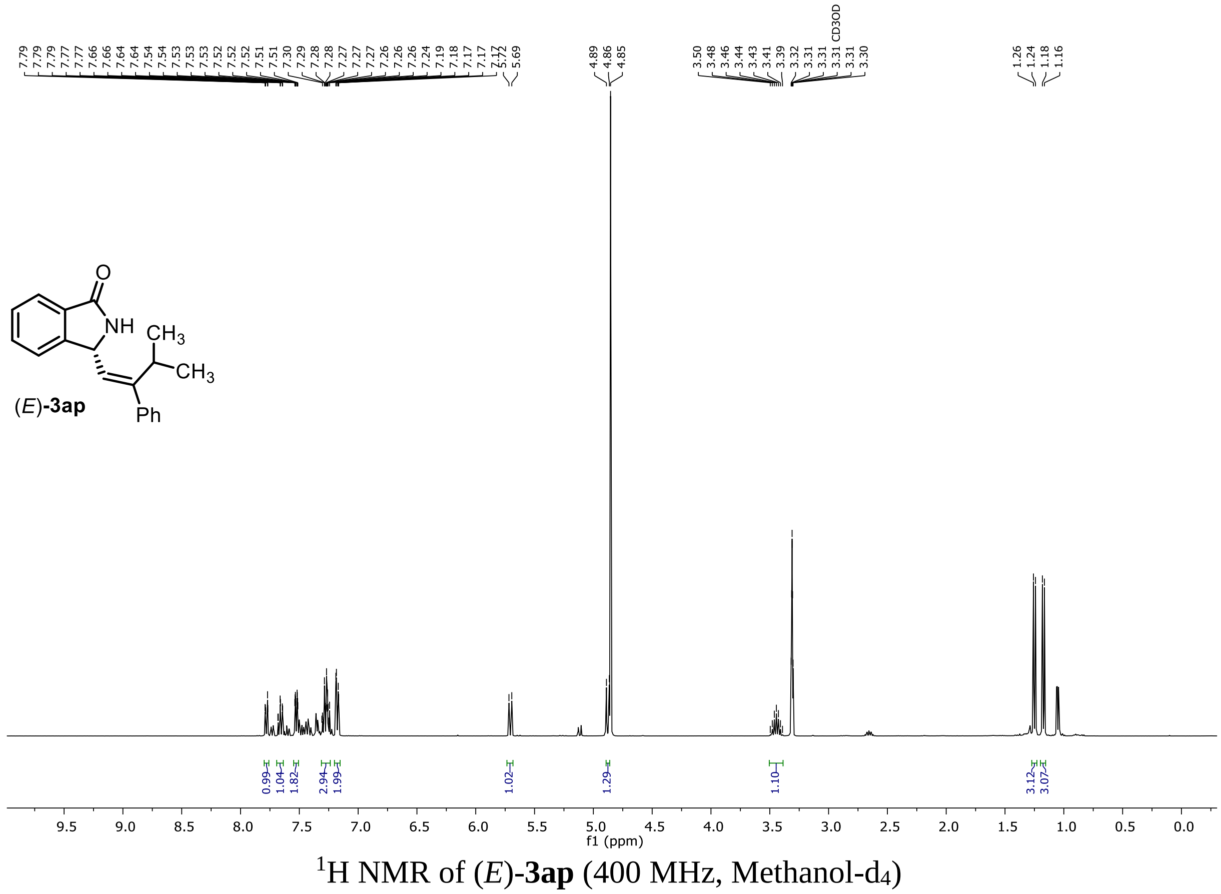

^{1}H NMR of (*E*)-**3ap** (400 MHz, Methanol-d$_4$)

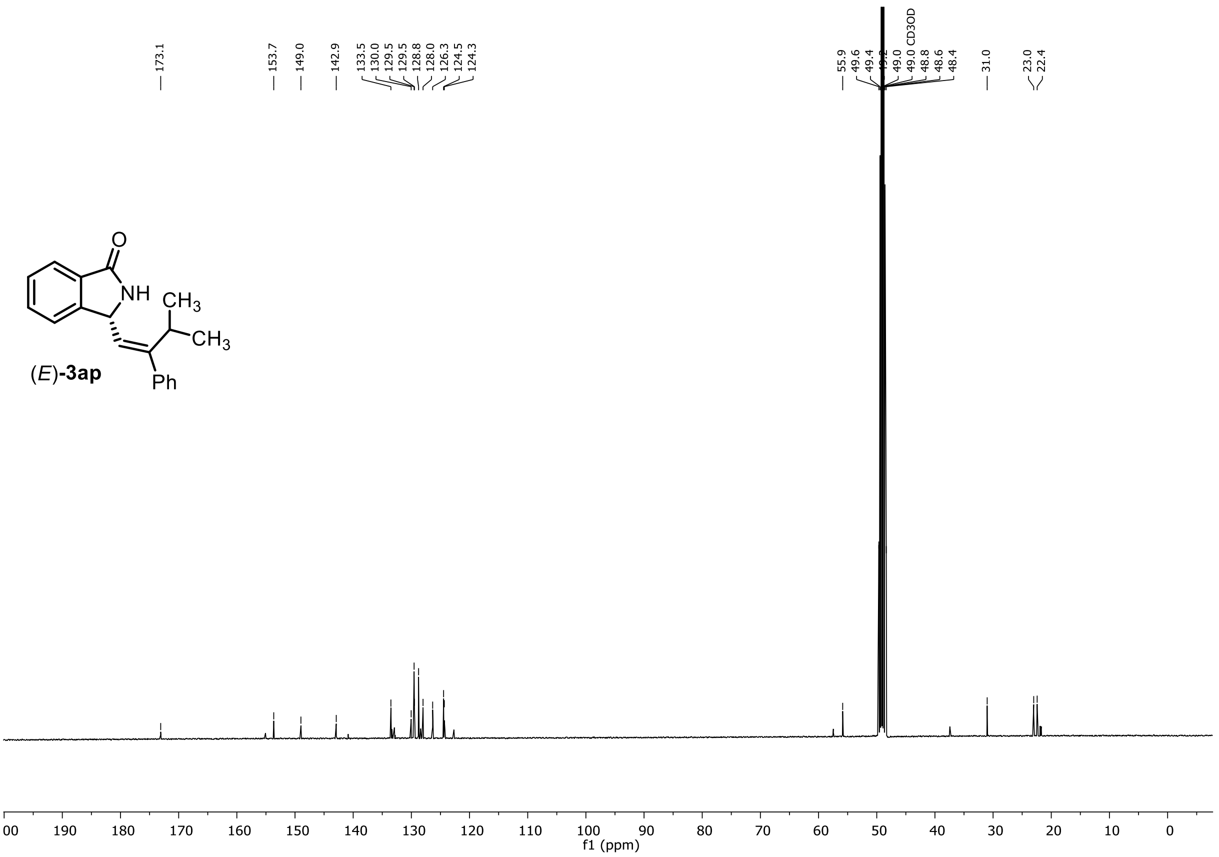

^{13}C NMR of (*E*)-**3ap** (101MHz, Methanol-d$_4$)

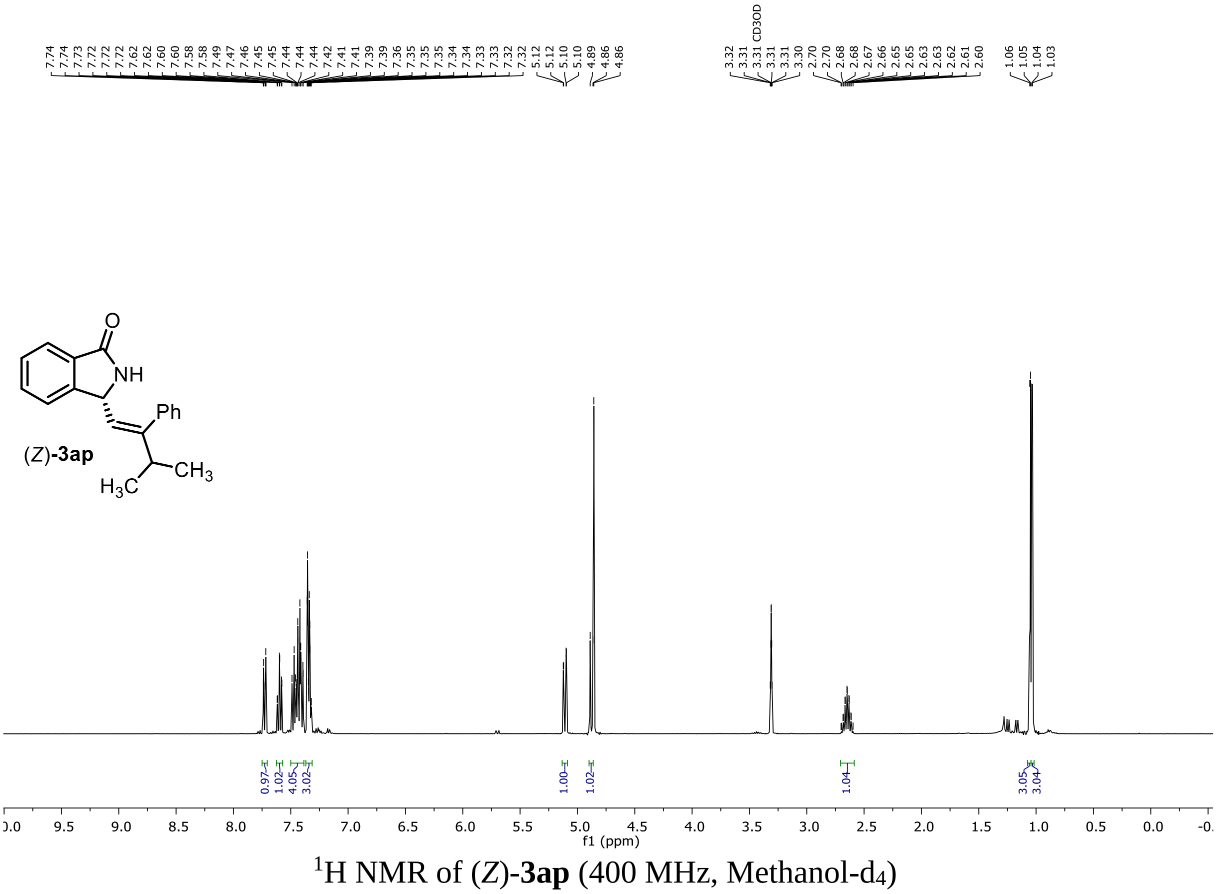

^{1}H NMR of (*Z*)-**3ap** (400 MHz, Methanol-d$_4$)

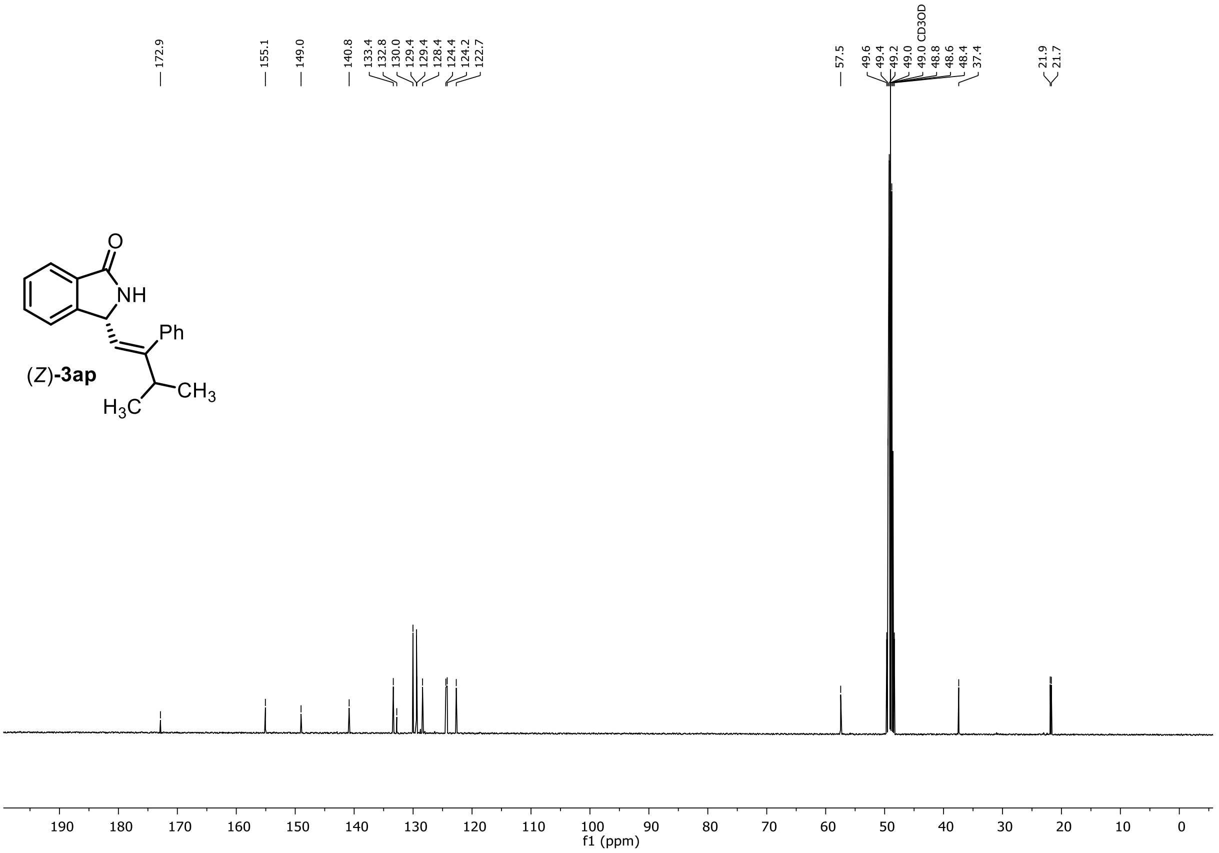

^{13}C NMR of (*Z*)-**3ap** (101 MHz, Methanol-d_4)

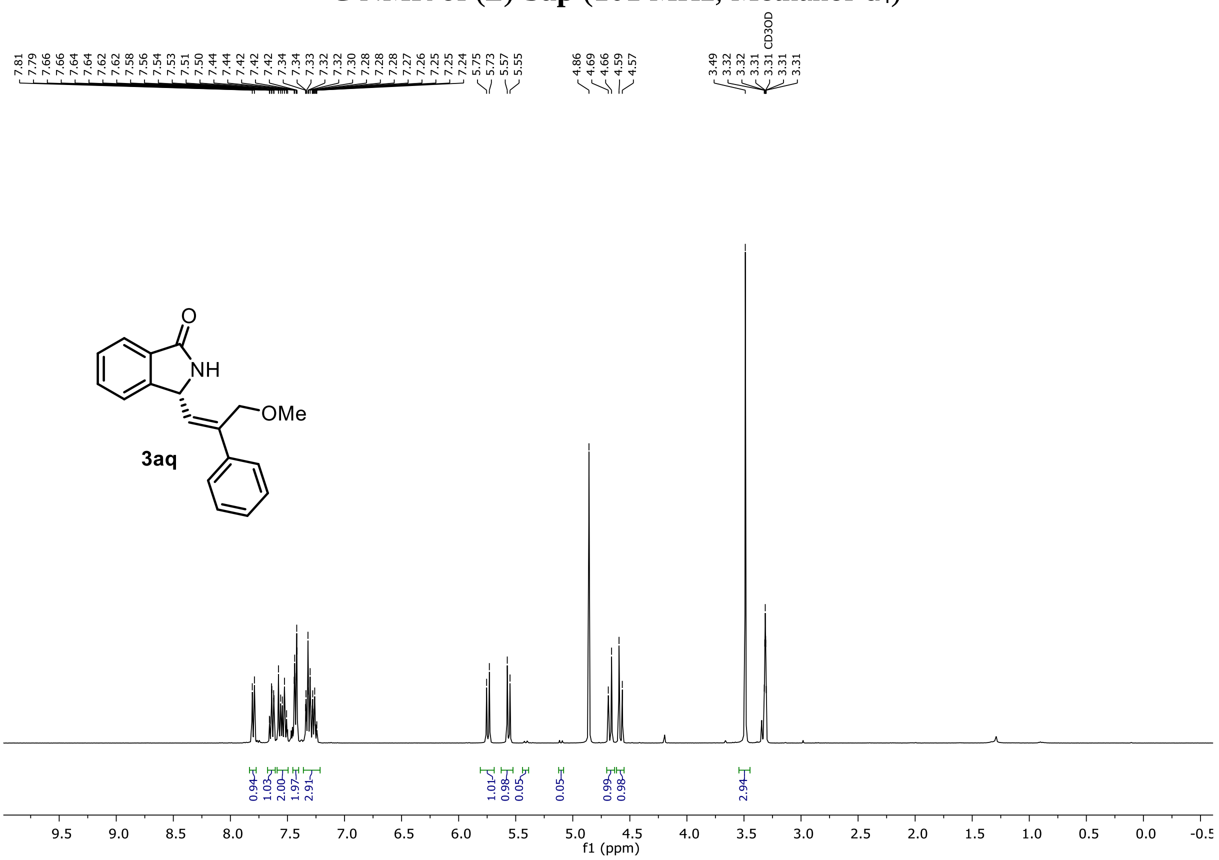

^{1}H NMR of **3aq** (400 MHz, Methanol-d_4)

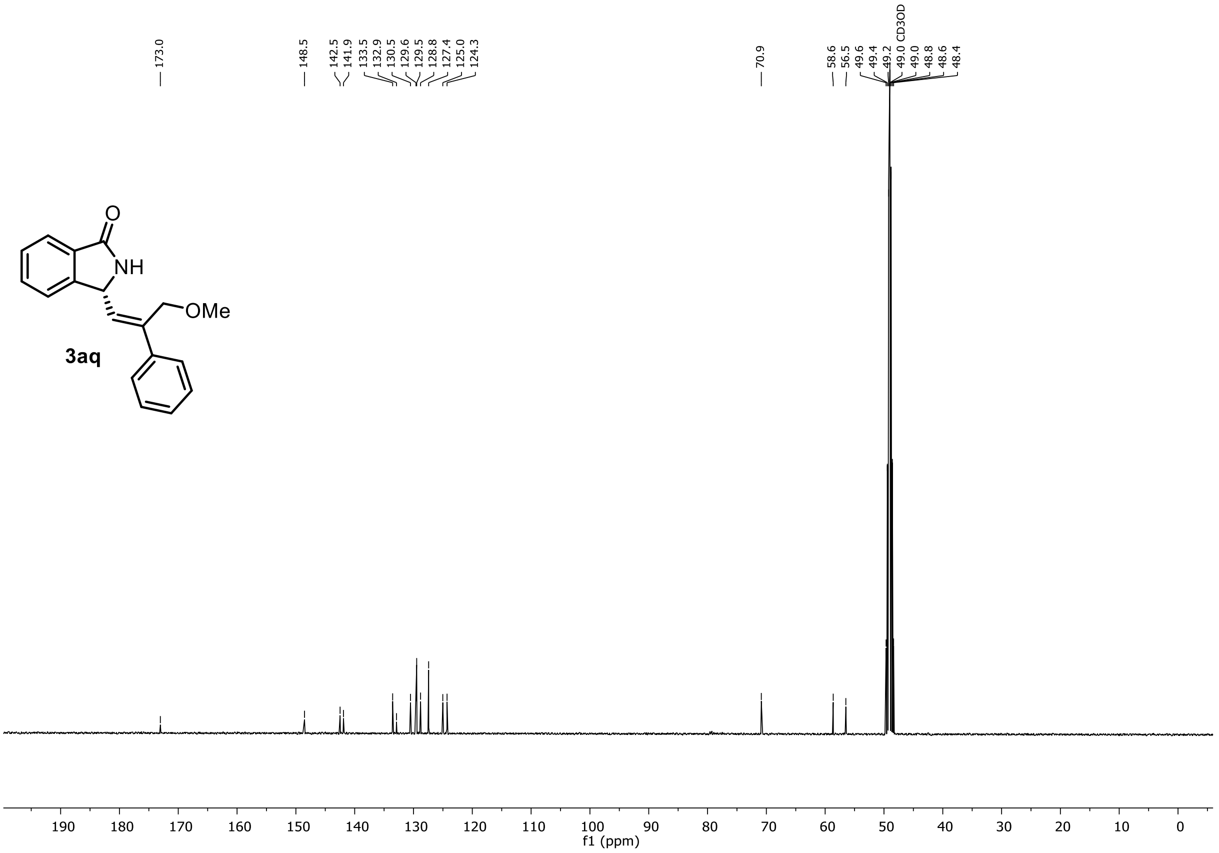

^{13}C NMR of **3aq** (101 MHz, Methanol-d$_4$)

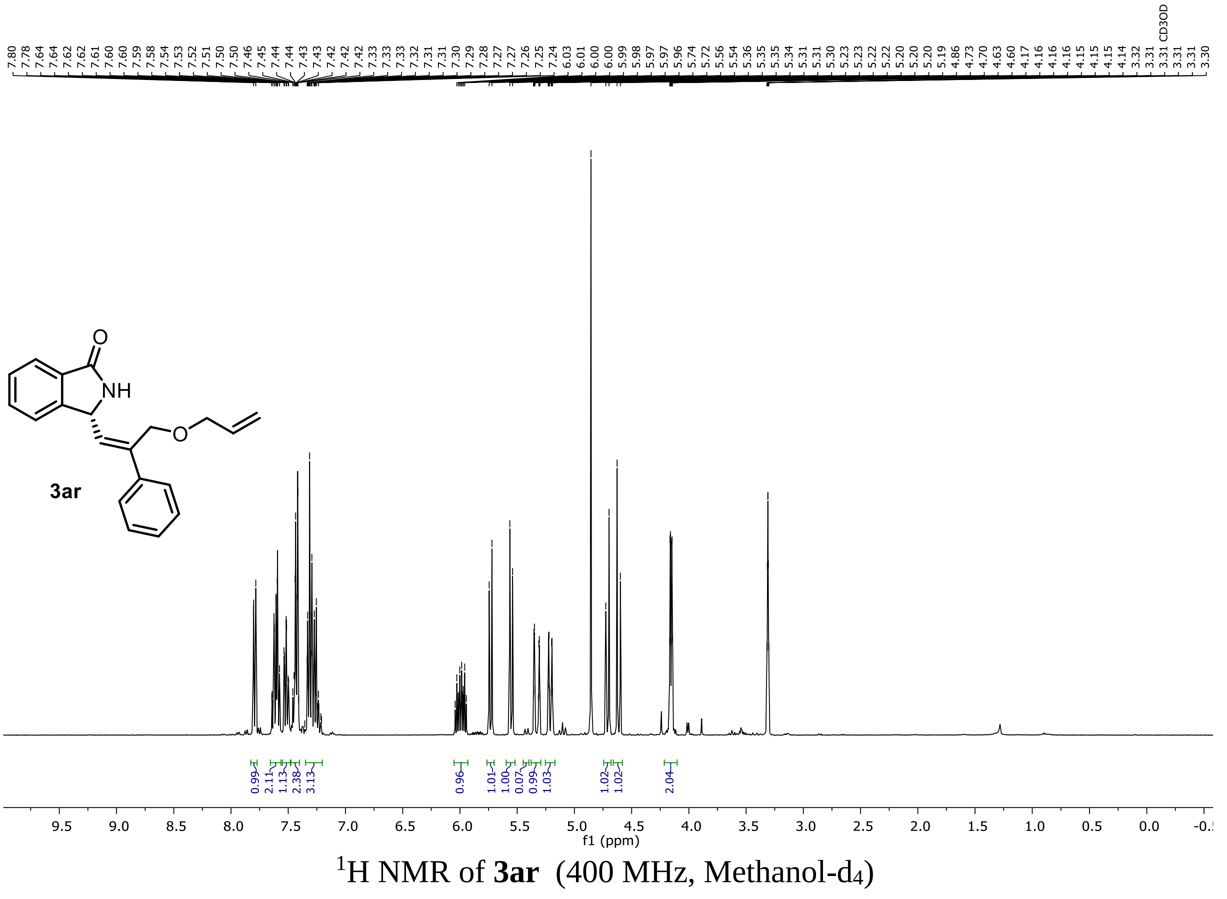

^{1}H NMR of **3ar** (400 MHz, Methanol-d$_4$)

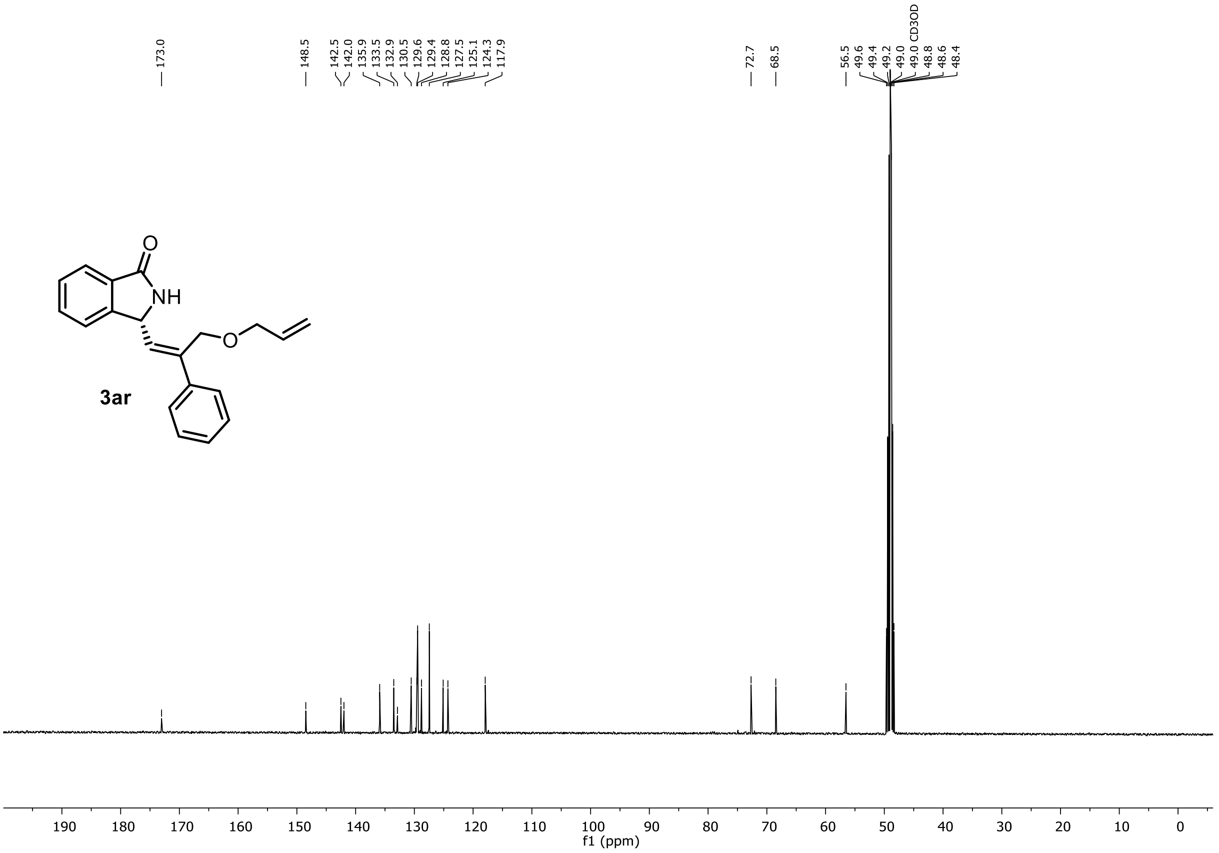

^{13}C NMR of **3ar** (101MHz, Methanol-d$_4$)

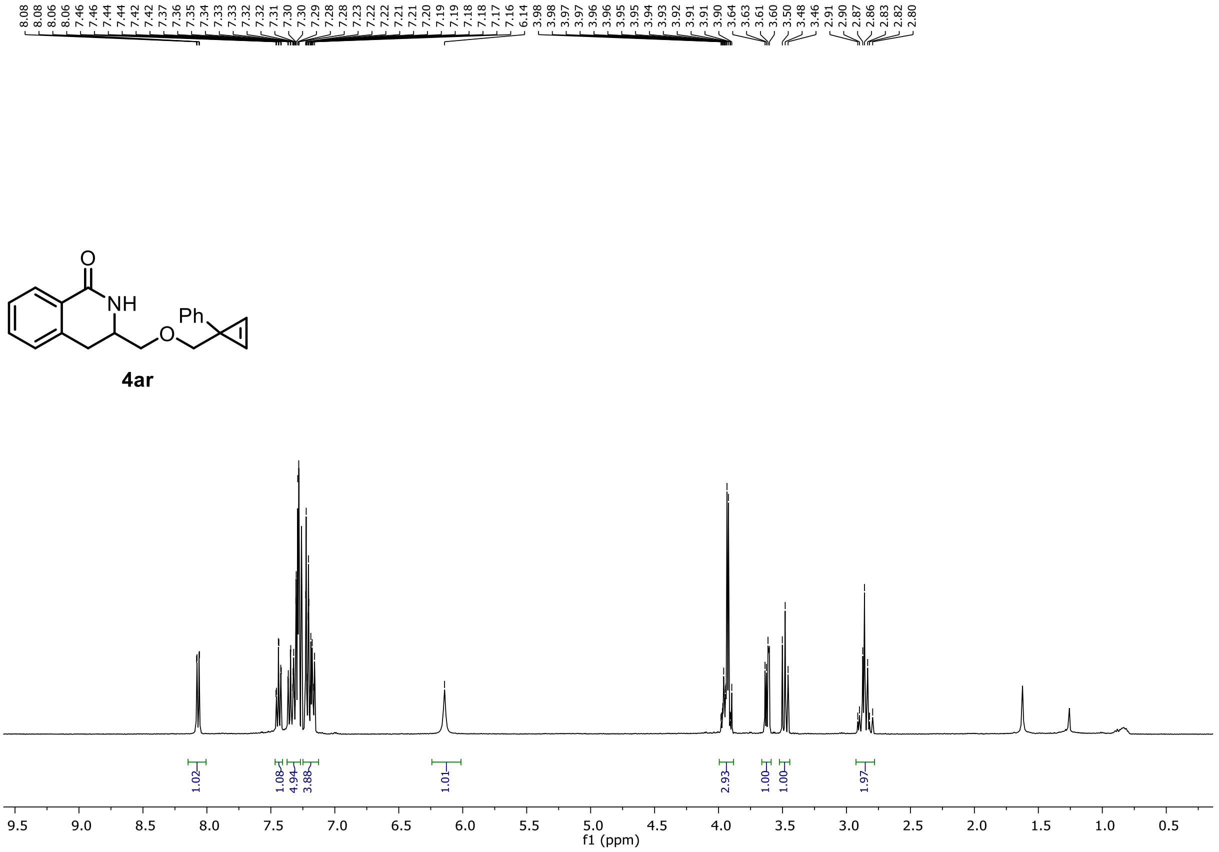

^{1}H NMR of **4ar** (400 MHz, CDCl$_3$)

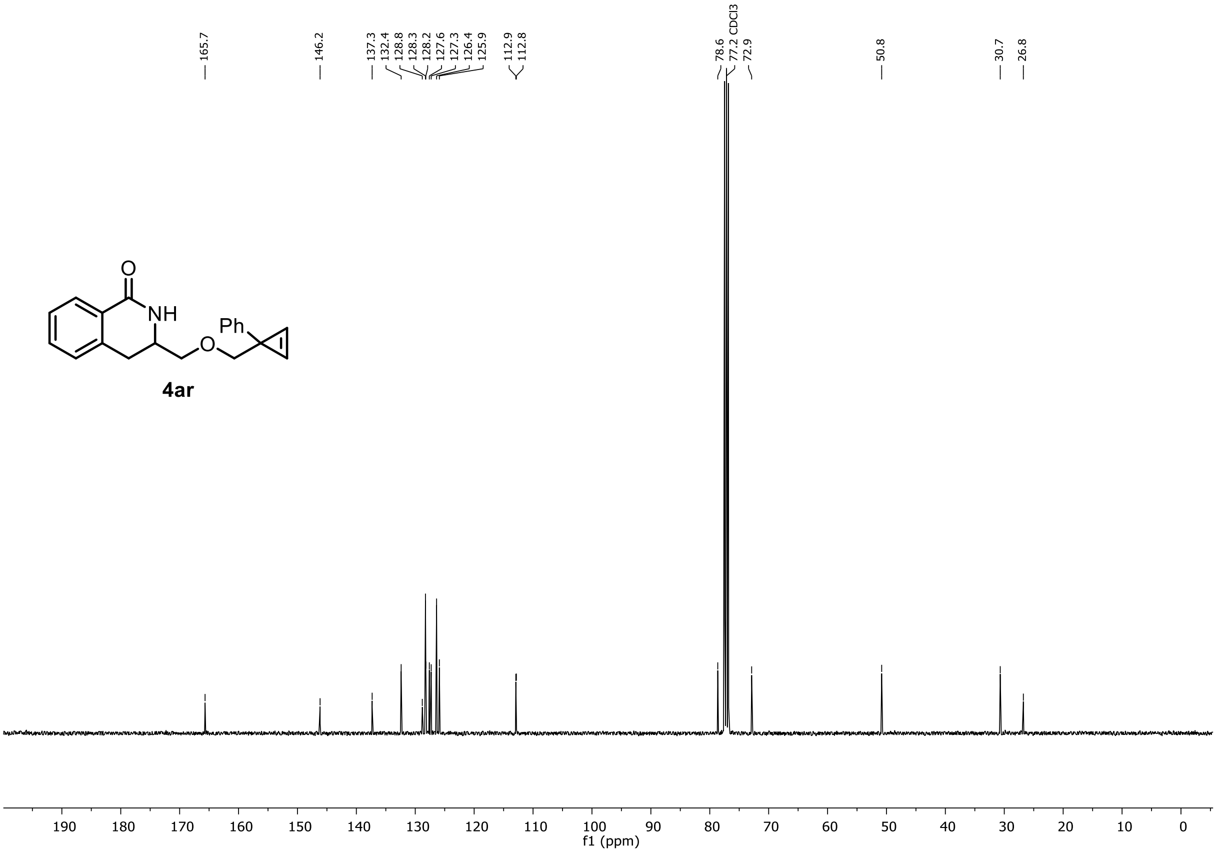

^{13}C NMR of 4**ar** (101 MHz, $CDCl_3$)

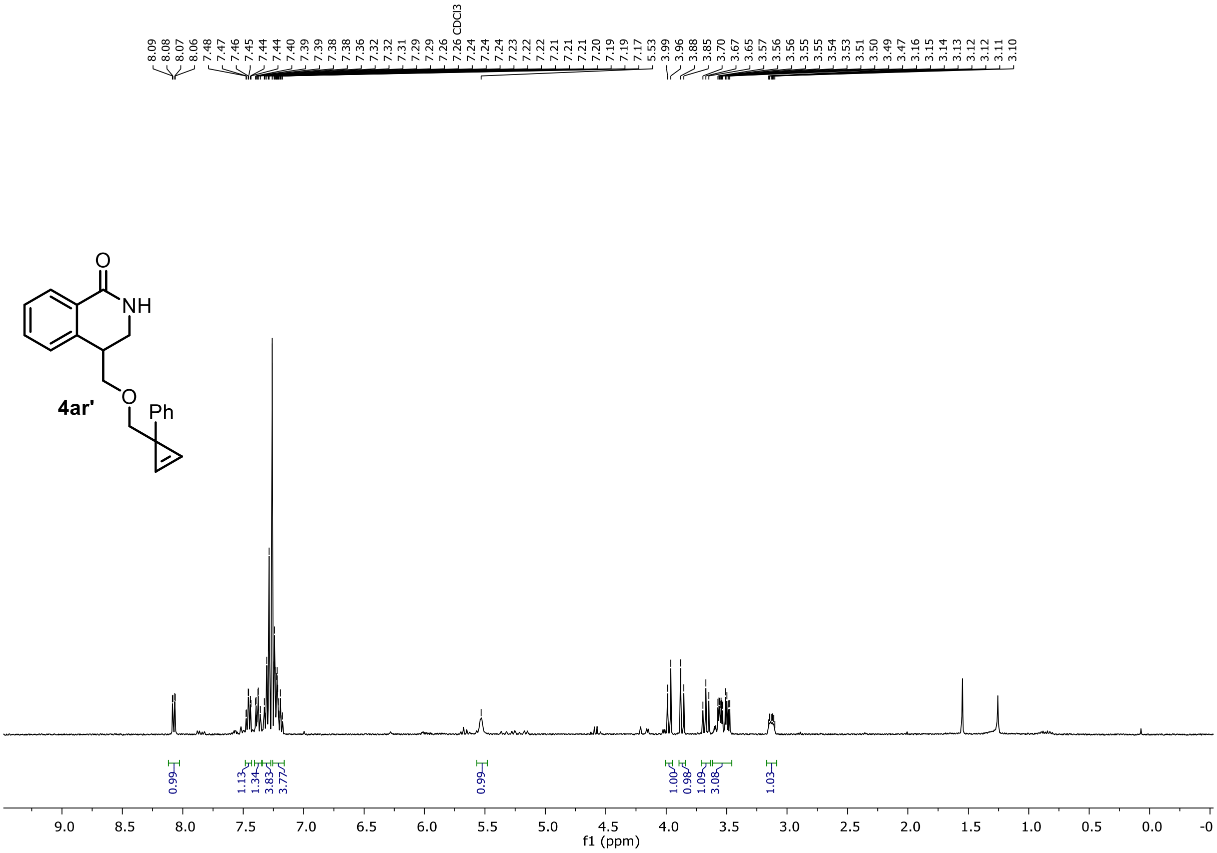

^{1}H NMR of **4ar'** (400 MHz, $CDCl_3$)

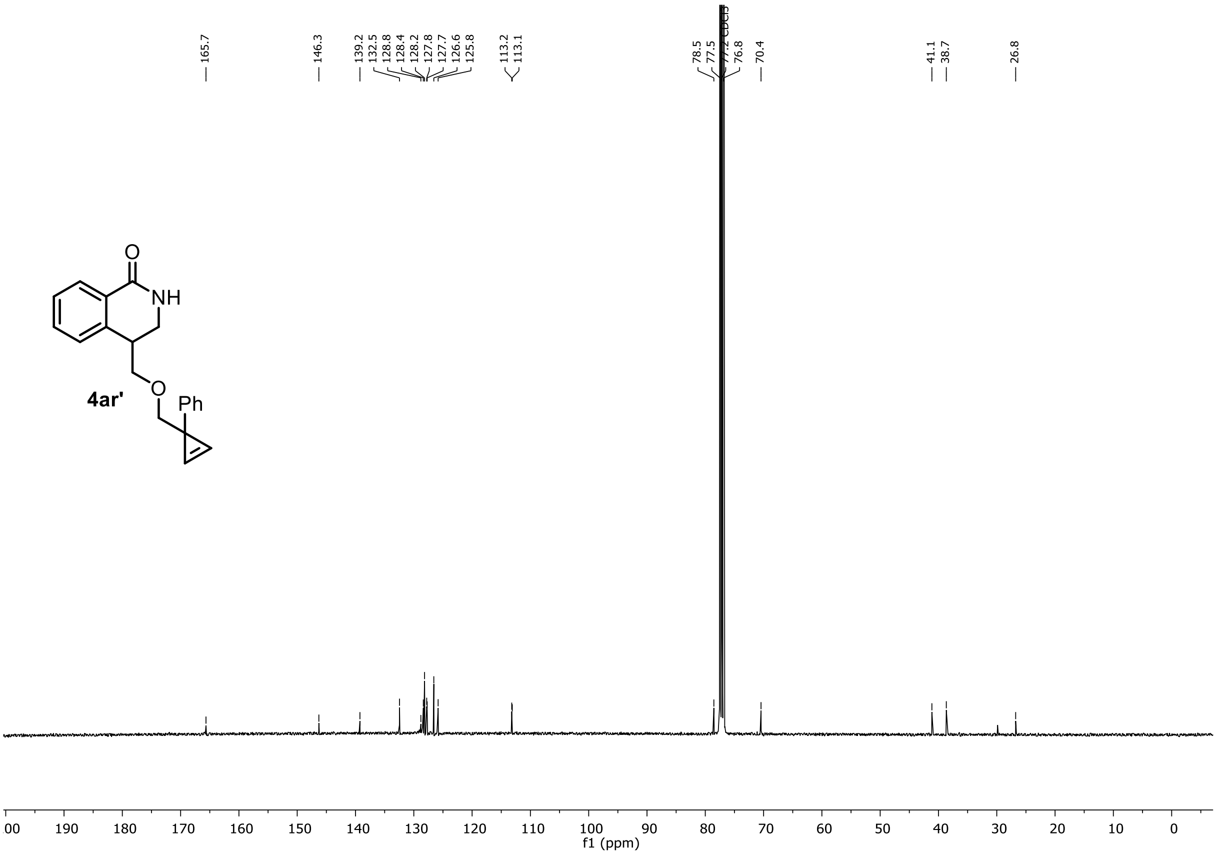

^{13}C NMR of **4ar'** (101 MHz, $CDCl_3$)

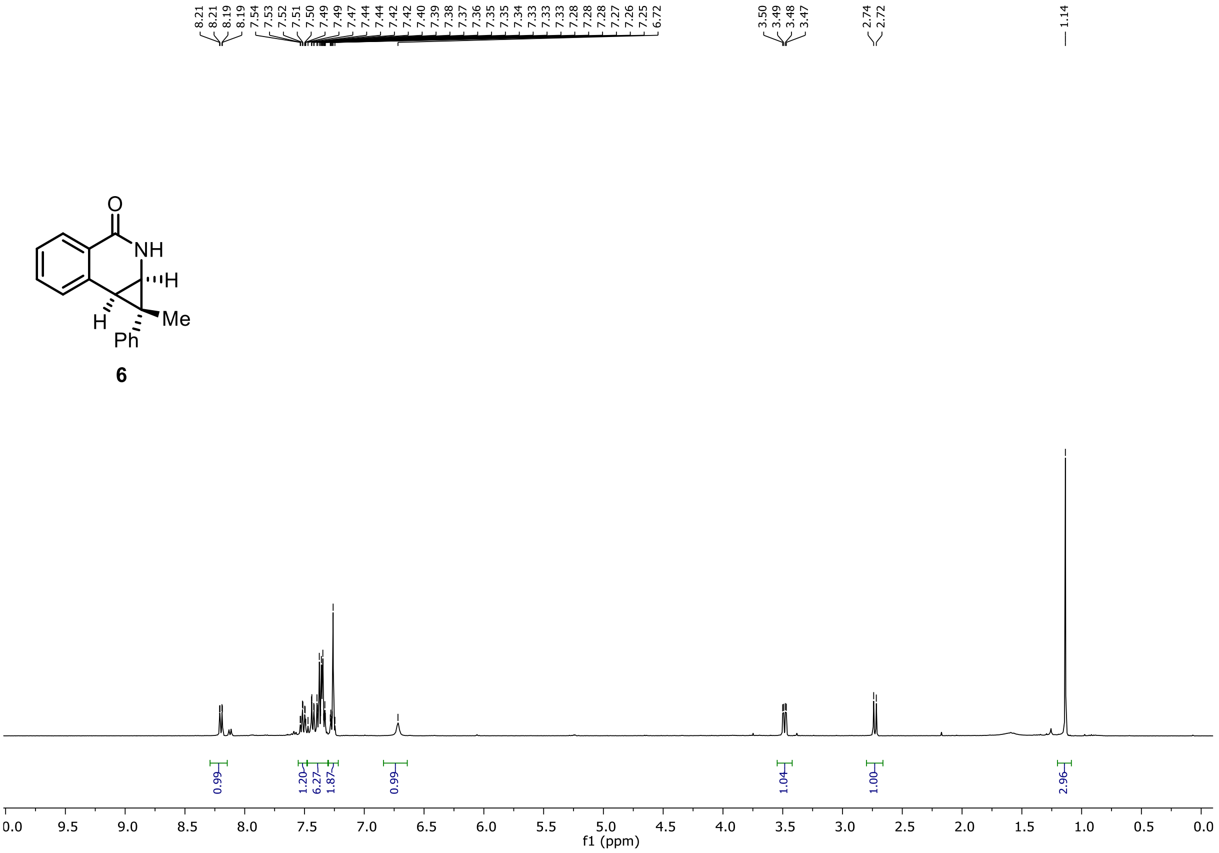

^{1}H NMR of **6** (400 MHz, $CDCl_3$)
